# Gold-catalyzed formal [4π + 2π]-cycloadditions of propiolate derivatives with unactivated nitriles†
†Electronic supplementary information (ESI) available. CCDC 1062512, 1062513 and 1062772. For ESI and crystallographic data in CIF or other electronic format see DOI: 10.1039/c5sc01950h


**DOI:** 10.1039/c5sc01950h

**Published:** 2015-07-20

**Authors:** Somnath Narayan Karad, Wei-Kang Chung, Rai-Shung Liu

**Affiliations:** a Department of Chemistry , National Tsing Hua University 101 , Sec. 2, Kuang-Fu Rd. , Hsinchu , 30013 , Taiwan . Email: rsliu@mx.nthu.edu.tw

## Abstract

Gold-catalyzed hetero-[4π + 2π]-cycloadditions of *tert*-butyl propiolates with unactivated nitriles are described; the resulting 6*H*-1,3-oxazin-6-ones are not easily accessible *via* conventional methods. This new finding enables a one-pot gold-catalyzed synthesis of highly substituted pyridines through sequential gold-catalyzed reactions of *tert*-butyl propiolates with nitriles, and then with electron-deficient alkynes in the same solvent. The utility of these [4 + 2]-cycloadditions is further expanded with various aldehydes, ketones and 2-phenyloxetane, yielding satisfactory yields of cycloadducts.

## Abstract 2

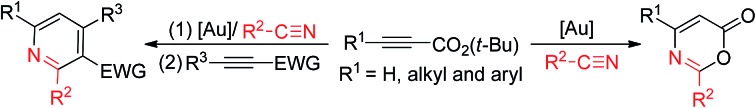
Gold-catalyzed hetero-[4π + 2π]-cycloadditions of *tert*-butyl propiolates with unactivated nitriles are described. This new finding enables a one-pot gold-catalyzed synthesis of highly substituted pyridines.

## Introduction

Metal-catalyzed [4π + 2π]-cycloadditions are powerful tools for the construction of carbo- or heterocyclic frameworks.^1^,^2^ Although common nitriles and alkynes represent common triple bond motifs, nitriles are generally less reactive than alkynes in catalytic [4π + 2π]-cycloadditions; the chemical stability of nitriles is reflected by their bond energy (854 kJ mol^–1^), being larger than that of alkynes (835 kJ mol^–1^).^3^ For instance, thermal [4π + 2π]-cycloadditions of dienes with unactivated nitriles required 600 °C (2 min) to give pyridine derivatives in 0.1–0.5% yields.4*a* In the context of catalytic [4π + 2π]-cycloadditions, not surprisingly, only one literature report documents both nitrile/1,3-diene and nitrile/1,3-enyne systems (eqn (1) and (2)).4b,c Ogoshi reported the first formal [4 + 2]-cycloadditions of common nitriles with dienes using Ni(0) catalysts (eqn (1)).4*b* Although Barluenga and Aguilar reported formal [4π + 2π]-cycloadditions of some 3-en-1-ynes with unactivated nitriles,4*c* such highly functionalized 3-en-1-ynes (X = *cis*-unsaturated ester, Z = alkoxy) are too specialized to reflect the reaction generality (eqn (2)). The [4π + 2π]-nitrile cycloadditions still remain an unsolved task for O- and N-substituted analogues of 1,3-dienes and 1,3-enynes (X = O, NR′, eqn (1) and (2)).^5^ In a significant advance, we here report the gold-catalyzed formal hetero-[4π + 2π]-cycloadditions^6^,^7^ of various propiolates with nitriles to afford 6*H*-1,3-oxazin-6-ones efficiently (eqn (3)).^8^ These findings enable the development of new cascade cycloadditions using three π-motifs including propiolates, nitriles and alkynes, yielding highly substituted pyridine derivatives. Notably, 6*H*-1,3-oxazin-6-ones are useful intermediates in various organic reactions whereas highly substituted pyridines are important structural cores commonly found in many bioactive molecules (see ESI Fig. S1†);^9^,^10^ their availability from convenient *t*-butyl propiolates increases the synthetic utility of this gold catalysis.1
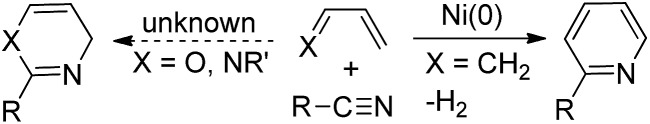

2
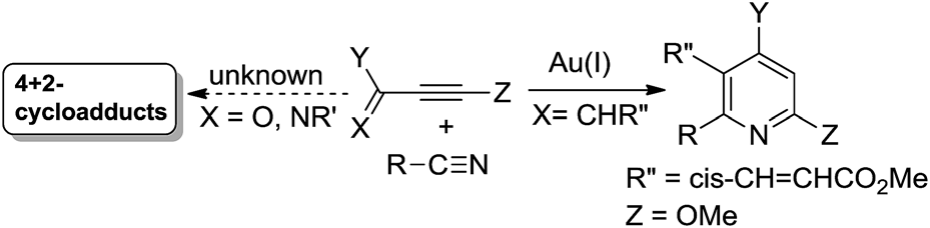

3
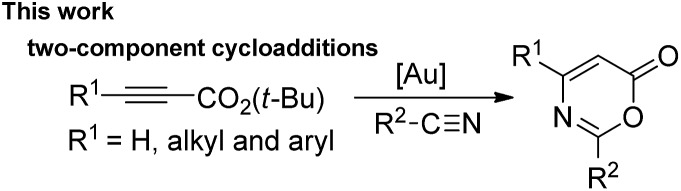

4
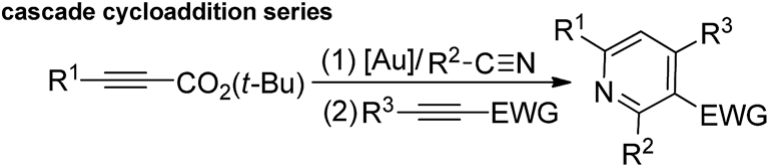



## Results and discussion

We envisage that direct [4π + 2π]-cycloadditions of propiolate derivatives with nitriles provide the most convenient synthesis of 6*H*-1,3-oxazin-6-ones such as **3**; the current procedures rely mainly on thermal rearrangement of *N*-acyl β-lactams.8a–d To test the feasibility, as shown in Table 1, *tert*-butyl hept-2-ynoate (**1a**, 1 equiv.) was treated with benzonitrile **2a** (3 equiv.) and AuCl_3_ (5 mol%) in hot DCE (70 °C, 16 h), affording the desired product **3a** in only a small yield (5%) together with the initial **1a** in 45% recovery (entry 1). The use of PPh_3_AuCl/AgSbF_6_ significantly increased the yield of the desired **3a** to 51% (entry 2). We also examined other cationic gold catalysts (5 mol%) including IPrAuCl/AgSbF_6_ and P(*t*-Bu)_2_(*o*-biphenyl)AuCl/AgSbF_6_, yielding compound **3a** in 64% and 85% yields, respectively (see entries 3 and 4). With the alteration of the silver salts as in P(*t*-Bu)_2_(*o*-biphenyl)AuCl/AgX (X = NTf_2_ and OTf), the product yields slightly decreased to 77% and 72%, respectively (entries 5 and 6). AgSbF_6_ (70 °C, 24 h) and Zn(OTf)_2_ (19 h) were found to be inactive in DCE, leading to a recovery of the starting compound **1a** in 72–75% yield (entries 7 and 8). The use of In(OTf)_3_, Sc(OTf)_3_ and TfOH in DCE gave hept-2-ynoic acid **1a′** in 65–72% yield and amide species **2a-H** (25–35% yield) along with unreacted starting compound **1a** (5–15% yield, entries 9–11). The yields of compound **3a** varied with the solvents (70 °C), with 65% in toluene (22 h), 82% in C_6_H_5_Cl (18 h) and 56% in 1,4-dioxane (19 h, entries 12–14).

**Table 1:** Tests of propiolate derivatives with gold catalysts

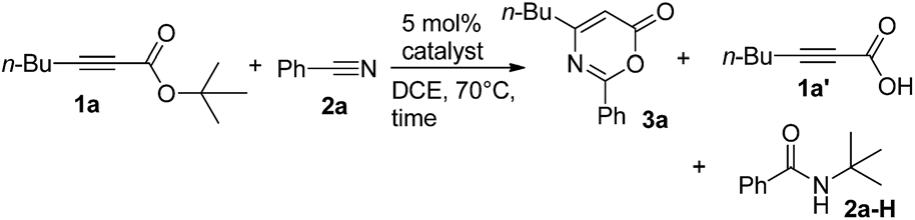							
Entries	Catalyst	Solvent	Time (h)	Yields^*a*^ ^,^^*b*^ (%)			
				**1a**	**3a**	**1a′**	**2a-H**
1	AuCl_3_	DCE	16	45	5	—	—
2	Ph_3_PAuCl/AgSbF_6_	DCE	12	—	51	—	—
3	IPrAuCl/AgSbF_6_	DCE	19	—	64	—	—
4	LAuCl/AgSbF_6_	DCE	18	—	85	—	—
5	LAuCl/AgNTf_2_	DCE	20	—	77	—	—
6	LAuCl/AgOTf	DCE	22	—	72	—	—
7	AgSbF_6_	DCE	24	75	—		
8	Zn(OTf)_2_^*c*^	DCE	19	72	—	—	—
9	In(OTf)_3_^*c*^	DCE	18	15	—	72	35
10	Sc(OTf)_3_^*c*^	DCE	22	10	—	65	32
11	HOTf^*c*^	DCE	15	5	—	67	25
12	LAuCl/AgSbF_6_	Toluene	22	—	65	—	—
13	LAuCl/AgSbF_6_	C_6_H_5_Cl	18	—	82	—	—
14	LAuCl/AgSbF_6_	1,4-Dioxane	19	—	56	—	—

^*a*^[**1a**] = 0.18 M.

^*b*^Product yields are reported after purification using a silica column. IPr = 1,3-bis(diisopropyl phenyl)-imidazol-2-ylidene, L = P(*t*-Bu)_2_(*o*-biphenyl), Tf = trifluoromethansesulfonyl.

^*c*^Reactions carried out at room temperature.

Table 2 assesses the reaction generality using various propiolate derivatives with varied nitriles. We first examined the reactions with unsubstituted propiolate species **1b**; its cycloaddition with benzonitrile **2a** proceeded smoothly to form the formal cycloadduct **3b** in 65% yield (entry 1). The reaction scope is extensible to aliphatically substituted propiolate species **1c–1e** (R = isopropyl, cyclopropyl and cyclohexyl), yielding the desired products **3c–3e** in satisfactory yields (77–85%, entries 2–4). This formal cycloaddition is also applicable to alkenyl-substituted propiolate **1f** to afford the corresponding product **3f** in 68% yield (entry 5). We tested the reactions on various phenyl-substituted propiolate species **1g–1j** bearing various *para*-substituents (X = H, OMe, F and Cl); their resulting cycloadducts **3g–3j** were obtained in satisfactory yields (65–72%, entries 6–9). We performed an X-ray diffraction study of product **3g** to confirm its molecular structure.^11^ We also prepared 2- and 3-thienyl-substituted propiolate derivatives **1k** and **1l**; their reactions with benzonitrile afforded cycloadducts **3k** and **3l** in reasonable yields (entries 10 and 11, 55–58%). Entries 12–15 show the tests of *tert*-butyl hept-2-ynoate **1a** with benzonitriles **2b–2e** bearing various *para*-substituents (X = OMe, Me, CO_2_Me, Cl) that afforded the desired cycloadducts **3m–3p** in satisfactory yields (62–76%). These catalytic cycloadditions were compatible with disparate nitriles including cyclohexyl nitrile (**2f**), cinnamonitrile (**2g**) and 3-thienyl nitrile (**2h**), affording the expected products **3q–3s** in satisfactory yields (66–78%, entries 16–18).

**Table 2:** Formal cycloadditions of various propiolates with nitriles

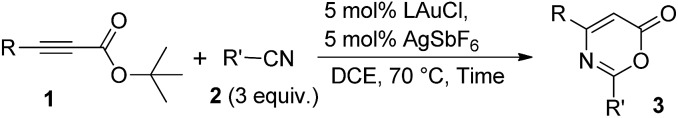
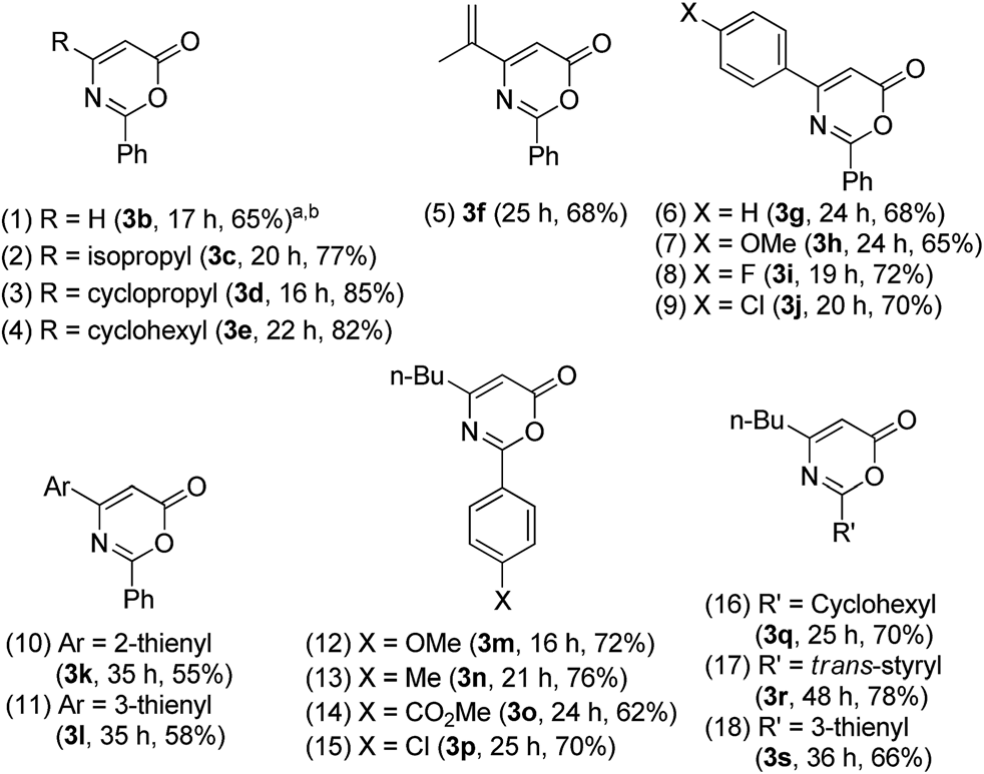

^*a*^ **2** (3 equiv.), [**1**] = 0.18 M.

^*b*^Product yields are reported after purification using a silica column. L = P(*t*-Bu)_2_(*o*-biphenyl).

As inferred from the chemistry of 2*H*-pyran-2-ones,^12^,^13^ one representative compound **3a** (1 equiv.) was treated with diethyl but-2-ynedioate (4 equiv.) in hot *p*-xylene (150 °C, 10 h) to afford tetrasubstituted pyridine **5a** in 96% yield; this reaction sequence presumably proceeds with intermediate **I** that is prone to a loss of CO_2_ (eqn (5)). As chlorobenzene is also an effective solvent for such a nitrile/propiolate cycloaddition (Table 1, entry 9), we developed a one-pot reaction involving the prior heating of a chlorobenzene solution of propiolate derivative **1a**, benzonitrile (3 equiv.) and P(*t*-Bu)_2_(*o*-biphenyl) AuCl/AgSbF_6_ (5 mol%) at 70 °C (18 h) in a sealed tube to ensure a complete consumption of starting compound **1a**; to this solution was added diethyl but-2-ynedioate (4 equiv.) with further heating at 150 °C for 20 h. This one-pot process delivered the desired pyridine **5a** in 80% yield (eqn (6)). If the three reactants in the same proportions were heated together with a gold catalyst in hot chlorobenzene (150 °C, 20 h), the yield of **5a** was decreased to 38% yield.5
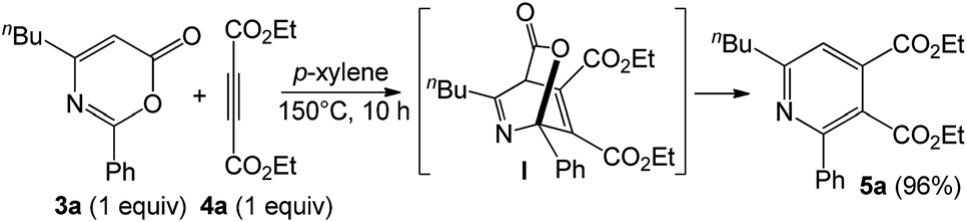

6
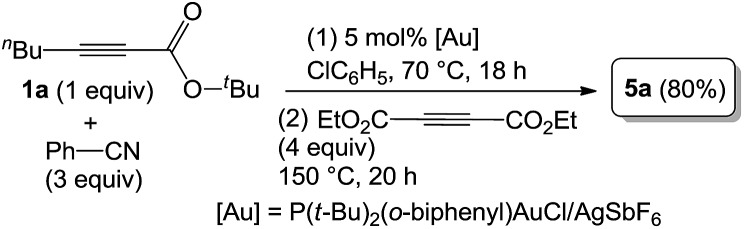



The easy operation of this one-pot reaction inspires us to examine the scope of the reaction using various propiolates, nitriles and alkynes; the results are summarized in Table 3. The procedures follow exactly that described in eqn (6). In the second stage of heating, the temperature is 150 °C for entries 1–7 and 180 °C for entries 8–12. Entry 1 shows the compatibility of these cycloadditions with unsubstituted propiolate derivative **1b** (R = H) that reacted sequentially with benzonitrile (**2a**) and diethyl but-2-ynedioate (**4a**) to yield the desired pyridine **5b** in 45% yield. We also tested the reactions on various alkyl-substituted propiolates **1c–1e** (R = isopropyl, cyclopropyl and cyclohexyl) that reacted with the same alkyne and benzonitrile to afford the desired pyridine species **5c–5e** in 73–76% yields (entries 2–4). The reaction is further applicable to aryl-substituted propiolates **1g** and **1l** (R = Ph, 3-thienyl) to deliver the desired pyridines **5f** and **5g** in 61% and 51% yield, respectively (entries 5 and 6). We tested the reactions of model propiolate (**1a**) and diethyl but-2-ynedioate (**4a**) with various nitriles (R^1^ = cyclohexyl, 3-thienyl and *trans*-styryl), affording the expected pyridine products **5h–5j** in satisfactory yields (68–75%, entries 7–9). The reactions were extensible to various unsymmetric alkynes **4b–4f** that reacted with propiolate (**1a**) and benzonitrile (**2a**) with excellent or high regioselectivity (entries 11–15). The reactions worked well for terminal alkynes **4b** (EWG = COOMe) and **4c** (EWG = COPh) to afford the desired pyridines **5k** and **5l** as single regioisomers, with respective yields of 83% and 76% (entries 10 and 11). For *n*-butyl propiolate **4d**, this one-pot sequence gave two inseparable isomeric products **5m**/**5m′** = 4/1, in a combined 82% yield (entry 12). For the other *n*-butyl and phenyl-substituted ynones **4e** and **4f** (EWG = COPh), their reactions afforded **5n** and **5o** with excellent regioselectivity and satisfactory yields (81–84%) (entries 13–14). The structures of representative compounds **5m** and **5n** were confirmed by proton NOE effects whereas the structure of cycloadduct **5o** was elucidated with an HMBC experiment (see ESI†).

**Table 3:** One-pot operations with nitriles, propiolates and alkynes

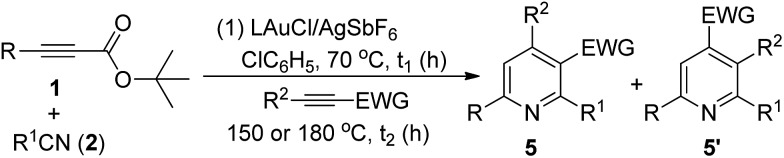
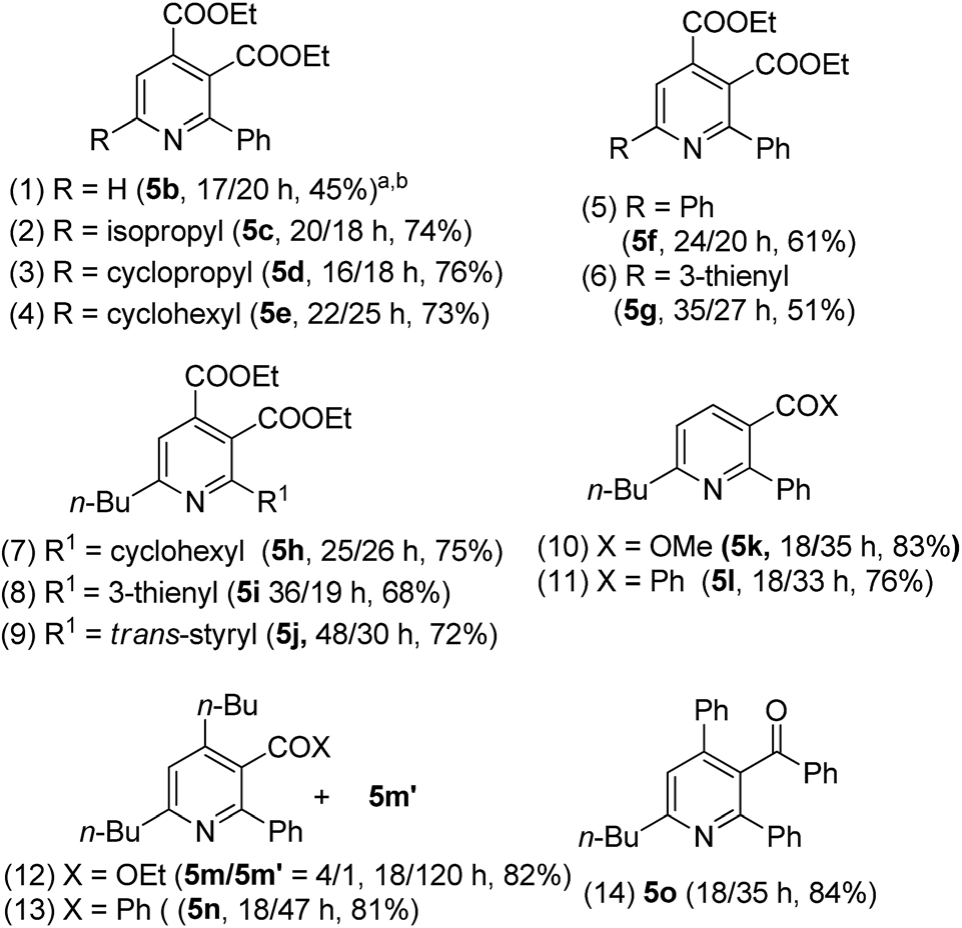

^*a*^5 mol% gold catalyst, L = P(*t*-Bu)_2_(*o*-biphenyl), R^1^CN (3 equiv.), R^2^CC–EWG (4 equiv.), 150 °C for entries 1–9 and 180 °C for entries 10–14.

^*b*^These data correspond to the reaction time *t*_1_/*t*_2_.

As nitriles are weakly nucleophilic, we envisage that aldehydes and ketones might be applicable substrates. To our pleasure, gold-catalyzed reactions of 3-phenylpropiolate **1g** with benzaldehyde, phenyl methyl ketone and acetone in hot dichloroethane (DCE) proceeded smoothly to afford formal cycloadducts **6a–6c** in high yields (86–89%, eqn (7)). The structure of compound **6a** was determined by X-ray diffraction.^11^ These carbonyl cycloadditions were also applicable to alkyl-substituted propiolates (**1a**) and (**1e**), yielding the desired compounds **6d** and **6e** in 87% and 77% yield, respectively (eqn (8)). Such a reaction was, notably, accessible to an eight-membered oxacyclic compound **6f** with 2.5 mol% 1,3-bis(diisopropyl phenyl)-imidazol-2-ylidene AuSbF_6_; it was isolated as a single regioisomer with 67% yield with 2-phenyloxetane (3 equiv.) and its molecular structure has been confirmed by X-ray diffraction.^11^ The compatibility of this gold catalysis with aldehydes, ketones and oxetanes truly reflects a broad applicability of these cycloadditions.7
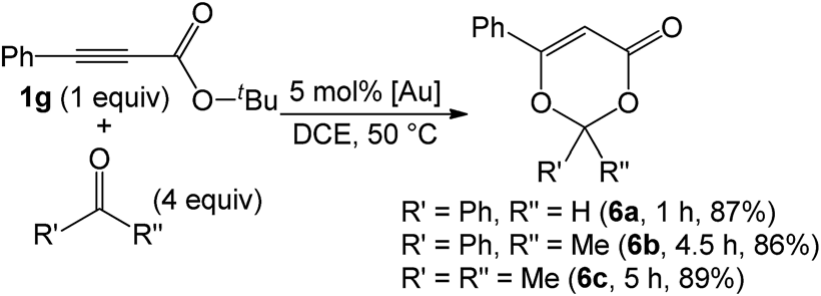

8
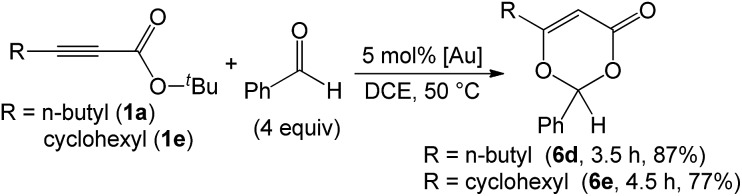

9
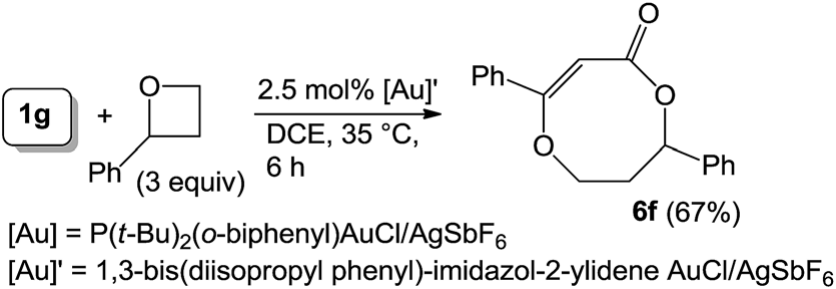



Prior to this work, Shin reported gold-catalyzed [4 + 2]-cycloadditions of alkenes with propiolic acid, which was, however, the only applicable substrate.6*a* Here, we employ diverse propiolate substrates to comply with not only nitriles but also aldehydes, ketones and oxetanes. To understand this discrepancy, we performed the reaction of 3-phenylpropiolic acid (**1g′**) with benzonitrile with the same gold catalyst in DCE, but the yield of the desired compound **1g** was only 8%, much smaller than that (68%) of its *tert*-butoxy derivative **1g** (Table 2, entry 5). Clearly, prior transformations of *t*-butoxy propiolates to the propiolate acids do not occur in the course of the reactions. For ethyl propiolate **1g′′**, its corresponding reaction with benzonitrile gave the amide-addition product **3g′** in 68% yield (eqn (10)); under this condition, benzonitrile was not effectively transformed into benzamide with this gold catalyst.^14^10
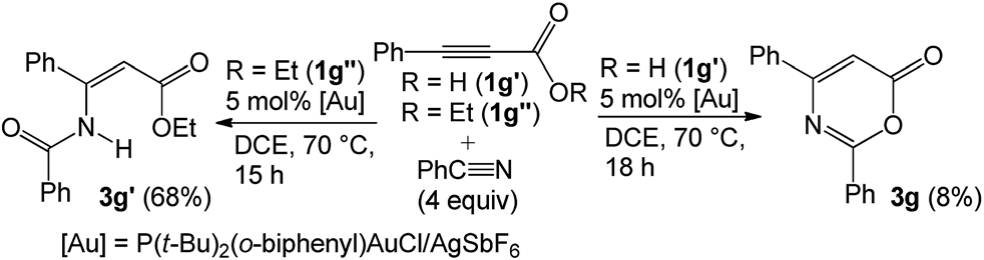



**Scheme 1:**
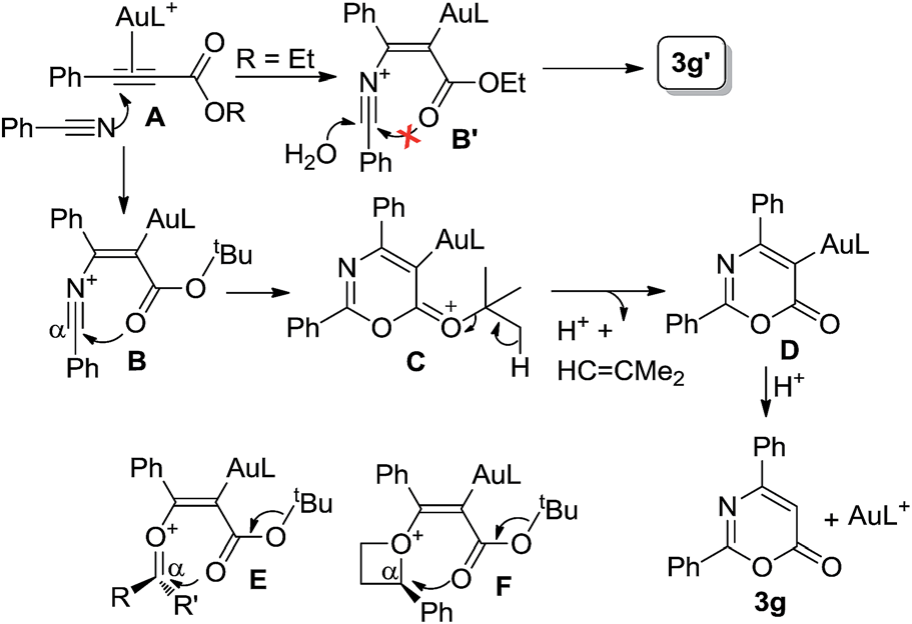
A postulated reaction mechanism.

The control experiments in eqn (10) indicate a mechanism involving a prior formation of nitrilium species **B***via* a π-alkyne activation, proceeding with an attack of nitrile at the gold-π-alkyne species **A**. As shown in Scheme 1, we postulate that the *tert*-butyoxy group of species **B** increases the nucleophilicity of a carbonyl group to attack this nitrilium moiety efficiently. This process releases a *tert*-butyl cation to induce a demetalation to form the observed cycloadduct **3g**. Beside nitriles, various aldehydes, ketones and oxetanes are more reactive than alkenes upon comparison of their applicable propiolates. We postulate that these nucleophiles generate intermediates **B**, **E** and **F** bearing a large positive charge on the reacting C_α_-carbons because of their adjacent oxonium and ammonium centers. We envisage that the propiolate cycloadditions match well with those nucleophiles that can develop highly polarized carbocations through π-alkyne activations.

## Conclusions

Unactivated nitriles are known to be stable triple-bond species, and their [4 + 2]-cycloadditions with 4π-bond motifs and other small molecules have few successful examples.^15^ This work reports the hetero-[4π + 2π]-cycloadditions of *tert*-butyl propiolates and nitriles catalyzed by gold catalysts. Such formal cycloadditions are applicable to diverse *tert*-butyl propiolates and nitriles, yielding useful 6*H*-1,3-oxazin-6-ones, which are not readily prepared with current methods.^8^ This new finding enables a one-pot gold-catalyzed synthesis of highly substituted pyridines through sequential reactions of *tert*-butyl propiolates with nitriles, and then with electron-deficient alkynes in the same solvent. The utility of these [4 + 2]-cycloadditions is further expanded with various aldehydes, ketones and 2-phenyloxetane, yielding satisfactory yields of cycloadducts. This work provides a new version of *tert*-butyl propiolates that feature useful four-atom building blocks with polar π-bond motifs such as nitriles, aldehydes and ketones, although their reactions with alkenes were reported to be restrictive.^8^

## Supplementary Material

Supplementary information

Crystal structure data
