# Supplementary material for: Gold-catalyzed formal [4π + 2π]-cycloadditions of propiolate derivatives with unactivated nitriles
Source: Chem Sci. 2015 Jul 20;6(10):5964–8. doi: 10.1039/c5sc01950h (PMC5950837; doi:10.1039/c5sc01950h)
Supplement: Supplementary file 1 [file SC-006-C5SC01950H-s001.pdf]

Electronic Supplementary Material (ESI) for Chemical Science.
This journal is © The Royal Society of Chemistry 2015

Supporting Information

Gold-catalyzed Formal [4π+2π]-Cycloadditions of Propiolate Derivatives with Unactivated Nitriles

Somnath Narayan Karad, Wei-Kang Chung and Rai-Shung Liu*

Department of Chemistry, National Tsing-Hua University, Hsinchu Taiwan, ROC
E-mail: rsliu@mx.nthu.edu.tw

Contents:

(1) Figure S1: Bioactive molecules containing 6*H*-1,3-oxazin-6-one and pyridine cores --1

(2) Representative synthetic procedures ---2

(3) Spectral data for key compounds (**1a-1k**, **3a-3s, 5a-5o, 6a-6f, 3g'**) --------------------6

(4) NOE effects for compounds **5m**, **5n** and **3g'**---26

(5) X-ray crystallographic structure and data for compound **3g, 6a, 6f**---------------------28

(6) ^{1}H and ^{13}C spectra of key compounds (**1a-1k**, **3a-3s, 5a-5o, 6a-6f, 3g')**---------------46

(1) Figure S1: Bioactive molecules pyridine cores

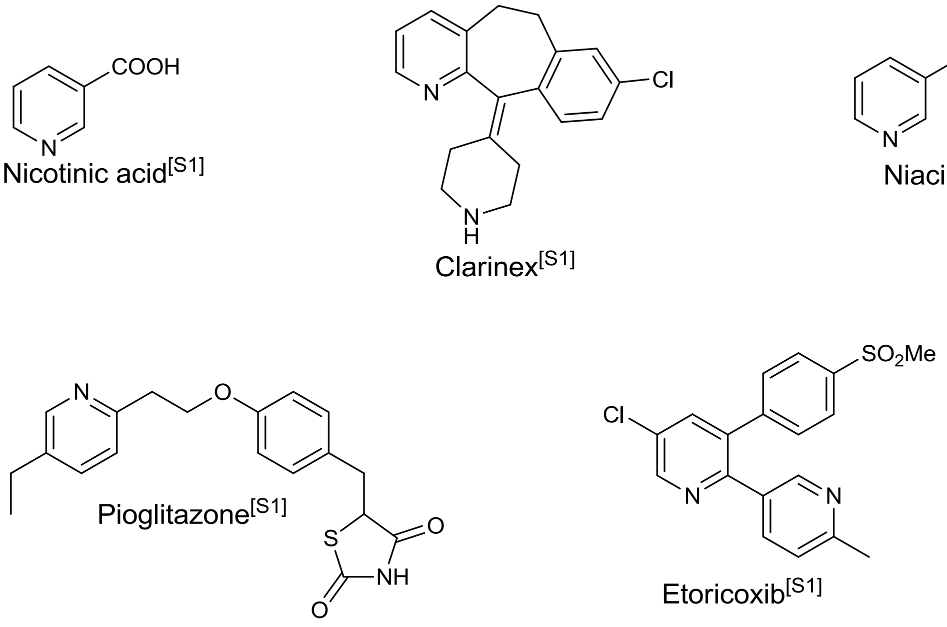

[S1] a) M. Baumann and I. R. Baxendale, *Beilstein J. Org. Chem.* 2013, **9**, 2265–2319; b) A. E. Goetz and N. K. Garg, *Nature Chemistry* 2013, **5**, 54–60.

[S2] a) Y. Yasuhara, T. Nishimura and T. Hayashi, *Chem. Commun.* 2010, **46**, 2130-2132; b) D. H. Wadsworth, S. M. Geer and M. R. Detty, *J. Org. Chem.* 1987, **52**, 3662-3668; c) S. Vercruysse, L. Cornelissen, F. Nahra, L. Collard and O. Riant, *Chem. Eur. J.* 2014, **20,** 1834-1838; d) H. Gao and J. Zhang, *Chem. Eur. J.* 2012, **18**, 2777–2782.

(2) Representative Synthetic procedures:

(a) General procedure:

Unless otherwise noted, all reactions were carried out under a nitrogen atmosphere in oven-dried glassware using standard syringe, cannula and septa apparatus. Tetrahydrofuran and hexanes were dried with sodium, benzophenone and distilled before use. Dichloromethane and DCE were dried over CaH_2 and distilled before use. Methanol and triethylamine (Et_3N) were stored over 4Å molecular sieves prior to use. Reagents were

purchased from commercial sources and used without purification, unless otherwise stated. Reactions were magnetically stirred and monitored by thin layer chromatography carried out on 0.25 mm E. Merck silica gel plate (60f- 254) using UV light as visualizing agents and ethanolic solution of phosphomolybdic acid, and heat as deveoling agents. ^{1}H NMR and ^{13}C NMR spectra were recorded on a Bruker 400, Varian 500 MHz and a Bruker 600 MHz spectrometers using chloroform-*d* ($CDCl_3$) as the internal standard.

(b) Preparation of *tert*-butyl 3-phenylpropiolate (1a). [S2]

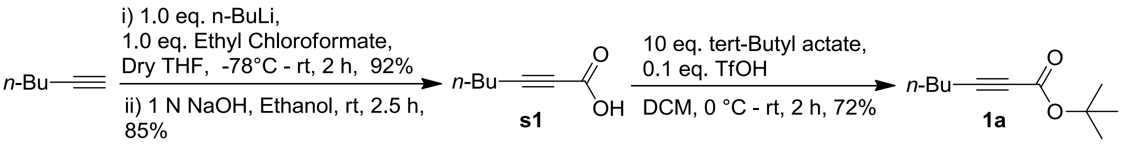

To a solution of 1-hexyne (1.0 g, 12.19 mmol) in dry THF (25 mL) at -78 ˚C was added *n*-BuLi (4.95 mL, 2.5 M in hexanes, 12.19 mmol), and reaction mixture was stirred for 30 min at -78 ˚C. Ethyl chloroformate (1.16 mL, 12.19 mmol) was then added, and the reaction mixture was warmed room temperature for additional stirring for 1.5 h. The reaction was quenched with ice cold water (100 mL) and extracted with Et_2O (3 x 100 mL). The organic layers were then combined and washed with brine (100 mL), and then dried over $MgSO_4$. The resulting organic layer was concentrated under reduced pressure, and the crude product was purified by flash chromatography on silica column (Hexanes/Ethyl acetate as a mobile phase) to afford 1.72g of ethyl hept-2-ynoate (**1a**) (92% yield, 11.21 mmol) as a colorless oil.

To a solution of ethyl hept-2-ynoate (1.0 g, 6.49 mmol) in 45 mL of ethanol was added slowly with stirring an aqueous sodium hydroxide solution (25 mL, 1 N). After 2.5 h, the reaction mixture was diluted with water (50 mL) and was washed with dichloromethane (2 X 25 mL). The aqueous phase was acidified with 20% HCl solution and was extracted with dichloromethane (3 X 50 mL). The combined extracts were dried over $MgSO_4$ and concentrated under reduced pressure to give 686 mg of hept-2-ynoic acid (85% yield, 5.44 mmol).

To a solution of hept-2-ynoic acid (1.0 g, 7.93 mmol) in 75 ml of DCM at 0 °C was added *tert*-Butyl acetate (10.6 ml, 79.36 mmol) and TfOH (0.070 ml, 0.79 mmol) dropwise. The resulting solution was warmed room temperature to stirred for 2 hours and carefully washed with saturated $NaHCO_3$. The aqueous layer was extracted with DCM (3x100 ml) and the combined extracts were washed with saturated NaCl then dried over $MgSO_4$, filtered, and concentrated under reduced pressure to give crude product. The crude product was purified by column chromatography on silica using ethyl acetate in hexanes as a mobile phase to give *tert*-butyl hept-2-ynoate (**1a)** (1.04 g, 2.63 mmol, 72%) as a colourless oil.

Other *tert*-butyl 3-propiolates **(1b – 1l)** were prepared by using the same procedure as that of *tert*-butyl hept-2-ynoate **(1a)**. Propiolate (**1g"**) was prepared by reported procedure [S2]

(c) Typical procedure for standard catalytic operations:

i) Typical procedure for the synthesis of 4-butyl-2-phenyl-6*H*-1,3-oxazin-6-one (3a).

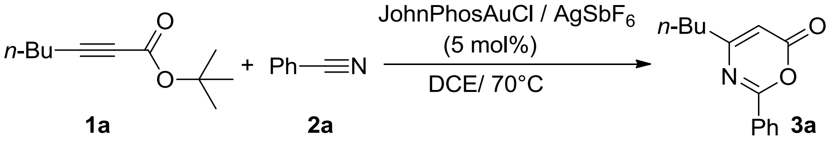

A two-neck flask was charged with chloro [(1,1′-biphenyl-2-yl)di-*tert*-butylphosphine] AuCl (14.6 mg, 0.0274 mmol) and silver hexafluoride (9.4 mg, 0.0274 mmol), and to this mixture was added dry DCE (1.0 mL). The resulting mixture was stirred at room temperature for 10 min. To this mixture was added a dry DCE solution (2 mL) of *tert*-butyl hept-2-ynoate (**1a)** (100 mg, 0.549 mmol) and benzonitrile (**2a**) (169 mg, 1.65 mmol) dropwise. The solution was stirred at 70 °C for 18 hours before filtration over a short celite bed. The solution was concentrated, and eluted through a silica column to give the desired 4-butyl-2-phenyl-6*H*-1,3-oxazin-6-one (**3a**) (106 mg, 0.467 mmol, 85 %) as colorless oil.

ii) Typical procedure for the synthesis of diethyl 6-butyl-2-phenylpyridine-3,4-dicarboxylate (5a).

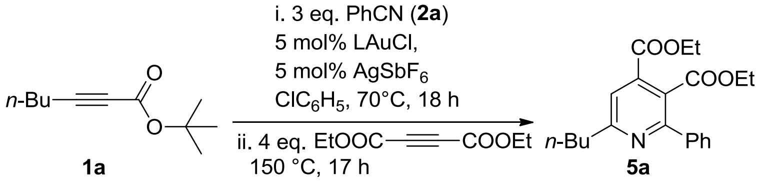

A sealed tube was charged with chloro[(1,1′-biphenyl-2-yl)di-*tert*-butylphosphine]AuCl (14.6 mg, 0.0274 mmol) and silver hexafluoride (9.4 mg, 0.0274 mmol), and to this mixture was added dry Chlorobenzene (1.0 mL). The resulting mixture was stirred at room temperature for 10 min. To this mixture was added a dry chlorobenzene solution (2 mL) of *tert*-butyl hept-2-ynoate (**1a**) (100 mg, 0.549 mmol) and benzonitrile (169 mg, 1.65 mmol) dropwise. After stirring at 70 °C for 18 hour, the resulting solution was added diethyl but-2-ynedioate (373 mg 2.13 mmol), and the reaction mixture was stirred at 150 °C for 20 h. The resulting mixture was filtered over a short celite bed, concentrated, and eluted through a silica column to give the desired diethyl 6-butyl-2-phenylpyridine-3,4-dicarboxylate **(5a)** (156 mg, 0.439 mmol, 80 %) as colorless oil.

iii) Typical procedure for the synthesis of 2,6-diphenyl-4*H*-1,3-dioxin-4-one (6a).

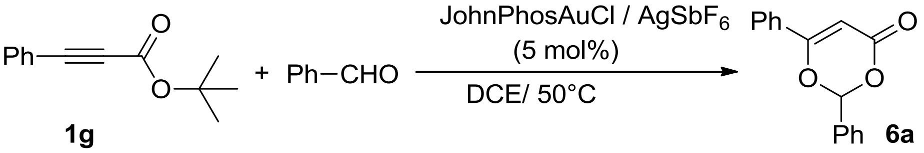

A two-neck flask was charged with chloro [(1,1′-biphenyl-2-yl)di-*tert*-butylphosphine] AuCl (13.1 mg, 0.0247 mmol) and silver hexafluoride (8.5 mg, 0.0247 mmol), and to this mixture was added dry DCE (1.0 mL). The resulting mixture was stirred at room temperature for 10 min. To this mixture was added a dry DCE solution (2 mL) of *tert*-butyl 3-phenylpropiolate (**1g)** (100 mg, 0.495 mmol) and benzaldehyde (209 mg, 1.98 mmol) dropwise. After stirring at 50 °C for 1 h, the reaction mixture was filtered over a short celite

bed, concentrated, and eluted through a silica column to give the desired 2,6-diphenyl-4*H*-1,3-dioxin-4-one (**6a**) (108 mg, 0.396 mmol, 87 %) as white solid.

Other cycloadducts **(6b-6e)** were prepared by using the same procedure as that of 2,6-diphenyl-4*H*-1,3-dioxin-4-one (**6a**).

iv) Typical procedure for the synthesis of (*Z*)-4,8-diphenyl-7,8-dihydro-1,5-dioxocin-2(6*H*)-one (6f).

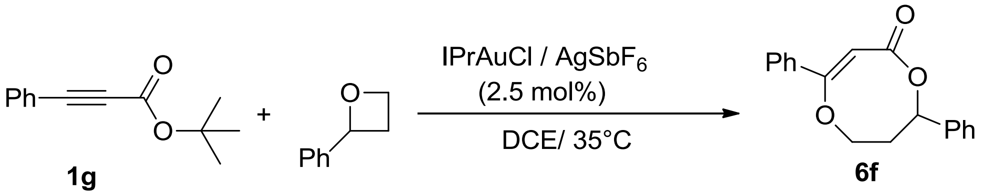

IPr = 1,3-bis(diisopropyl phenyl)-imidazol-2-ylidene

A two-neck flask was charged with 1,3-bis(2,6-diisopropylphenyl)imidazol-2-ylidene AuCl (7.68 mg, 0.0124 mmol) and silver hexafluoride (4.24 mg, 0.0124 mmol), and to this mixture was added dry DCE (1.0 mL). The resulting mixture was stirred at room temperature for 10 min. To this mixture was added a dry DCE solution (2 mL) of *tert*-butyl 3-phenylpropiolate (**1g**) (100 mg, 0.495 mmol) and freshly prepared 2-phenyloxetane (199 mg, 1.48 mmol) dropwise. After stirring at 35 ℃ for 6 h, the reaction mixture was filtered over a short celite bed, concentrated, and eluted through a silica column to give the desired (*Z*)-4,8-diphenyl-7,8-dihydro-1,5-dioxocin-2(6*H*)-one (**7a**) (93 mg, 0.331 mmol, 67 %) as white solid.

(3) Spectral data:

Spectral data for *tert*-butyl hept-2-ynoate (1a).

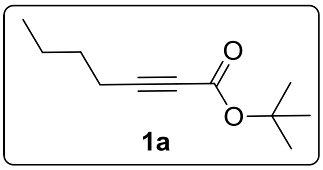

Colorless oil; ^{1}H NMR (600 MHz, $CDCl_3$): δ 2.27 (t, *J* = 7.2, 2 H), 1.54 ~ 1.49 (m, 2 H),

1.46 (s, 9 H), 1.43 ~ 1.35 (m, 2 H), 0.88 (t, J = 7.3 Hz, 3 H); ^{13}C NMR (150 MHz, $CDCl_3$): δ 153.0, 87.0, 82.8, 74.4, 29.6, 28.0, 21.9, 18.3, 13.4; HRMS calcd. for $C_{11}H_{18}O_2$: 182.1307; found: 182.1306.

Spectral data for *tert*-butyl propiolate (1b).

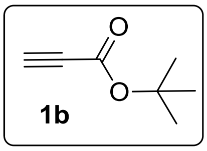

Colorless oil; ^1H NMR (600 MHz, $CDCl_3$): δ 2.74 (s, 1 H), 1.45 (s, 9 H); ^{13}C NMR (150 MHz, $CDCl_3$): δ 151.6, 84.0, 75.9, 72.2, 27.8; HRMS calcd. for $C_7H_{10}O_2$: 126.0681; found: 126.0681.

Spectral data for *tert* - butyl 4-methylpent-2-ynoate (1c).

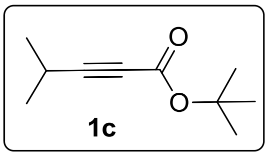

Colorless oil; ^1H NMR (600 MHz, $CDCl_3$): δ 2.66 ~ 2.59 (m, 1 H), 1.46 (s, 9 H), 1.19 (d, J = 6.6 Hz, 6 H); ^{13}C NMR (150 MHz, $CDCl_3$): δ 153.1, 91.5, 82.8, 73.7, 28.0, 21.8, 20.4; HRMS calcd. for $C_{10}H_{16}O_2$: 168.1150; found: 168.1155.

Spectral data for *tert*-butyl 3-cyclopropylpropiolate (1d).

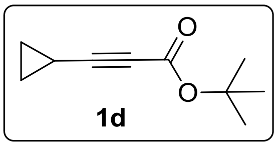

Colorless oil; ^1H NMR (600 MHz, $CDCl_3$): δ 1.40 (s, 9 H), 1.29 ~ 1.25 (m, 1 H), 0.83 ~ 0.80 (m, 4 H); ^{13}C NMR (150 MHz, $CDCl_3$): δ 152.7, 90.3, 82.6, 69.7, 27.9, 8.8, -0.81; HRMS calcd. for $C_{10}H_{14}O_2$: 166.0994; found: 166.1000.

Spectral data for *tert* -butyl 3-cyclohexylpropiolate (1e).

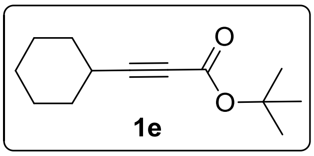

Colorless oil; ^{1}H NMR (600 MHz, $CDCl_3$): δ 2.43 ~ 2.39 (m, 1 H), 1.78 ~ 1.75 (m, 2 H), 1.66 ~ 1.64 (m, 2 H), 1.46 ~ 1.40 (m, 12 H), 1.27 ~ 1.22 (m, 3 H); ^{13}C NMR (150 MHz, $CDCl_3$): δ 153.1, 90.2, 82.6, 74.3, 31.4, 28.7, 27.9, 25.5, 24.6; HRMS calcd. for $C_{13}H_{20}O_2$: 208.1463; found: 208.1463.

Spectral data for *tert* -butyl 4-methylpent-4-en-2-ynoate (1f).

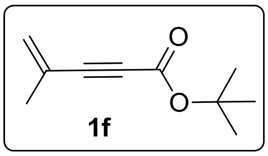

Colorless oil; ^{1}H NMR (600 MHz, $CDCl_3$): δ 5.53 ~ 5.52 (m, 1 H), 5.44 ~ 5.43 (m, 1 H), 1.89 (m, 3 H), 1.47 (s, 9 H); ^{13}C NMR (150 MHz, $CDCl_3$): δ 153.0, 126.9, 124.5, 84.7, 83.3, 80.8, 28.0, 22.4; HRMS calcd. for $C_{10}H_{14}O_2$: 166.0994; found: 166.0996.

Spectral data for *tert-* butyl 3-phenylpropiolate (1g).

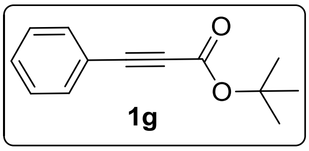

Colorless oil; ^{1}H NMR (600 MHz, $CDCl_3$): δ 7.55 ~ 7.53 (m, 2 H), 7.41 ~ 7.38 (m, 1 H), 7.34 ~ 7.31 (m, 2 H), 1.52 (s, 9 H); ^{13}C NMR (150 MHz, $CDCl_3$): δ 153.1, 132.8, 130.3, 128.5, 120.0, 83.8, 83.4, 82.0, 28.0; HRMS calcd. for $C_{13}H_{14}O_2$: 202.0994; found: 202.0984.

Spectral data for *tert*-butyl 3-(4-methoxyphenyl)propiolate (1h).

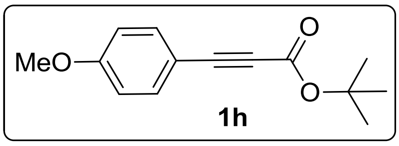

White solid; ^{1}H NMR (400 MHz, $CDCl_3$): δ 7.50 (d, *J* = 8.8 Hz, 2 H), 6.84 (d, *J* = 8.8 Hz, 2 H), 3.80 (s, 3 H), 1.52 (s, 9 H); ^{13}C NMR (100 MHz, $CDCl_3$): δ 161.2, 153.3, 134.6, 114.1,

111.6, 84.5, 83.1, 81.3, 55.2, 28.0; HRMS calcd. for $C_{14}H_{16}O_3$: 232.1099; found: 232.1096.

Spectral data for *tert*-butyl 3-(4-fluorophenyl)propiolate (1i).

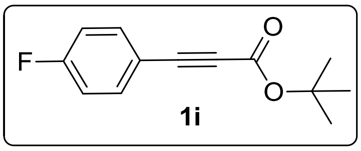

White solid; ^{1}H NMR (600 MHz, $CDCl_3$): δ 7.53 ~ 7.51 (m, 2 H), 7.03 ~ 7.00 (m, 2 H), 1.50 (s, 9 H); ^{13}C NMR (150 MHz, $CDCl_3$): δ 163.6 (d, *J* = 252.0 Hz), 152.9, 135.0 (d, *J* = 9.0 Hz), 115.9 (d, *J* = 22.5 Hz), 83.5, 82.6, 81.8, 28.0 (one carbon merged with others); HRMS calcd. for $C_{13}H_{13}FO_2$: 220.0900; found: 220.0903.

Spectral data for *tert*-butyl 3-(4-chlorophenyl)propiolate (1j).

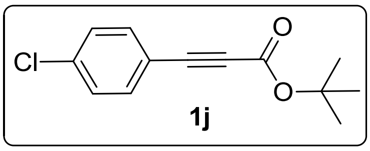

White solid; ^{1}H NMR (600 MHz, $CDCl_3$): δ 7.47 (d, *J* = 8.4 Hz, 2 H), 7.31 (d, *J* = 8.4 Hz, 2 H), 1.51 (s, 9 H); ^{13}C NMR (150 MHz, $CDCl_3$): δ 152.9, 136.6, 134.0, 128.9, 118.5, 83.7, 82.8, 82.4, 28.0; HRMS calcd. for $C_{13}H_{13}ClO_2$: 236.0604; found: 236.0601.

Spectral data for *tert*- butyl 3-(thiophen-2-yl)propiolate (1k).

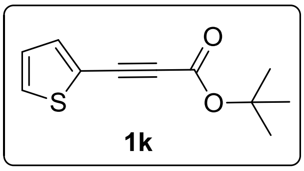

Colorless oil; ^{1}H NMR (600 MHz, $CDCl_3$): δ 7.67 (dd, *J* = 3.0, 1.2 Hz, 1 H), 7.26 (d, *J* = 5.1, 3.0 Hz, 1 H), 7.17 (d, *J* = 5.1, 1.2 Hz, 1 H), 1.50 (s, 9 H); ^{13}C NMR (150 MHz, $CDCl_3$): δ 153.1, 133.1, 130.1, 125.9, 119.1, 83.4, 81.9, 79.2, 28.0; HRMS calcd. for $C_{11}H_{12}O_2S$: 208.0558; found: 208.0559.

Spectral data for *tert*- butyl 3-(thiophen-3-yl)propiolate (1l).

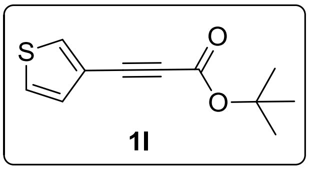

Colorless oil; ^{1}H NMR (600 MHz, $CDCl_3$): δ 7.39 ~ 7.37 (m, 2 H), 6.99 ~ 6.96 (m, 1 H), 1.49 (s, 9 H); ^{13}C NMR (150 MHz, $CDCl_3$): δ 152.9, 136.0, 130.6, 127.3, 119.6, 86.0, 83.5, 77.6, 27.9; HRMS calcd. for $C_{11}H_{12}O_2S$: 208.0558; found: 208.0550.

Spectral data for 4-butyl-2-phenyl-6*H*-1,3-oxazin-6-one (3a).

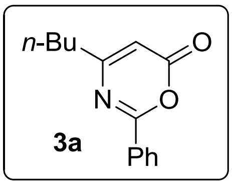

White solid; ^{1}H NMR (600 MHz, $CDCl_3$): 8.20 (dd, *J* = 8.4, 1.2 Hz, 2 H), 7.55 ~ 7.52 (m, 1 H), 7.45 ~ 7.43 (m, 2 H), 5.97 (m, 1 H), 2.50 (t, *J* = 7.5 Hz, 2 H), 1.68 ~ 1.63 (m, 2 H), 1.41 ~ 1.35 (m, 2 H), 0.92 (t, *J* = 7.5 Hz, 3 H); ^{13}C NMR (150 MHz, $CDCl_3$): δ 169.8, 162.9, 159.2, 133.1, 129.8, 128.6, 128.5, 105.0, 36.7, 29.0, 22.1, 13.7; HRMS calcd. for $C_{14}H_{15}NO_2$: 229.1103; found: 229.1099.

Spectral data for 2-phenyl-6*H*-1,3-oxazin-6-one (3b).

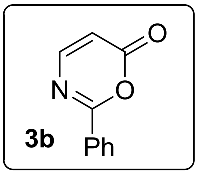

White solid; ^{1}H NMR (600 MHz, $CDCl_3$): δ 8.20 ~ 8.18 (m, 2 H), 7.80 (d, *J* = 6.6 Hz, 1 H), 7.58 ~ 7.55 (m, 1 H), 7.48 ~ 7.45 (m, 2 H), 6.19 (d, *J* = 6.6, 1 H); ^{13}C NMR (150 MHz, $CDCl_3$): δ 164.7, 158.3, 154.6, 133.4, 129.6, 128.8, 128.5, 109.5; HRMS calcd. for $C_{10}H_7NO_2$: 173.0477; found: 173.0480.

Spectral data for 4-isopropyl-2-phenyl-6*H*-1,3-oxazin-6-one (3c).

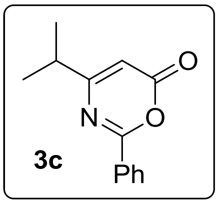

White solid; ^{1}H NMR (600 MHz, $CDCl_3$): δ 8.20 ~ 8.18 (m, 2 H), 7.53 ~ 7.50 (m, 1 H), 7.44 ~ 7.42 (m, 2 H), 5.97 (d, J = 0.6 Hz, 1 H), 2.74 ~ 2.69 (m, 1 H), 1.23 (d, J = 7.2 Hz, 6 H); ^{13}C NMR (150 MHz, $CDCl_3$): δ 174.3, 162.9, 159.6, 133.0, 129.8, 128.6, 128.4, 103.0, 35.1, 20.2; HRMS calcd. for $C_{13}H_{13}NO_2$: 215.0946; found: 215.0944.

Spectral data for 4-cyclopropyl-2-phenyl-6*H*-1,3-oxazin-6-one (3d).

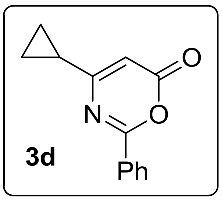

White solid; ^{1}H NMR (600 MHz, $CDCl_3$): δ 8.14 ~ 8.12 (m, 2 H), 7.54 ~ 7.51 (m, 1 H), 7.44 ~ 7.41 (m, 2 H), 6.04 (s, 1 H), 1.81 ~ 1.77 (m, 1 H), 1.24 ~ 1.21 (m, 2 H), 1.04 ~ 1.01 (m, 2 H); ^{13}C NMR (150 MHz, $CDCl_3$): δ 170.8, 163.4, 158.8, 133.1, 129.8, 128.6, 128.4, 102.7, 16.6, 9.3; HRMS calcd. for $C_{13}H_{11}NO_2$: 213.0790; found: 213.0785.

Spectral data for 4-cyclohexyl-2-phenyl-6*H*-1,3-oxazin-6-one (3e).

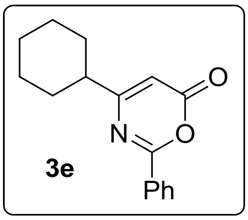

White solid; ^{1}H NMR (600 MHz, $CDCl_3$): δ 8.21 ~ 8.19 (m, 2 H), 7.54 ~ 7.51 (m, 1 H), 7.46 ~ 7.43 (m, 2 H), 5.95 (d, J = 0.6 Hz, 1 H), 2.40 ~ 2.36 (m, 1 H), 1.95 ~ 1.93 (m, 2 H), 1.84 ~ 1.81 (m, 2 H), 1.74 ~ 1.71 (m, 1 H), 1.44 ~ 1.31 (m, 4 H), 1.26 ~ 1.19 (m, 1 H); ^{13}C NMR (150 MHz, $CDCl_3$): δ 173.4, 162.8, 159.8, 133.0, 129.9, 128.6, 128.5, 103.3, 44.9, 30.5, 25.8, 25.7; HRMS calcd. for $C_{16}H_{17}NO_2$: 255.1259; found: 255.1251.

Spectral data for 2-phenyl-4-(prop-1-en-2-yl)-6*H*-1,3-oxazin-6-one (3f).

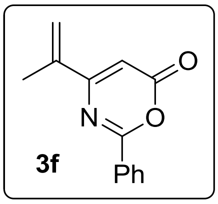

White solid; ^{1}H NMR (600 MHz, $CDCl_3$): δ 8.25 ~ 8.23 (m, 2 H), 7.57 ~ 7.54 (m, 1 H), 7.48 ~ 7.45 (m, 2 H), 6.52 (d, *J* = 0.6 Hz, 1 H), 6.13 (s, 1 H), 5.57 (m, 1 H), 2.06 ~ 2.05 (m, 3 H); ^{13}C NMR (150 MHz, $CDCl_3$): δ 161.5, 160.2, 138.7, 133.1, 130.0, 128.7, 128.5, 123.5, 102.2, 18.8 (one carbon merged with others); HRMS calcd. for $C_{13}H_{11}NO_2$: 213.0790; found: 213.0787.

Spectral data for 2,4-diphenyl-6*H*-1,3-oxazin-6-one (3g).

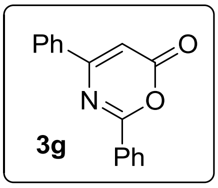

White solid; ^{1}H NMR (600 MHz, $CDCl_3$): δ 8.35 ~ 8.34 (m, 2 H), 8.09 ~ 8.08 (m, 2 H), 7.61 ~ 7.49 (m, 6 H), 6.59 (s, 1 H); ^{13}C NMR (150 MHz, $CDCl_3$): δ 163.2, 161.9, 159.9, 134.5, 133.4, 131.9, 130.1, 129.0, 128.8, 128.7, 127.4, 101.7; HRMS calcd. for $C_{16}H_{11}NO_2$: 249.0790; found: 249.0785.

Spectral data for 4-(4-methoxyphenyl)-2-phenyl-6H-1,3-oxazin-6-one (3h).

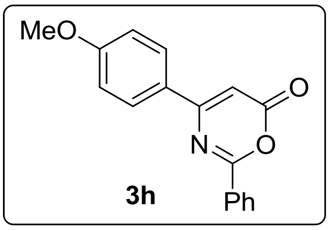

White solid; ^{1}H NMR (600 MHz, $CDCl_3$): δ 8.32 (d, *J* = 7.8 Hz, 2 H), 8.05 (d, *J* = 9.0 Hz, 2 H), 7.59 ~ 7.57 (m, 1 H), 7.51 ~ 7.58 (m, 2 H), 6.99 (d, *J* = 9.0 Hz, 2 H), 6.46 (s, 1 H), 3.87 (s, 3 H); ^{13}C NMR (150 MHz, $CDCl_3$): δ 162.8, 162.8, 161.4, 160.1, 133.2, 130.1, 129.2, 128.7, 128.7, 126.8, 114.3, 99.5, 55.5; HRMS calcd. for $C_{17}H_{13}NO_3$: 279.0895; found: 279.0894.

Spectral data for 4-(4-fluorophenyl)-2-phenyl-6*H*-1,3-oxazin-6-one (3i).

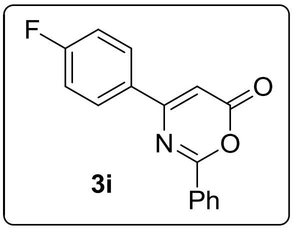

White solid; ^{1}H NMR (600 MHz, $CDCl_3$): δ 8.31 (dd, *J* = 8.4, 1.2 Hz, 2 H), 8.10 ~ 8.07 (m, 2 H), 7.61 ~ 7.58 (m, 1 H), 7.52 ~ 7.49 (m, 2 H), 7.19 ~ 7.16 (m, 2 H), 6.51 (s, 1 H); ^{13}C NMR (150 MHz, $CDCl_3$): δ 165.1 (d, *J* = 252.0 Hz), 163.3, 160.7, 159.7, 133.5, 130.6, 129.9, 129.6 (d, *J* = 9.0 Hz), 128.8, 127.7, 116.1 (d, *J* = 22.5 Hz), 101.2; HRMS calcd. for $C_{16}H_{10}FNO_2$: 267.0696; found: 267.0702.

Spectral data for 4-(4-chlorophenyl)-2-phenyl-6*H*-1,3-oxazin-6-one (3j).

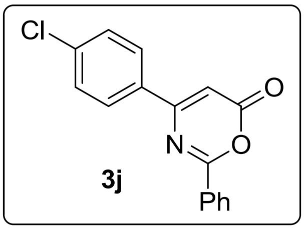

White solid; ^{1}H NMR (600 MHz, $CDCl_3$): δ 8.32 ~ 8.31 (m, 2 H), 8.02 (dd, *J* = 6.6, 1.8 Hz, 2 H), 7.62 ~ 7.59 (m, 1 H), 7.52 ~ 7.50 (m, 2 H), 7.47 ~ 7.46 (m, 2 H), 6.55 (s, 1 H); ^{13}C NMR (150 MHz, $CDCl_3$): δ 163.4, 160.7, 159.6, 138.2, 133.5, 132.9, 129.8, 129.2, 128.8, 128.7, 128.6, 101.7; HRMS calcd. for $C_{16}H_{10}ClNO_2$: 283.0400; found: 283.0404.

Spectral data for 2-phenyl-4-(thiophen-2-yl)-6*H*-1,3-oxazin-6-one (3k).

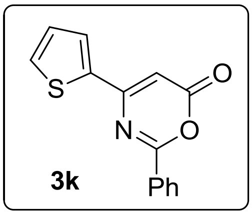

White solid; ^{1}H NMR (600 MHz, $CDCl_3$): δ 8.30 ~ 8.29 (m, 2 H), 7.80 (dd, *J* = 3.6, 0.9 Hz, 1 H), 7.61 ~ 7.58 (m, 2 H), 7.52 ~ 7.49 (m, 2 H), 7.18 (dd, *J* = 4.8, 3.6 Hz, 1 H), 6.39 (s, 1 H); ^{13}C NMR (150 MHz, $CDCl_3$): δ 163.5, 159.5, 156.8, 139.9, 133.5, 131.9, 129.7, 129.0, 128.9, 128.8, 128.7, 98.5; HRMS calcd. for $C_{14}H_9NO_2S$: 255.0354; found: 255.0355.

Spectral data for 2-phenyl-4-(thiophen-3-yl)-6*H*-1,3-oxazin-6-one (3l).

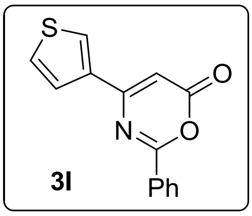

White solid; ^{1}H NMR (600 MHz, $CDCl_3$): δ 8.29 ~ 8.25 (m, 3 H), 7.59 ~ 7.56 (m, 1 H), 7.52 ~ 7.47 (m, 3 H), 7.41 (dd, *J* = 4.8, 3.0 Hz, 1 H), 6.36 (s, 1 H); ^{13}C NMR (150 MHz, $CDCl_3$): δ 163.2, 159.9, 157.2, 137.8, 133.3, 129.8, 129.6, 128.7, 128.6, 127.4, 125.3, 100.6; HRMS calcd. for $C_{14}H_9NO_2S$: 255.0354; found: 255.0354.

Spectral data for 4-butyl-2-(4-methoxyphenyl)-6*H*-1,3-oxazin-6-one (3m).

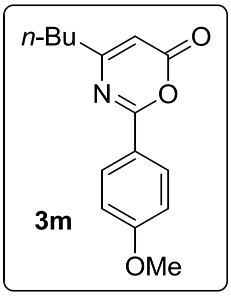

White solid; ^{1}H NMR (600 MHz, $CDCl_3$): δ 8.13 (d, *J* = 9.0 Hz, 2 H), 6.91 (d, *J* = 9.0 Hz, 2 H), 5.89 (s, 1 H), 3.83 (s, 3 H), 2.46 (t, *J* = 7.8 Hz, 2 H), 1.64 ~ 1.61 (m, 2 H), 1.38 ~ 1.34 (m, 2 H), 0.91 (t, *J* = 7.5 Hz, 3 H); ^{13}C NMR (150 MHz, $CDCl_3$): δ 170.1, 163.6, 162.9, 159.6, 130.5, 122.1, 114.0, 103.9, 55.4, 36.8, 29.0, 22.1, 13.7; HRMS calcd. for $C_{15}H_{17}NO_3$: 259.1208; found: 259.1210.

Spectral data for 4-butyl-2-(*p*-tolyl)-6H-1,3-oxazin-6-one (3n).

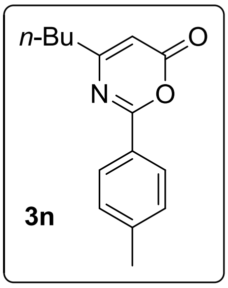

Colorless oil; ^{1}H NMR (600 MHz, $CDCl_3$): δ 8.10 (d, *J* = 8.4 Hz, 2 H), 7.26 (d, *J* = 8.4 Hz, 2

H), 5.96 (s, 1 H), 2.51 (t, *J* = 7.8 Hz, 2 H), 2.41 (s, 3 H), 1.71 ~ 1.66 (m, 2 H), 1.44 ~ 1.38 (m, 2 H), 0.96 (t, *J* = 7.5 Hz, 3 H); ^{13}C NMR (150 MHz, $CDCl_3$): δ 169.9, 163.0, 159.3, 143.9, 129.3, 128.4, 127.0, 104.5, 36.7, 29.0, 22.1, 21.5, 13.7; HRMS calcd. for $C_{15}H_{17}NO_2$: 243.1259; found: 243.1260.

Spectral data for methyl 4-(4-butyl-6-oxo-6*H*-1,3-oxazin-2-yl)benzoate (3o).

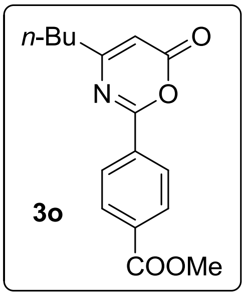

White solid; ^{1}H NMR (600 MHz, $CDCl_3$): δ 8.29 (d, *J* = 8.4 Hz, 2 H), 8.12 (d, *J* = 8.4 Hz, 2 H), 6.04 (s, 1 H), 3.94 (s, 3 H), 2.55 ~ 2.52 (m, 2 H), 1.71 ~ 1.66 (m, 2 H), 1.43 ~ 1.37 (m, 2 H), 0.95 (t, *J* = 7.5 Hz, 3 H); ^{13}C NMR (150 MHz, $CDCl_3$): δ 169.7, 166.1, 162.0, 158.9, 134.0, 133.7, 129.8, 128.5, 105.8, 52.5, 36.8, 29.1, 22.2, 13.8; HRMS calcd. for $C_{16}H_{17}NO_4$: 287.1158; found: 287.1158.

Spectral data for methyl 4-butyl-2-(4-chlorophenyl)-6*H*-1,3-oxazin-6-one (3p).

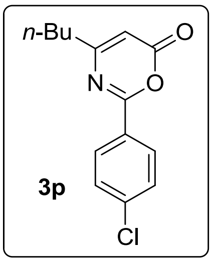

White solid; ^{1}H NMR (600 MHz, $CDCl_3$): δ 8.17 ~ 8.16 (m, 2 H), 7.46 ~ 7.45 (m, 2 H), 6.02 (s, 1 H), 2.54 (t, *J* = 7.5 Hz, 2 H), 1.70 ~ 1.68 (m, 2 H), 1.44 ~ 1.40 (m, 2 H), 0.97 (t, *J* = 7.5 Hz, 3 H); ^{13}C NMR (150 MHz, $CDCl_3$): δ 169.7, 162.0, 158.9, 139.6, 129.8, 129.0, 128.2, 105.1, 36.7, 29.0, 21.1, 13.7; HRMS calcd. for $C_{14}H_{14}ClNO_2$: 263.0713; found: 263.0713.

Spectral data for 4-butyl-2-cyclohexyl-6*H*-1,3-oxazin-6-one (3q).

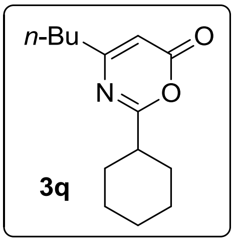

Colorless oil; ^{1}H NMR (600 MHz, $CDCl_3$): δ 5.84 (s, 1 H), 2.49 ~ 2.45 (m, 1 H), 2.36 (t, *J* = 7.8 Hz, 2 H), 1.93 ~ 1.90 (m, 2 H), 1.76 ~ 1.73 (m, 2 H), 1.64 ~ 1.44 (m, 5 H), 1.31 ~ 1.16 (m, 5 H), 0.86 (t, *J* = 7.5 Hz, 3 H); ^{13}C NMR (150 MHz, $CDCl_3$): δ 172.2, 169.3, 159.8, 104.7, 43.4, 36.5, 29.5, 28.8, 25.4, 25.3, 22.0, 13.6; HRMS calcd. for $C_{14}H_{21}NO_2$: 235.1572; found: 235.1572.

Spectral data for (*E*)-4-butyl-2-styryl-6*H*-1,3-oxazin-6-one (3r).

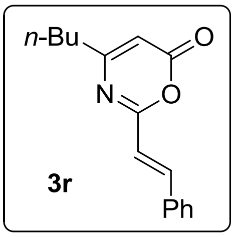

Colorless oil; ^{1}H NMR (600 MHz, $CDCl_3$): δ 7.80 (d, *J* = 16.2 Hz, 1 H), 7.53 ~ 7.51 (m, 2H), 7.37 ~ 7.36 (m, 3 H), 6.67 (d, *J* = 16.2 Hz, 1 H), 5.91 (s, 1 H), 2.44 (t, *J* = 7.5 Hz, 2 H), 1.64 ~ 1.58 (m, 2 H), 1.39 ~ 1.33 (m, 2 H), 0.91 (t, *J* = 7.5 Hz, 3 H); ^{13}C NMR (150 MHz, $CDCl_3$): δ 169.8, 163.1, 159.1, 143.4, 134.3, 130.6, 128.9, 128.1, 118.5, 104.8, 36.7, 29.1, 22.1, 13.7; HRMS calcd. for $C_{16}H_{17}NO_2$: 255.1259; found: 255.1259.

Spectral data for 4-butyl-2-(thiophen-3-yl)-6*H*-1,3-oxazin-6-one (3s).

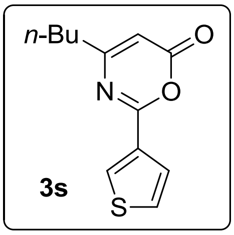

Colorless oil; ^{1}H NMR (600 MHz, $CDCl_3$): δ 8.17 (dd, *J* = 3.0, 1.2 Hz, 1 H), 7.61 (dd, *J* = 4.8, 1.2 Hz, 1 H), 7.29 (dd, *J* = 4.8, 3.0 Hz, 1 H), 5.88 (s, 1 H), 2.41 (t, *J* = 7.5 Hz, 2 H), 1.61

~ 1.55 (m, 2 H), 1.35 ~ 1.28 (m, 2 H), 0.87 (t, *J* = 7.5 Hz, 3 H); ^{13}C NMR (150 MHz, $CDCl_3$): δ 170.0, 159.3, 158.9, 132.9, 131.5, 126.8, 126.6, 104.4, 36.5, 28.8, 22.0, 13.6; HRMS calcd. for $C_{12}H_{13}NO_2S$: 235.0667; found: 235.0668.

Spectral data for diethyl 6-butyl-2-phenylpyridine-3,4-dicarboxylate (5a).

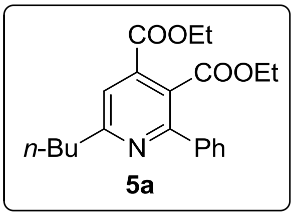

Colorless liquid; ^{1}H NMR (600 MHz, $CDCl_3$): δ 7.57 ~ 7.54 (m, 3 H), 7.40 ~ 7.37 (m, 3 H), 4.36 (q, *J* = 7.2 Hz, 2 H), 4.14 (q, *J* = 7.2 Hz, 2 H), 2.89 (t, *J* = 7.2 Hz, 2 H), 1.76 ~ 1.71 (m, 2 H), 1.40 ~ 1.34 (m, 5 H), 1.05 (t, *J* = 7.2 Hz, 3 H), 0.92 (t, *J* = 7.2 Hz, 3 H); ^{13}C NMR (150 MHz, $CDCl_3$): δ 168.0, 165.0, 164.0, 156.9, 139.2, 137.2, 128.7, 128.5, 128.2, 125.8, 120.1, 62.1, 61.6, 38.0, 31.7, 22.4, 14.0, 13.8, 13.5; HRMS calcd. for $C_{21}H_{25}NO_4$: 355.1784; found: 355.1785.

Spectral data for diethyl 2-phenylpyridine-3,4-dicarboxylate (5b).

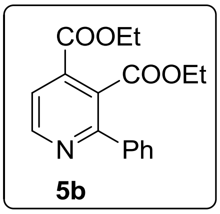

Colorless oil; ^{1}H NMR (600 MHz, $CDCl_3$): δ 8.83 (d, *J* = 4.8 Hz, 1 H), 7.74 (d, *J* = 4.8 Hz, 1 H), 7.58 ~ 7.56 (m, 2 H), 7.41 ~ 7.40 (m, 3 H), 4.38 (q, *J* = 7.2 Hz, 2 H), 4.18 (q, *J* = 7.2 Hz, 2 H), 1.37 (t, *J* = 7.2 Hz, 3 H), 1.07 (t, *J* = 7.2 Hz, 3 H); ^{13}C NMR (150 MHz, $CDCl_3$): δ 167.6, 164.6, 157.6, 150.5, 138.8, 136.9, 129.0, 128.6, 128.5, 128.3, 121.2, 62.3, 61.8, 14.0, 13.6; HRMS calcd. for $C_{17}H_{17}NO_4$: 299.1158; found: 299.1160.

Spectral data for diethyl 6-isopropyl-2-phenylpyridine-3,4-dicarboxylate (5c).

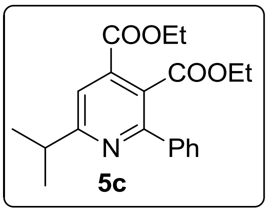

Colorless oil; ^{1}H NMR (600 MHz, $CDCl_3$): δ 7.60 ~ 7.58 (m, 3 H), 7.39 ~ 7.35 (m, 3 H), 4.34 (q, J = 7.2 Hz, 2 H), 4.15 (q, J = 6.6 Hz, 2 H), 3.19 ~ 3.14 (m, 1 H), 1.33 ~ 1.30 (m, 9 H), 1.04 (t, J = 7.2 Hz, 3 H); ^{13}C NMR (150 MHz, $CDCl_3$): δ 168.5, 167.9, 164.9, 156.4, 139.1, 137.4, 128.5, 128.4, 127.9, 125.7, 117.9, 61.9, 61.4, 36.2, 22.1, 13.8, 13.4; HRMS calcd. for $C_{20}H_{23}NO_4$: 341.1627; found: 341.1629.

Spectral data for diethyl 6-cyclopropyl-2-phenylpyridine-3,4-dicarboxylate (5d).

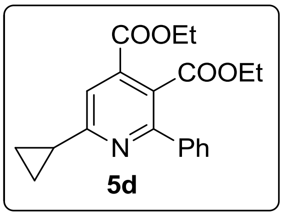

Colorless oil; ^{1}H NMR (600 MHz, $CDCl_3$): δ 7.56 ~ 7.55 (m, 2 H), 7.53 (s, 1 H), 7.39 ~ 7.36 (m, 3 H), 4.36 (q, J = 7.2 Hz, 2 H), 4.15 (q, J = 7.2 Hz, 2 H), 2.16 ~ 2.11 (m, 1 H), 1.35 (t, J = 7.2 Hz, 3 H), 1.13 ~ 1.10 (m, 2 H), 1.07 ~ 1.00 (m, 5 H); ^{13}C NMR (150 MHz, $CDCl_3$): δ 168.1, 165.1, 164.4, 156.7, 139.3, 137.0, 128.6, 128.5, 128.0, 125.0, 118.5, 62.0, 61.5, 17.4, 13.9, 13.5, 10.6; HRMS calcd. for $C_{20}H_{21}NO_4$: 339.1471; found: 339.1469.

Spectral data for diethyl 6-cyclohexyl-2-phenylpyridine-3,4-dicarboxylate (5e).

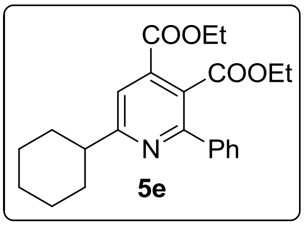

Colorless oil; ^{1}H NMR (600 MHz, $CDCl_3$): δ 7.59 ~ 7.57 (m, 3 H), 7.39 ~ 7.35 (m, 3 H), 4.35 (q, J = 7.2 Hz, 2 H), 4.15 (q, J = 7.2 Hz, 2 H), 2.86 ~ 2.80 (m, 1 H), 1.98 ~ 1.96 (m, 2 H), 1.84 ~ 1.81 (m, 2 H), 1.72 ~ 1.70 (m, 1 H), 1.59 ~ 1.52 (m, 2 H), 1.41 ~ 1.23 (m, 6 H), 1.05 (t, J = 7.2 Hz, 3 H); ^{13}C NMR (150 MHz, $CDCl_3$): δ 168.0, 167.8, 165.0, 156.4, 139.2,

137.3, 128.6, 128.4, 128.0, 125.7, 118.3, 62.0, 61.4, 46.3, 32.5, 26.2, 25.7, 13.9, 13.4,; HRMS calcd. for $C_{23}H_{27}NO_4$: 381.1940; found: 381.1939.

Spectral data for diethyl 2,6-diphenylpyridine-3,4-dicarboxylate (5f).

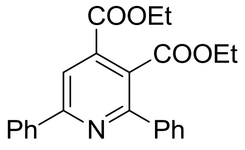

5f

Colorless oil; ^{1}H NMR (600 MHz, $CDCl_3$): δ 8.17 (s, 1 H), 8.13 ~ 8.11 (m, 2 H), 7.70 ~ 7.69 (m, 2 H), 7.49 ~ 7.42 (m, 6 H), 4.42 (q, *J* = 7.2 Hz, 2 H), 4.21 (q, *J* = 7.2 Hz, 2 H), 1.40 (t, *J* = 7.2 Hz, 3 H), 1.10 (t, *J* = 7.2 Hz, 3 H); ^{13}C NMR (150 MHz, $CDCl_3$): δ 168.0, 165.0, 158.0, 157.2, 139.2, 138.0, 137.7, 129.9, 128.9, 128.8, 128.7, 128.2, 127.2, 126.6, 117.7, 62.3, 61.8, 14.0, 13.6; HRMS calcd. for $C_{23}H_{21}NO_4$: 375.1471; found: 375.1472.

Spectral data for diethyl 2-phenyl-6-(thiophen-3-yl)pyridine-3,4-dicarboxylate (5g).

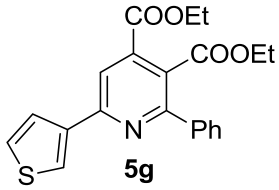

5g

Colorless oil; ^{1}H NMR (600 MHz, $CDCl_3$): δ 8.05 (dd, *J* = 3.0, 1.2 Hz, 1 H), 8.02 (s, 1 H), 7.74 (dd, *J* = 4.8, 1.2 Hz, 1 H), 7.69 ~ 7.67 (m, 2 H), 7.44 ~ 7.41 (m, 3 H), 7.36 (dd, *J* = 4.8, 3.0 Hz, 1 H), 4.40 (q, *J* = 7.2 Hz, 2 H), 4.21 (q, *J* = 7.2 Hz, 2 H), 1.38 (t, *J* = 7.2 Hz, 3 H), 1.10 (t, *J* = 7.2 Hz, 3 H); ^{13}C NMR (150 MHz, $CDCl_3$): δ 167.8, 164.8, 157.1, 153.9, 140.7, 139.0, 137.9, 128.8, 128.5, 128.0, 126.4, 126.1, 125.9, 125.3, 117.3, 62.1, 61.6, 13.9, 13.5; HRMS calcd. for $C_{21}H_{19}NO_4S$: 381.1035; found: 381.1036.

Spectral data for diethyl diethyl 6-butyl-2-cyclohexylpyridine-3,4-dicarboxylate (5h).

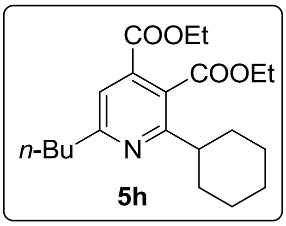

Colorless oil; ^{1}H NMR (600 MHz, $CDCl_3$): δ 7.37 (s, 1 H), 4.38 (q, *J* = 7.2 Hz, 2 H), 4.30 (q, *J* = 7.2 Hz, 2 H), 2.77 (t, *J* = 7.8 Hz, 2 H), 2.70 ~ 2.65 (m, 1 H), 1.78 ~ 1.63 (m, 10 H), 1.35 ~ 1.26 (m, 10 H), 0.89 (t, *J* = 7.2 Hz, 3 H); ^{13}C NMR (150 MHz, $CDCl_3$): δ 168.6, 165.1, 163.5, 162.7, 135.7, 125.0, 118.8, 61.8, 61.4, 43.7, 37.8, 32.1, 31.4, 26.3, 25.7, 22.2, 14.0, 13.9, 13.8; HRMS calcd. for $C_{21}H_{31}NO_4$: 361.2253; found: 361.2253.

Spectral data for diethyl 6-butyl-2-(thiophen-3-yl)pyridine-3,4-dicarboxylate (5i).

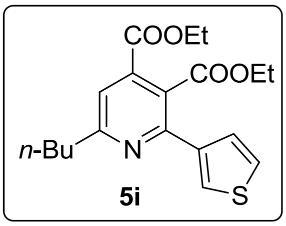

Colorless oil; ^{1}H NMR (600 MHz, $CDCl_3$): δ 7.64 (dd, *J* = 3.0, 1.2 Hz, 1 H), 7.54 (s, 1 H), 7.41 (dd, *J* = 5.1, 1.2 Hz, 1 H), 7.30 (dd, *J* = 5.1, 3.0 Hz, 1 H), 4.33 (q, *J* = 7.2 Hz, 2 H), 4.26 (q, *J* = 7.2 Hz, 2 H), 2.84 (t, *J* = 7.8 Hz, 2 H), 1.74 ~ 1.68 (m, 2 H), 1.38 ~ 1.32 (m, 5 H), 1.17 (t, *J* = 7.2 Hz, 3 H), 0.90 (t, *J* = 7.2 Hz, 3 H); ^{13}C NMR (150 MHz, $CDCl_3$): δ 168.3, 164.6, 163.8, 151.4, 139.9, 136.5, 128.0, 125.4, 125.3, 125.1, 119.9, 62.0, 61.7, 37.8, 31.5, 22.2; HRMS calcd. for $C_{19}H_{23}NO_4S$: 361.1348; found: 361.1347.

Spectral data for (*E*)-diethyl 6-butyl-2-styrylpyridine-3,4-dicarboxylate (5j).

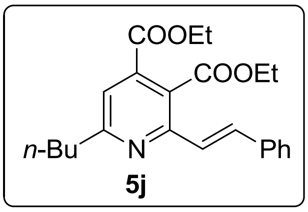

Colorless oil; ^{1}H NMR (600 MHz, $CDCl_3$): δ 7.92 (d, *J* = 15.6 Hz, 1 H), 7.55 (d, *J* = 7.8 Hz, 2 H), 7.45 (s, 1 H), 7.34 (t, *J* = 7.5 Hz, 2 H), 7.28 ~ 7.26 (m, 1 H), 7.22 (d, *J* = 15.6 Hz, 1 H), 4.45 (q, *J* = 7.2 Hz, 2 H), 4.35 (q, *J* = 7.2 Hz, 2 H), 2.87 (t, *J* = 7.8 Hz, 2 H), 1.79 ~ 1.74 (m,

2 H), 1.43 ~ 1.34 (m, 8 H), 0.95 (t, *J* = 7.2 Hz, 3 H); ^{13}C NMR (150 MHz, $CDCl_3$): δ 168.0, 165.0, 163.9, 152.1, 136.9, 136.4, 135.8, 128.5, 127.3, 124.7, 123.2, 120.0, 61.9, 61.8, 38.0, 31.4, 22.3, 14.0, 13.9, 13.8 (one carbon merged with others); HRMS calcd. for $C_{23}H_{27}NO_4$: 381.1940; found: 381.1940.

Spectral data for methyl 6-butyl-2-phenylnicotinate (5k).

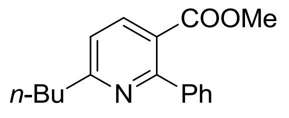

5k

Colorless oil; ^{1}H NMR (600 MHz, $CDCl_3$): δ 7.98 (d, *J* = 7.8 Hz, 1 H), 7.52 (d, *J* = 7.2 Hz, 2 H), 7.41 ~ 7.35 (m, 3 H), 7.13 (d, *J* = 7.8 Hz, 1 H), 3.63 (s, 3 H), 2.86 (t, *J* = 7.8 Hz, 2 H), 1.76 ~ 1.71 (m, 2 H), 1.40 ~ 1.36 (m, 2 H), 0.92 (t, *J* = 7.5 Hz, 3 H); ^{13}C NMR (150 MHz, $CDCl_3$): δ 168.4, 164.6, 158.2, 140.2, 137.9, 128.3, 128.2, 127.8, 123.8, 120.2, 51.8, 38.0, 31.5, 22.2, 13.7; HRMS calcd. for $C_{17}H_{19}NO_2$: 269.1416; found: 269.1415.

Spectral data for (6-butyl-2-phenylpyridin-3-yl)(phenyl)methanone (5l).

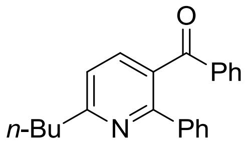

5l

Colorless oil; ^{1}H NMR (600 MHz, $CDCl_3$): δ 7.74 (d, *J* = 7.8 Hz, 1 H), 7.66 ~ 7.64 (m, 2 H), 7.51 ~ 7.49 (m, 2 H), 7.42 ~ 7.39 (m, 1 H), 7.28 ~ 7.26 (m, 2 H), 7.23 ~ 7.16 (m, 4 H), 2.94 (t, *J* = 8.1 Hz, 2 H), 1.85 ~ 1.80 (m, 2 H), 1.49 ~ 1.43 (m, 2 H), 0.98 (t, *J* = 7.3 Hz, 3 H); ^{13}C NMR (150 MHz, $CDCl_3$): δ 197.5, 164.2, 156.9, 139.5, 137.3, 136.9, 133.0, 131.6, 129.7, 129.2, 128.5, 128.2, 120.3, 38.2, 31.7, 22.5, 13.9 (one carbon merged with others); HRMS calcd. for $C_{22}H_{21}NO$: 315.1623; found: 315.1621.

Spectral data for ethyl 4,6-dibutyl-2-phenylnicotinate (5m) (Major isomer) + ethyl 3,6-dibutyl-2-phenylisonicotinate (5m') (Minor isomer).

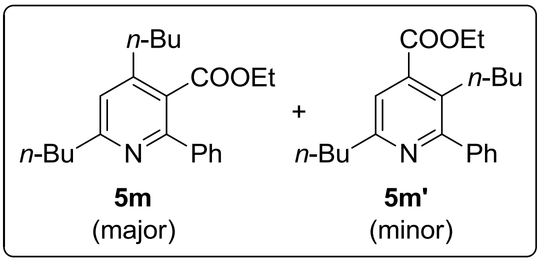

Spectral data for ethyl 4,6-dibutyl-2-phenylnicotinate (**5m**) (Major isomer): Colorless oil; ^{1}H NMR (600 MHz, $CDCl_3$): δ 7.55 ~ 7.53 (m, 2 H), 7.40 ~ 7.31 (m, 3 H), 6.97 (s, 1 H), 4.05 (q, *J* = 7.2 Hz, 2 H), 2.80 (t, *J* = 7.8 Hz, 2 H), 2.65 (t, *J* = 8.1 Hz, 2 H), 1.73 ~ 1.67 (m, 2 H), 1.61 ~ 1.56 (m, 2 H), 1.40 ~ 1.34 (m, 4 H), 0.96 ~ 0.89 (m, 9 H); ^{13}C NMR (150 MHz, $CDCl_3$): δ 169.1, 162.7, 156.3, 150.0, 140.5, 128.7, 128.3, 128.1, 126.3, 121.3, 61.1, 38.0, 32.8, 32.6, 31.8, 22.5, 22.4, 13.8, 13.7, 13.5; HRMS calcd. for $C_{22}H_{29}NO_2$: 339.2198; found: 339.2201. Spectral data for ethyl 3,6-dibutyl-2-phenylisonicotinate (**5m'**) (Minor isomer): Colorless oil; ^{1}H NMR (600 MHz, $CDCl_3$): δ 4.38 (q, *J* = 7.2 Hz, 2 H), 1.15 ~ 1.12 (m, 2 H), 0.70 (t, *J* = 7.8 Hz, 3 H); ^{13}C NMR (150 MHz, $CDCl_3$): δ 167.6, 160.4, 159.5, 140.9, 139.5, 128.2, 128.0, 127.7, 120.6, 37.7, 33.4, 32.0, 28.7, 22.6, 14.1; HRMS calcd. for $C_{22}H_{29}NO_2$: 339.2198; found: 339.2201.

Spectral data for (4,6-dibutyl-2-phenylpyridin-3-yl)(phenyl)methanone (5n).

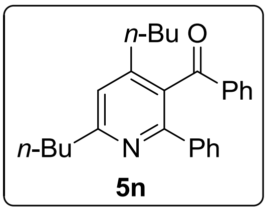

Colorless oil; ^{1}H NMR (600 MHz, $CDCl_3$): δ 7.56 (dd, *J* = 8.1, 1.5 Hz, 1 H), 7.44 ~ 7.42 (m, 2 H), 7.39 ~ 7.37 (m, 1 H), 7.24 (t, *J* = 7.8 Hz, 2 H), 7.17 ~ 7.11 (m, 3 H), 7.09 (s, 1 H), 2.89 (t, *J* = 8.1 Hz, 2 H), 2.52 (t, *J* = 7.5 Hz, 2 H), 1.83 ~ 1.78 (m, 2 H), 1.52 ~ 1.42 (m, 4 H), 1.29 ~ 1.23 (m, 2 H), 0.98 (t, *J* = 7.5 Hz, 3 H), 0.81 (t, *J* = 7.5 Hz, 3 H); ^{13}C NMR (150 MHz, $CDCl_3$): δ 198.5, 162.7, 155.7, 150.4, 140.0, 137.7, 133.1, 131.5, 129.2, 129.1, 128.2, 128.1, 128.0, 121.5, 38.2, 32.6, 32.5, 32.0, 22.6, 22.5, 14.0, 13.7; HRMS calcd. for $C_{26}H_{29}NO$:

371.2249; found: 371.2247.

Spectral data for (6-butyl-2,4-diphenylpyridin-3-yl)(phenyl)methanone (5o).

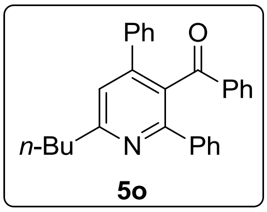

Colorless oil; ^{1}H NMR (600 MHz, CDCl$_3$): δ 7.56 ~ 7.54 (m, 2 H), 7.49 ~ 7.47 (m, 2 H), 7.35 ~ 7.32 (m, 1 H), 7.27 ~ 7.18 (m, 11 H), 2.98 (t, *J* = 7.8 Hz, 2 H), 1.88 ~ 1.83 (m, 2 H), 1.52 ~ 1.46 (m, 2 H), 1.00 (t, *J* = 7.5 Hz, 3 H); ^{13}C NMR (150 MHz, CDCl$_3$): δ 197.8, 163.0, 156.6, 149.3, 139.8, 138.2, 137.8, 132.9, 131.0, 129.3, 129.2, 128.6, 128.3, 128.2, 128.1, 128.1, 121.9, 38.2, 32.0, 22.6, 14.0 (one carbon merged with others); HRMS calcd. for $C_{28}H_{25}NO$: 391.1936; found: 391.1937.

Spectral data for 2,6-diphenyl-4*H*-1,3-dioxin-4-one (6a).

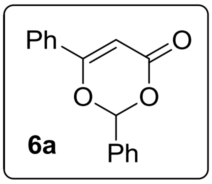

White solid; ^{1}H NMR (600 MHz, CDCl$_3$): δ 7.75 ~ 7.73 (m, 2 H), 7.67 ~ 7.65 (m, 2 H), 7.53 ~ 7.43 (m, 6 H), 6.55 (s, 1 H), 6.06 (s, 1 H); ^{13}C NMR (150 MHz, CDCl$_3$): δ 168.1, 162.9, 133.7, 132.5, 130.4, 130.1, 128.9, 128.6, 126.6, 126.5, 100.3, 93.2; HRMS calcd. for $C_{16}H_{12}O_3$: 252.0786; found: 252.0786.

Spectral data for 2-methyl-2,6-diphenyl-4*H*-1,3-dioxin-4-one (6b).

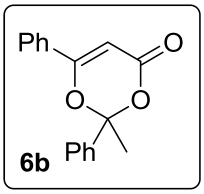

White solid; ^{1}H NMR (600 MHz, CDCl$_3$): δ 7.75 (d, *J* = 7.2 Hz, 2 H), 7.51 ~ 7.48 (m, 3 H), 7.43 (t, *J* = 7.5 Hz, 2 H), 7.30 ~ 7.29 (m, 3 H), 5.80 (s, 1 H), 1.99 (s, 3 H); ^{13}C NMR (150

MHz, $CDCl_3$): δ 165.0, 162.0, 139.9, 132.1, 130.5, 129.0, 128.8, 128.5, 126.1, 124.7, 106.7, 93.3, 29.4; HRMS calcd. for $C_{17}H_{14}O_3$: 266.0943; found: 266.0944.

Spectral data for 2,2-dimethyl-6-phenyl-4*H*-1,3-dioxin-4-one (6c).

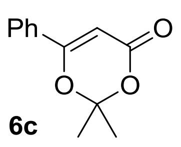

6c

White solid; ^{1}H NMR (600 MHz, $CDCl_3$): δ 7.61 ~ 7.60 (m, 2 H), 7.44 ~ 7.41 (m, 1 H), 7.37 ~ 7.35 (m, 2 H), 5.81 (s, 1 H), 1.72 (s, 6 H); ^{13}C NMR (150 MHz, $CDCl_3$): δ 164.7, 161.5, 131.8, 130.7, 128.5, 126.0, 106.3, 90.9, 24.7; HRMS calcd. for $C_{12}H_{12}O_3$: 204.0786; found: 204.0787.

Spectral data for 6-butyl-2-phenyl-4*H*-1,3-dioxin-4-one (6d).

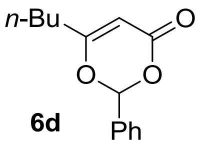

6d

Colorless liquid; ^{1}H NMR (600 MHz, $CDCl_3$): δ 7.53 ~ 7.51 (m, 2 H), 7.40 ~ 7.37 (m, 3 H), 6.30 (s, 1 H), 5.36 (s, 1 H), 2.30 ~ 2.27 (m, 2 H), 1.54 ~ 1.49 (m, 2 H), 1.35 ~ 1.28 (m, 2 H), 0.87 (t, J = 7.5 Hz, 3 H); ^{13}C NMR (150 MHz, $CDCl_3$): δ 175.3, 162.1, 133.5, 130.0, 128.3, 126.3, 99.6, 95.3, 32.6, 27.6, 21.8, 13.4; HRMS calcd. for $C_{14}H_{16}O_3$: 232.1099; found: 232.1091.

Spectral data for 6-cyclohexyl-2-phenyl-4*H*-1,3-dioxin-4-one (6e).

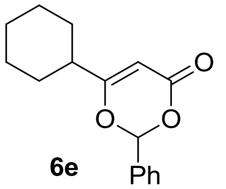

6e

White solid; ^{1}H NMR (600 MHz, $CDCl_3$): δ 7.56 ~ 7.55 (m, 2 H), 7.44 ~ 7.43 (m, 3 H), 6.30 (s, 1 H), 5.38 (d, J = 0.6 Hz, 1 H), 2.26 ~ 2.22 (m, 1 H), 1.93 ~ 1.88 (m, 2 H), 1.80 ~ 1.78 (m, 2 H), 1.70 ~ 1.67 (m, 1 H), 1.34 ~ 1.17 (m, 5 H); ^{13}C NMR (150 MHz, $CDCl_3$): δ 179.0,

162.8, 133.8, 130.3, 128.6, 126.5, 99.9, 93.8, 41.7, 29.7, 29.5, 25.6, 25.5 (one carbon merged with others); HRMS calcd. for $C_{16}H_{18}O_3$: 258.1256; found: 258.1258.

Spectral data for (*Z*)-4,8-diphenyl-7,8-dihydro-1,5-dioxocin-2(6*H*)-one (6f).

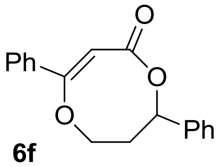

6f

White solid; ^{1}H NMR (600 MHz, $CDCl_3$): δ 7.68 ~ 7.66 (m, 2 H), 7.44 ~ 7.31 (m, 8 H), 5.67 (dd, *J* = 10.5, 2.7 Hz, 1 H), 5.56 (s, 1 H), 4.61 ~ 4.58 (m, 1 H), 4.48 (td, *J* = 12.6, 2.4 Hz, 1 H), 2.48 ~ 2.42 (m, 1 H), 2.21 ~ 2.16 (m, 1 H); ^{13}C NMR (150 MHz, $CDCl_3$): δ 167.8, 163.6, 138.6, 135.0, 130.6, 128.6, 128.5, 128.3, 126.7, 125.9, 89.7, 76.7, 67.3, 37.9; HRMS calcd. for $C_{18}H_{16}O_3$: 280.1099; found: 280.1100.

Spectral data for (*Z*)-ethyl 3-benzamido-3-phenylacrylate (3g').

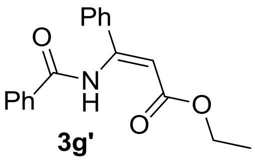

3g'

Colorless liquid; ^{1}H NMR (600 MHz, $CDCl_3$): δ 11.67 (s, 1 H), 7.99 ~ 7.97 (m, 2 H), 7.56 ~ 7.53 (m, 1 H), 7.49 ~ 7.35 (m, 7 H), 5.38 (s, 1 H), 4.23 (q, *J* = 7.2 Hz, 2 H), 1.31 (t, *J* = 7.2 Hz, 3 H); ^{13}C NMR (150 MHz, $CDCl_3$): δ 169.1, 164.8, 155.2, 136.1, 133.6, 132.5, 129.5, 128.8, 128.1, 127.9, 127.1, 101.6, 60.4, 14.3; HRMS calcd. for $C_{18}H_{17}NO_3$: 295.1208; found: 295.1207.

Spectral data for hept-2-ynoic acid (1a').

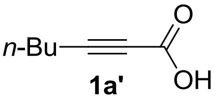

1a'

Colorless liquid; ^{1}H NMR (600 MHz, $CDCl_3$): δ 2.34 (t, *J* = 7.2 Hz, 2 H), 1.57 ~ 1.52 (m, 2 H), 1.44 ~ 1.38 (m, 2 H), 0.90 (t, *J* = 7.5 Hz, 3 H); ^{13}C NMR (150 MHz, $CDCl_3$): δ 158.1, 92.7, 72.6, 29.3, 21.9, 18.4, 13.4.

Spectral data for *N*-(tert-butyl)benzamide (2a-H).

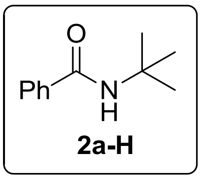

White solid; ^{1}H NMR (600 MHz, $CDCl_3$): δ 7.70 ~ 7.68 (m, 2 H), 7.45 ~ 7.37 (m, 3 H), 5.92 (bs, 1 H), 1.45 (s, 9 H); ^{13}C NMR (150 MHz, $CDCl_3$): δ 166.9, 135.9, 131.0, 128.4, 126.7, 51.6, 28.9.

(4) NOE of compound ethyl 4,6-dibutyl-2-phenylnicotinate (5m).

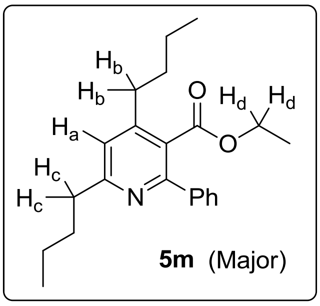

Sr. no.	Irradiation	Intensity increase % (Key peaks)
1)	H_a (δ 6.97)	H_b (δ 2.80, 2.18%), H_c (δ 2.65, 1.76%)
2)	H_b (δ 2.80)	H_a (δ 6.97, 1.39%), H_d (δ 4.05, 0.16%)
3)	H_c (δ 2.65)	H_a (δ 6.97, 0.84%)

NOE of compound (4,6-dibutyl-2-phenylpyridin-3-yl)(phenyl)methanone (5n).

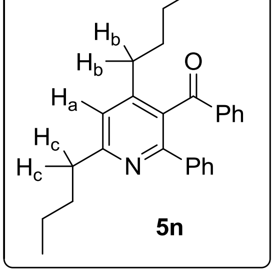

Sr. no.	Irradiation	Intensity increase % (Key peaks)
1)	H_a (δ 7.09)	H_b (δ 2.89, 1.94%), H_c (δ 2.52, 1.87%)
2)	H_b (δ 2.89)	H_a (δ 7.09, 1.27%)
3)	H_c (δ 2.52)	H_a (δ 7.09, 1.25%)

NOE of compound (*Z*)-ethyl 3-benzamido-3-phenylacrylate (3g').

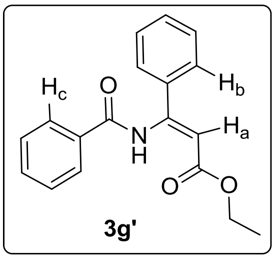

Sr. no.	Irradiation	Intensity increase % (Key peaks)
1)	H_a (δ 5.38)	H_b (δ 7.40 ~ 7.35, 5.19%)
2)	NH (δ 11.67)	H_c (δ 7.99 ~ 7.97, 15.40%)

(5) a) X-Ray crystallographic structure and data for compound (3g):

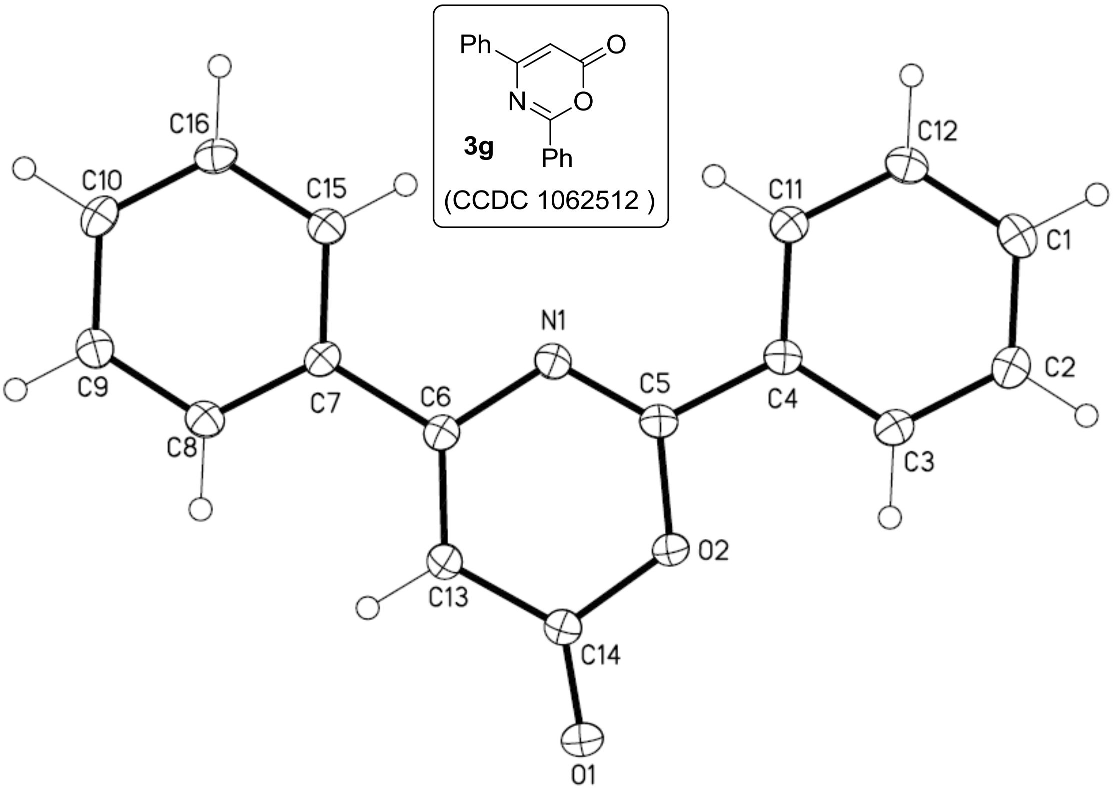

Table 1. Crystal data and structure refinement for 140928LT.

Identification code	140928LT	
Empirical formula	C16 H11 N O2	
Formula weight	249.26	
Temperature	100(2) K	
Wavelength	0.71073 Å	
Crystal system	Monoclinic	
Space group	P 21/c	
Unit cell dimensions	a = 15.281(4) Å	α= 90°.
	b = 3.8302(9) Å	β= 125.705(11)°.
	c = 24.754(4) Å	γ = 90°.
Volume	1176.5(5) Å ^{3}	
Z	4	
Density (calculated)	1.407 Mg/m^3	
Absorption coefficient	0.094 mm^{-1}	
F(000)	520	
Crystal size	0.30 x 0.10 x 0.02 mm^3	
Theta range for data collection	1.641 to 30.051°.	
Index ranges	-21<=h<=21, -5<=k<=5, -34<=l<=34	
Reflections collected	18434	

Independent reflections	3418 [R(int) = 0.0753]
Completeness to theta = 25.242°	99.7 %
Absorption correction	Semi-empirical from equivalents
Max. and min. transmission	0.9485 and 0.7481
Refinement method	Full-matrix least-squares on F^2
Data / restraints / parameters	3418 / 0 / 172
Goodness-of-fit on F^2	1.111
Final R indices [I>2sigma(I)]	R1 = 0.0718, wR2 = 0.2014
R indices (all data)	R1 = 0.0897, wR2 = 0.2162
Extinction coefficient	n/a
Largest diff. peak and hole	0.936 and -0.425 e.Å $^{-3}$

Table 2. Atomic coordinates (x 10^4) and equivalent isotropic displacement parameters (Å ^{2}x 10^3) for 140928LT. U(eq) is defined as one third of the trace of the orthogonalized U^{ij} tensor.

	x	y	z	U(eq)
O(1)	5436(1)	472(6)	4383(1)	31(1)
O(2)	4237(1)	2609(4)	3377(1)	18(1)
N(1)	2587(1)	4984(4)	3059(1)	14(1)
C(1)	2660(2)	5498(5)	1036(1)	17(1)
C(2)	3600(2)	3865(5)	1543(1)	17(1)
C(3)	3798(2)	3429(5)	2163(1)	15(1)
C(4)	3058(1)	4633(5)	2279(1)	13(1)
C(5)	3265(1)	4121(5)	2935(1)	13(1)
C(6)	2861(1)	4274(5)	3693(1)	13(1)
C(7)	2021(1)	5105(5)	3796(1)	12(1)
C(8)	2152(2)	4183(5)	4388(1)	15(1)
C(9)	1334(2)	4831(5)	4461(1)	16(1)
C(10)	378(2)	6393(5)	3952(1)	16(1)
C(11)	2109(2)	6260(5)	1766(1)	15(1)
C(12)	1917(2)	6674(5)	1146(1)	16(1)
C(13)	3823(2)	2822(6)	4171(1)	17(1)
C(14)	4571(2)	1863(6)	4027(1)	19(1)
C(15)	1060(2)	6697(5)	3288(1)	14(1)
C(16)	243(2)	7325(5)	3365(1)	16(1)

Table 3. Bond lengths [Å] and angles [°] for 140928LT.

O(1)-C(14)	1.204(2)
O(2)-C(5)	1.357(2)
O(2)-C(14)	1.403(2)
N(1)-C(5)	1.284(2)
N(1)-C(6)	1.396(2)
C(1)-C(2)	1.388(3)
C(1)-C(12)	1.389(3)
C(1)-H(1)	0.9500
C(2)-C(3)	1.390(3)
C(2)-H(3)	0.9500
C(3)-C(4)	1.397(3)
C(3)-H(4)	0.9500
C(4)-C(11)	1.398(3)
C(4)-C(5)	1.477(2)
C(6)-C(13)	1.357(3)
C(6)-C(7)	1.481(2)
C(7)-C(15)	1.398(3)
C(7)-C(8)	1.402(2)
C(8)-C(9)	1.387(3)
C(8)-H(9)	0.9500
C(9)-C(10)	1.390(3)
C(9)-H(10)	0.9500
C(10)-C(16)	1.389(3)
C(10)-H(2)	0.9500
C(11)-C(12)	1.392(3)
C(11)-H(6)	0.9500
C(12)-H(5)	0.9500
C(13)-C(14)	1.429(3)
C(13)-H(7)	0.9500
C(15)-C(16)	1.390(3)
C(15)-H(8)	0.9500
C(16)-H(11)	0.9500
C(5)-O(2)-C(14)	120.41(14)
C(5)-N(1)-C(6)	117.71(16)
C(2)-C(1)-C(12)	120.08(17)
C(2)-C(1)-H(1)	120.0

C(12)-C(1)-H(1)	120.0
C(1)-C(2)-C(3)	119.83(17)
C(1)-C(2)-H(3)	120.1
C(3)-C(2)-H(3)	120.1
C(2)-C(3)-C(4)	120.32(17)
C(2)-C(3)-H(4)	119.8
C(4)-C(3)-H(4)	119.8
C(3)-C(4)-C(11)	119.76(17)
C(3)-C(4)-C(5)	120.19(16)
C(11)-C(4)-C(5)	120.03(16)
N(1)-C(5)-O(2)	124.95(16)
N(1)-C(5)-C(4)	123.04(16)
O(2)-C(5)-C(4)	112.01(15)
C(13)-C(6)-N(1)	121.37(16)
C(13)-C(6)-C(7)	123.13(17)
N(1)-C(6)-C(7)	115.48(16)
C(15)-C(7)-C(8)	118.99(17)
C(15)-C(7)-C(6)	119.96(16)
C(8)-C(7)-C(6)	120.99(16)
C(9)-C(8)-C(7)	120.11(17)
C(9)-C(8)-H(9)	119.9
C(7)-C(8)-H(9)	119.9
C(8)-C(9)-C(10)	120.55(17)
C(8)-C(9)-H(10)	119.7
C(10)-C(9)-H(10)	119.7
C(16)-C(10)-C(9)	119.72(17)
C(16)-C(10)-H(2)	120.1
C(9)-C(10)-H(2)	120.1
C(12)-C(11)-C(4)	119.44(17)
C(12)-C(11)-H(6)	120.3
C(4)-C(11)-H(6)	120.3
C(1)-C(12)-C(11)	120.56(18)
C(1)-C(12)-H(5)	119.7
C(11)-C(12)-H(5)	119.7
C(6)-C(13)-C(14)	120.44(17)
C(6)-C(13)-H(7)	119.8
C(14)-C(13)-H(7)	119.8
O(1)-C(14)-O(2)	115.97(17)

O(1)-C(14)-C(13)	128.94(18)
O(2)-C(14)-C(13)	115.09(16)
C(16)-C(15)-C(7)	120.50(17)
C(16)-C(15)-H(8)	119.8
C(7)-C(15)-H(8)	119.8
C(10)-C(16)-C(15)	120.13(17)
C(10)-C(16)-H(11)	119.9
C(15)-C(16)-H(11)	119.9

Symmetry transformations used to generate equivalent atoms:

Table 4. Anisotropic displacement parameters ($Å^2 x 10^3$) for 140928LT. The anisotropic displacement factor exponent takes the form: $-2\pi^2[h^2 a^{*2}U^{11} + ... + 2 h k a^* b^* U^{12}]$

	U^{11}	U^{22}	U^{33}	U^{23}	U^{13}	U^{12}
O(1)	23(1)	48(1)	24(1)	16(1)	15(1)	19(1)
O(2)	15(1)	21(1)	17(1)	5(1)	10(1)	6(1)
N(1)	16(1)	9(1)	16(1)	2(1)	10(1)	0(1)
C(1)	23(1)	12(1)	17(1)	-1(1)	12(1)	-4(1)
C(2)	20(1)	12(1)	22(1)	-1(1)	14(1)	-1(1)
C(3)	15(1)	10(1)	19(1)	1(1)	10(1)	0(1)
C(4)	14(1)	8(1)	15(1)	0(1)	8(1)	-2(1)
C(5)	15(1)	7(1)	16(1)	1(1)	8(1)	0(1)
C(6)	16(1)	8(1)	17(1)	0(1)	10(1)	0(1)
C(7)	14(1)	7(1)	17(1)	-1(1)	10(1)	0(1)
C(8)	18(1)	10(1)	17(1)	1(1)	10(1)	1(1)
C(9)	21(1)	11(1)	18(1)	0(1)	12(1)	0(1)
C(10)	18(1)	10(1)	23(1)	-3(1)	14(1)	-1(1)
C(11)	16(1)	11(1)	18(1)	1(1)	10(1)	0(1)
C(12)	18(1)	11(1)	18(1)	2(1)	9(1)	0(1)
C(13)	18(1)	20(1)	17(1)	4(1)	11(1)	4(1)
C(14)	18(1)	24(1)	18(1)	5(1)	11(1)	5(1)
C(15)	17(1)	10(1)	16(1)	0(1)	9(1)	1(1)
C(16)	17(1)	11(1)	19(1)	0(1)	9(1)	3(1)

Table 5. Hydrogen coordinates (x 10^4) and isotropic displacement parameters ($Å^2 x 10^3$) for 140928LT.

	x	y	z	U(eq)
H(1)	2524	5813	613	20
H(3)	4106	3048	1467	20
H(4)	4441	2307	2510	18
H(9)	2802	3114	4739	18
H(10)	1427	4201	4863	19
H(2)	-180	6821	4004	19
H(6)	1600	7076	1840	18
H(5)	1271	7770	796	20
H(7)	4001	2439	4603	21
H(8)	964	7353	2886	17
H(11)	-409	8395	3016	20

b) X-Ray crystallographic structure and data for compound (6a):

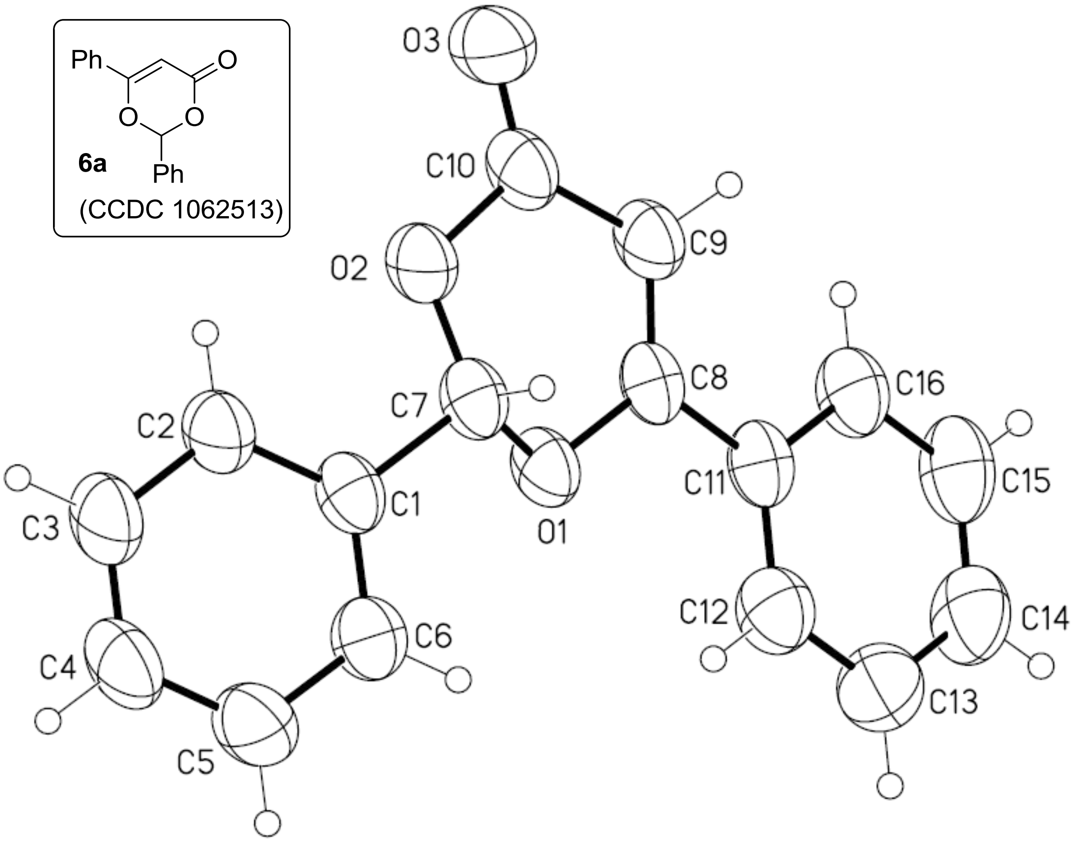

Table 1. Crystal data and structure refinement for 141246_0m.

Identification code	141246_0m	
Empirical formula	C16 H12 O3	
Formula weight	252.26	
Temperature	296(2) K	
Wavelength	0.71073 Å	
Crystal system	Monoclinic	
Space group	P 21/n	
Unit cell dimensions	a = 6.0914(7) Å	α= 90°.
	b = 8.5177(11) Å	β= 90.598(7)°.
	c = 23.947(3) Å	γ = 90°.
Volume	1242.4(3) $Å^3$	
Z	4	
Density (calculated)	1.349 Mg/m^3	
Absorption coefficient	0.093 mm^{-1}	
F(000)	528	
Crystal size	0.30 x 0.05 x 0.05 mm^3	
Theta range for data collection	0.850 to 26.424°.	
Index ranges	-7<=h<=7, -10<=k<=10, -29<=l<=29	
Reflections collected	8860	
Independent reflections	2531 [R(int) = 0.0528]	
Completeness to theta = 25.242°	99.6 %	
Absorption correction	Semi-empirical from equivalents	
Max. and min. transmission	0.9485 and 0.7225	
Refinement method	Full-matrix least-squares on F^2	
Data / restraints / parameters	2531 / 0 / 174	
Goodness-of-fit on F^2	1.388	
Final R indices [I>2sigma(I)]	R1 = 0.1269, wR2 = 0.3426	
R indices (all data)	R1 = 0.1690, wR2 = 0.3920	
Extinction coefficient	0.27(5)	
Largest diff. peak and hole	0.866 and -1.044 e.$Å^{-3}$	

Table 2. Atomic coordinates (x 10^4) and equivalent isotropic displacement parameters ($Å^2$x 10^3) for 141246_0m. U(eq) is defined as one third of the trace of the orthogonalized U^{ij} tensor.

	x	y	z	U(eq)
C(1)	4946(8)	6899(5)	3242(2)	49(1)

C(2)	6341(10)	6736(6)	2793(2)	62(1)
C(3)	5650(11)	5880(7)	2330(2)	71(2)
C(4)	3641(10)	5194(7)	2310(2)	68(2)
C(5)	2253(10)	5353(7)	2758(2)	72(2)
C(6)	2902(9)	6213(7)	3224(2)	60(1)
C(7)	5653(8)	7843(5)	3747(2)	48(1)
C(8)	5804(8)	7754(5)	4710(2)	48(1)
C(9)	7512(8)	8750(6)	4687(2)	53(1)
C(10)	8836(9)	8857(6)	4185(2)	54(1)
C(11)	4600(8)	7309(6)	5217(2)	50(1)
C(12)	2561(9)	6618(7)	5180(2)	63(1)
C(13)	1380(11)	6245(8)	5650(2)	75(2)
C(14)	2294(12)	6548(7)	6168(2)	75(2)
C(15)	4333(12)	7224(7)	6216(2)	72(2)
C(16)	5517(10)	7601(7)	5748(2)	60(1)
O(1)	5066(6)	7014(4)	4240(1)	51(1)
O(2)	7928(6)	8103(4)	3728(1)	57(1)
O(3)	10624(6)	9401(5)	4147(1)	71(1)

Table 3. Bond lengths [Å] and angles [°] for 141246_0m.

C(1)-C(6)	1.375(7)
C(1)-C(2)	1.384(7)
C(1)-C(7)	1.511(6)
C(2)-C(3)	1.389(7)
C(2)-H(2)	0.9300
C(3)-C(4)	1.357(8)
C(3)-H(3)	0.9300
C(4)-C(5)	1.379(9)
C(4)-H(4)	0.9300
C(5)-C(6)	1.387(7)
C(5)-H(5)	0.9300
C(6)-H(6)	0.9300
C(7)-O(2)	1.404(6)
C(7)-O(1)	1.423(5)
C(7)-H(7)	0.9800
C(8)-C(9)	1.344(7)

C(8)-O(1)	1.364(5)
C(8)-C(11)	1.473(7)
C(9)-C(10)	1.458(7)
C(9)-H(9)	0.9300
C(10)-O(3)	1.188(6)
C(10)-O(2)	1.380(5)
C(11)-C(12)	1.377(8)
C(11)-C(16)	1.407(7)
C(12)-C(13)	1.380(8)
C(12)-H(12)	0.9300
C(13)-C(14)	1.379(8)
C(13)-H(13)	0.9300
C(14)-C(15)	1.373(10)
C(14)-H(14)	0.9300
C(15)-C(16)	1.377(8)
C(15)-H(15)	0.9300
C(16)-H(16)	0.9300
C(6)-C(1)-C(2)	119.7(4)
C(6)-C(1)-C(7)	120.2(4)
C(2)-C(1)-C(7)	120.2(4)
C(1)-C(2)-C(3)	119.3(5)
C(1)-C(2)-H(2)	120.3
C(3)-C(2)-H(2)	120.3
C(4)-C(3)-C(2)	121.3(5)
C(4)-C(3)-H(3)	119.3
C(2)-C(3)-H(3)	119.3
C(3)-C(4)-C(5)	119.4(5)
C(3)-C(4)-H(4)	120.3
C(5)-C(4)-H(4)	120.3
C(4)-C(5)-C(6)	120.3(5)
C(4)-C(5)-H(5)	119.8
C(6)-C(5)-H(5)	119.8
C(1)-C(6)-C(5)	120.0(5)
C(1)-C(6)-H(6)	120.0
C(5)-C(6)-H(6)	120.0
O(2)-C(7)-O(1)	111.3(4)
O(2)-C(7)-C(1)	109.3(4)

O(1)-C(7)-C(1)	109.1(4)
O(2)-C(7)-H(7)	109.1
O(1)-C(7)-H(7)	109.1
C(1)-C(7)-H(7)	109.1
C(9)-C(8)-O(1)	120.4(4)
C(9)-C(8)-C(11)	126.0(4)
O(1)-C(8)-C(11)	113.5(4)
C(8)-C(9)-C(10)	120.5(4)
C(8)-C(9)-H(9)	119.7
C(10)-C(9)-H(9)	119.7
O(3)-C(10)-O(2)	118.7(4)
O(3)-C(10)-C(9)	127.1(4)
O(2)-C(10)-C(9)	113.9(4)
C(12)-C(11)-C(16)	118.9(5)
C(12)-C(11)-C(8)	120.9(4)
C(16)-C(11)-C(8)	120.2(5)
C(11)-C(12)-C(13)	121.6(5)
C(11)-C(12)-H(12)	119.2
C(13)-C(12)-H(12)	119.2
C(14)-C(13)-C(12)	118.8(6)
C(14)-C(13)-H(13)	120.6
C(12)-C(13)-H(13)	120.6
C(15)-C(14)-C(13)	120.7(5)
C(15)-C(14)-H(14)	119.7
C(13)-C(14)-H(14)	119.7
C(14)-C(15)-C(16)	120.7(5)
C(14)-C(15)-H(15)	119.6
C(16)-C(15)-H(15)	119.6
C(15)-C(16)-C(11)	119.3(6)
C(15)-C(16)-H(16)	120.4
C(11)-C(16)-H(16)	120.4
C(8)-O(1)-C(7)	111.8(3)
C(10)-O(2)-C(7)	115.8(4)

Symmetry transformations used to generate equivalent atoms:

Table 4. Anisotropic displacement parameters ($Å^2 x 10^3$) for 141246_0m. The anisotropic displacement factor exponent takes the form: $-2\pi^2[h^2 a^{*2}U^{11} + ... + 2 h k a^* b^* U^{12}]$

	U^{11}	U^{22}	U^{33}	U^{23}	U^{13}	U^{12}
C(1)	65(3)	47(2)	34(2)	0(2)	-4(2)	1(2)
C(2)	71(3)	68(3)	46(3)	-8(2)	6(2)	-10(3)
C(3)	93(4)	81(4)	41(3)	-12(3)	11(3)	-13(3)
C(4)	92(4)	69(3)	43(3)	-11(2)	-5(3)	-12(3)
C(5)	75(4)	84(4)	59(3)	-10(3)	-5(3)	-15(3)
C(6)	64(3)	72(3)	45(3)	-7(2)	-1(2)	-5(3)
C(7)	62(3)	47(2)	37(2)	-1(2)	-2(2)	3(2)
C(8)	62(3)	47(2)	35(2)	-4(2)	-2(2)	6(2)
C(9)	65(3)	58(3)	37(2)	-5(2)	-1(2)	-6(2)
C(10)	68(3)	60(3)	35(2)	-5(2)	-6(2)	-5(2)
C(11)	64(3)	48(3)	38(2)	-1(2)	-2(2)	10(2)
C(12)	72(4)	66(3)	51(3)	-2(2)	0(2)	-1(3)
C(13)	83(4)	72(4)	71(4)	0(3)	14(3)	-12(3)
C(14)	101(5)	74(4)	49(3)	9(3)	20(3)	-4(4)
C(15)	100(5)	78(4)	38(3)	1(2)	3(3)	10(3)
C(16)	76(3)	67(3)	37(2)	-3(2)	0(2)	3(3)
O(1)	67(2)	51(2)	34(2)	-2(1)	-1(1)	-5(2)
O(2)	66(2)	69(2)	35(2)	-7(1)	2(1)	-10(2)
O(3)	72(3)	90(3)	50(2)	-5(2)	1(2)	-26(2)

Table 5. Hydrogen coordinates (x 10^4) and isotropic displacement parameters (Å ^{2}x 10 ^{3}) for 141246_0m.

	x	y	z	U(eq)
H(2)	7726	7195	2802	74
H(3)	6584	5774	2028	86
H(4)	3203	4622	1998	81
H(5)	876	4882	2748	87
H(6)	1955	6325	3523	73
H(7)	4893	8857	3741	58
H(9)	7852	9377	4994	64
H(12)	1965	6397	4829	76

H(13)	-8	5798	5618	90
H(14)	1521	6292	6489	90
H(15)	4922	7429	6569	86
H(16)	6908	8043	5783	72

c) X-Ray crystallographic structure and data for compound (6f):

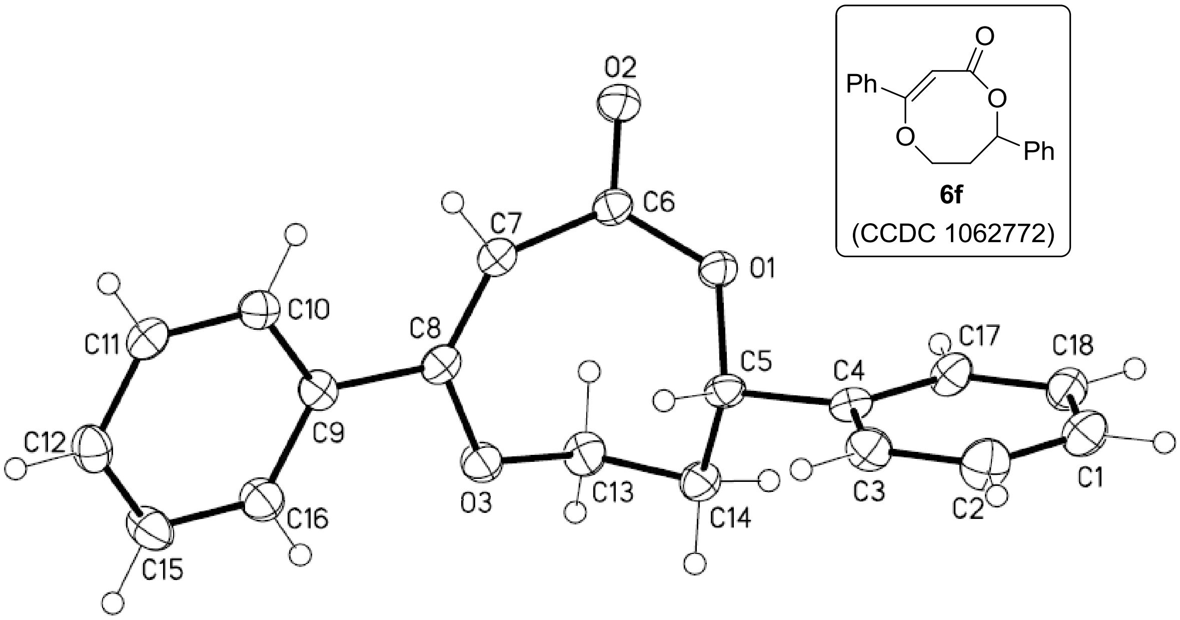

Table 1. Crystal data and structure refinement for 150135LT_0m.

Identification code	150135LT_0m	
Empirical formula	C18 H16 O3	
Formula weight	280.31	
Temperature	100(2) K	
Wavelength	0.71073 Å	
Crystal system	Monoclinic	
Space group	P 21	
Unit cell dimensions	a = 7.9460(7) Å	α= 90°.
	b = 9.4371(9) Å	β= 100.506(3)°.
	c = 9.6309(10) Å	γ = 90°.
Volume	710.09(12) Å ^{3}	
Z	2	
Density (calculated)	1.311 Mg/m^3	
Absorption coefficient	0.089 mm^{-1}	
F(000)	296	
Crystal size	0.25 x 0.20 x 0.10 mm^3	

Theta range for data collection	2.151 to 26.425°.
Index ranges	-5<=h<=9, -11<=k<=11, -12<=l<=12
Reflections collected	5336
Independent reflections	2853 [R(int) = 0.0289]
Completeness to theta = 25.242°	99.8 %
Absorption correction	Semi-empirical from equivalents
Max. and min. transmission	0.9485 and 0.8629
Refinement method	Full-matrix least-squares on F^2
Data / restraints / parameters	2853 / 1 / 191
Goodness-of-fit on F^2	1.085
Final R indices [I>2sigma(I)]	R1 = 0.0380, wR2 = 0.0998
R indices (all data)	R1 = 0.0446, wR2 = 0.1154
Absolute structure parameter	-0.1(17)
Extinction coefficient	n/a
Largest diff. peak and hole	0.204 and -0.267 e.Å $^{-3}$

Table 2. Atomic coordinates (x 10^4) and equivalent isotropic displacement parameters (Å ^{2}x 10^3) for 150135LT_0m. U(eq) is defined as one third of the trace of the orthogonalized U^{ij} tensor.

	x	y	z	U(eq)
O(1)	5400(2)	8933(2)	8638(2)	20(1)
O(2)	5810(2)	7922(2)	10711(2)	26(1)
O(3)	1069(2)	8669(2)	7980(2)	23(1)
C(1)	8174(4)	10267(4)	4664(3)	29(1)
C(2)	7038(4)	9164(3)	4235(3)	30(1)
C(3)	5858(4)	8770(4)	5061(3)	24(1)
C(4)	5787(3)	9478(3)	6311(3)	19(1)
C(5)	4479(3)	9079(3)	7194(3)	18(1)
C(6)	4880(4)	8034(3)	9569(3)	18(1)
C(7)	3327(3)	7159(3)	9211(3)	19(1)
C(8)	1711(4)	7433(3)	8556(3)	18(1)
C(9)	328(3)	6348(3)	8394(3)	18(1)
C(10)	659(4)	4934(3)	8768(3)	20(1)
C(11)	-640(4)	3946(3)	8547(3)	21(1)
C(12)	-2306(4)	4343(3)	7952(3)	24(1)
C(13)	2008(4)	9972(3)	8256(3)	22(1)
C(14)	3125(4)	10220(3)	7170(3)	22(1)

C(15)	-2641(4)	5744(4)	7592(4)	27(1)
C(16)	-1345(4)	6744(3)	7812(3)	24(1)
C(17)	6922(4)	10589(3)	6729(3)	22(1)
C(18)	8102(4)	10978(3)	5909(4)	25(1)

Table 3. Bond lengths [Å] and angles [°] for 150135LT_0m.

O(1)-C(6)	1.353(4)
O(1)-C(5)	1.456(3)
O(2)-C(6)	1.213(3)
O(3)-C(8)	1.351(3)
O(3)-C(13)	1.438(4)
C(1)-C(18)	1.384(5)
C(1)-C(2)	1.390(5)
C(1)-H(1)	0.9500
C(2)-C(3)	1.387(4)
C(2)-H(16)	0.9500
C(3)-C(4)	1.387(4)
C(3)-H(15)	0.9500
C(4)-C(17)	1.394(4)
C(4)-C(5)	1.506(4)
C(5)-C(14)	1.519(4)
C(5)-H(12)	1.0000
C(6)-C(7)	1.472(4)
C(7)-C(8)	1.349(4)
C(7)-H(11)	0.9500
C(8)-C(9)	1.489(4)
C(9)-C(10)	1.395(4)
C(9)-C(16)	1.397(4)
C(10)-C(11)	1.378(4)
C(10)-H(10)	0.9500
C(11)-C(12)	1.394(4)
C(11)-H(9)	0.9500
C(12)-C(15)	1.381(5)
C(12)-H(2)	0.9500
C(13)-C(14)	1.508(4)
C(13)-H(3)	0.9900

C(13)-H(6)	0.9900
C(14)-H(5)	0.9900
C(14)-H(4)	0.9900
C(15)-C(16)	1.384(4)
C(15)-H(8)	0.9500
C(16)-H(7)	0.9500
C(17)-C(18)	1.381(4)
C(17)-H(14)	0.9500
C(18)-H(13)	0.9500

C(6)-O(1)-C(5)	122.1(2)
C(8)-O(3)-C(13)	121.2(2)
C(18)-C(1)-C(2)	119.5(3)
C(18)-C(1)-H(1)	120.2
C(2)-C(1)-H(1)	120.2
C(3)-C(2)-C(1)	120.0(3)
C(3)-C(2)-H(16)	120.0
C(1)-C(2)-H(16)	120.0
C(2)-C(3)-C(4)	120.5(3)
C(2)-C(3)-H(15)	119.8
C(4)-C(3)-H(15)	119.8
C(3)-C(4)-C(17)	119.1(3)
C(3)-C(4)-C(5)	120.7(3)
C(17)-C(4)-C(5)	120.1(3)
O(1)-C(5)-C(4)	106.5(2)
O(1)-C(5)-C(14)	108.2(2)
C(4)-C(5)-C(14)	111.9(2)
O(1)-C(5)-H(12)	110.0
C(4)-C(5)-H(12)	110.0
C(14)-C(5)-H(12)	110.0
O(2)-C(6)-O(1)	116.5(3)
O(2)-C(6)-C(7)	120.7(3)
O(1)-C(6)-C(7)	122.7(2)
C(8)-C(7)-C(6)	133.5(3)
C(8)-C(7)-H(11)	113.2
C(6)-C(7)-H(11)	113.2
C(7)-C(8)-O(3)	127.8(3)
C(7)-C(8)-C(9)	122.6(3)

O(3)-C(8)-C(9)	109.5(2)
C(10)-C(9)-C(16)	118.7(3)
C(10)-C(9)-C(8)	122.0(2)
C(16)-C(9)-C(8)	119.2(3)
C(11)-C(10)-C(9)	120.4(3)
C(11)-C(10)-H(10)	119.8
C(9)-C(10)-H(10)	119.8
C(10)-C(11)-C(12)	120.7(3)
C(10)-C(11)-H(9)	119.7
C(12)-C(11)-H(9)	119.7
C(15)-C(12)-C(11)	119.1(3)
C(15)-C(12)-H(2)	120.5
C(11)-C(12)-H(2)	120.5
O(3)-C(13)-C(14)	110.8(2)
O(3)-C(13)-H(3)	109.5
C(14)-C(13)-H(3)	109.5
O(3)-C(13)-H(6)	109.5
C(14)-C(13)-H(6)	109.5
H(3)-C(13)-H(6)	108.1
C(13)-C(14)-C(5)	112.6(2)
C(13)-C(14)-H(5)	109.1
C(5)-C(14)-H(5)	109.1
C(13)-C(14)-H(4)	109.1
C(5)-C(14)-H(4)	109.1
H(5)-C(14)-H(4)	107.8
C(12)-C(15)-C(16)	120.7(3)
C(12)-C(15)-H(8)	119.6
C(16)-C(15)-H(8)	119.6
C(15)-C(16)-C(9)	120.4(3)
C(15)-C(16)-H(7)	119.8
C(9)-C(16)-H(7)	119.8
C(18)-C(17)-C(4)	120.4(3)
C(18)-C(17)-H(14)	119.8
C(4)-C(17)-H(14)	119.8
C(17)-C(18)-C(1)	120.4(3)
C(17)-C(18)-H(13)	119.8
C(1)-C(18)-H(13)	119.8

Symmetry transformations used to generate equivalent atoms:

Table 4. Anisotropic displacement parameters ($Å^2 x 10^3$) for 150135LT_0m. The anisotropic displacement factor exponent takes the form: $-2\pi^2[h^2 a^{*2}U^{11} + ... + 2 h k a^* b^* U^{12}]$

	U^{11}	U^{22}	U^{33}	U^{23}	U^{13}	U^{12}
O(1)	21(1)	21(1)	16(1)	2(1)	1(1)	-2(1)
O(2)	25(1)	29(1)	20(1)	4(1)	-3(1)	-6(1)
O(3)	21(1)	16(1)	32(1)	4(1)	2(1)	2(1)
C(1)	31(2)	36(2)	21(2)	4(1)	7(1)	-1(1)
C(2)	37(2)	33(2)	20(2)	-6(1)	7(1)	-4(2)
C(3)	27(1)	25(2)	19(2)	-5(1)	1(1)	-3(1)
C(4)	22(1)	19(1)	15(2)	1(1)	1(1)	3(1)
C(5)	21(1)	19(2)	13(1)	0(1)	0(1)	0(1)
C(6)	21(1)	15(1)	18(1)	1(1)	3(1)	1(1)
C(7)	22(1)	18(2)	18(2)	2(1)	4(1)	0(1)
C(8)	23(1)	17(1)	16(2)	0(1)	6(1)	1(1)
C(9)	18(1)	21(1)	16(1)	-4(1)	6(1)	2(1)
C(10)	21(1)	22(2)	18(2)	0(1)	4(1)	2(1)
C(11)	27(1)	18(2)	18(1)	1(1)	6(1)	1(1)
C(12)	24(1)	25(2)	24(2)	-5(1)	5(1)	-5(1)
C(13)	24(1)	13(1)	30(2)	0(1)	5(1)	1(1)
C(14)	23(1)	18(2)	24(2)	3(1)	2(1)	0(1)
C(15)	20(1)	25(2)	35(2)	-2(1)	0(1)	2(1)
C(16)	23(1)	20(2)	29(2)	1(1)	3(1)	1(1)
C(17)	29(2)	20(2)	18(2)	1(1)	6(1)	-1(1)
C(18)	27(2)	25(2)	22(2)	3(1)	2(1)	-5(1)

Table 5. Hydrogen coordinates ($x 10^4$) and isotropic displacement parameters ($Å^2 x 10^3$) for 150135LT_0m.

	x	y	z	U(eq)
H(1)	8994	10531	4107	35
H(16)	7070	8681	3374	36
H(15)	5093	8008	4769	29

H(12)	3925	8159	6858	22
H(11)	3501	6200	9501	23
H(10)	1787	4649	9177	24
H(9)	-398	2985	8802	25
H(2)	-3197	3659	7797	29
H(3)	2730	9934	9208	27
H(6)	1197	10771	8237	27
H(5)	3696	11152	7347	26
H(4)	2397	10250	6219	26
H(8)	-3772	6025	7188	33
H(7)	-1596	7706	7565	29
H(14)	6884	11082	7583	27
H(13)	8869	11738	6202	30

```
Current Data Parameters
NAME            SNK-5022
EXPNO                  1
PROCNO                 1

F2 - Acquisition Parameters
Date_           20141205
Time                8.17
INSTRUM            spect
PROBHD   5 mm QNP  1H/1
PULPROG               zg
TD                 33556
SOLVENT            CDCl3
NS                    16
DS                     0
SWH         12019.230 Hz
FIDRES       0.358184 Hz
AQ          1.3959796 sec
RG                    64
DW             41.600 usec
DE               6.50 usec
TE              294.9 K
D1         2.00000000 sec
MCREST   0 sec
MCWRK      0.01500000 sec

======== CHANNEL f1 ========
NUC1                  1H
P1                 10.00 usec
PL1      0 dB
SFO1       598.6035916 MHz

F2 - Processing parameters
SI                 32768
SF         598.6000302 MHz
WDW                   no
SSB      0
LB       0 Hz
GB       0
PC                  0.10
```

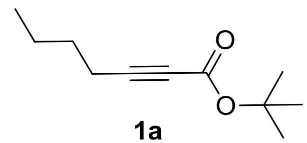

1a

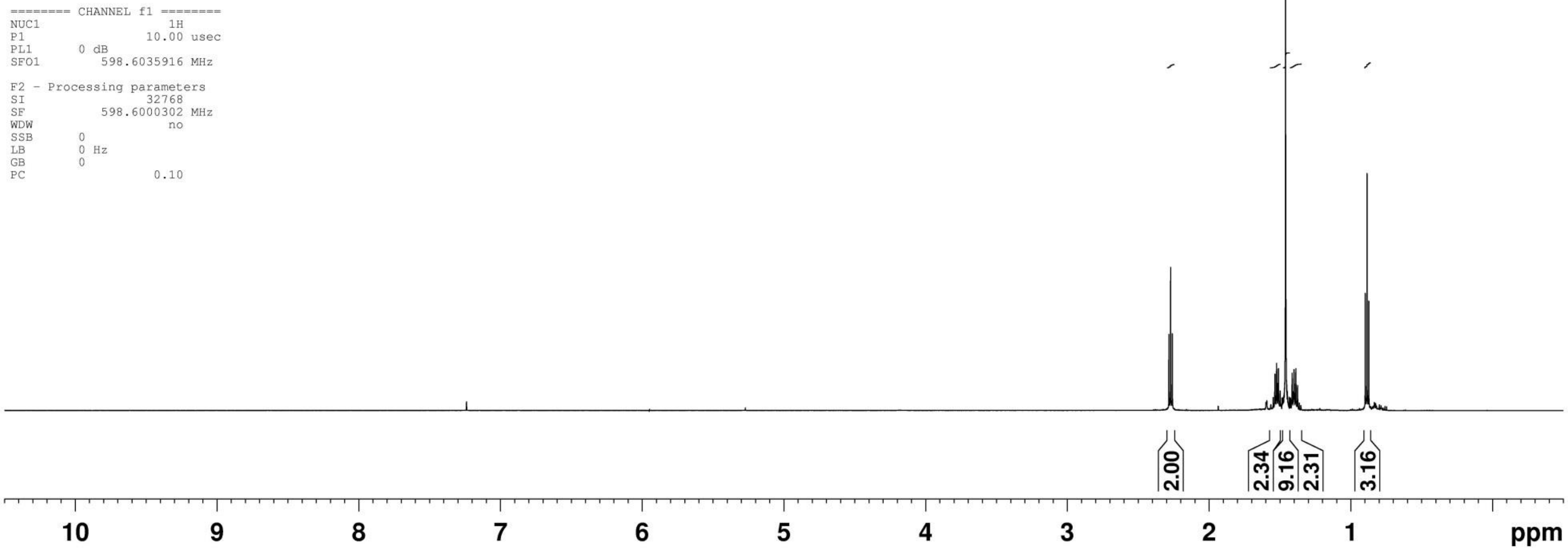

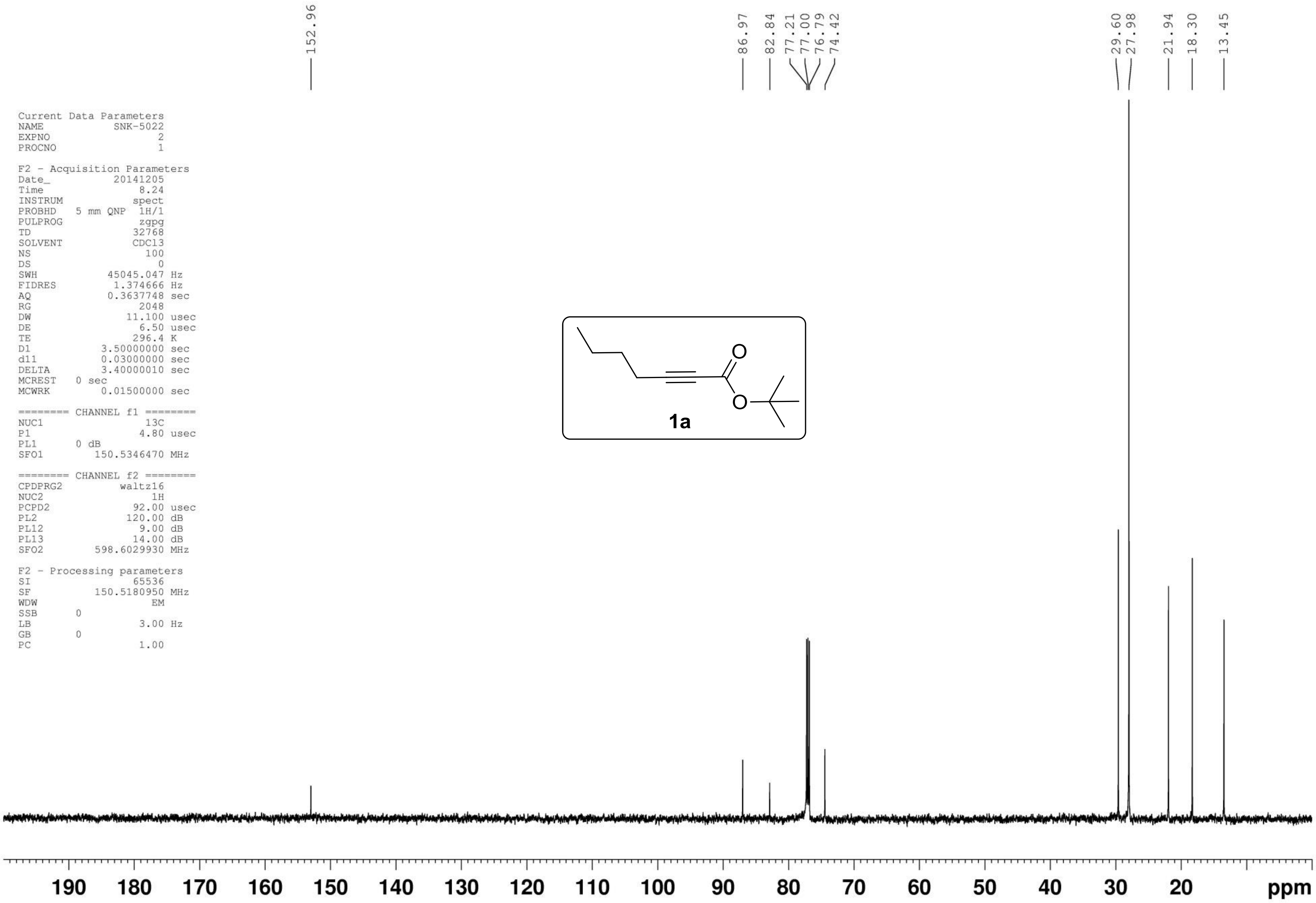
152.96
86.97
82.84
77.21
77.00
76.79
74.42
29.60
27.98
21.94
18.30
13.45
Current Data Parameters
NAME SNK-5022
EXPNO 2
PROCNO 1
F2 - Acquisition Parameters
Date_ 20141205
Time 8.24
INSTRUM spect
PROBHD 5 mm QNP 1H/1
PULPROG zgpg
TD 32768
SOLVENT CDCl3
NS 100
DS 0
SWH 45045.047 Hz
FIDRES 1.374666 Hz
AQ 0.3637748 sec
RG 2048
DW 11.100 usec
DE 6.50 usec
TE 296.4 K
D1 3.50000000 sec
d11 0.03000000 sec
DELTA 3.40000010 sec
MCREST 0 sec
MCWRK 0.01500000 sec
======== CHANNEL f1 ========
NUC1 13C
P1 4.80 usec
PL1 0 dB
SFO1 150.5346470 MHz
======== CHANNEL f2 ========
CPDPRG2 waltz16
NUC2 1H
PCPD2 92.00 usec
PL2 120.00 dB
PL12 9.00 dB
PL13 14.00 dB
SFO2 598.6029930 MHz
F2 - Processing parameters
SI 65536
SF 150.5180950 MHz
WDW EM
SSB 0
LB 3.00 Hz
GB 0
PC 1.00
O
O
1a
190 180 170 160 150 140 130 120 110 100 90 80 70 60 50 40 30 20 ppm

```
Current Data Parameters
NAME             SNK-5037
EXPNO                   1
PROCNO                  1

F2 - Acquisition Parameters
Date_            20141216
Time                12.53
INSTRUM             spect
PROBHD   5 mm QNP  1H/1
PULPROG                zg
TD                  33556
SOLVENT             CDCl3
NS                     16
DS                      0
SWH             12019.230 Hz
FIDRES           0.358184 Hz
AQ              1.3959796 sec
RG                     32
DW                 41.600 usec
DE                   6.50 usec
TE                  294.0 K
D1             2.00000000 sec
MCREST   0 sec
MCWRK          0.01500000 sec

======== CHANNEL f1 ========
NUC1                   1H
P1                  10.00 usec
PL1      0 dB
SFO1          598.6035916 MHz

F2 - Processing parameters
SI                  32768
SF            598.6000302 MHz
WDW                    no
SSB      0
LB       0 Hz
GB       0
PC                   0.10
```

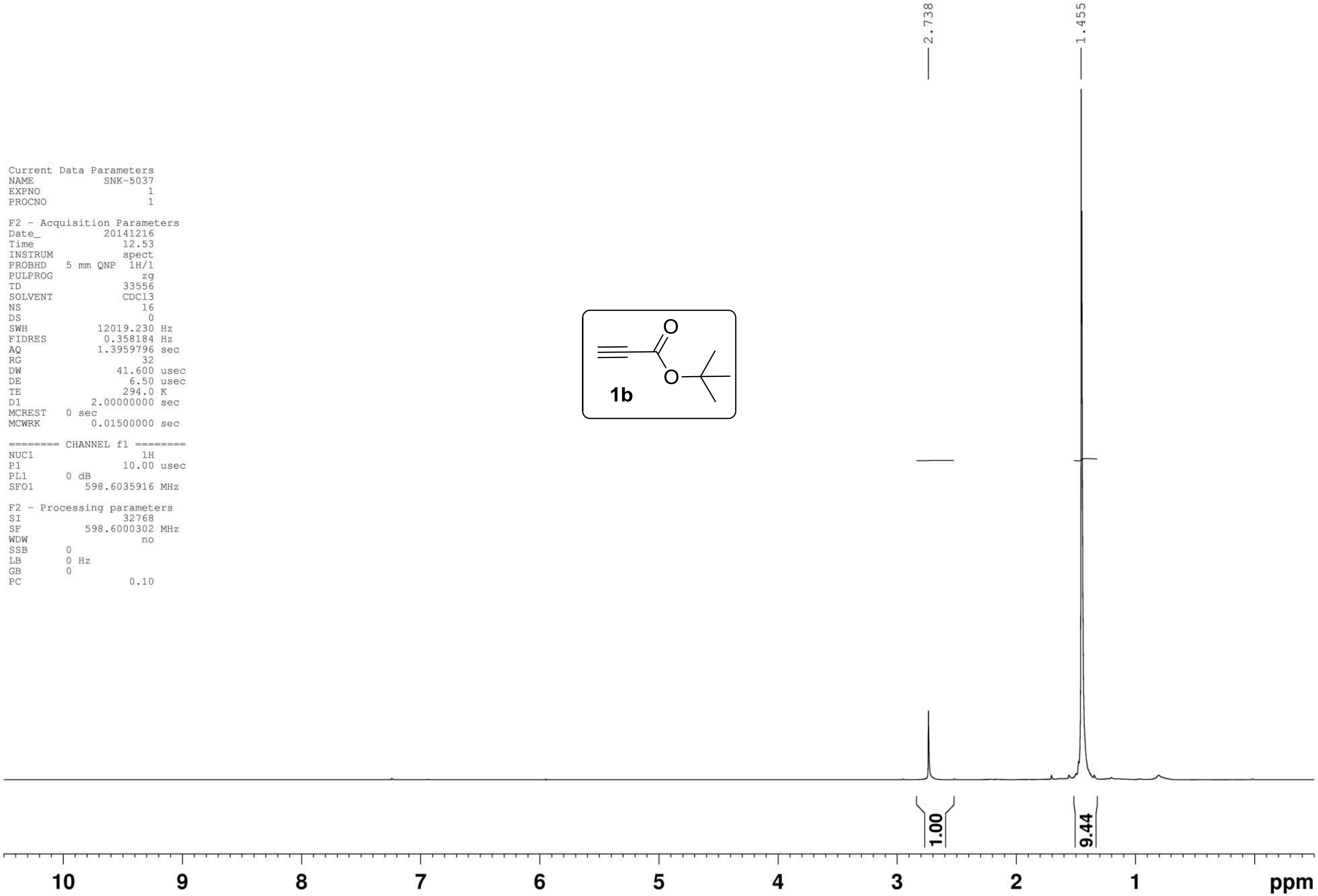

```
Current Data Parameters
NAME            SNK-5037
EXPNO                  2
PROCNO                 1

F2 - Acquisition Parameters
Date_           20141216
Time               13.03
INSTRUM            spect
PROBHD   5 mm QNP  1H/1
PULPROG             zgpg
TD                 32768
SOLVENT            CDCl3
NS                   159
DS                     0
SWH            45045.047 Hz
FIDRES          1.374666 Hz
AQ             0.3637748 sec
RG                  2048
DW                11.100 usec
DE                  6.50 usec
TE                 295.9 K
D1            3.50000000 sec
d11           0.03000000 sec
DELTA         3.40000010 sec
MCREST   0 sec
MCWRK         0.01500000 sec

======== CHANNEL f1 ========
NUC1                 13C
P1                  4.80 usec
PL1      0 dB
SFO1        150.5346470 MHz

======== CHANNEL f2 ========
CPDPRG2          waltz16
NUC2                  1H
PCPD2              92.00 usec
PL2               120.00 dB
PL12                9.00 dB
PL13               14.00 dB
SFO2        598.6029930 MHz

F2 - Processing parameters
SI                 65536
SF          150.5180985 MHz
WDW                   EM
SSB      0
LB                  3.00 Hz
GB       0
PC                  1.00
```

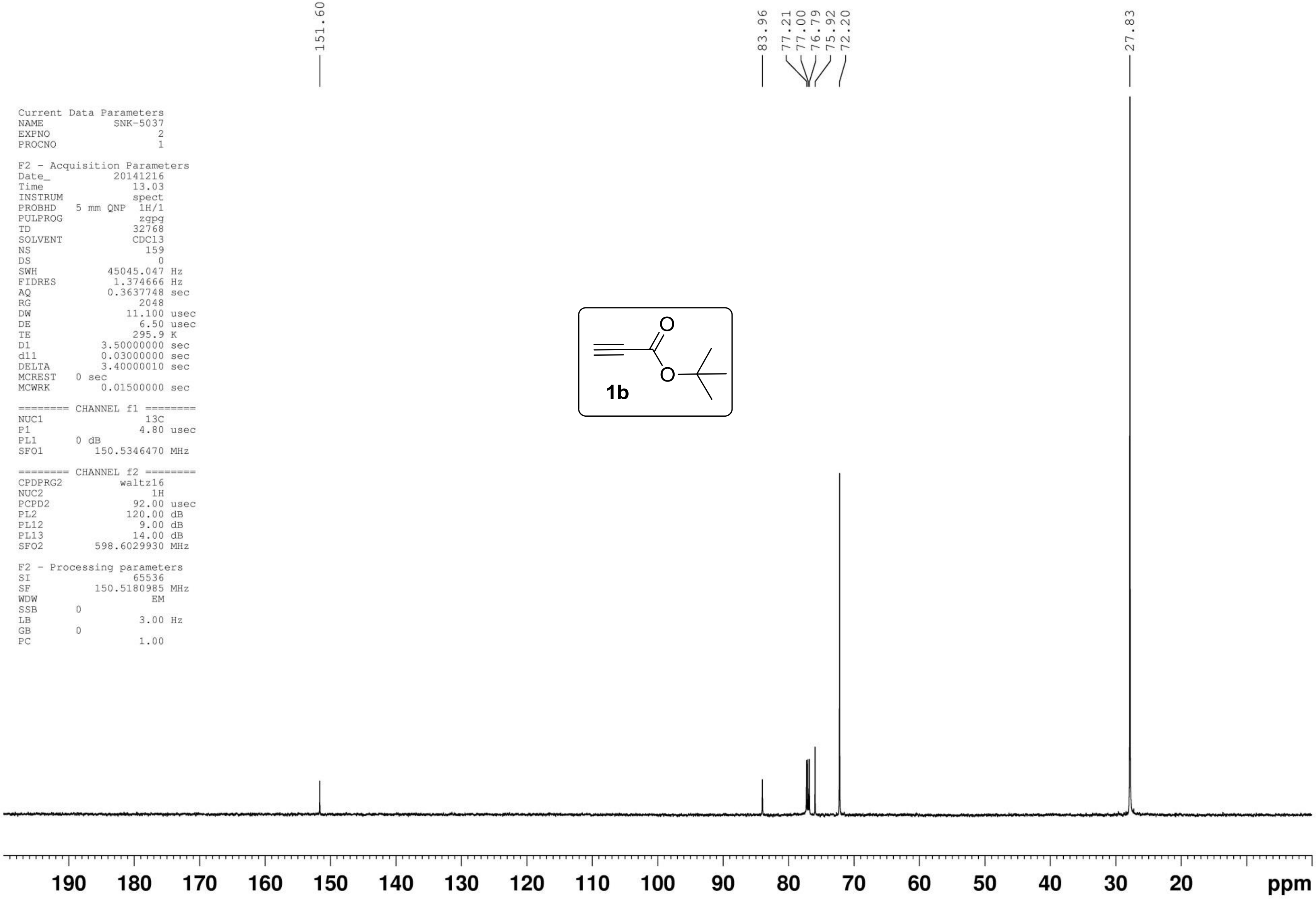

```
Current Data Parameters
NAME            SNK-5027
EXPNO                  1
PROCNO                 1

F2 - Acquisition Parameters
Date_           20141211
Time                8.17
INSTRUM            spect
PROBHD   5 mm QNP  1H/1
PULPROG               zg
TD                 33556
SOLVENT            CDCl3
NS                    16
DS                     0
SWH             8389.262 Hz
FIDRES          0.250008 Hz
AQ             1.9999876 sec
RG                   128
DW                59.600 usec
DE                  6.50 usec
TE                 295.0 K
D1            2.00000000 sec
MCREST   0 sec
MCWRK         0.01500000 sec

======== CHANNEL f1 ========
NUC1                  1H
P1                 10.00 usec
PL1      0 dB
SFO1         598.6029930 MHz

F2 - Processing parameters
SI                 32768
SF           598.6000306 MHz
WDW                   no
SSB      0
LB       0 Hz
GB       0
PC                  0.10
```

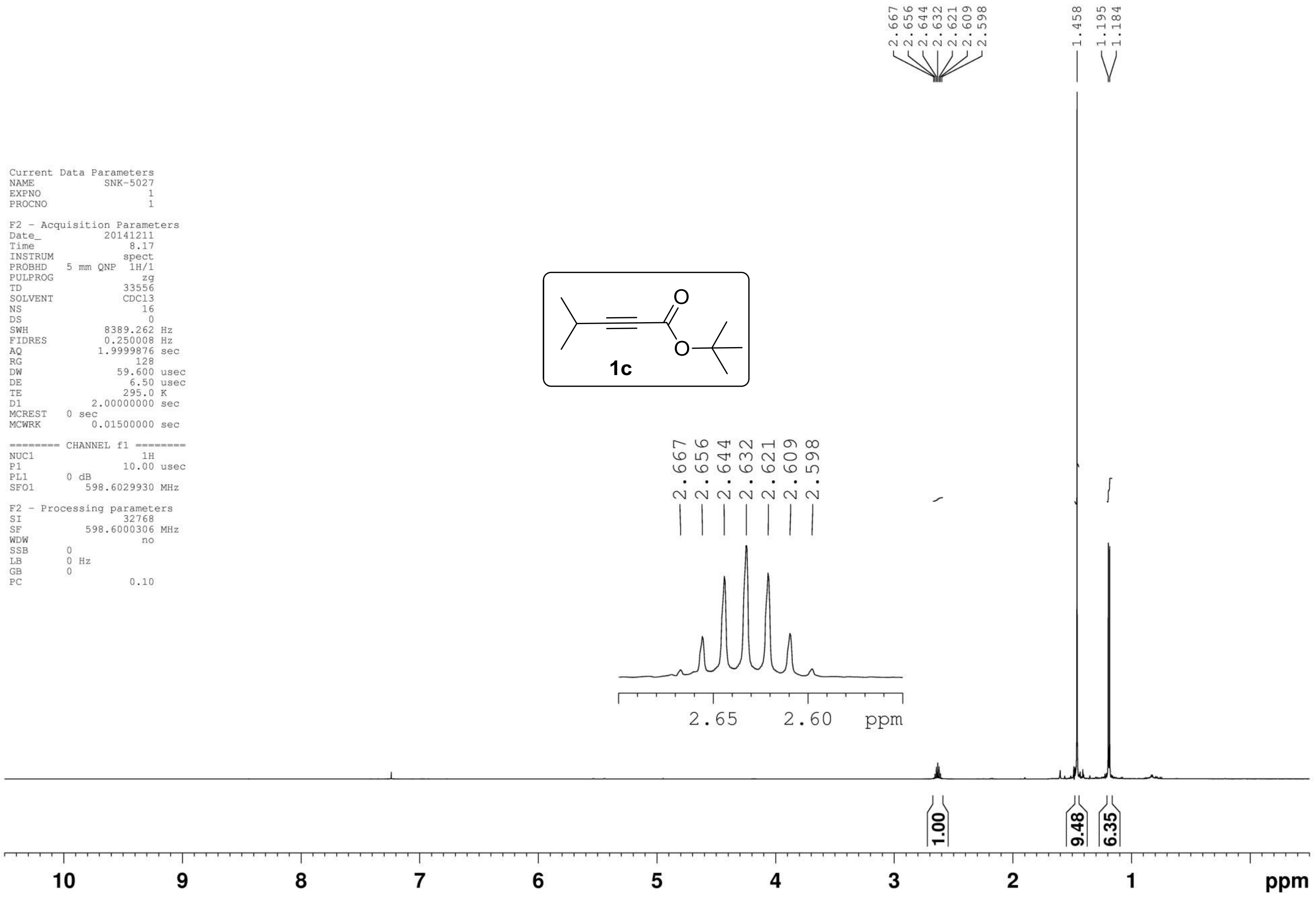

Current Data Parameters
NAME SNK-5027
EXPNO 2
PROCNO 1

F2 - Acquisition Parameters
Date_ 20141211
Time 8.24
INSTRUM spect
PROBHD 5 mm QNP 1H/1
PULPROG zgpg
TD 32768
SOLVENT CDCl3
NS 100
DS 0
SWH 45045.047 Hz
FIDRES 1.374666 Hz
AQ 0.3637748 sec
RG 2048
DW 11.100 usec
DE 6.50 usec
TE 296.2 K
D1 3.50000000 sec
d11 0.03000000 sec
DELTA 3.40000010 sec
MCREST 0 sec
MCWRK 0.01500000 sec

======== CHANNEL f1 ========
NUC1 13C
P1 4.80 usec
PL1 0 dB
SFO1 150.5346470 MHz

======== CHANNEL f2 ========
CPDPRG2 waltz16
NUC2 1H
PCPD2 92.00 usec
PL2 120.00 dB
PL12 9.00 dB
PL13 14.00 dB
SFO2 598.6029930 MHz

F2 - Processing parameters
SI 65536
SF 150.5180955 MHz
WDW EM
SSB 0
LB 3.00 Hz
GB 0
PC 1.00

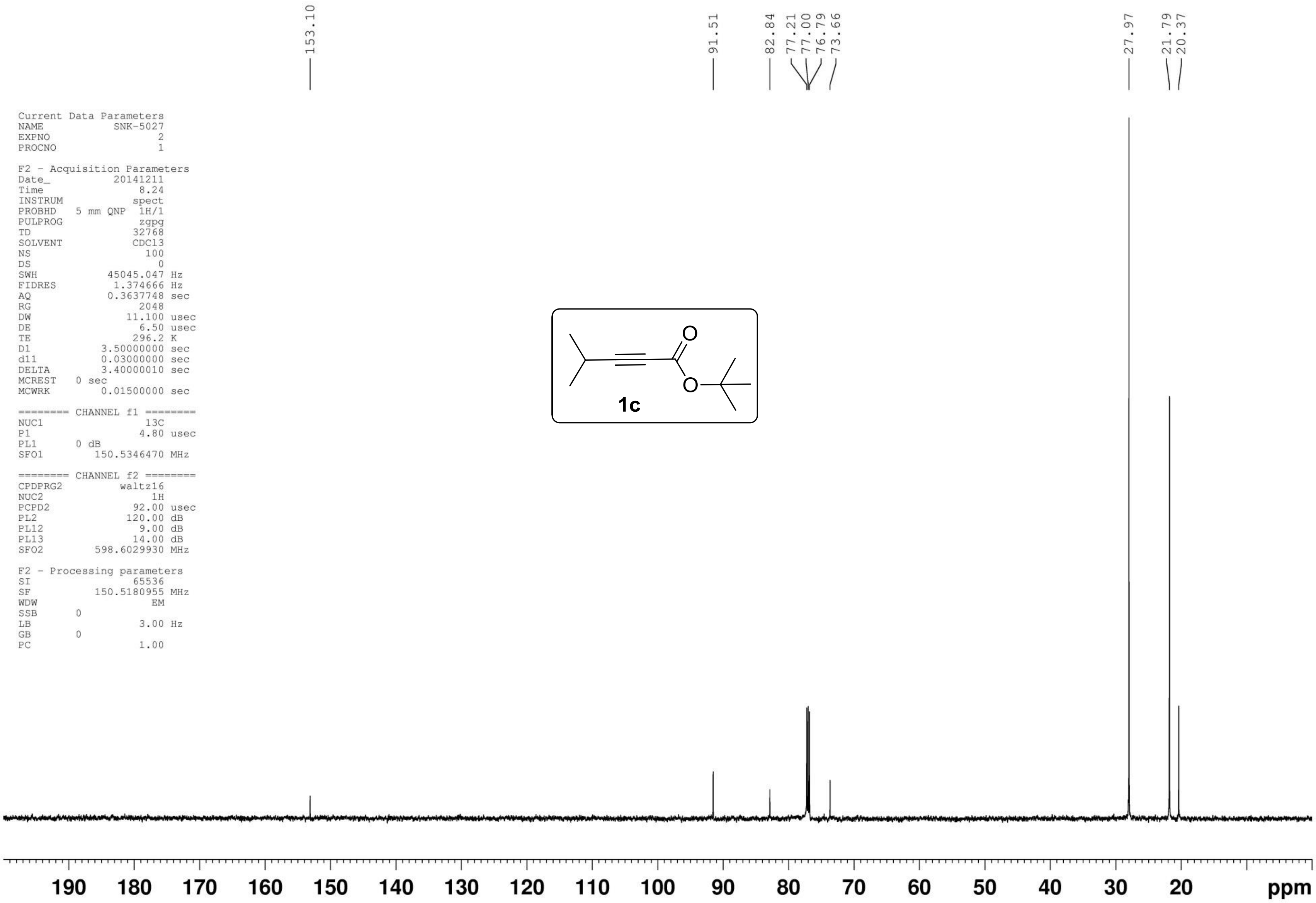

```
Current Data Parameters
NAME            SNK-5020
EXPNO                  1
PROCNO                 1

F2 - Acquisition Parameters
Date_           20141205
Time                7.35
INSTRUM            spect
PROBHD   5 mm QNP  1H/1
PULPROG               zg
TD                 33556
SOLVENT            CDCl3
NS                    16
DS                     0
SWH            12019.230 Hz
FIDRES          0.358184 Hz
AQ             1.3959796 sec
RG                    64
DW                41.600 usec
DE                  6.50 usec
TE                 295.0 K
D1            2.00000000 sec
MCREST   0 sec
MCWRK         0.01500000 sec

======== CHANNEL f1 ========
NUC1                  1H
P1                 10.00 usec
PL1      0 dB
SFO1         598.6035916 MHz

F2 - Processing parameters
SI                 32768
SF           598.6000309 MHz
WDW                   no
SSB      0
LB       0 Hz
GB       0
PC                  0.10
```

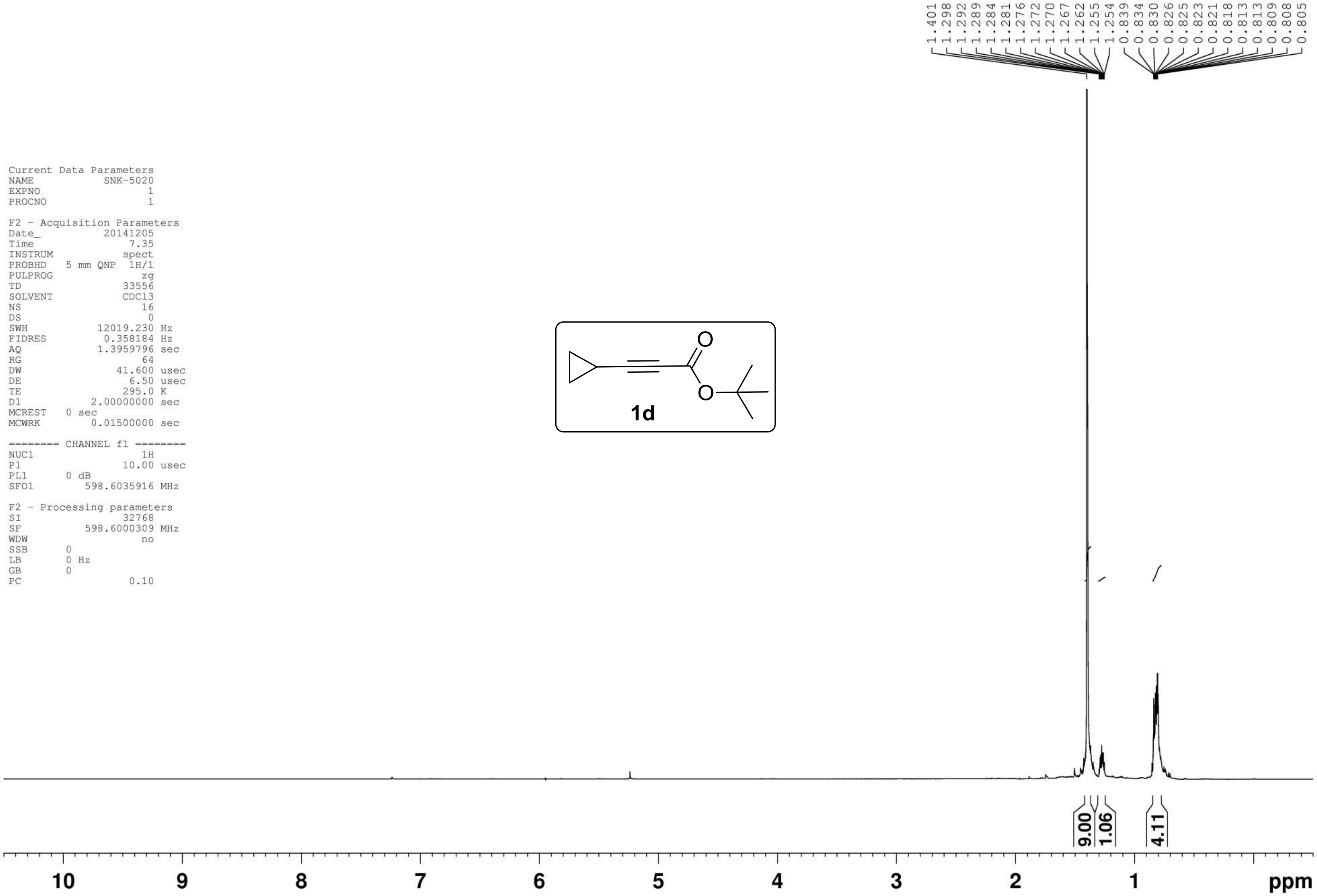

```
Current Data Parameters
NAME            SNK-5020
EXPNO                  2
PROCNO                 1

F2 - Acquisition Parameters
Date_           20141205
Time                7.42
INSTRUM            spect
PROBHD   5 mm QNP  1H/1
PULPROG             zgpg
TD                 32768
SOLVENT            CDCl3
NS                   100
DS                     0
SWH            45045.047 Hz
FIDRES          1.374666 Hz
AQ             0.3637748 sec
RG                  2048
DW                11.100 usec
DE                  6.50 usec
TE                 296.4 K
D1            3.50000000 sec
d11           0.03000000 sec
DELTA         3.40000010 sec
MCREST   0 sec
MCWRK         0.01500000 sec

======== CHANNEL f1 ========
NUC1                 13C
P1                  4.80 usec
PL1      0 dB
SFO1         150.5346470 MHz

======== CHANNEL f2 ========
CPDPRG2          waltz16
NUC2                  1H
PCPD2              92.00 usec
PL2               120.00 dB
PL12                9.00 dB
PL13               14.00 dB
SFO2         598.6029930 MHz

F2 - Processing parameters
SI                 65536
SF           150.5181033 MHz
WDW                   EM
SSB      0
LB                  3.00 Hz
GB       0
PC                  1.00
```

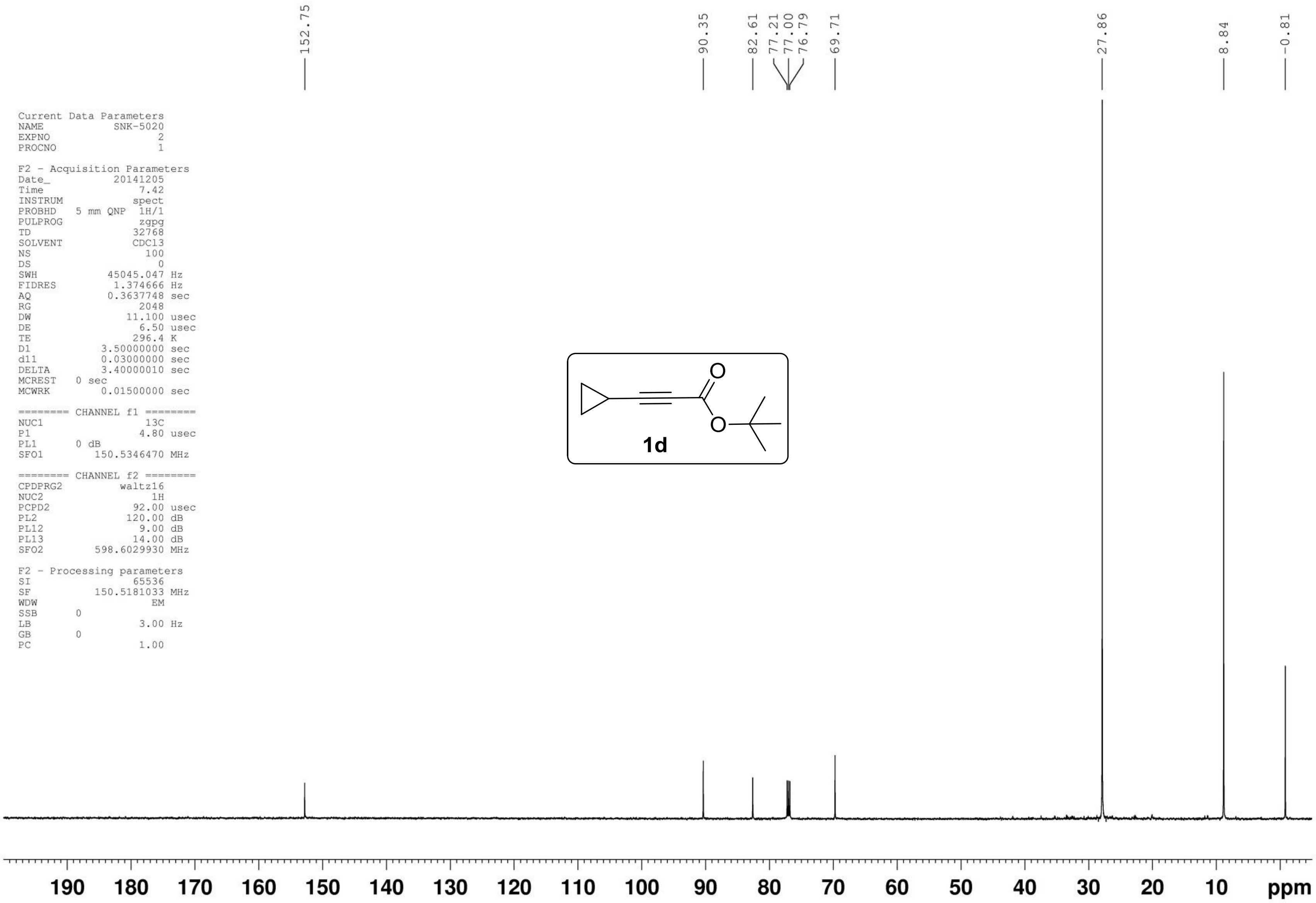

1d

2.435
2.430
2.420
2.414
2.408
2.398
1.781
1.780
1.772
1.759
1.669
1.662
1.656
1.652
1.645
1.642
1.482
1.466
1.464
1.463
1.462
1.461
1.460
1.459
1.458
1.456
1.453
1.450
1.444
1.441
1.438
1.436
1.429
1.413
1.413
1.402

Current Data Parameters
NAME SNK-5023
EXPNO 1
PROCNO 1

F2 - Acquisition Parameters
Date_ 20141206
Time 12.18
INSTRUM spect
PROBHD 5 mm QNP 1H/1
PULPROG zg
TD 33556
SOLVENT CDCl3
NS 16
DS 0
SWH 8389.262 Hz
FIDRES 0.250008 Hz
AQ 1.9999876 sec
RG 32
DW 59.600 usec
DE 6.50 usec
TE 294.7 K
D1 2.00000000 sec
MCREST 0 sec
MCWRK 0.01500000 sec

======== CHANNEL f1 ========
NUC1 1H
P1 10.00 usec
PL1 0 dB
SFO1 598.6029930 MHz

F2 - Processing parameters
SI 32768
SF 598.6000291 MHz
WDW no
SSB 0
LB 0 Hz
GB 0
PC 0.10

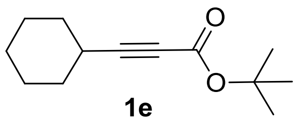

1e

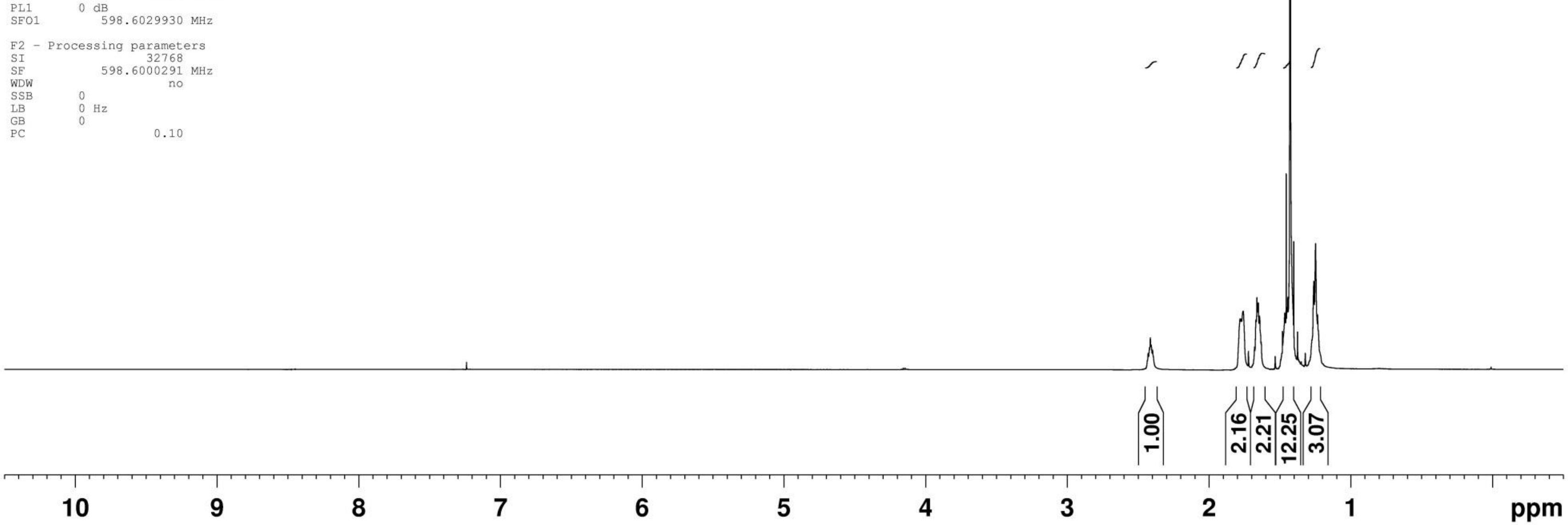

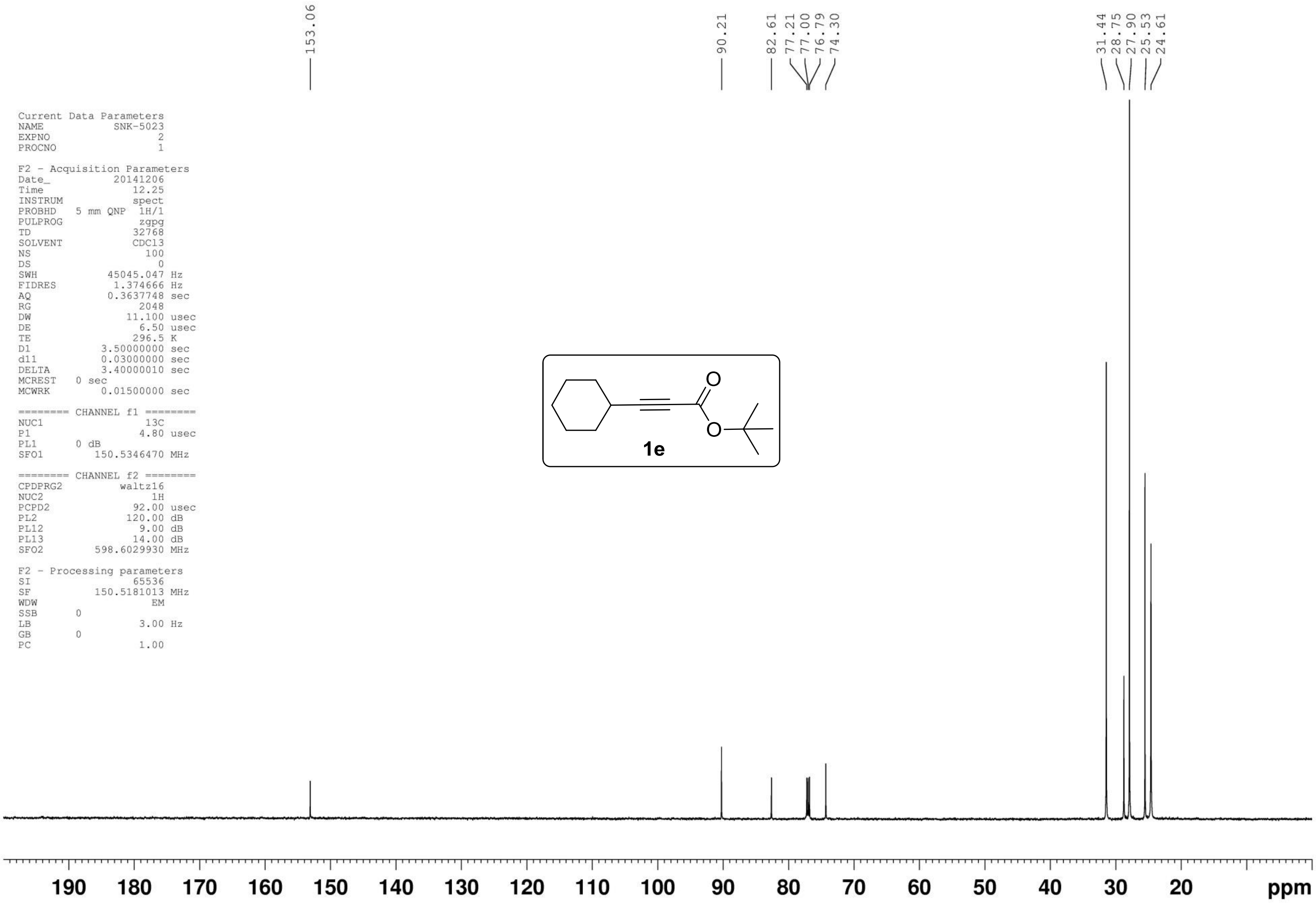
153.06
90.21
82.61
77.21
77.00
76.79
74.30
31.44
28.75
27.90
25.53
24.61
Current Data Parameters
NAME SNK-5023
EXPNO 2
PROCNO 1
F2 - Acquisition Parameters
Date_ 20141206
Time 12.25
INSTRUM spect
PROBHD 5 mm QNP 1H/1
PULPROG zgpg
TD 32768
SOLVENT CDCl3
NS 100
DS 0
SWH 45045.047 Hz
FIDRES 1.374666 Hz
AQ 0.3637748 sec
RG 2048
DW 11.100 usec
DE 6.50 usec
TE 296.5 K
D1 3.50000000 sec
d11 0.03000000 sec
DELTA 3.40000010 sec
MCREST 0 sec
MCWRK 0.01500000 sec
======== CHANNEL f1 ========
NUC1 13C
P1 4.80 usec
PL1 0 dB
SFO1 150.5346470 MHz
======== CHANNEL f2 ========
CPDPRG2 waltz16
NUC2 1H
PCPD2 92.00 usec
PL2 120.00 dB
PL12 9.00 dB
PL13 14.00 dB
SFO2 598.6029930 MHz
F2 - Processing parameters
SI 65536
SF 150.5181013 MHz
WDW EM
SSB 0
LB 3.00 Hz
GB 0
PC 1.00
O
O
1e
190 180 170 160 150 140 130 120 110 100 90 80 70 60 50 40 30 20 ppm

Current Data Parameters
NAME SNK-5031
EXPNO 1
PROCNO 1

F2 - Acquisition Parameters
Date_ 20141215
Time 10.57
INSTRUM spect
PROBHD 5 mm QNP 1H/1
PULPROG zg
TD 33556
SOLVENT CDCl3
NS 16
DS 0
SWH 8389.262 Hz
FIDRES 0.250008 Hz
AQ 1.9999876 sec
RG 64
DW 59.600 usec
DE 6.50 usec
TE 295.2 K
D1 2.00000000 sec
MCREST 0 sec
MCWRK 0.01500000 sec

======== CHANNEL f1 ========
NUC1 1H
P1 10.00 usec
PL1 0 dB
SFO1 598.6029930 MHz

F2 - Processing parameters
SI 32768
SF 598.6000301 MHz
WDW no
SSB 0
LB 0 Hz
GB 0
PC 0.10

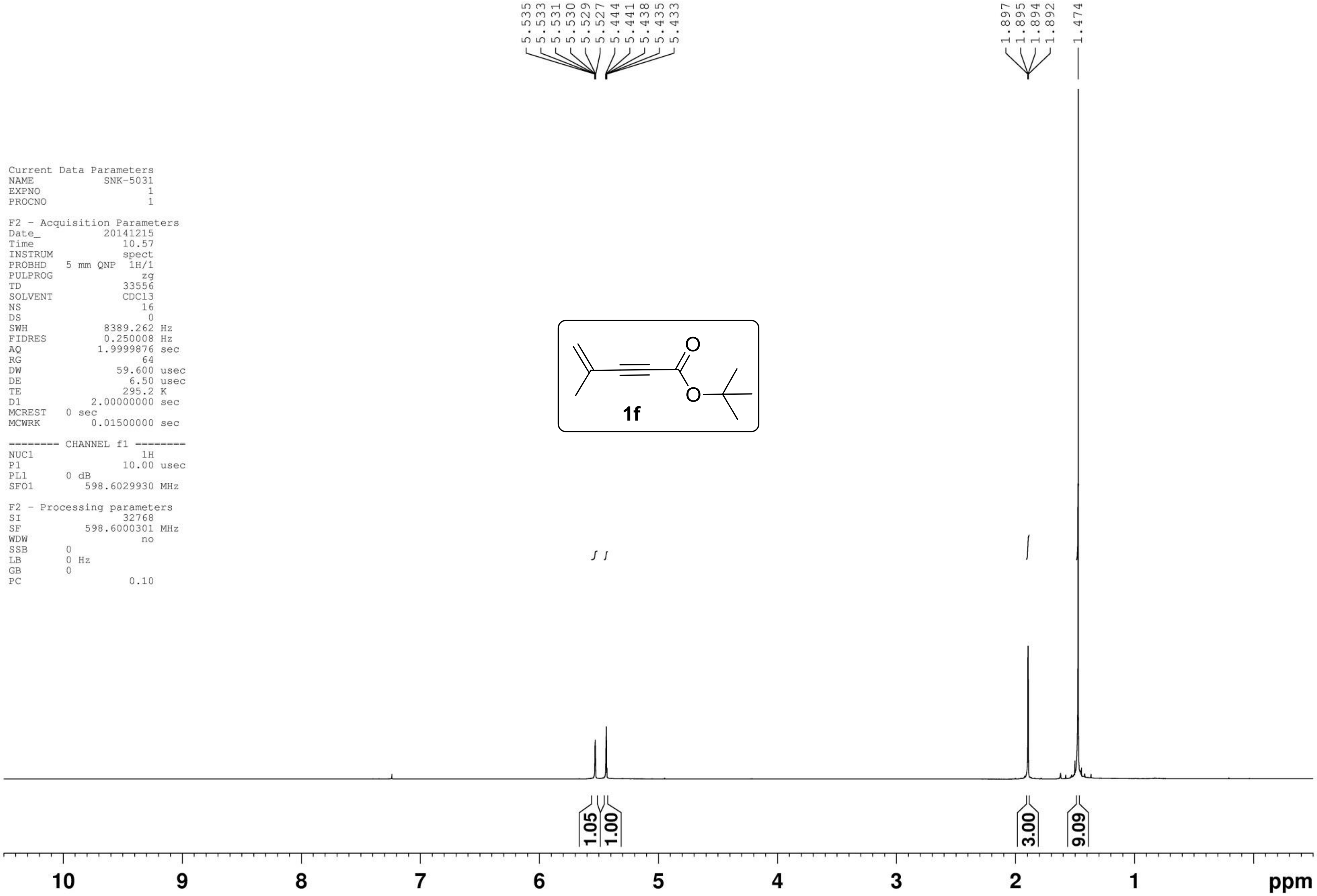

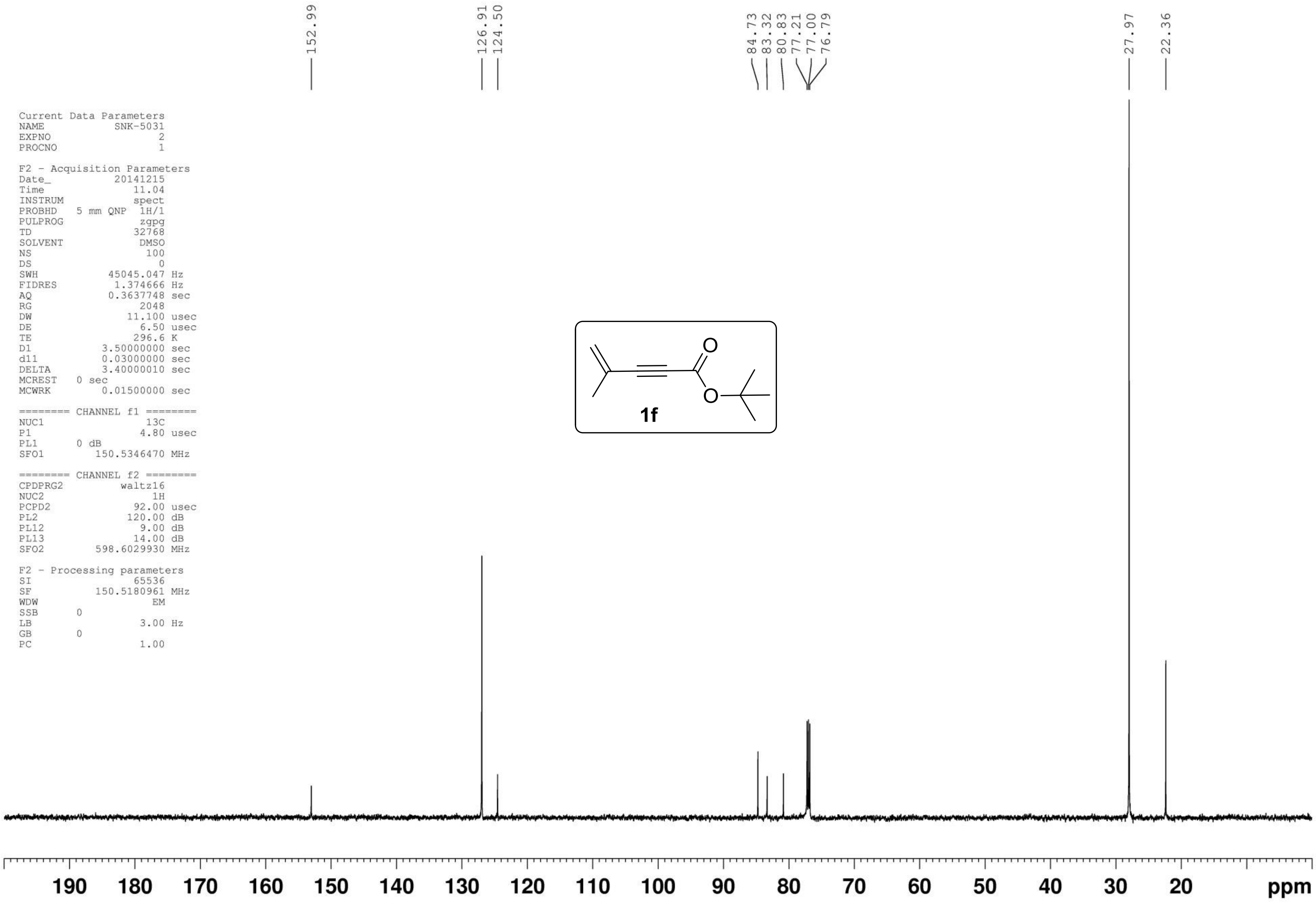
152.99
126.91
124.50
84.73
83.32
80.83
77.21
77.00
76.79
27.97
22.36
Current Data Parameters
NAME SNK-5031
EXPNO 2
PROCNO 1
F2 - Acquisition Parameters
Date_ 20141215
Time 11.04
INSTRUM spect
PROBHD 5 mm QNP 1H/1
PULPROG zgpg
TD 32768
SOLVENT DMSO
NS 100
DS 0
SWH 45045.047 Hz
FIDRES 1.374666 Hz
AQ 0.3637748 sec
RG 2048
DW 11.100 usec
DE 6.50 usec
TE 296.6 K
D1 3.50000000 sec
d11 0.03000000 sec
DELTA 3.40000010 sec
MCREST 0 sec
MCWRK 0.01500000 sec
======== CHANNEL f1 ========
NUC1 13C
P1 4.80 usec
PL1 0 dB
SFO1 150.5346470 MHz
======== CHANNEL f2 ========
CPDPRG2 waltz16
NUC2 1H
PCPD2 92.00 usec
PL2 120.00 dB
PL12 9.00 dB
PL13 14.00 dB
SFO2 598.6029930 MHz
F2 - Processing parameters
SI 65536
SF 150.5180961 MHz
WDW EM
SSB 0
LB 3.00 Hz
GB 0
PC 1.00
O
O
1f
190 180 170 160 150 140 130 120 110 100 90 80 70 60 50 40 30 20 ppm

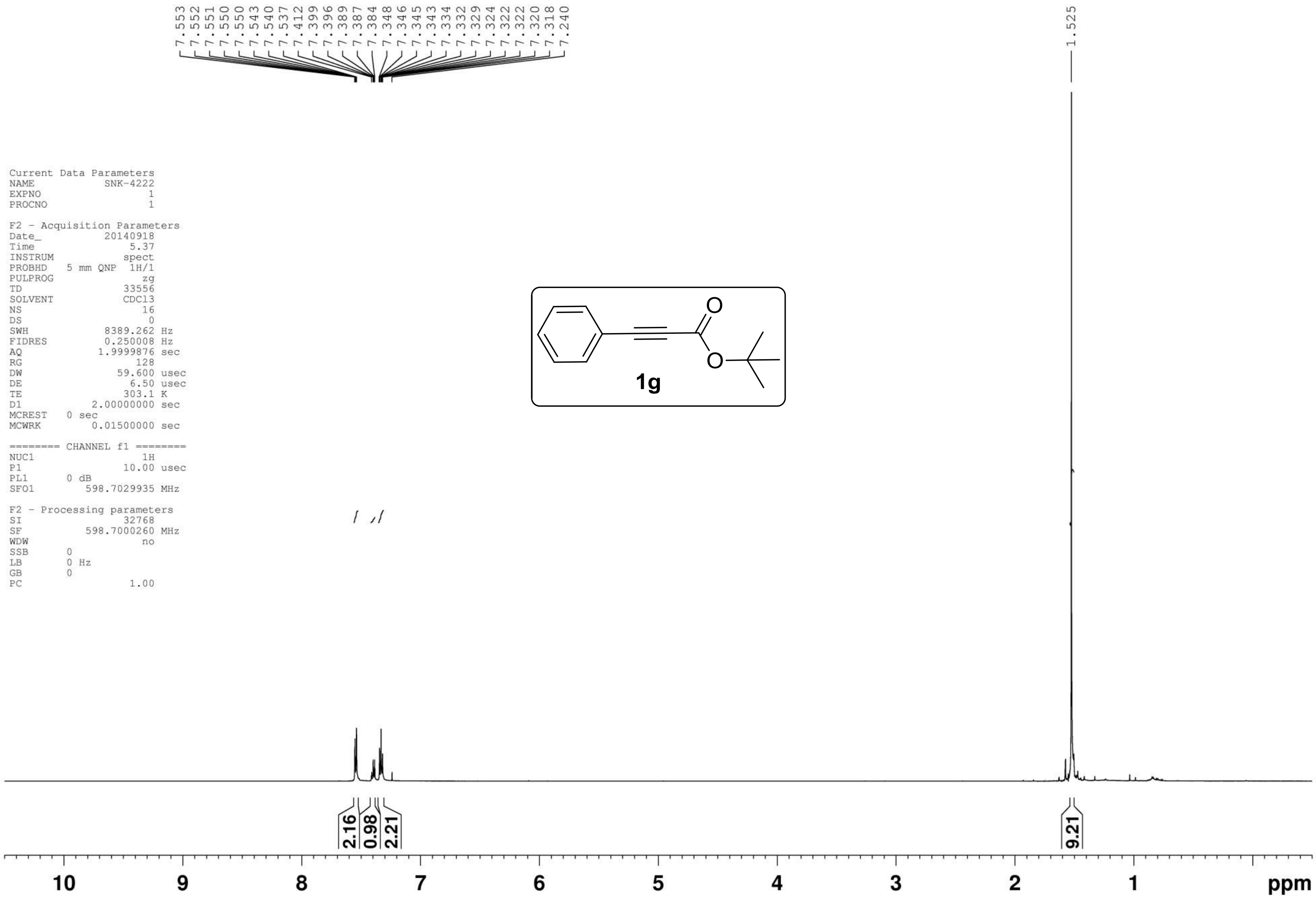

1g

```
Current Data Parameters
NAME             SNK-4222
EXPNO                   1
PROCNO                  1

F2 - Acquisition Parameters
Date_            20140918
Time                 5.37
INSTRUM             spect
PROBHD   5 mm QNP  1H/1
PULPROG                zg
TD                  33556
SOLVENT             CDCl3
NS                     16
DS                      0
SWH              8389.262 Hz
FIDRES           0.250008 Hz
AQ              1.9999876 sec
RG                    128
DW                 59.600 usec
DE                   6.50 usec
TE                  303.1 K
D1             2.00000000 sec
MCREST   0 sec
MCWRK          0.01500000 sec

======== CHANNEL f1 ========
NUC1                   1H
P1                  10.00 usec
PL1        0 dB
SFO1          598.7029935 MHz

F2 - Processing parameters
SI                  32768
SF            598.7000260 MHz
WDW                    no
SSB        0
LB         0 Hz
GB         0
PC                   1.00
```

```
Current Data Parameters
NAME            SNK-4222
EXPNO                  2
PROCNO                 1

F2 - Acquisition Parameters
Date_           20140918
Time                5.44
INSTRUM            spect
PROBHD   5 mm QNP  1H/1
PULPROG             zgpg
TD                 32768
SOLVENT            CDC13
NS                   100
DS                     0
SWH            45045.047 Hz
FIDRES          1.374666 Hz
AQ             0.3637748 sec
RG                  2048
DW                11.100 usec
DE                  6.50 usec
TE                 304.3 K
D1            3.50000000 sec
d11           0.03000000 sec
DELTA         3.40000010 sec
MCREST   0 sec
MCWRK         0.01500000 sec

======== CHANNEL f1 ========
NUC1                 13C
P1                  4.80 usec
PL1      0 dB
SFO1         150.5597948 MHz

======== CHANNEL f2 ========
CPDPRG2          waltz16
NUC2                  1H
PCPD2              92.00 usec
PL2               120.00 dB
PL12                9.00 dB
PL13               14.00 dB
SFO2         598.7029935 MHz

F2 - Processing parameters
SI                 65536
SF           150.5432369 MHz
WDW                   EM
SSB      0
LB                  3.00 Hz
GB       0
PC                  1.00
```

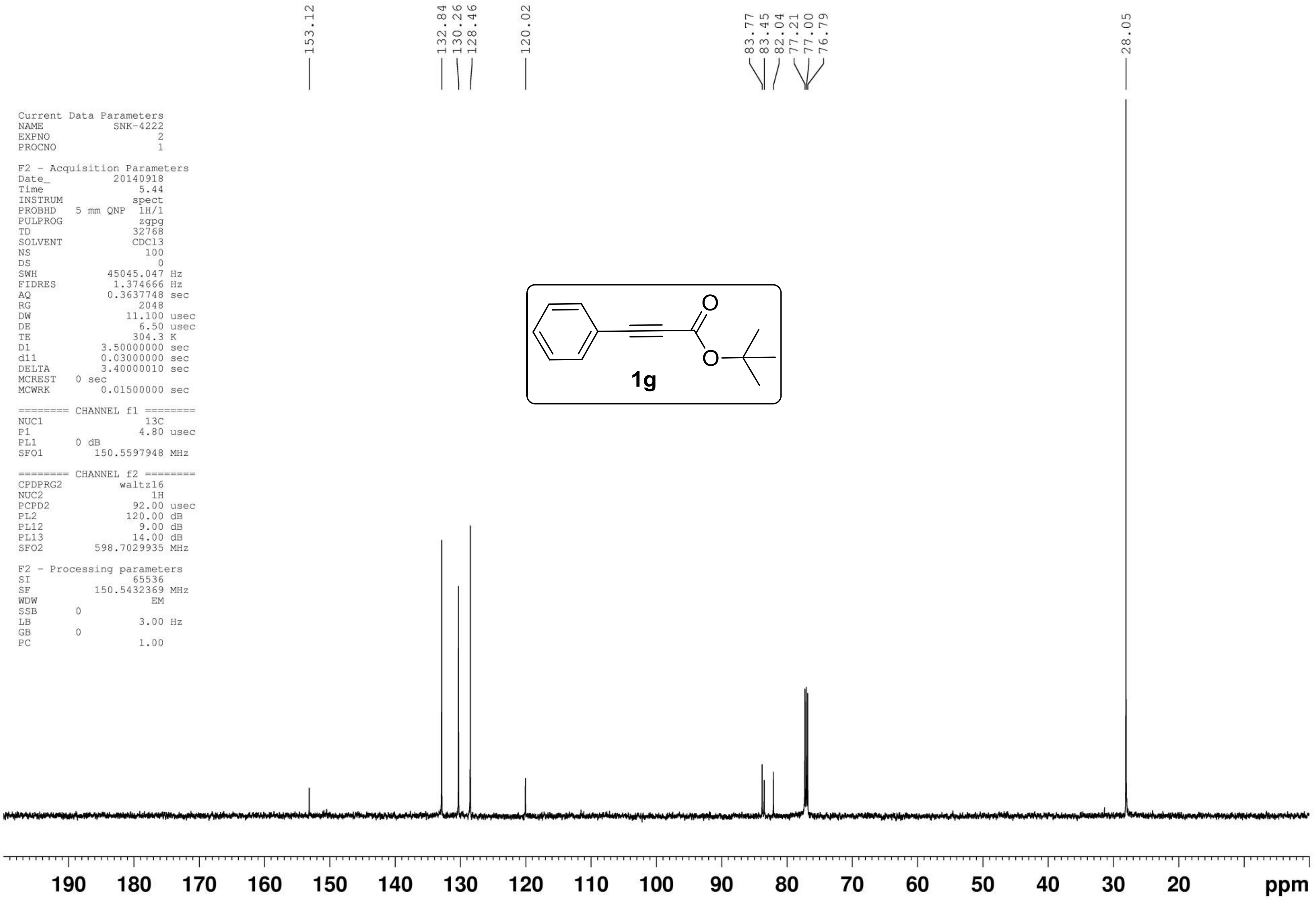

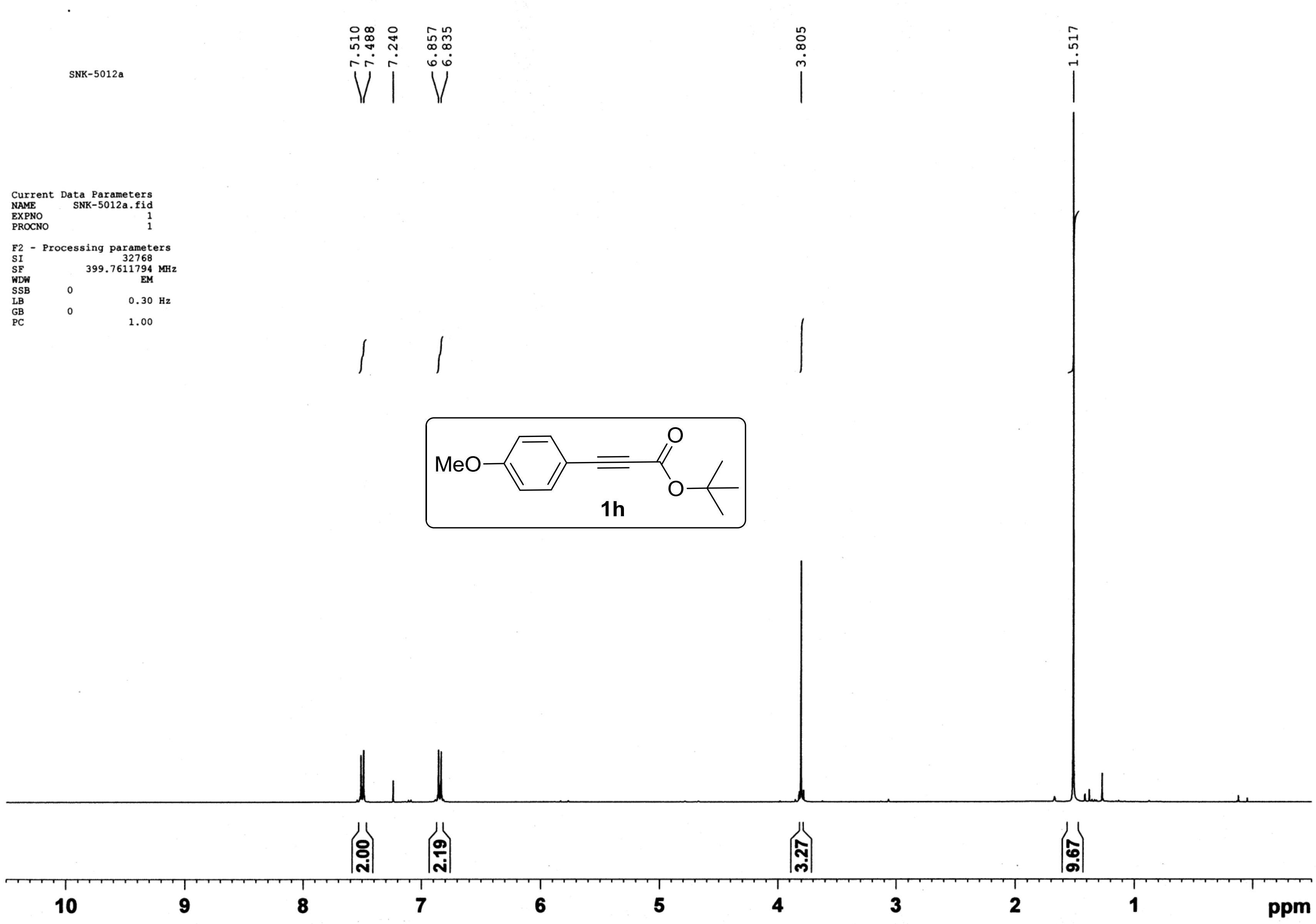
SNK-5012a
7.510
7.488
7.240
6.857
6.835
3.805
1.517
Current Data Parameters
NAME SNK-5012a.fid
EXPNO 1
PROCNO 1
F2 - Processing parameters
SI 32768
SF 399.7611794 MHz
WDW EM
SSB 0
LB 0.30 Hz
GB 0
PC 1.00
MeO
O
O
1h
2.00
2.19
3.27
9.67
10
9
8
7
6
5
4
3
2
1
ppm

```
Current Data Parameters
NAME      SNK-5012aC.fid
EXPNO                  1
PROCNO                 1

F2 - Processing parameters
SI                 65536
SF          100.5214650 MHz
WDW                   EM
SSB       0
LB                  0.30 Hz
GB        0
PC                  1.00
```

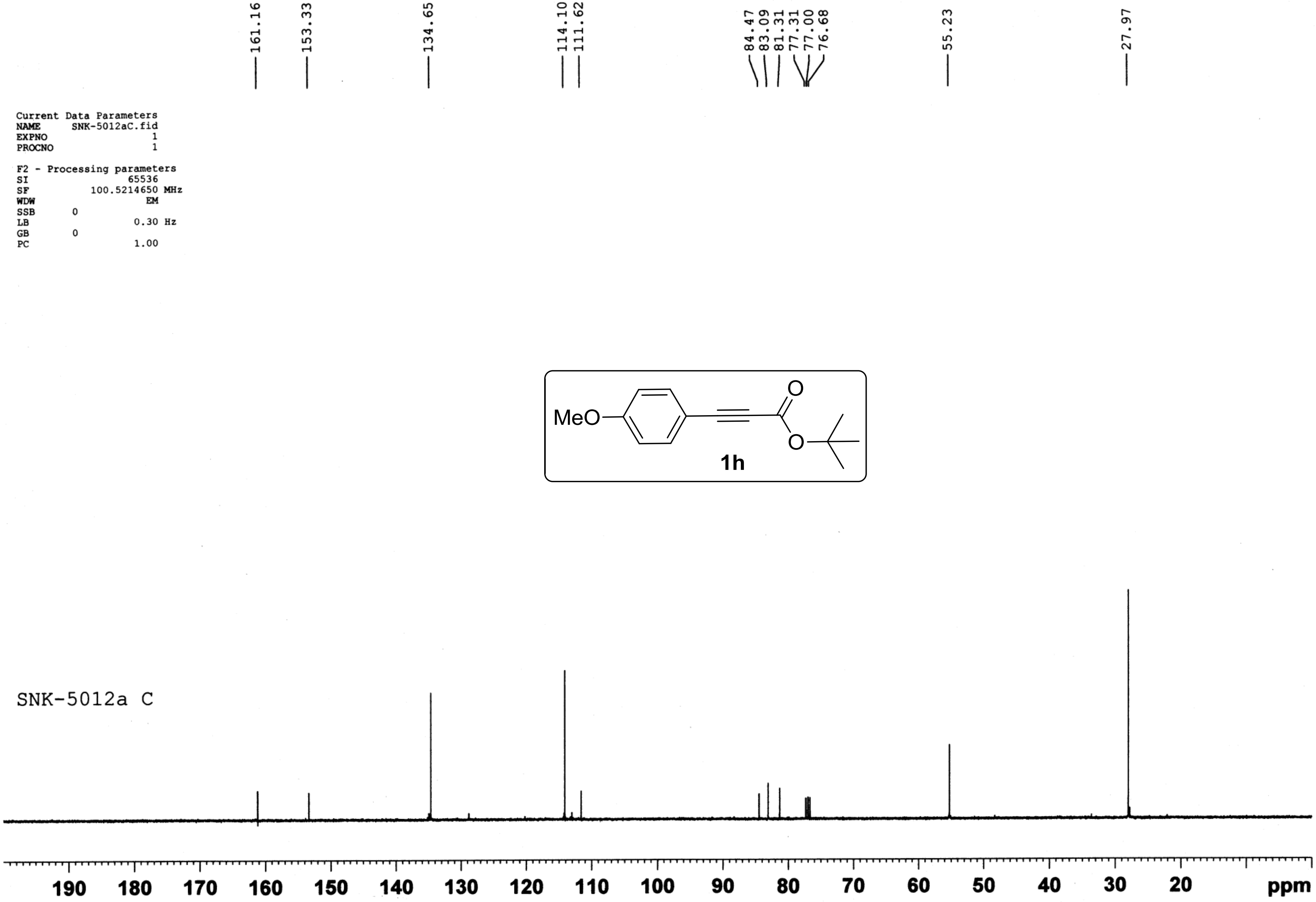

Current Data Parameters
NAME SNK-5015
EXPNO 1
PROCNO 1

F2 - Acquisition Parameters
Date_ 20141203
Time 7.06
INSTRUM spect
PROBHD 5 mm QNP 1H/1
PULPROG zg
TD 33556
SOLVENT CDCl3
NS 16
DS 0
SWH 8389.262 Hz
FIDRES 0.250008 Hz
AQ 1.9999876 sec
RG 32
DW 59.600 usec
DE 6.50 usec
TE 297.0 K
D1 2.00000000 sec
MCREST 0 sec
MCWRK 0.01500000 sec

======== CHANNEL f1 ========
NUC1 1H
P1 10.00 usec
PL1 0 dB
SFO1 598.6029930 MHz

F2 - Processing parameters
SI 32768
SF 598.6000283 MHz
WDW no
SSB 0
LB 0 Hz
GB 0
PC 0.10

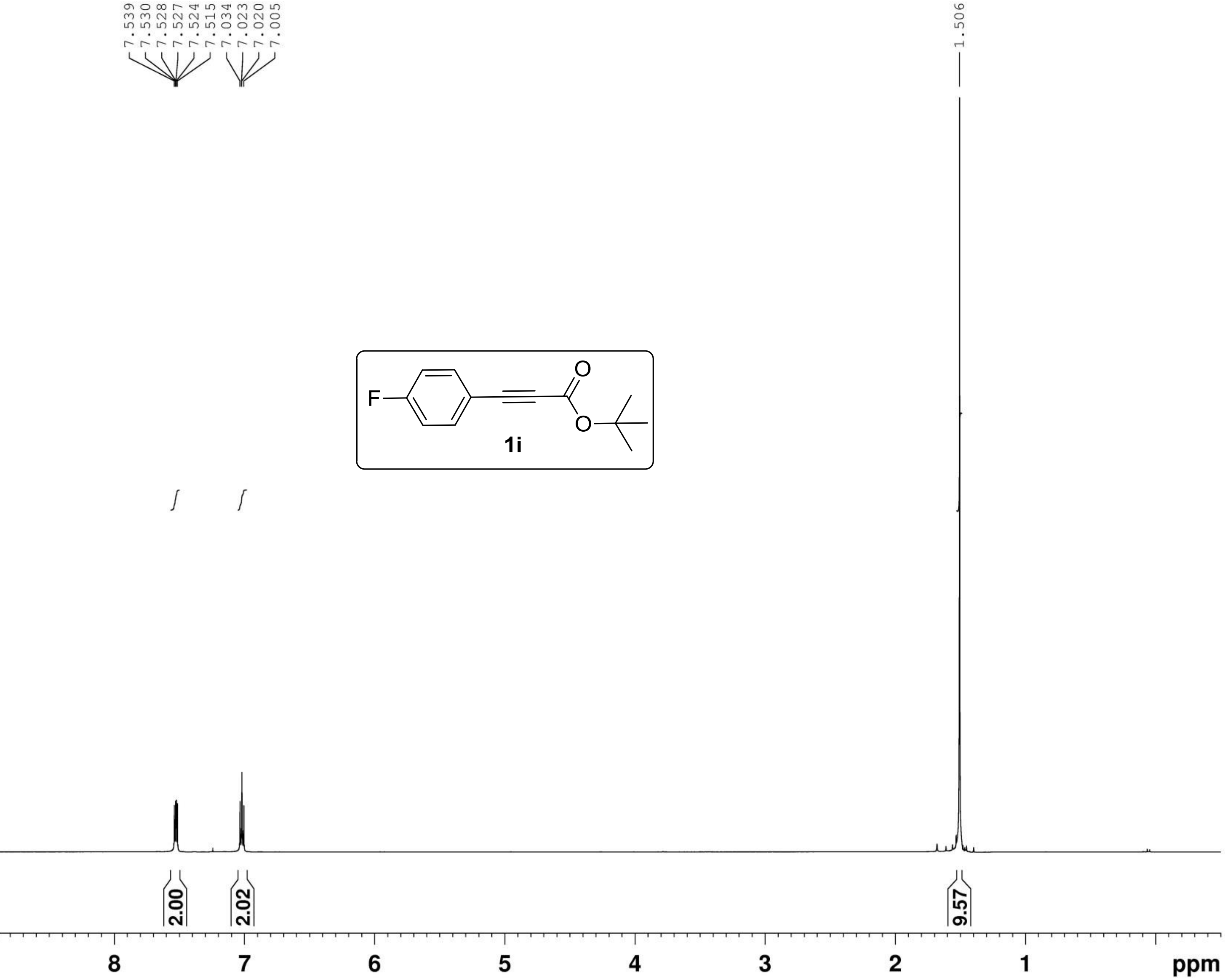

1i

```
Current Data Parameters
NAME            SNK-5015
EXPNO                  2
PROCNO                 1

F2 - Acquisition Parameters
Date_           20141203
Time                7.20
INSTRUM            spect
PROBHD   5 mm QNP  1H/1
PULPROG             zgpg
TD                 32768
SOLVENT            CDCl3
NS                   222
DS                     0
SWH            45045.047 Hz
FIDRES          1.374666 Hz
AQ             0.3637748 sec
RG                  2048
DW                11.100 usec
DE                  6.50 usec
TE                 297.7 K
D1            3.50000000 sec
d11           0.03000000 sec
DELTA         3.40000010 sec
MCREST   0 sec
MCWRK         0.01500000 sec

======== CHANNEL f1 ========
NUC1                 13C
P1                  4.80 usec
PL1      0 dB
SFO1         150.5346470 MHz

======== CHANNEL f2 ========
CPDPRG2          waltz16
NUC2                  1H
PCPD2              92.00 usec
PL2               120.00 dB
PL12                9.00 dB
PL13               14.00 dB
SFO2         598.6029930 MHz

F2 - Processing parameters
SI                 65536
SF           150.5180987 MHz
WDW                   EM
SSB      0
LB                  3.00 Hz
GB       0
PC                  1.00
```

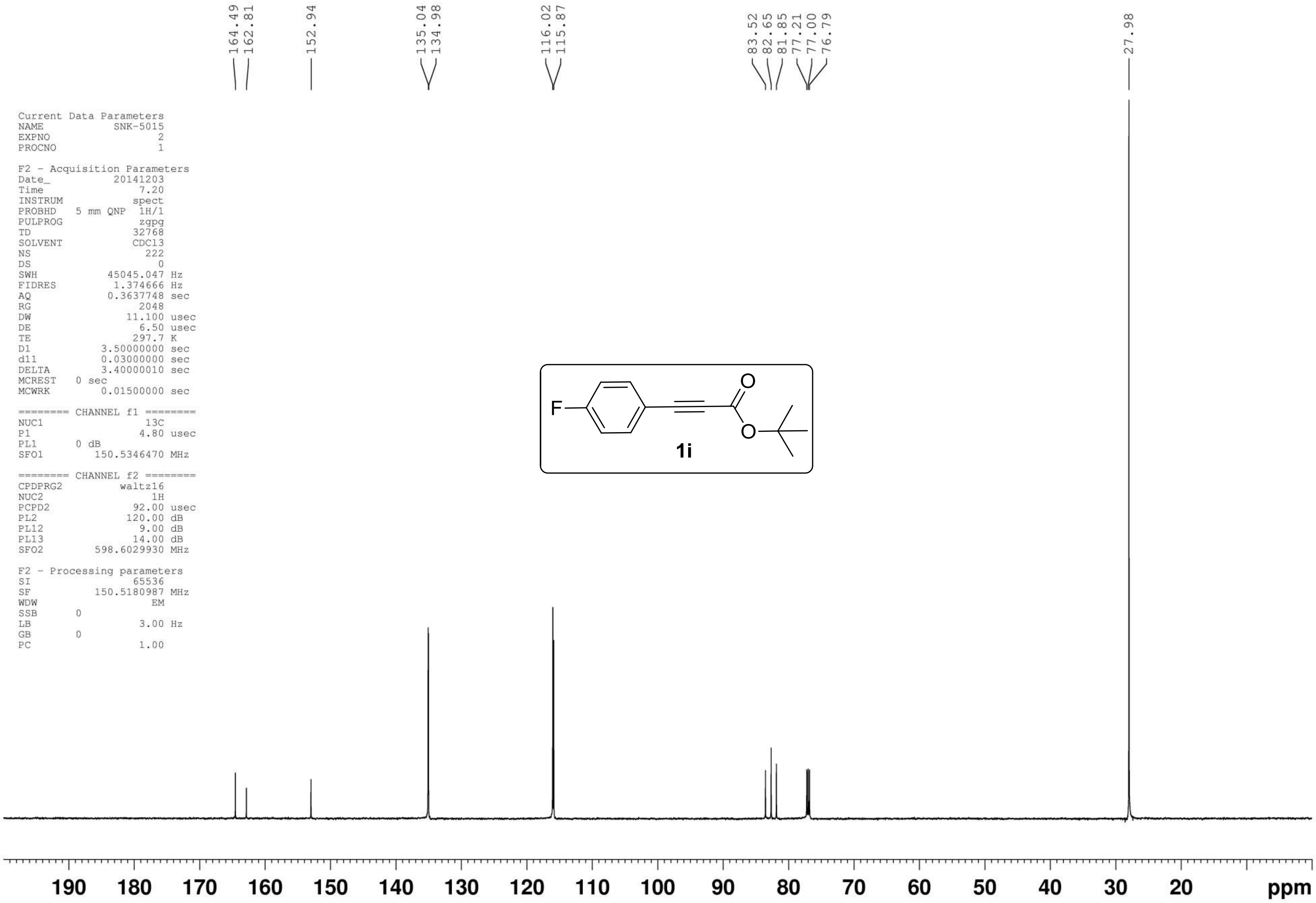

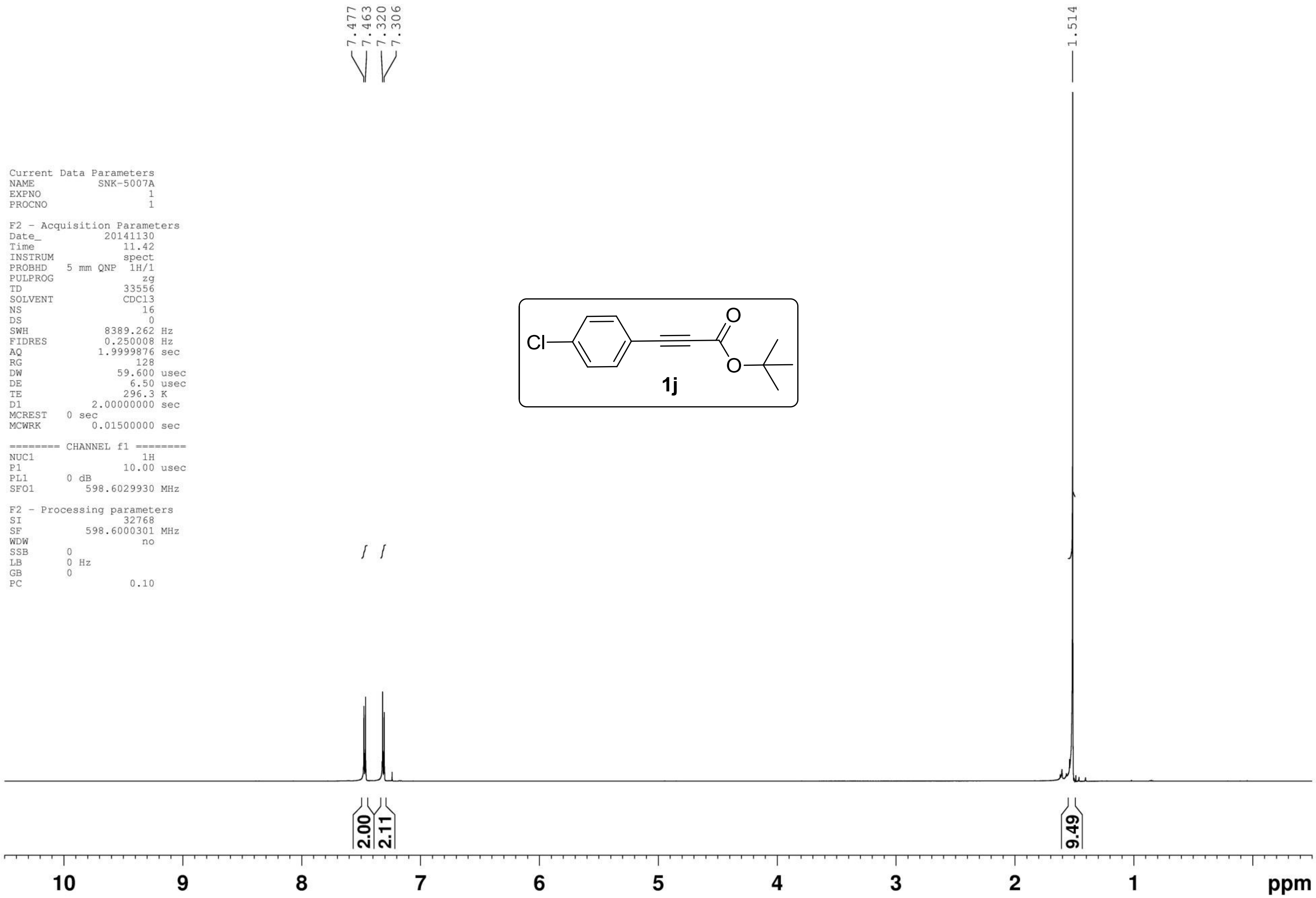
7.477
7.463
7.320
7.306
1.514
Current Data Parameters
NAME SNK-5007A
EXPNO 1
PROCNO 1
F2 - Acquisition Parameters
Date_ 20141130
Time 11.42
INSTRUM spect
PROBHD 5 mm QNP 1H/1
PULPROG zg
TD 33556
SOLVENT CDCl3
NS 16
DS 0
SWH 8389.262 Hz
FIDRES 0.250008 Hz
AQ 1.9999876 sec
RG 128
DW 59.600 usec
DE 6.50 usec
TE 296.3 K
D1 2.00000000 sec
MCREST 0 sec
MCWRK 0.01500000 sec
======== CHANNEL f1 ========
NUC1 1H
P1 10.00 usec
PL1 0 dB
SFO1 598.6029930 MHz
F2 - Processing parameters
SI 32768
SF 598.6000301 MHz
WDW no
SSB 0
LB 0 Hz
GB 0
PC 0.10
Cl
O
O
1j
2.00
2.11
9.49
10
9
8
7
6
5
4
3
2
1
ppm

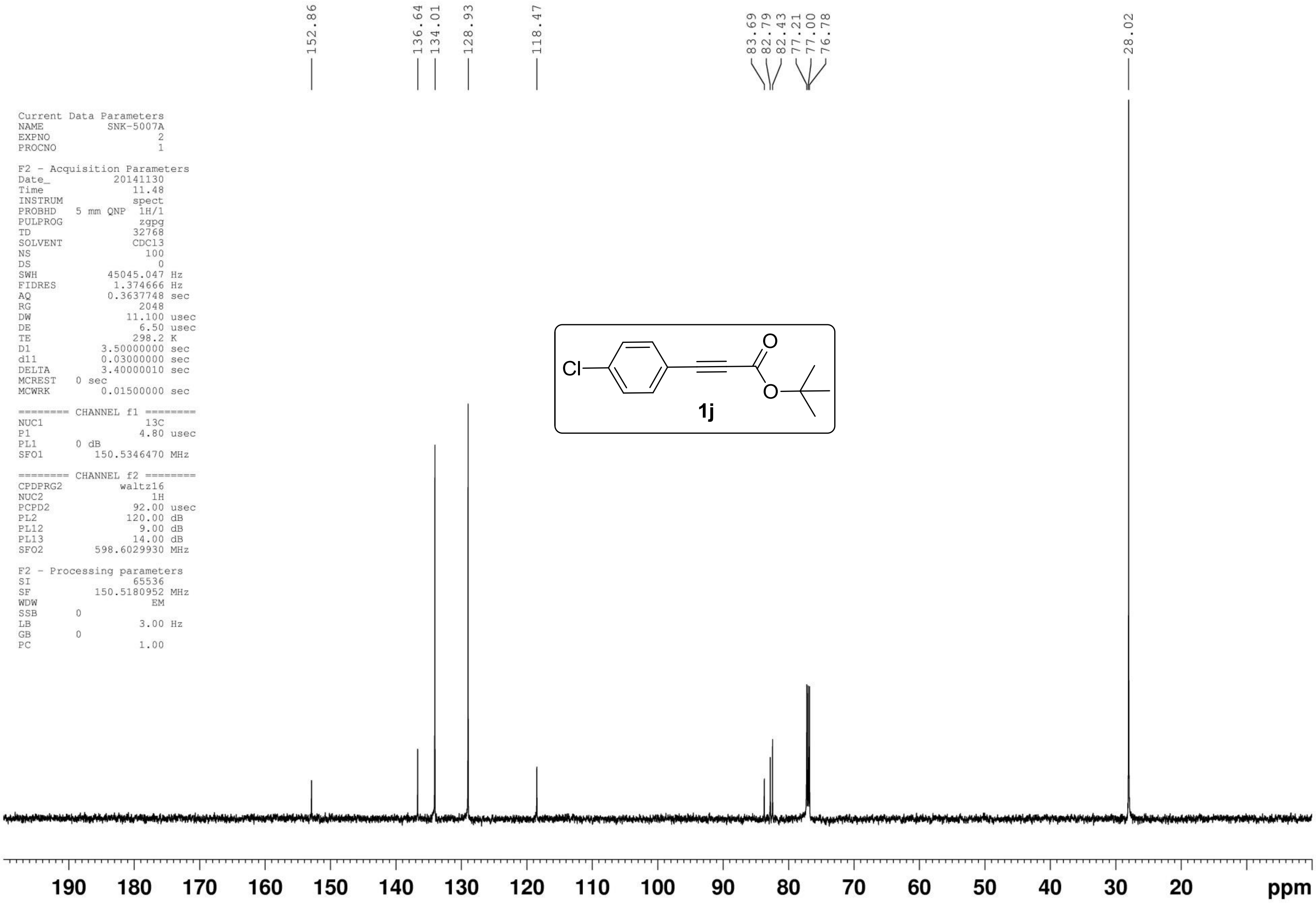
152.86
136.64
134.01
128.93
118.47
83.69
82.79
82.43
77.21
77.00
76.78
28.02
Current Data Parameters
NAME SNK-5007A
EXPNO 2
PROCNO 1
F2 - Acquisition Parameters
Date_ 20141130
Time 11.48
INSTRUM spect
PROBHD 5 mm QNP 1H/1
PULPROG zgpg
TD 32768
SOLVENT CDCl3
NS 100
DS 0
SWH 45045.047 Hz
FIDRES 1.374666 Hz
AQ 0.3637748 sec
RG 2048
DW 11.100 usec
DE 6.50 usec
TE 298.2 K
D1 3.50000000 sec
d11 0.03000000 sec
DELTA 3.40000010 sec
MCREST 0 sec
MCWRK 0.01500000 sec
======== CHANNEL f1 ========
NUC1 13C
P1 4.80 usec
PL1 0 dB
SFO1 150.5346470 MHz
======== CHANNEL f2 ========
CPDPRG2 waltz16
NUC2 1H
PCPD2 92.00 usec
PL2 120.00 dB
PL12 9.00 dB
PL13 14.00 dB
SFO2 598.6029930 MHz
F2 - Processing parameters
SI 65536
SF 150.5180952 MHz
WDW EM
SSB 0
LB 3.00 Hz
GB 0
PC 1.00
Cl
O
O
1j
190 180 170 160 150 140 130 120 110 100 90 80 70 60 50 40 30 20 ppm

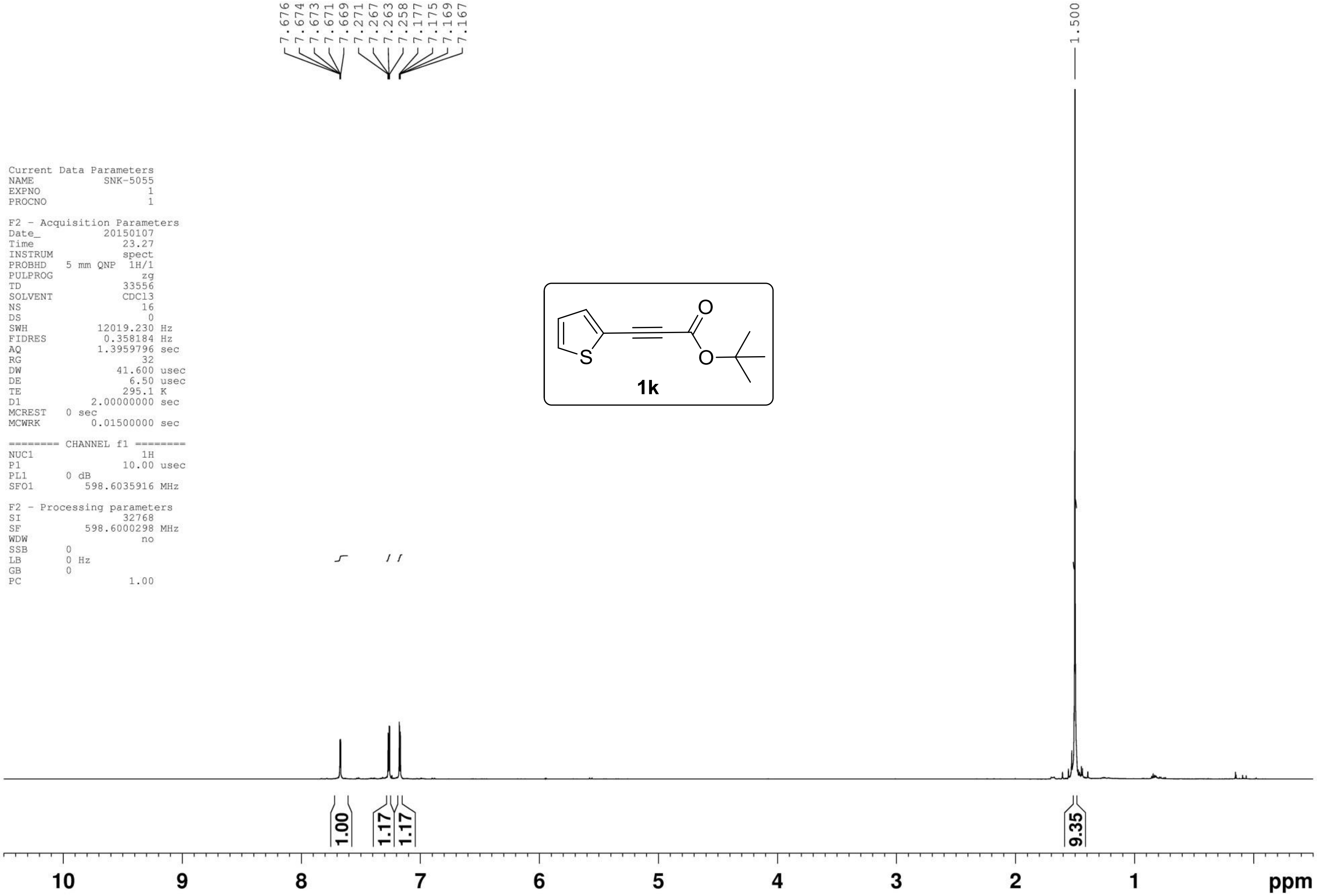

1k

```
Current Data Parameters
NAME             SNK-5055
EXPNO                   1
PROCNO                  1

F2 - Acquisition Parameters
Date_            20150107
Time                23.27
INSTRUM             spect
PROBHD   5 mm QNP  1H/1
PULPROG                zg
TD                  33556
SOLVENT             CDCl3
NS                     16
DS                      0
SWH             12019.230 Hz
FIDRES           0.358184 Hz
AQ              1.3959796 sec
RG                     32
DW                 41.600 usec
DE                   6.50 usec
TE                  295.1 K
D1             2.00000000 sec
MCREST   0 sec
MCWRK          0.01500000 sec

======== CHANNEL f1 ========
NUC1                   1H
P1                  10.00 usec
PL1      0 dB
SFO1          598.6035916 MHz

F2 - Processing parameters
SI                  32768
SF            598.6000298 MHz
WDW                    no
SSB      0
LB       0 Hz
GB       0
PC                   1.00
```

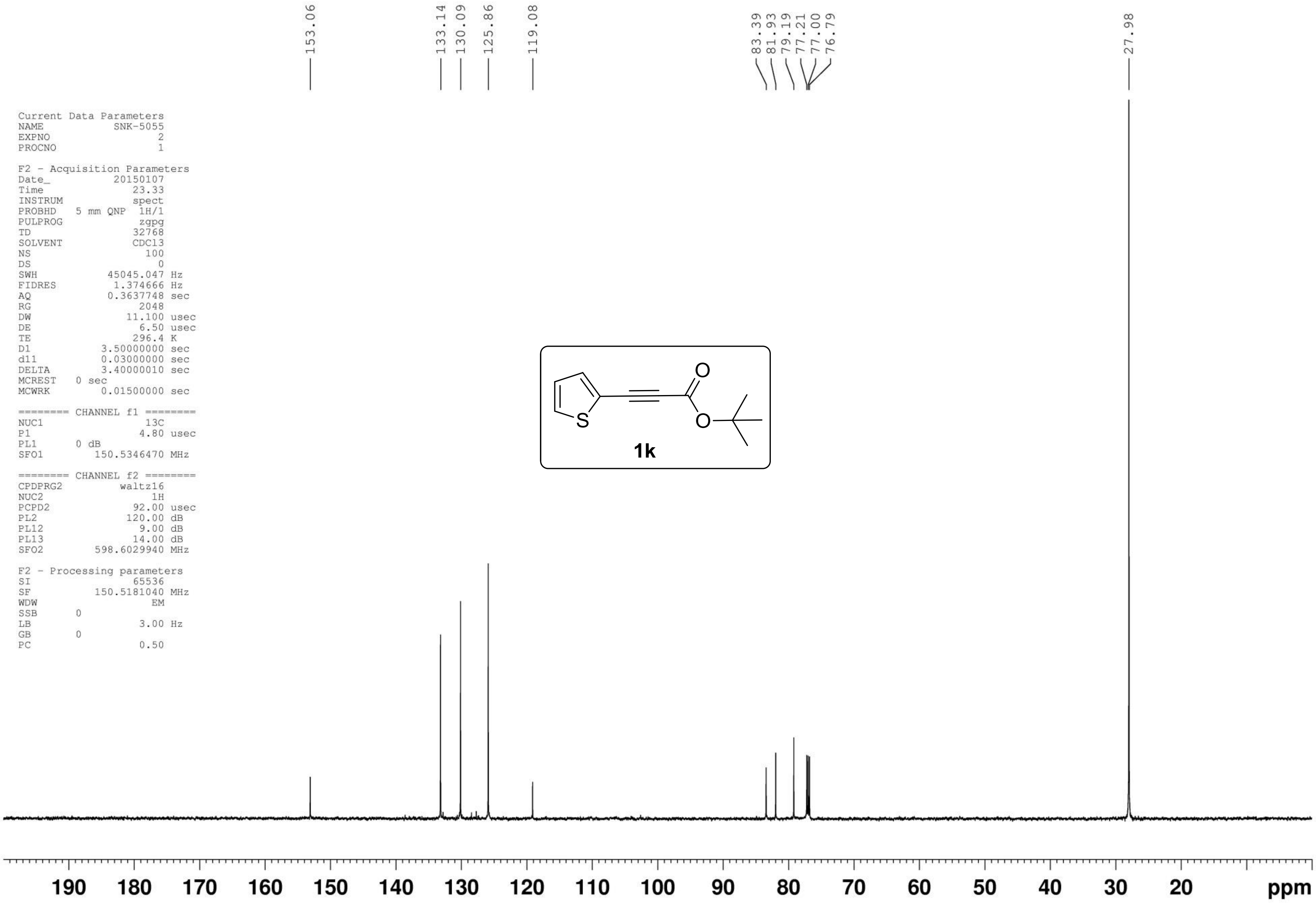


```
Current Data Parameters
NAME            SNK-5055
EXPNO                  2
PROCNO                 1

F2 - Acquisition Parameters
Date_           20150107
Time               23.33
INSTRUM            spect
PROBHD   5 mm QNP  1H/1
PULPROG             zgpg
TD                 32768
SOLVENT            CDCl3
NS                   100
DS                     0
SWH            45045.047 Hz
FIDRES          1.374666 Hz
AQ             0.3637748 sec
RG                  2048
DW                11.100 usec
DE                  6.50 usec
TE                 296.4 K
D1            3.50000000 sec
d11           0.03000000 sec
DELTA         3.40000010 sec
MCREST   0 sec
MCWRK         0.01500000 sec

======== CHANNEL f1 ========
NUC1                 13C
P1                  4.80 usec
PL1      0 dB
SFO1         150.5346470 MHz

======== CHANNEL f2 ========
CPDPRG2          waltz16
NUC2                  1H
PCPD2              92.00 usec
PL2               120.00 dB
PL12                9.00 dB
PL13               14.00 dB
SFO2         598.6029940 MHz

F2 - Processing parameters
SI                 65536
SF           150.5181040 MHz
WDW                   EM
SSB      0
LB                  3.00 Hz
GB       0
PC                  0.50
```

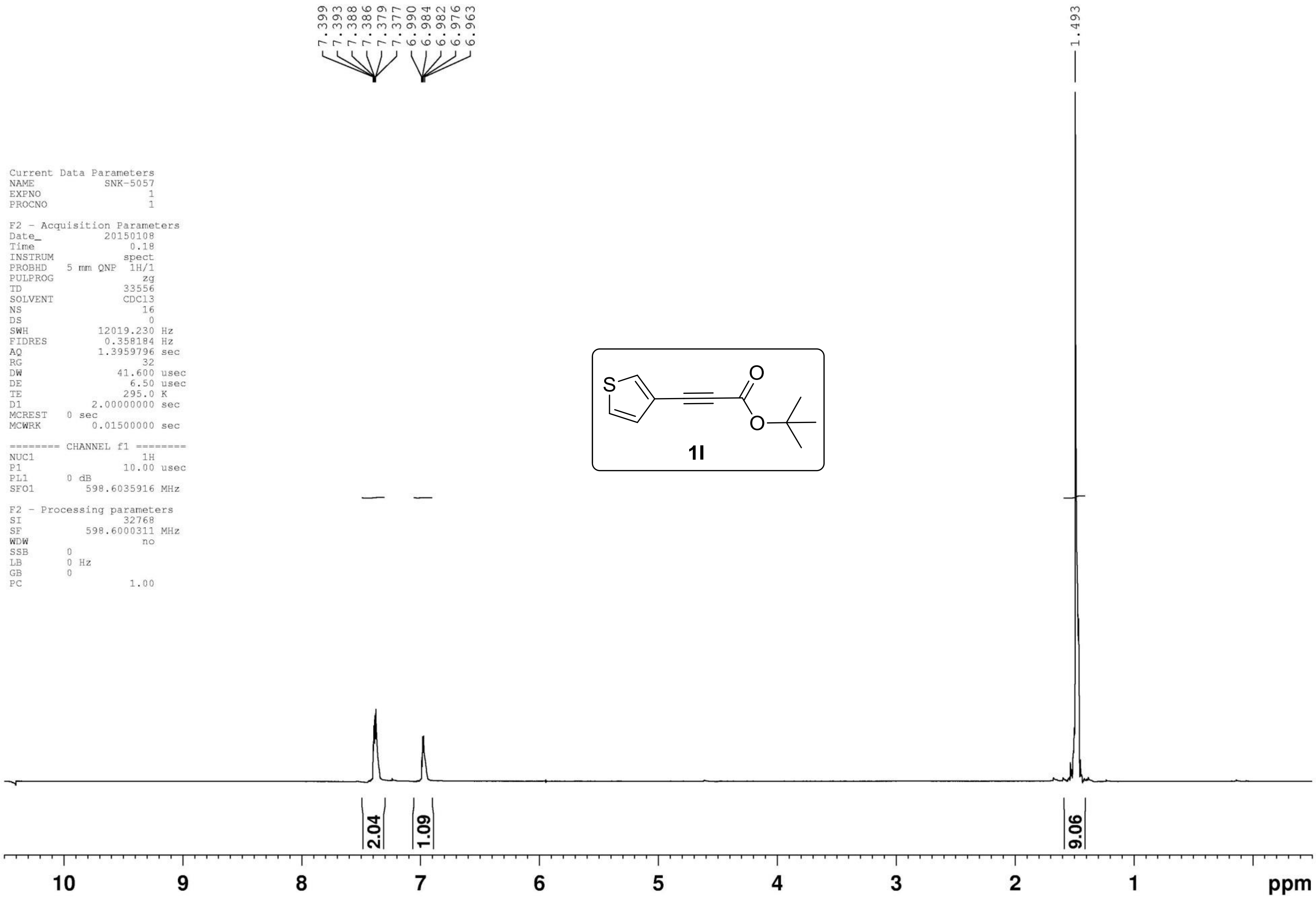
7.399
7.393
7.388
7.386
7.379
7.377
6.990
6.984
6.982
6.976
6.963
1.493
Current Data Parameters
NAME SNK-5057
EXPNO 1
PROCNO 1
F2 - Acquisition Parameters
Date_ 20150108
Time 0.18
INSTRUM spect
PROBHD 5 mm QNP 1H/1
PULPROG zg
TD 33556
SOLVENT CDCl3
NS 16
DS 0
SWH 12019.230 Hz
FIDRES 0.358184 Hz
AQ 1.3959796 sec
RG 32
DW 41.600 usec
DE 6.50 usec
TE 295.0 K
D1 2.00000000 sec
MCREST 0 sec
MCWRK 0.01500000 sec
======== CHANNEL f1 ========
NUC1 1H
P1 10.00 usec
PL1 0 dB
SFO1 598.6035916 MHz
F2 - Processing parameters
SI 32768
SF 598.6000311 MHz
WDW no
SSB 0
LB 0 Hz
GB 0
PC 1.00
S
O
O
1l
2.04
1.09
9.06
10
9
8
7
6
5
4
3
2
1
ppm

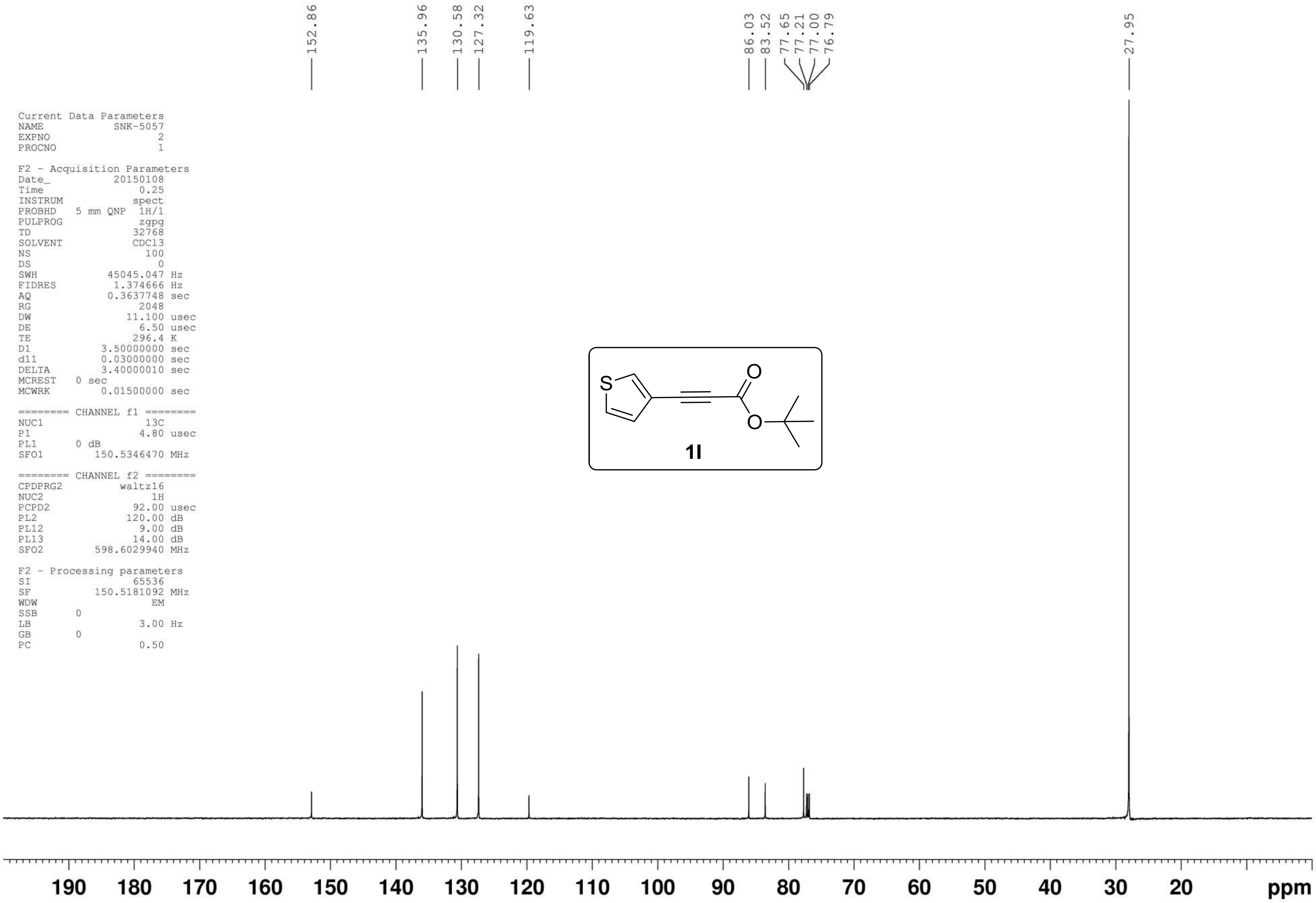
152.86
135.96
130.58
127.32
119.63
86.03
83.52
77.65
77.21
77.00
76.79
27.95
Current Data Parameters
NAME SNK-5057
EXPNO 2
PROCNO 1
F2 - Acquisition Parameters
Date_ 20150108
Time 0.25
INSTRUM spect
PROBHD 5 mm QNP 1H/1
PULPROG zgpg
TD 32768
SOLVENT CDCl3
NS 100
DS 0
SWH 45045.047 Hz
FIDRES 1.374666 Hz
AQ 0.3637748 sec
RG 2048
DW 11.100 usec
DE 6.50 usec
TE 296.4 K
D1 3.50000000 sec
d11 0.03000000 sec
DELTA 3.40000010 sec
MCREST 0 sec
MCWRK 0.01500000 sec
======== CHANNEL f1 ========
NUC1 13C
P1 4.80 usec
PL1 0 dB
SFO1 150.5346470 MHz
======== CHANNEL f2 ========
CPDPRG2 waltz16
NUC2 1H
PCPD2 92.00 usec
PL2 120.00 dB
PL12 9.00 dB
PL13 14.00 dB
SFO2 598.6029940 MHz
F2 - Processing parameters
SI 65536
SF 150.5181092 MHz
WDW EM
SSB 0
LB 3.00 Hz
GB 0
PC 0.50
S
O
O
1l
190
180
170
160
150
140
130
120
110
100
90
80
70
60
50
40
30
20
ppm

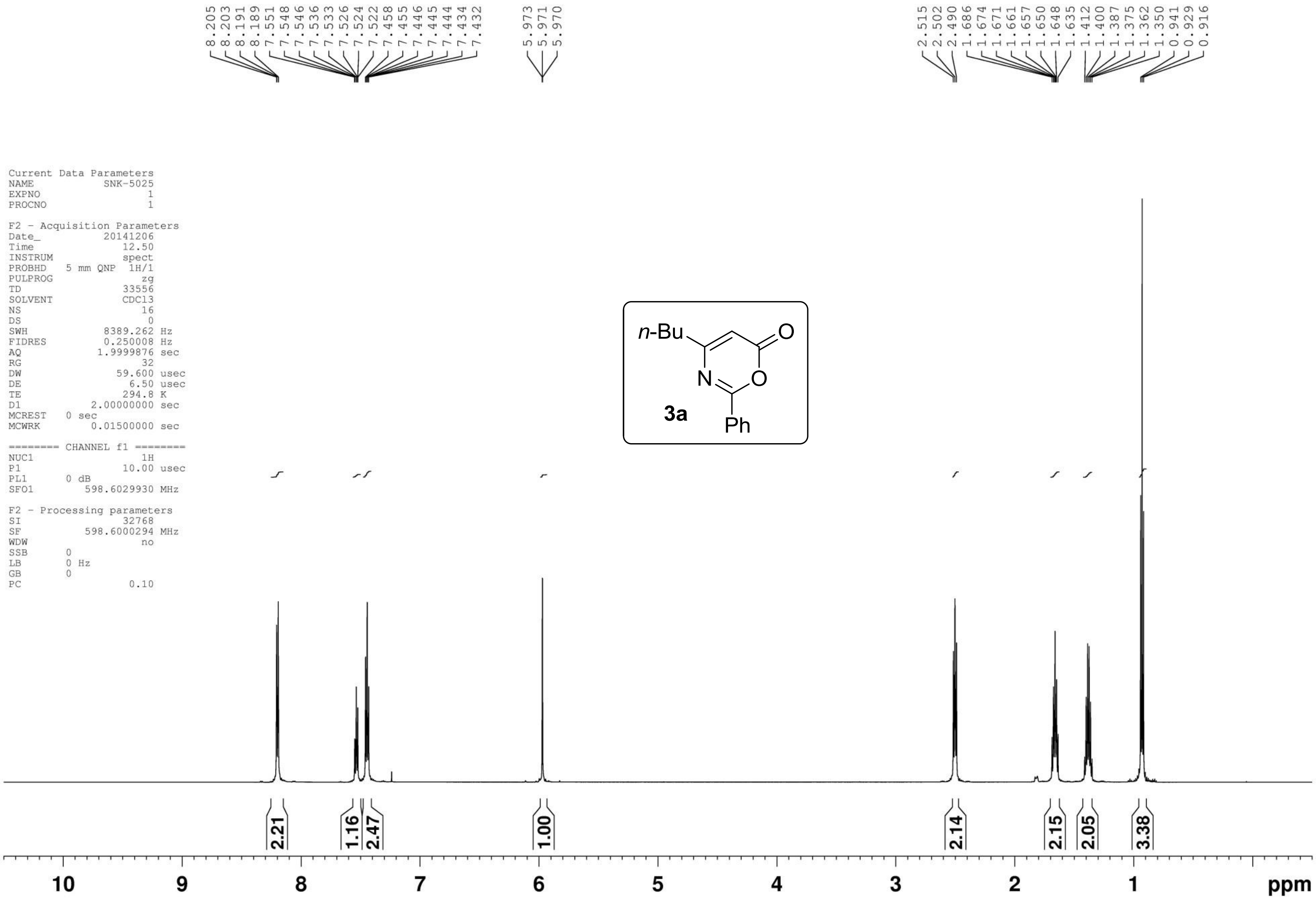

Current Data Parameters
NAME SNK-5025
EXPNO 1
PROCNO 1

F2 - Acquisition Parameters
Date_ 20141206
Time 12.50
INSTRUM spect
PROBHD 5 mm QNP 1H/1
PULPROG zg
TD 33556
SOLVENT CDCl3
NS 16
DS 0
SWH 8389.262 Hz
FIDRES 0.250008 Hz
AQ 1.9999876 sec
RG 32
DW 59.600 usec
DE 6.50 usec
TE 294.8 K
D1 2.00000000 sec
MCREST 0 sec
MCWRK 0.01500000 sec

======== CHANNEL f1 ========
NUC1 1H
P1 10.00 usec
PL1 0 dB
SFO1 598.6029930 MHz

F2 - Processing parameters
SI 32768
SF 598.6000294 MHz
WDW no
SSB 0
LB 0 Hz
GB 0
PC 0.10

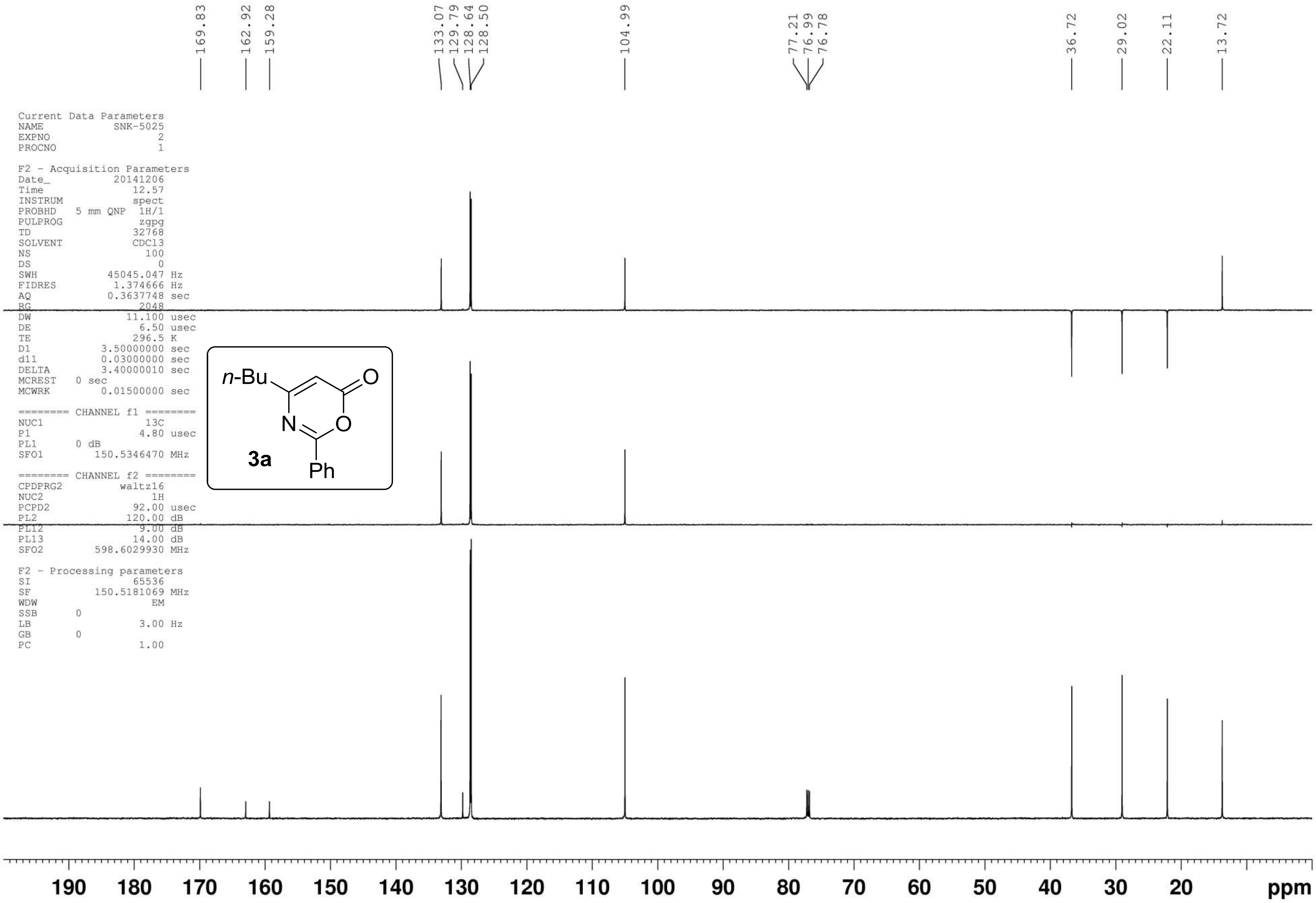
169.83
162.92
159.28
133.07
129.79
128.64
128.50
104.99
77.21
76.99
76.78
36.72
29.02
22.11
13.72
Current Data Parameters
NAME SNK-5025
EXPNO 2
PROCNO 1
F2 - Acquisition Parameters
Date_ 20141206
Time 12.57
INSTRUM spect
PROBHD 5 mm QNP 1H/1
PULPROG zgpg
TD 32768
SOLVENT CDCl3
NS 100
DS 0
SWH 45045.047 Hz
FIDRES 1.374666 Hz
AQ 0.3637748 sec
RG 2048
DW 11.100 usec
DE 6.50 usec
TE 296.5 K
D1 3.50000000 sec
d11 0.03000000 sec
DELTA 3.40000010 sec
MCREST 0 sec
MCWRK 0.01500000 sec
======== CHANNEL f1 ========
NUC1 13C
P1 4.80 usec
PL1 0 dB
SFO1 150.5346470 MHz
======== CHANNEL f2 ========
CPDPRG2 waltz16
NUC2 1H
PCPD2 92.00 usec
PL2 120.00 dB
PL12 9.00 dB
PL13 14.00 dB
SFO2 598.6029930 MHz
F2 - Processing parameters
SI 65536
SF 150.5181069 MHz
WDW EM
SSB 0
LB 3.00 Hz
GB 0
PC 1.00
190 180 170 160 150 140 130 120 110 100 90 80 70 60 50 40 30 20 ppm
n-Bu
O
N
O
3a
Ph

8.201
8.199
8.191
8.187
8.185
7.809
7.798
7.587
7.585
7.583
7.575
7.572
7.562
7.560
7.558
7.489
7.487
7.484
7.473
7.464
7.461
7.459
7.240
6.201
6.190

1.678

Current Data Parameters
NAME SNK-5038
EXPNO 1
PROCNO 1

F2 - Acquisition Parameters
Date_ 20141218
Time 7.19
INSTRUM spect
PROBHD 5 mm QNP 1H/1
PULPROG zg
TD 33556
SOLVENT CDCl3
NS 16
DS 0
SWH 12019.230 Hz
FIDRES 0.358184 Hz
AQ 1.3959796 sec
RG 32
DW 41.600 usec
DE 6.50 usec
TE 293.2 K
D1 2.00000000 sec
MCREST 0 sec
MCWRK 0.01500000 sec

======== CHANNEL f1 ========
NUC1 1H
P1 10.00 usec
PL1 0 dB
SFO1 598.6035916 MHz

F2 - Processing parameters
SI 32768
SF 598.6000302 MHz
WDW EM
SSB 0
LB 0.10 Hz
GB 0
PC 0.10

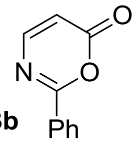

3b

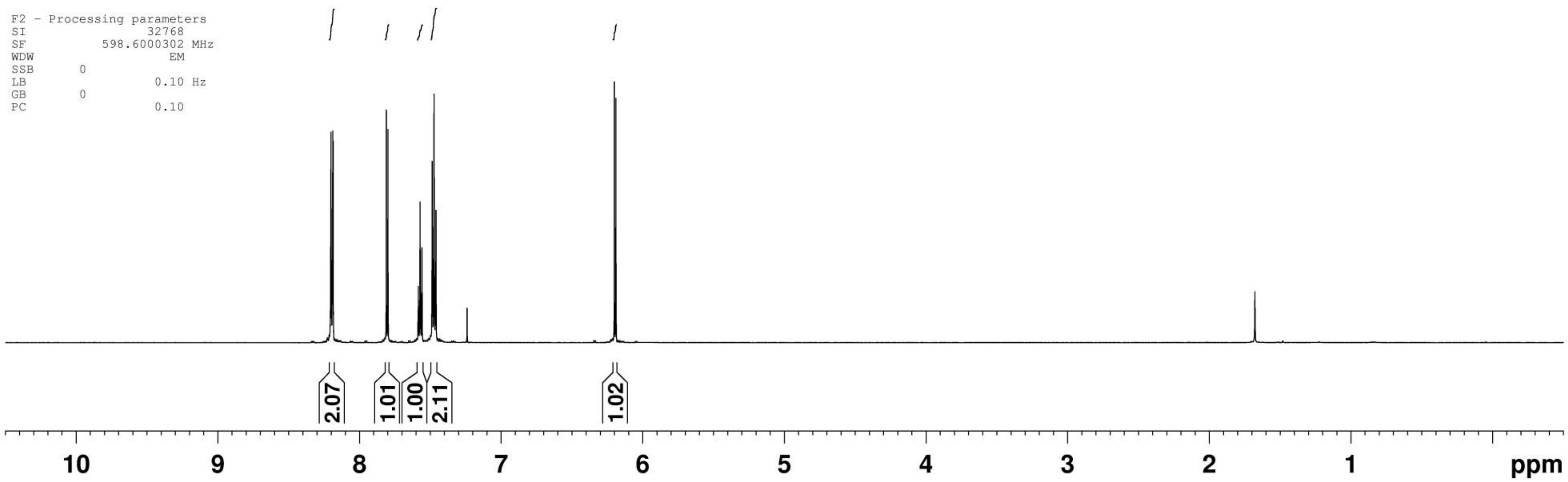

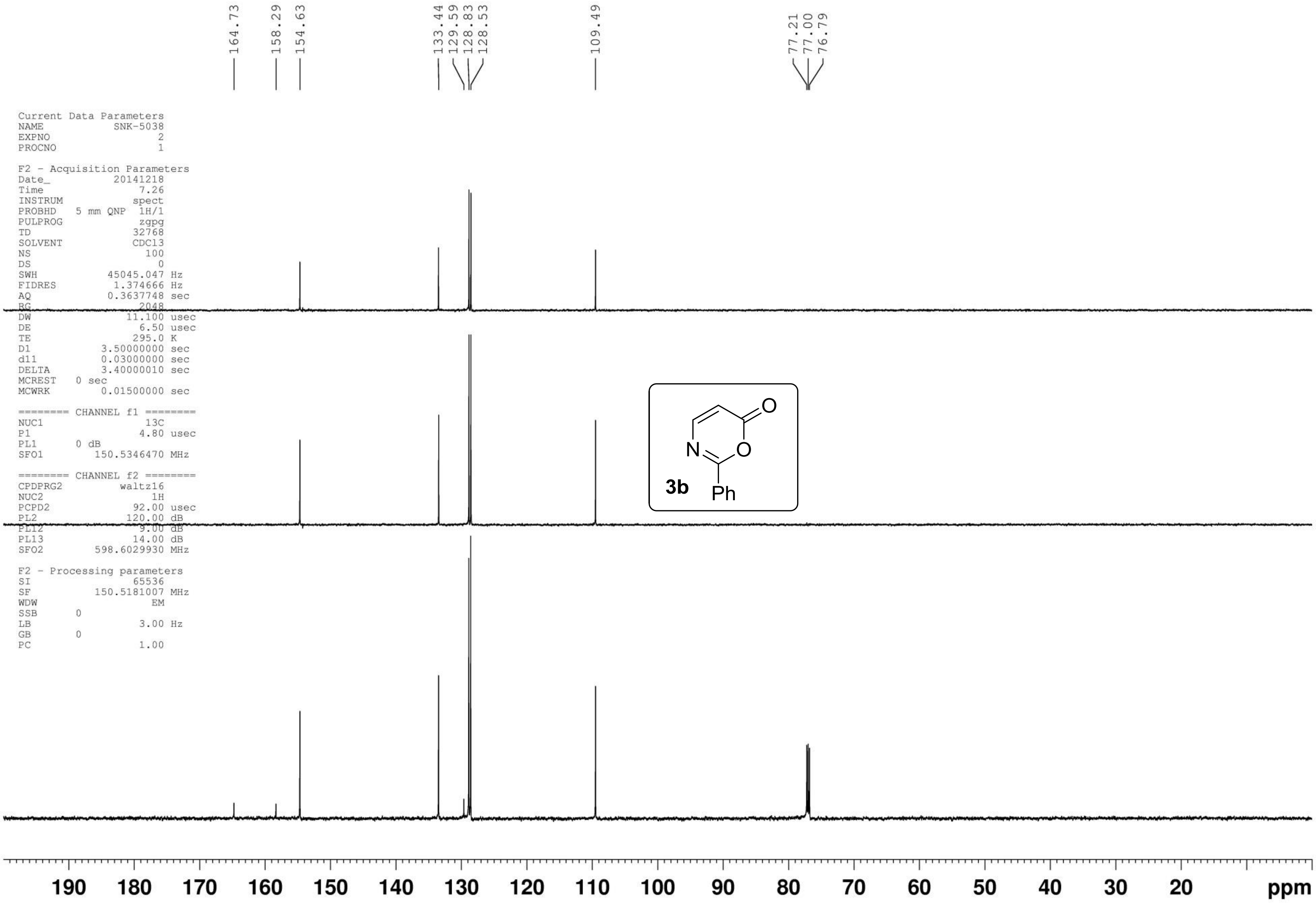
164.73
158.29
154.63
133.44
129.59
128.83
128.53
109.49
77.21
77.00
76.79
Current Data Parameters
NAME SNK-5038
EXPNO 2
PROCNO 1
F2 - Acquisition Parameters
Date_ 20141218
Time 7.26
INSTRUM spect
PROBHD 5 mm QNP 1H/1
PULPROG zgpg
TD 32768
SOLVENT CDC13
NS 100
DS 0
SWH 45045.047 Hz
FIDRES 1.374666 Hz
AQ 0.3637748 sec
RG 2048
DW 11.100 usec
DE 6.50 usec
TE 295.0 K
D1 3.50000000 sec
d11 0.03000000 sec
DELTA 3.40000010 sec
MCREST 0 sec
MCWRK 0.01500000 sec
======== CHANNEL f1 ========
NUC1 13C
P1 4.80 usec
PL1 0 dB
SFO1 150.5346470 MHz
======== CHANNEL f2 ========
CPDPRG2 waltz16
NUC2 1H
PCPD2 92.00 usec
PL2 120.00 dB
PL12 9.00 dB
PL13 14.00 dB
SFO2 598.6029930 MHz
F2 - Processing parameters
SI 65536
SF 150.5181007 MHz
WDW EM
SSB 0
LB 3.00 Hz
GB 0
PC 1.00
N
O
O
3b
Ph
190 180 170 160 150 140 130 120 110 100 90 80 70 60 50 40 30 20 ppm

Current Data Parameters
NAME SNK-5028
EXPNO 1
PROCNO 1

F2 - Acquisition Parameters
Date_ 20141215
Time 9.21
INSTRUM spect
PROBHD 5 mm QNP 1H/1
PULPROG zg
TD 33556
SOLVENT CDCl3
NS 16
DS 0
SWH 8389.262 Hz
FIDRES 0.250008 Hz
AQ 1.9999876 sec
RG 64
DW 59.600 usec
DE 6.50 usec
TE 294.8 K
D1 2.00000000 sec
MCREST 0 sec
MCWRK 0.01500000 sec

======== CHANNEL f1 ========
NUC1 1H
P1 10.00 usec
PL1 0 dB
SFO1 598.6029930 MHz

F2 - Processing parameters
SI 32768
SF 598.6000286 MHz
WDW no
SSB 0
LB 0 Hz
GB 0
PC 0.10

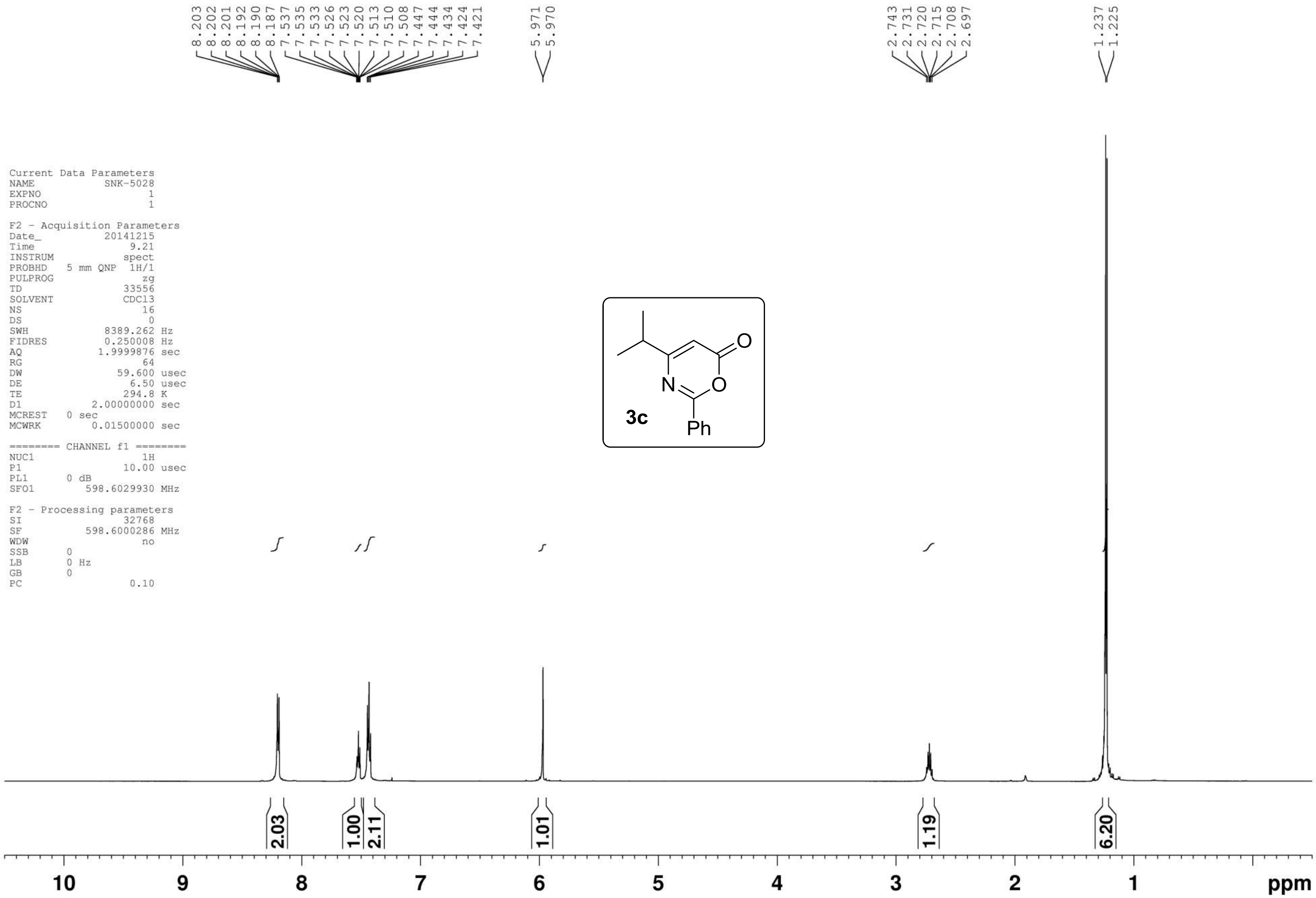

3c

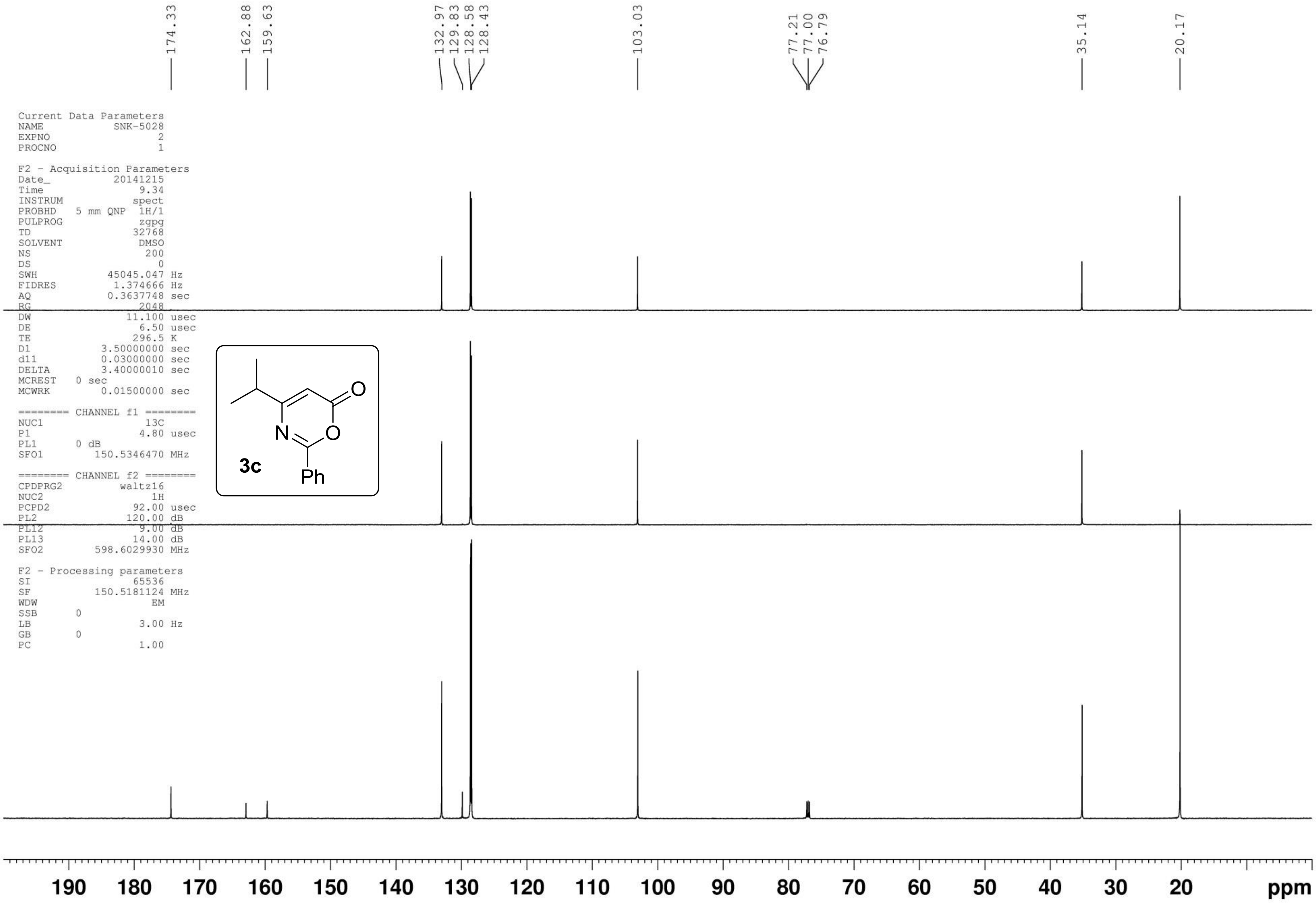
174.33
162.88
159.63
132.97
129.83
128.58
128.43
103.03
77.21
77.00
76.79
35.14
20.17
Current Data Parameters
NAME SNK-5028
EXPNO 2
PROCNO 1
F2 - Acquisition Parameters
Date_ 20141215
Time 9.34
INSTRUM spect
PROBHD 5 mm QNP 1H/1
PULPROG zgpg
TD 32768
SOLVENT DMSO
NS 200
DS 0
SWH 45045.047 Hz
FIDRES 1.374666 Hz
AQ 0.3637748 sec
RG 2048
DW 11.100 usec
DE 6.50 usec
TE 296.5 K
D1 3.50000000 sec
d11 0.03000000 sec
DELTA 3.40000010 sec
MCREST 0 sec
MCWRK 0.01500000 sec
======== CHANNEL f1 ========
NUC1 13C
P1 4.80 usec
PL1 0 dB
SFO1 150.5346470 MHz
======== CHANNEL f2 ========
CPDPRG2 waltz16
NUC2 1H
PCPD2 92.00 usec
PL2 120.00 dB
PL12 9.00 dB
PL13 14.00 dB
SFO2 598.6029930 MHz
F2 - Processing parameters
SI 65536
SF 150.5181124 MHz
WDW EM
SSB 0
LB 3.00 Hz
GB 0
PC 1.00
O
N
O
3c
Ph
190 180 170 160 150 140 130 120 110 100 90 80 70 60 50 40 30 20 ppm

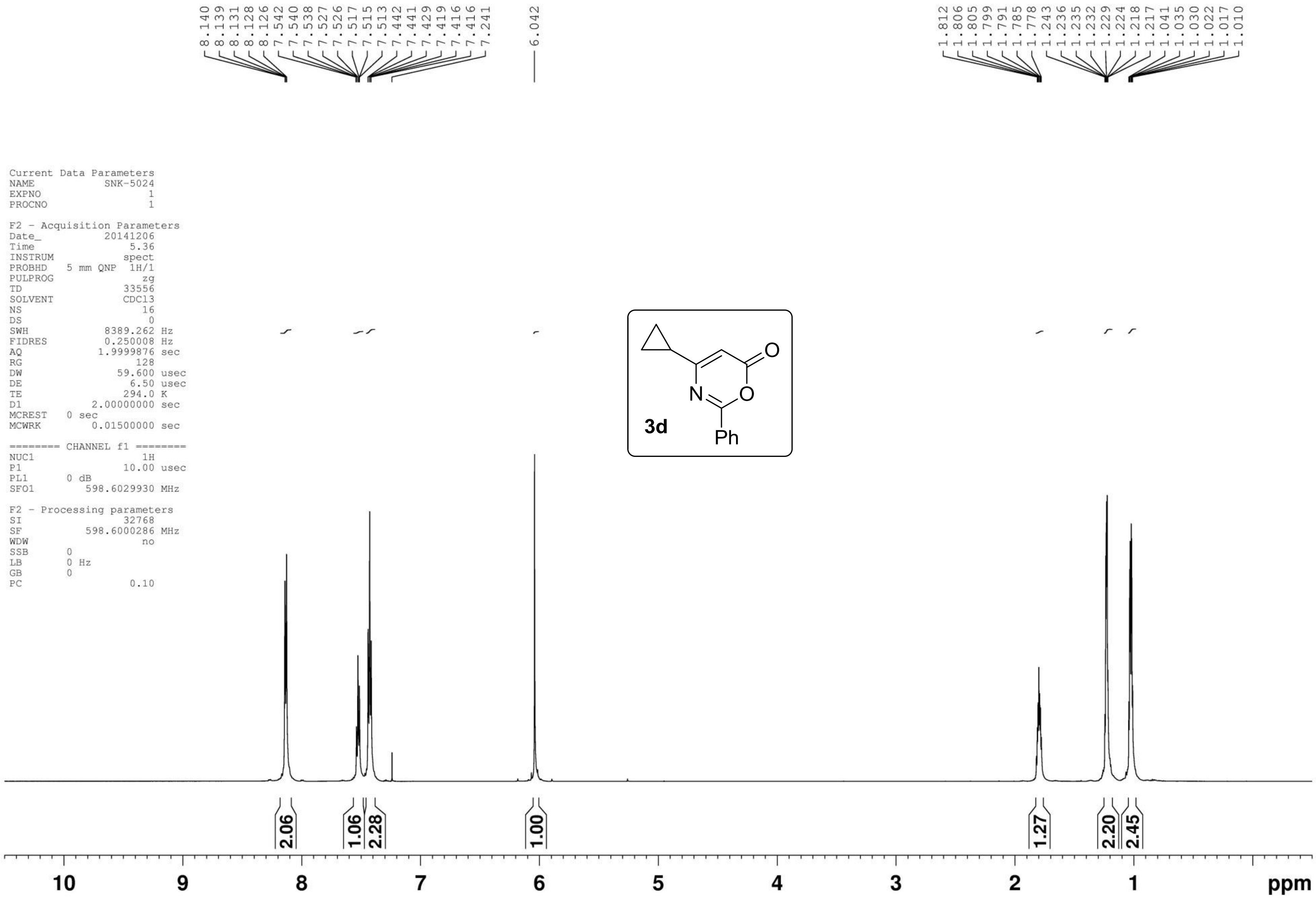
8.140
8.139
8.131
8.128
8.126
7.542
7.540
7.538
7.527
7.526
7.517
7.515
7.513
7.442
7.441
7.429
7.419
7.416
7.416
7.241
6.042
1.812
1.806
1.805
1.799
1.791
1.785
1.778
1.243
1.236
1.235
1.232
1.229
1.224
1.218
1.217
1.041
1.035
1.030
1.022
1.017
1.010
Current Data Parameters
NAME SNK-5024
EXPNO 1
PROCNO 1
F2 - Acquisition Parameters
Date_ 20141206
Time 5.36
INSTRUM spect
PROBHD 5 mm QNP 1H/1
PULPROG zg
TD 33556
SOLVENT CDCl3
NS 16
DS 0
SWH 8389.262 Hz
FIDRES 0.250008 Hz
AQ 1.9999876 sec
RG 128
DW 59.600 usec
DE 6.50 usec
TE 294.0 K
D1 2.00000000 sec
MCREST 0 sec
MCWRK 0.01500000 sec
======== CHANNEL f1 ========
NUC1 1H
P1 10.00 usec
PL1 0 dB
SFO1 598.6029930 MHz
F2 - Processing parameters
SI 32768
SF 598.6000286 MHz
WDW no
SSB 0
LB 0 Hz
GB 0
PC 0.10
O
N
O
3d
Ph
2.06
1.06
2.28
1.00
1.27
2.20
2.45
10
9
8
7
6
5
4
3
2
1
ppm

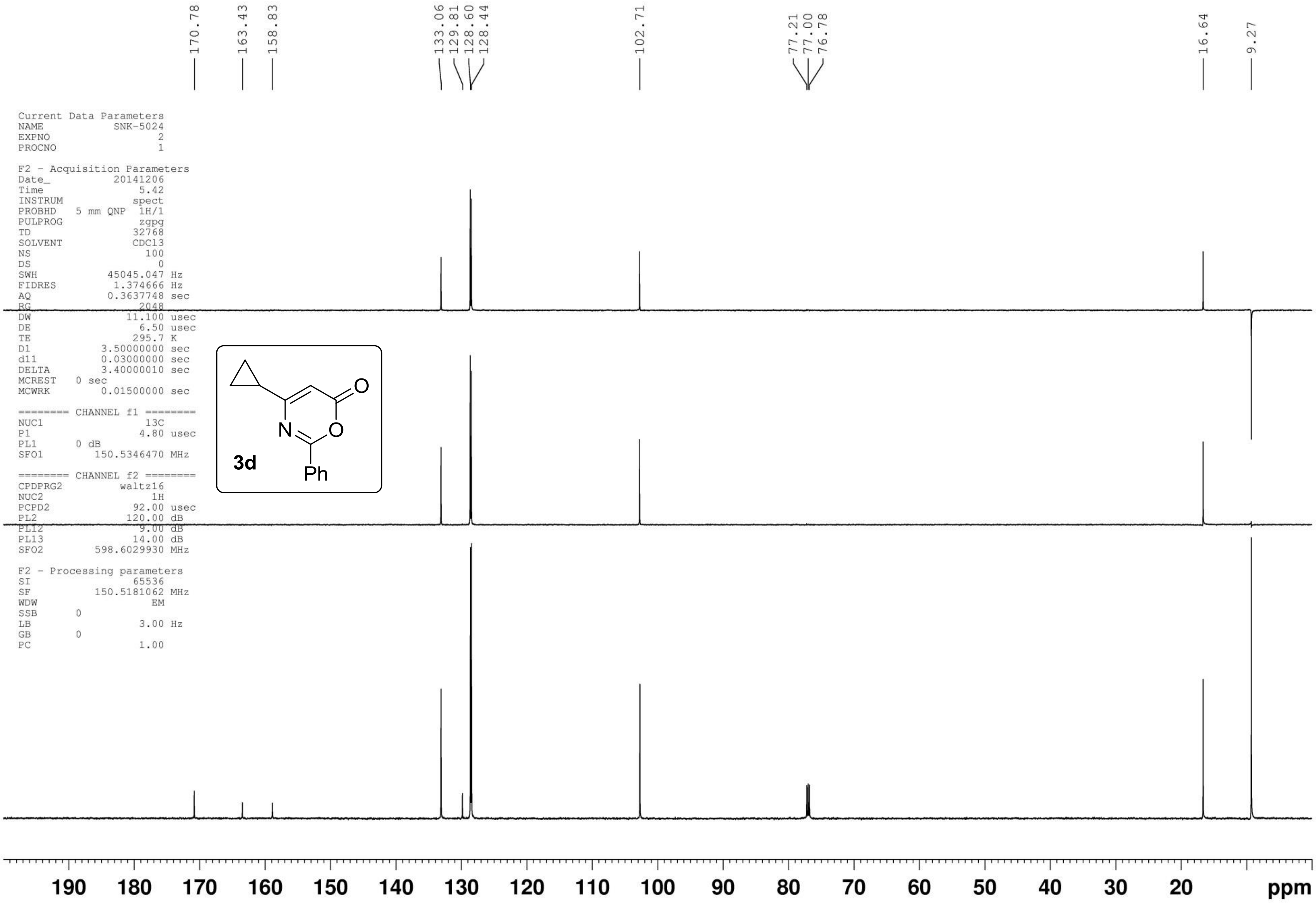
170.78
163.43
158.83
133.06
129.81
128.60
128.44
102.71
77.21
77.00
76.78
16.64
9.27
Current Data Parameters
NAME SNK-5024
EXPNO 2
PROCNO 1
F2 - Acquisition Parameters
Date_ 20141206
Time 5.42
INSTRUM spect
PROBHD 5 mm QNP 1H/1
PULPROG zgpg
TD 32768
SOLVENT CDCl3
NS 100
DS 0
SWH 45045.047 Hz
FIDRES 1.374666 Hz
AQ 0.3637748 sec
RG 2048
DW 11.100 usec
DE 6.50 usec
TE 295.7 K
D1 3.50000000 sec
d11 0.03000000 sec
DELTA 3.40000010 sec
MCREST 0 sec
MCWRK 0.01500000 sec
======== CHANNEL f1 ========
NUC1 13C
P1 4.80 usec
PL1 0 dB
SFO1 150.5346470 MHz
======== CHANNEL f2 ========
CPDPRG2 waltz16
NUC2 1H
PCPD2 92.00 usec
PL2 120.00 dB
PL12 9.00 dB
PL13 14.00 dB
SFO2 598.6029930 MHz
F2 - Processing parameters
SI 65536
SF 150.5181062 MHz
WDW EM
SSB 0
LB 3.00 Hz
GB 0
PC 1.00
O
N
O
3d
Ph
190 180 170 160 150 140 130 120 110 100 90 80 70 60 50 40 30 20 ppm

Current Data Parameters
NAME SNK-5026
EXPNO 1
PROCNO 1

F2 - Acquisition Parameters
Date_ 20141209
Time 6.15
INSTRUM spect
PROBHD 5 mm QNP 1H/1
PULPROG zg
TD 33556
SOLVENT CDCl3
NS 16
DS 0
SWH 8389.262 Hz
FIDRES 0.250008 Hz
AQ 1.9999876 sec
RG 128
DW 59.600 usec
DE 6.50 usec
TE 294.9 K
D1 2.00000000 sec
MCREST 0 sec
MCWRK 0.01500000 sec

======== CHANNEL f1 ========
NUC1 1H
P1 10.00 usec
PL1 0 dB
SFO1 598.6029930 MHz

F2 - Processing parameters
SI 32768
SF 598.6000286 MHz
WDW no
SSB 0
LB 0 Hz
GB 0
PC 0.10

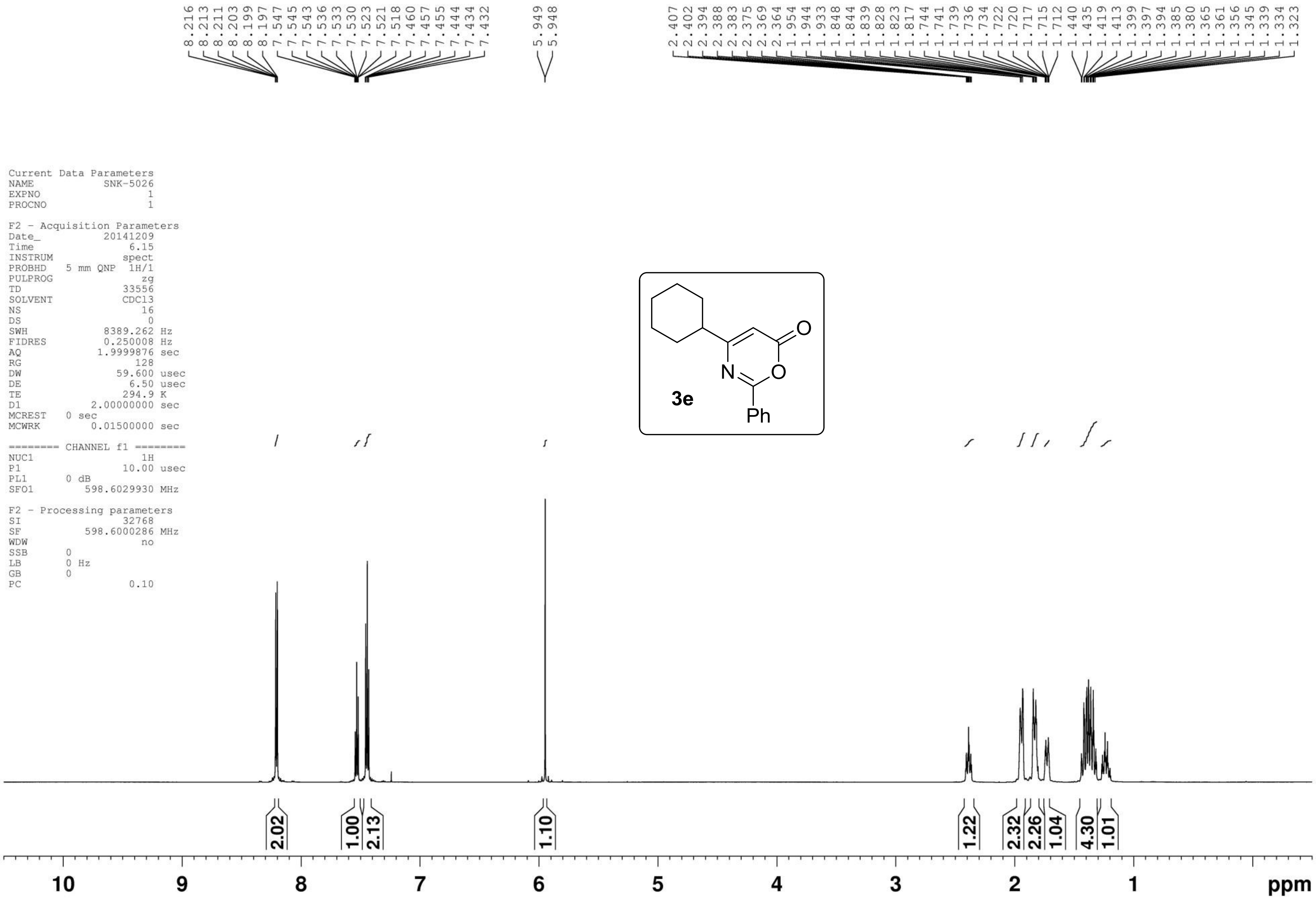

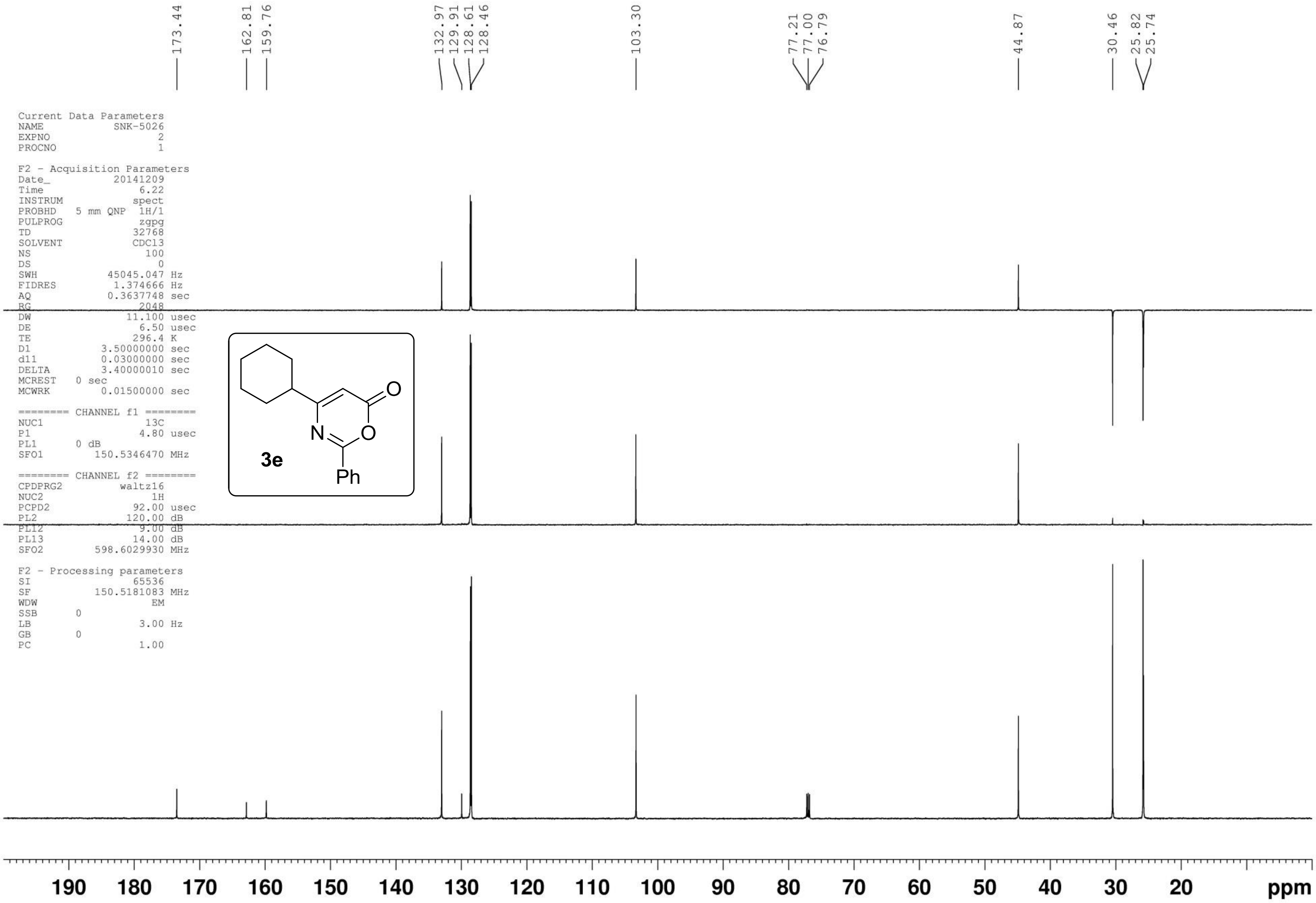

Current Data Parameters
NAME SNK-5026
EXPNO 2
PROCNO 1

F2 - Acquisition Parameters
Date_ 20141209
Time 6.22
INSTRUM spect
PROBHD 5 mm QNP 1H/1
PULPROG zgpg
TD 32768
SOLVENT CDCl3
NS 100
DS 0
SWH 45045.047 Hz
FIDRES 1.374666 Hz
AQ 0.3637748 sec
RG 2048
DW 11.100 usec
DE 6.50 usec
TE 296.4 K
D1 3.50000000 sec
d11 0.03000000 sec
DELTA 3.40000010 sec
MCREST 0 sec
MCWRK 0.01500000 sec

======== CHANNEL f1 ========
NUC1 13C
P1 4.80 usec
PL1 0 dB
SFO1 150.5346470 MHz

======== CHANNEL f2 ========
CPDPRG2 waltz16
NUC2 1H
PCPD2 92.00 usec
PL2 120.00 dB
PL12 9.00 dB
PL13 14.00 dB
SFO2 598.6029930 MHz

F2 - Processing parameters
SI 65536
SF 150.5181083 MHz
WDW EM
SSB 0
LB 3.00 Hz
GB 0
PC 1.00

3e

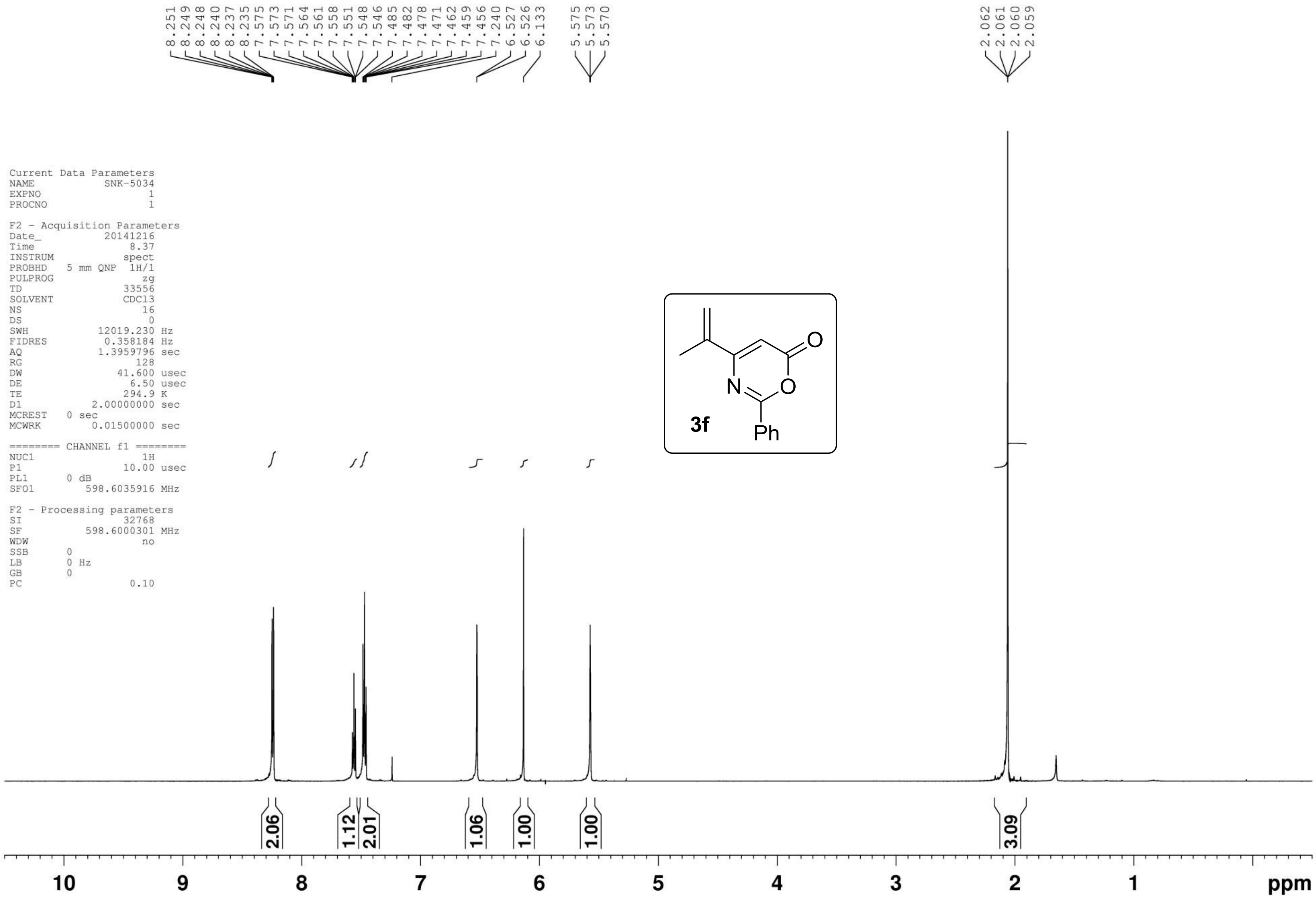
8.251
8.249
8.248
8.240
8.237
8.235
7.575
7.573
7.571
7.564
7.561
7.558
7.551
7.548
7.546
7.485
7.482
7.478
7.471
7.462
7.459
7.456
7.240
6.527
6.526
6.133
5.575
5.573
5.570
2.062
2.061
2.060
2.059
Current Data Parameters
NAME SNK-5034
EXPNO 1
PROCNO 1
F2 - Acquisition Parameters
Date_ 20141216
Time 8.37
INSTRUM spect
PROBHD 5 mm QNP 1H/1
PULPROG zg
TD 33556
SOLVENT CDCl3
NS 16
DS 0
SWH 12019.230 Hz
FIDRES 0.358184 Hz
AQ 1.3959796 sec
RG 128
DW 41.600 usec
DE 6.50 usec
TE 294.9 K
D1 2.00000000 sec
MCREST 0 sec
MCWRK 0.01500000 sec
======== CHANNEL f1 ========
NUC1 1H
P1 10.00 usec
PL1 0 dB
SFO1 598.6035916 MHz
F2 - Processing parameters
SI 32768
SF 598.6000301 MHz
WDW no
SSB 0
LB 0 Hz
GB 0
PC 0.10
O
N
O
3f
Ph
2.06
1.12
2.01
1.06
1.00
1.00
3.09
10
9
8
7
6
5
4
3
2
1
ppm

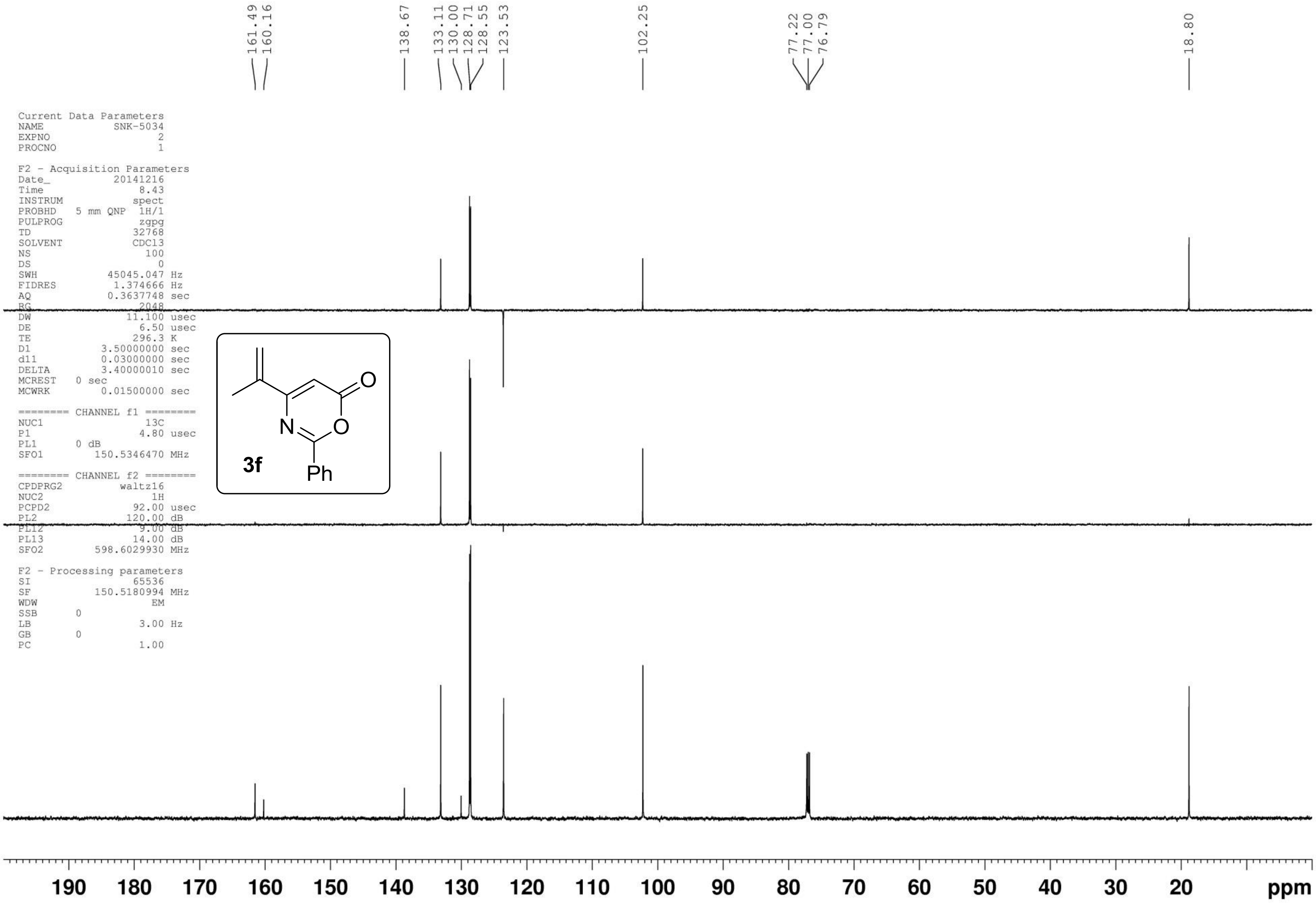
161.49
160.16
138.67
133.11
130.00
128.71
128.55
123.53
102.25
77.22
77.00
76.79
18.80
Current Data Parameters
NAME SNK-5034
EXPNO 2
PROCNO 1
F2 - Acquisition Parameters
Date_ 20141216
Time 8.43
INSTRUM spect
PROBHD 5 mm QNP 1H/1
PULPROG zgpg
TD 32768
SOLVENT CDCl3
NS 100
DS 0
SWH 45045.047 Hz
FIDRES 1.374666 Hz
AQ 0.3637748 sec
RG 2048
DW 11.100 usec
DE 6.50 usec
TE 296.3 K
D1 3.50000000 sec
d11 0.03000000 sec
DELTA 3.40000010 sec
MCREST 0 sec
MCWRK 0.01500000 sec
======== CHANNEL f1 ========
NUC1 13C
P1 4.80 usec
PL1 0 dB
SFO1 150.5346470 MHz
======== CHANNEL f2 ========
CPDPRG2 waltz16
NUC2 1H
PCPD2 92.00 usec
PL2 120.00 dB
PL12 9.00 dB
PL13 14.00 dB
SFO2 598.6029930 MHz
F2 - Processing parameters
SI 65536
SF 150.5180994 MHz
WDW EM
SSB 0
LB 3.00 Hz
GB 0
PC 1.00
O
N
O
3f
Ph
190 180 170 160 150 140 130 120 110 100 90 80 70 60 50 40 30 20 ppm

```
Current Data Parameters
NAME            SNK-4224
EXPNO                  1
PROCNO                 1

F2 - Acquisition Parameters
Date_          20140917
Time              12.30
INSTRUM           spect
PROBHD   5 mm QNP  1H/1
PULPROG              zg
TD                33556
SOLVENT           CDCl3
NS                   16
DS                    0
SWH            8389.262 Hz
FIDRES         0.250008 Hz
AQ            1.9999876 sec
RG                  512
DW               59.600 usec
DE                 6.50 usec
TE                303.3 K
D1           2.00000000 sec
MCREST       0.00000000 sec
MCWRK        0.01500000 sec

======== CHANNEL f1 ========
NUC1                 1H
P1                10.00 usec
PL1                0.00 dB
SFO1        598.7029935 MHz

F2 - Processing parameters
SI                32768
SF          598.7000263 MHz
WDW                  no
SSB                   0
LB                 0.00 Hz
GB                    0
PC                 1.00

1D NMR plot parameters
CX                20.00 cm
CY                 6.00 cm
F1P              10.000 ppm
F1              5987.00 Hz
F2P              -0.500 ppm
F2              -299.35 Hz
PPMCM           0.52500 ppm/cm
HZCM          314.31750 Hz/cm
```

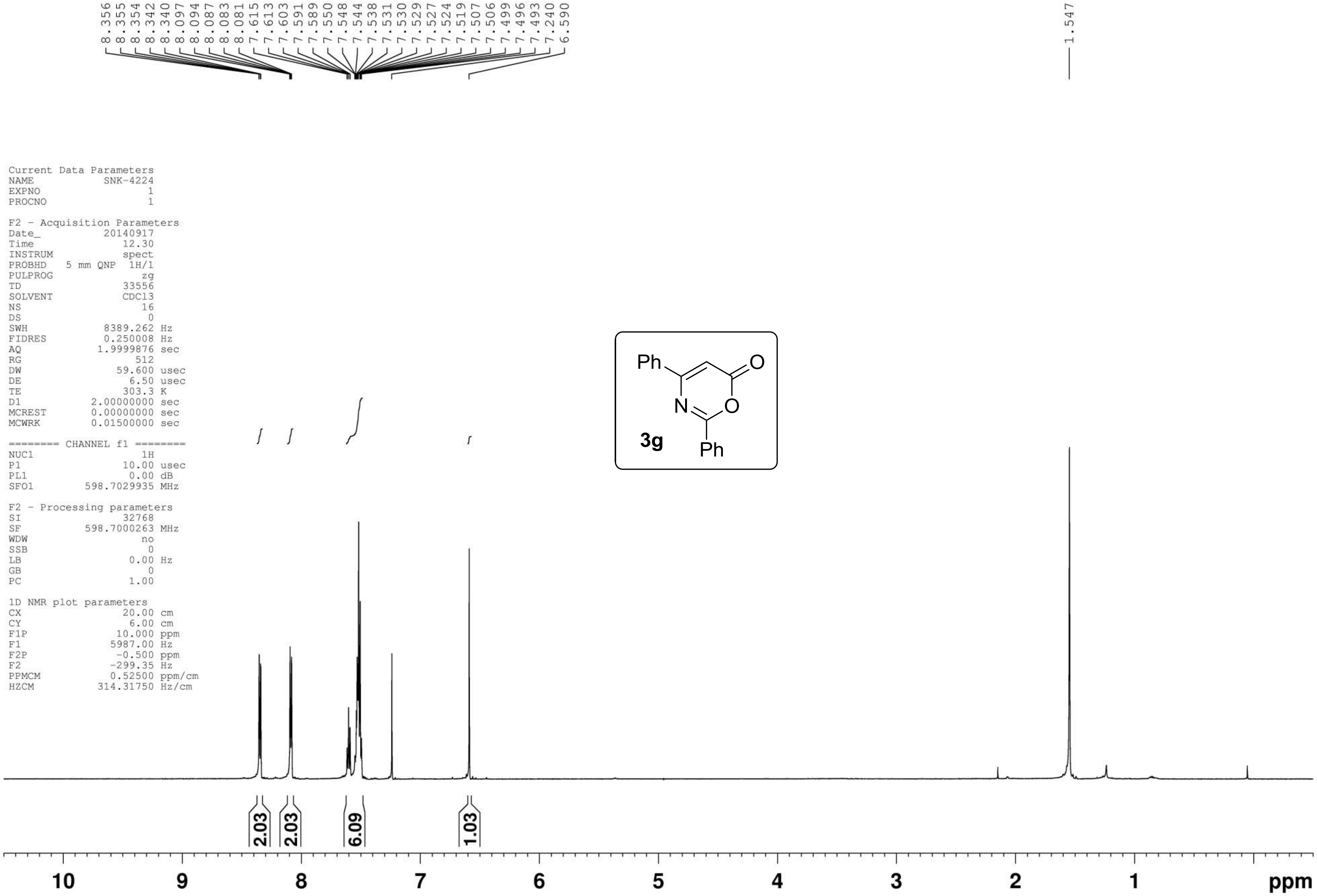

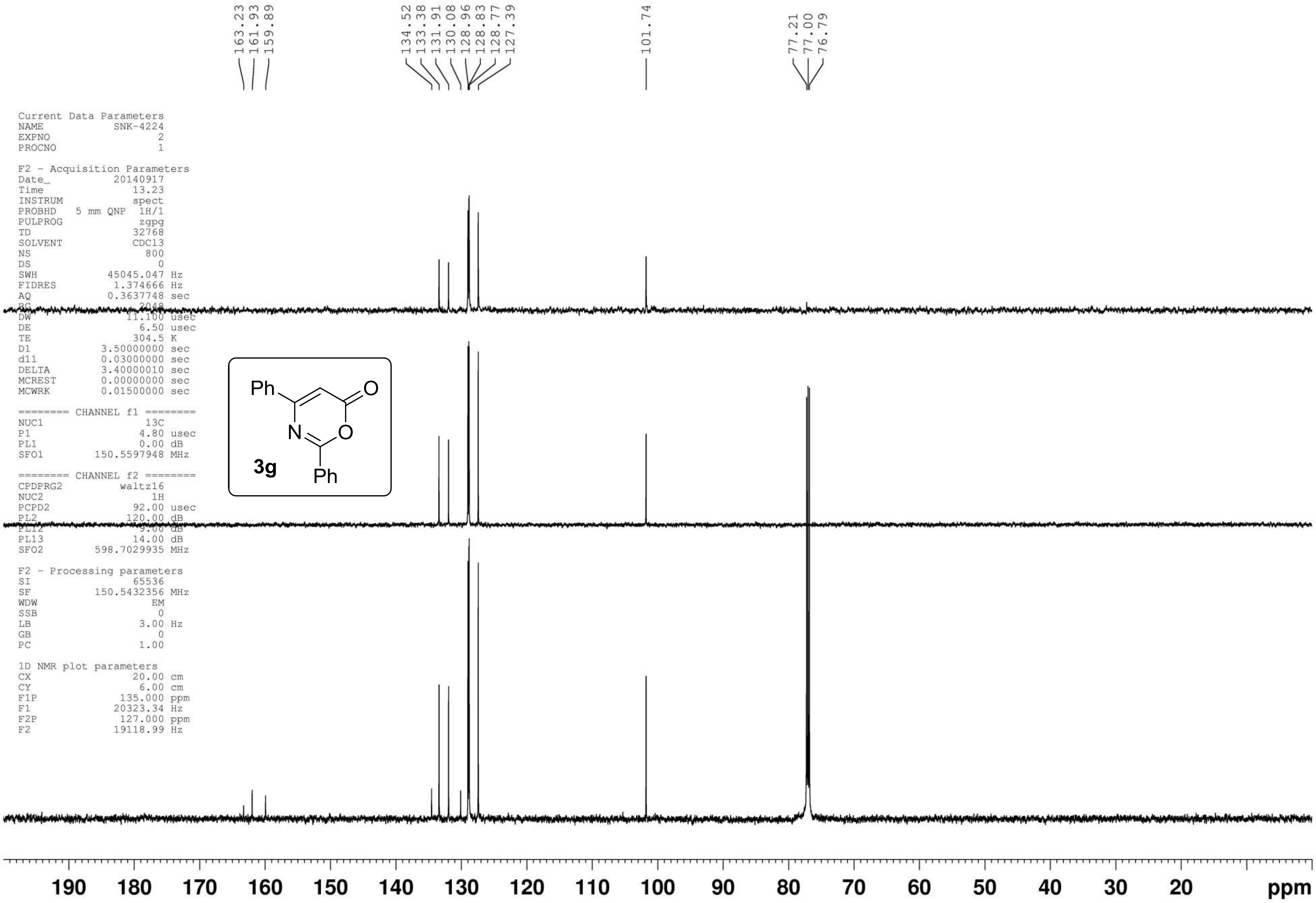

Current Data Parameters
NAME SNK-4224
EXPNO 2
PROCNO 1

F2 - Acquisition Parameters
Date_ 20140917
Time 13.23
INSTRUM spect
PROBHD 5 mm QNP 1H/1
PULPROG zgpg
TD 32768
SOLVENT CDCl3
NS 800
DS 0
SWH 45045.047 Hz
FIDRES 1.374666 Hz
AQ 0.3637748 sec
RG 2048
DW 11.100 usec
DE 6.50 usec
TE 304.5 K
D1 3.50000000 sec
d11 0.03000000 sec
DELTA 3.40000010 sec
MCREST 0.00000000 sec
MCWRK 0.01500000 sec

======== CHANNEL f1 ========
NUC1 13C
P1 4.80 usec
PL1 0.00 dB
SFO1 150.5597948 MHz

======== CHANNEL f2 ========
CPDPRG2 waltz16
NUC2 1H
PCPD2 92.00 usec
PL2 120.00 dB
PL12 9.00 dB
PL13 14.00 dB
SFO2 598.7029935 MHz

F2 - Processing parameters
SI 65536
SF 150.5432356 MHz
WDW EM
SSB 0
LB 3.00 Hz
GB 0
PC 1.00

1D NMR plot parameters
CX 20.00 cm
CY 6.00 cm
F1P 135.000 ppm
F1 20323.34 Hz
F2P 127.000 ppm
F2 19118.99 Hz

8.325
8.313
8.311
8.058
8.043
7.597
7.595
7.585
7.574
7.572
7.571
7.513
7.500
7.490
7.488
7.240
6.997
6.982
6.460
3.867

Current Data Parameters
NAME SNK-5013A
EXPNO 1
PROCNO 1

F2 - Acquisition Parameters
Date_ 20141202
Time 3.58
INSTRUM spect
PROBHD 5 mm QNP 1H/1
PULPROG zg
TD 33556
SOLVENT CDCl3
NS 16
DS 0
SWH 8389.262 Hz
FIDRES 0.250008 Hz
AQ 1.9999876 sec
RG 128
DW 59.600 usec
DE 6.50 usec
TE 295.6 K
D1 2.00000000 sec
MCREST 0 sec
MCWRK 0.01500000 sec

======== CHANNEL f1 ========
NUC1 1H
P1 10.00 usec
PL1 0 dB
SFO1 598.6029930 MHz

F2 - Processing parameters
SI 32768
SF 598.6000301 MHz
WDW no
SSB 0
LB 0 Hz
GB 0
PC 0.10

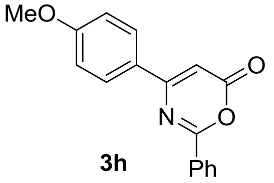

3h

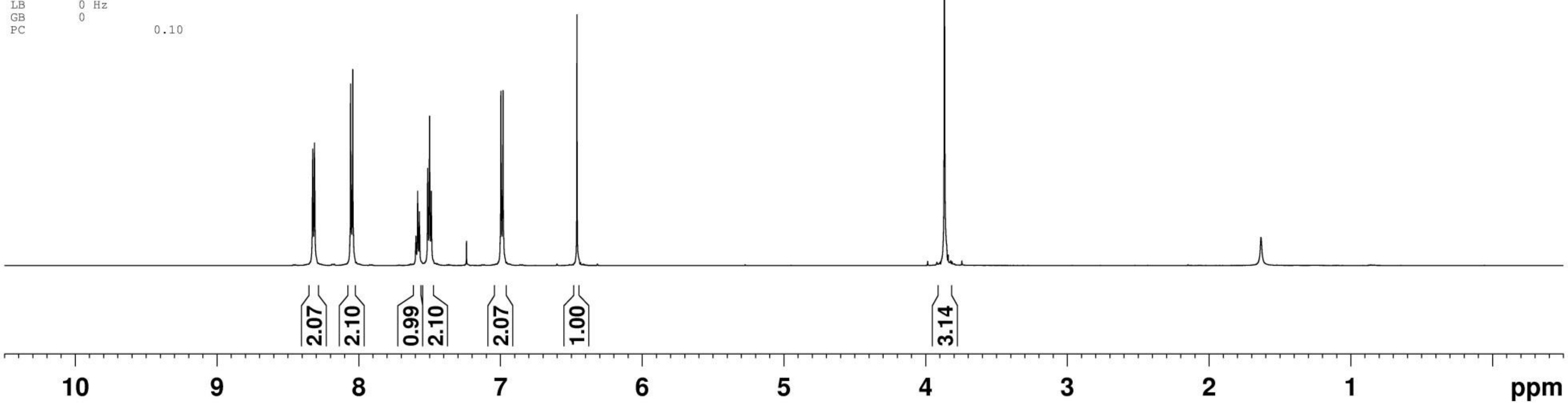

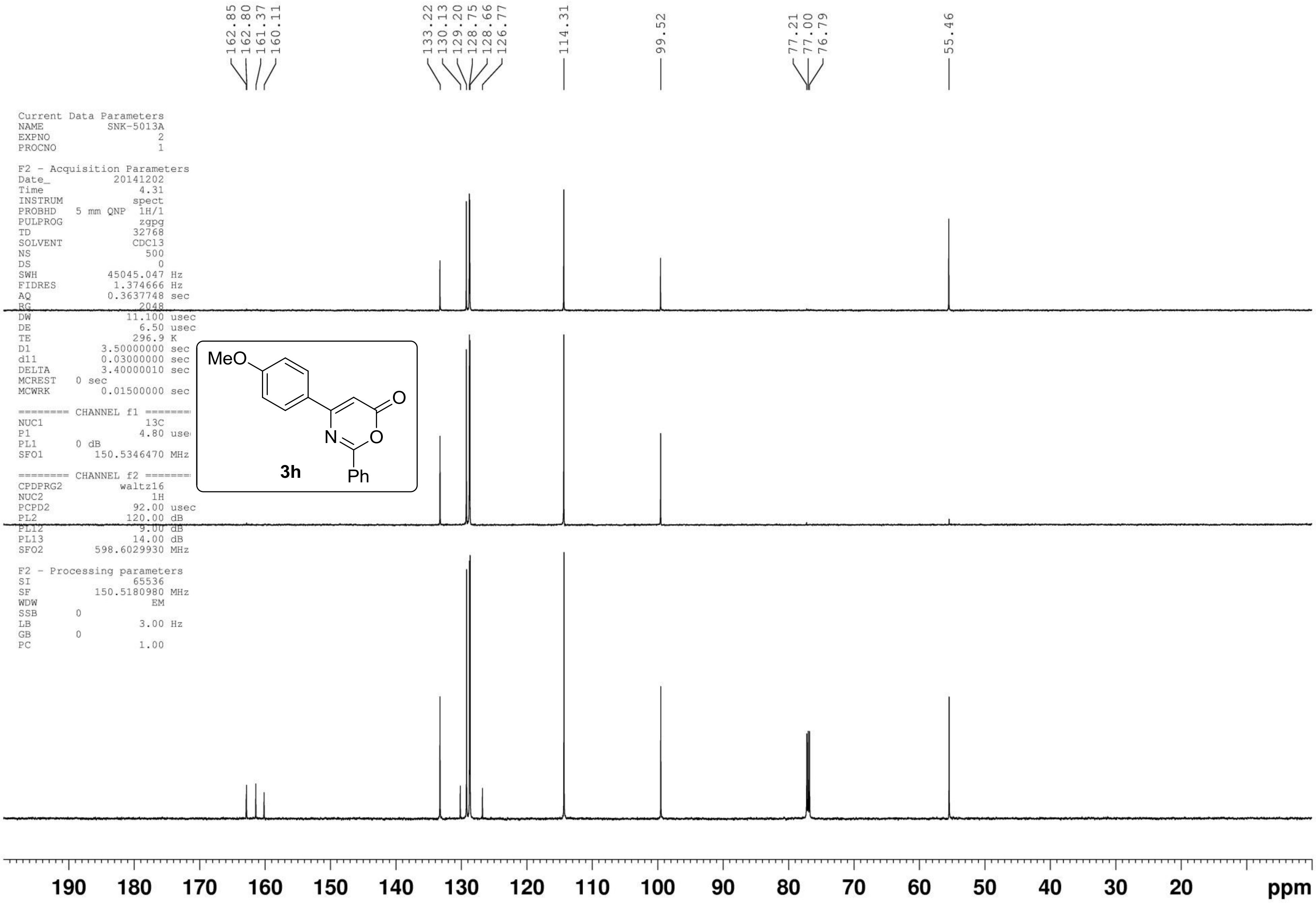
162.85
162.80
161.37
160.11
133.22
130.13
129.20
128.75
128.66
126.77
114.31
99.52
77.21
77.00
76.79
55.46
Current Data Parameters
NAME SNK-5013A
EXPNO 2
PROCNO 1
F2 - Acquisition Parameters
Date_ 20141202
Time 4.31
INSTRUM spect
PROBHD 5 mm QNP 1H/1
PULPROG zgpg
TD 32768
SOLVENT CDCl3
NS 500
DS 0
SWH 45045.047 Hz
FIDRES 1.374666 Hz
AQ 0.3637748 sec
RG 2048
DW 11.100 usec
DE 6.50 usec
TE 296.9 K
D1 3.50000000 sec
d11 0.03000000 sec
DELTA 3.40000010 sec
MCREST 0 sec
MCWRK 0.01500000 sec
======== CHANNEL f1 ========
NUC1 13C
P1 4.80 usec
PL1 0 dB
SFO1 150.5346470 MHz
======== CHANNEL f2 ========
CPDPRG2 waltz16
NUC2 1H
PCPD2 92.00 usec
PL2 120.00 dB
PL12 9.00 dB
PL13 14.00 dB
SFO2 598.6029930 MHz
F2 - Processing parameters
SI 65536
SF 150.5180980 MHz
WDW EM
SSB 0
LB 3.00 Hz
GB 0
PC 1.00
MeO
O
N
O
Ph
3h
190 180 170 160 150 140 130 120 110 100 90 80 70 60 50 40 30 20 ppm

8.323
8.321
8.309
8.307
8.100
8.096
8.091
8.088
8.088
8.085
8.079
8.076
7.614
7.612
7.610
7.603
7.599
7.597
7.589
7.587
7.585
7.522
7.519
7.508
7.499
7.496
7.240
7.194
7.190
7.185
7.182
7.179
7.168
7.165
6.515

1.633

```
Current Data Parameters
NAME            SNK-5017
EXPNO                  1
PROCNO                 1

F2 - Acquisition Parameters
Date_           20141209
Time                5.43
INSTRUM            spect
PROBHD   5 mm QNP  1H/1
PULPROG               zg
TD                 33556
SOLVENT            CDCl3
NS                    16
DS                     0
SWH             8389.262 Hz
FIDRES          0.250008 Hz
AQ             1.9999876 sec
RG                   128
DW                59.600 usec
DE                  6.50 usec
TE                 295.0 K
D1            2.00000000 sec
MCREST   0 sec
MCWRK         0.01500000 sec

======== CHANNEL f1 ========
NUC1                  1H
P1                 10.00 usec
PL1      0 dB
SFO1         598.6029930 MHz

F2 - Processing parameters
SI                 32768
SF           598.6000296 MHz
WDW                   no
SSB      0
LB       0 Hz
GB       0
PC                  0.10
```

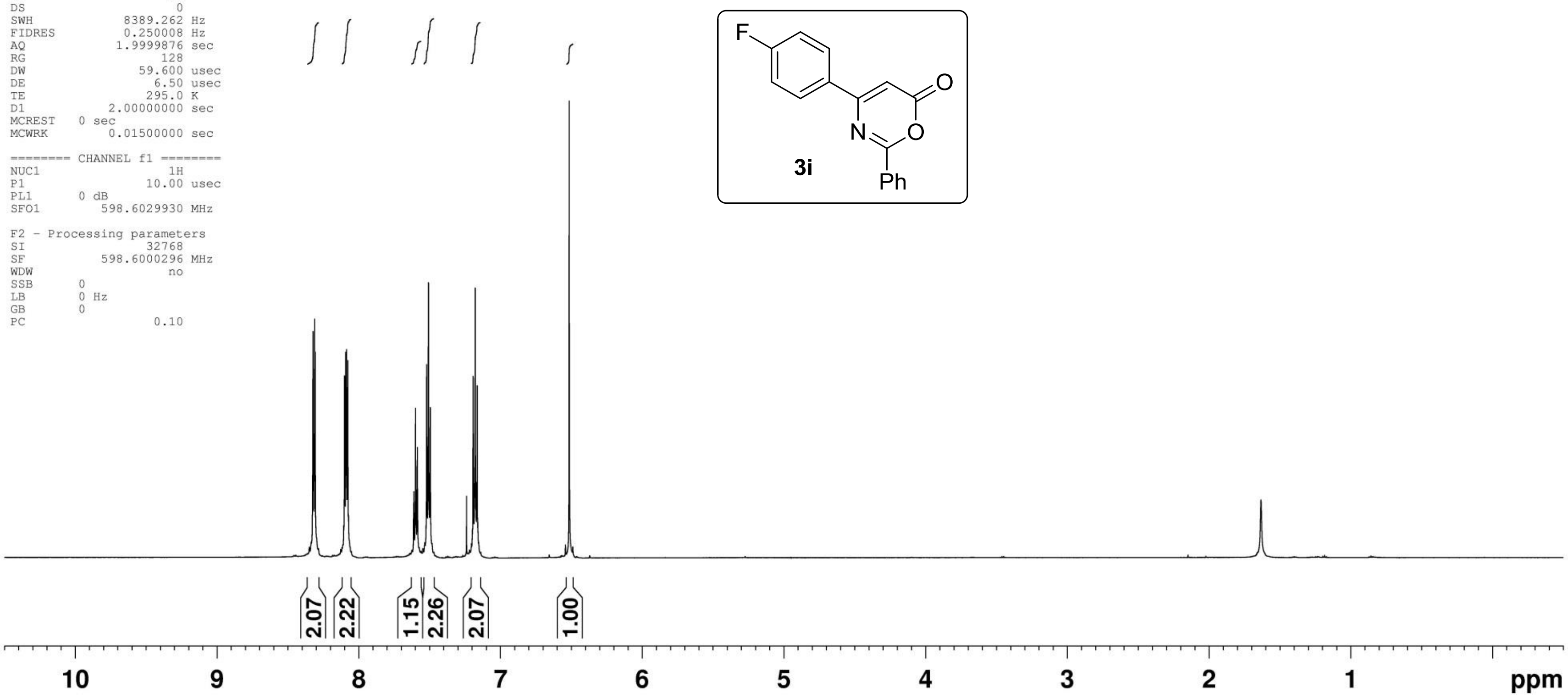

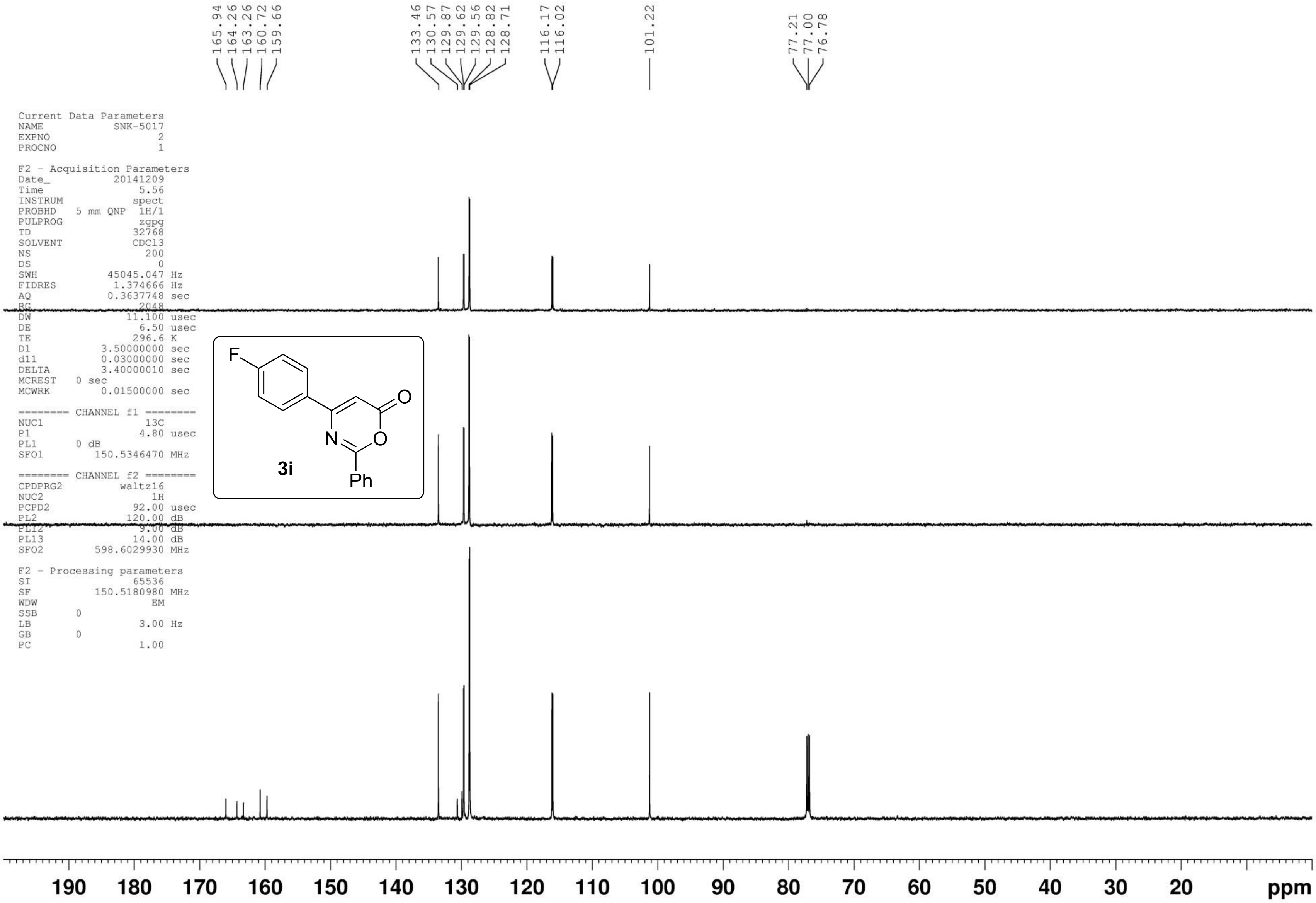
165.94
164.26
163.26
160.72
159.66
133.46
130.57
129.87
129.62
129.56
128.82
128.71
116.17
116.02
101.22
77.21
77.00
76.78
Current Data Parameters
NAME SNK-5017
EXPNO 2
PROCNO 1
F2 - Acquisition Parameters
Date_ 20141209
Time 5.56
INSTRUM spect
PROBHD 5 mm QNP 1H/1
PULPROG zgpg
TD 32768
SOLVENT CDCl3
NS 200
DS 0
SWH 45045.047 Hz
FIDRES 1.374666 Hz
AQ 0.3637748 sec
RG 2048
DW 11.100 usec
DE 6.50 usec
TE 296.6 K
D1 3.50000000 sec
d11 0.03000000 sec
DELTA 3.40000010 sec
MCREST 0 sec
MCWRK 0.01500000 sec
======== CHANNEL f1 ========
NUC1 13C
P1 4.80 usec
PL1 0 dB
SFO1 150.5346470 MHz
======== CHANNEL f2 ========
CPDPRG2 waltz16
NUC2 1H
PCPD2 92.00 usec
PL2 120.00 dB
PL12 9.00 dB
PL13 14.00 dB
SFO2 598.6029930 MHz
F2 - Processing parameters
SI 65536
SF 150.5180980 MHz
WDW EM
SSB 0
LB 3.00 Hz
GB 0
PC 1.00
F
O
N
O
Ph
3i
190 180 170 160 150 140 130 120 110 100 90 80 70 60 50 40 30 20 ppm

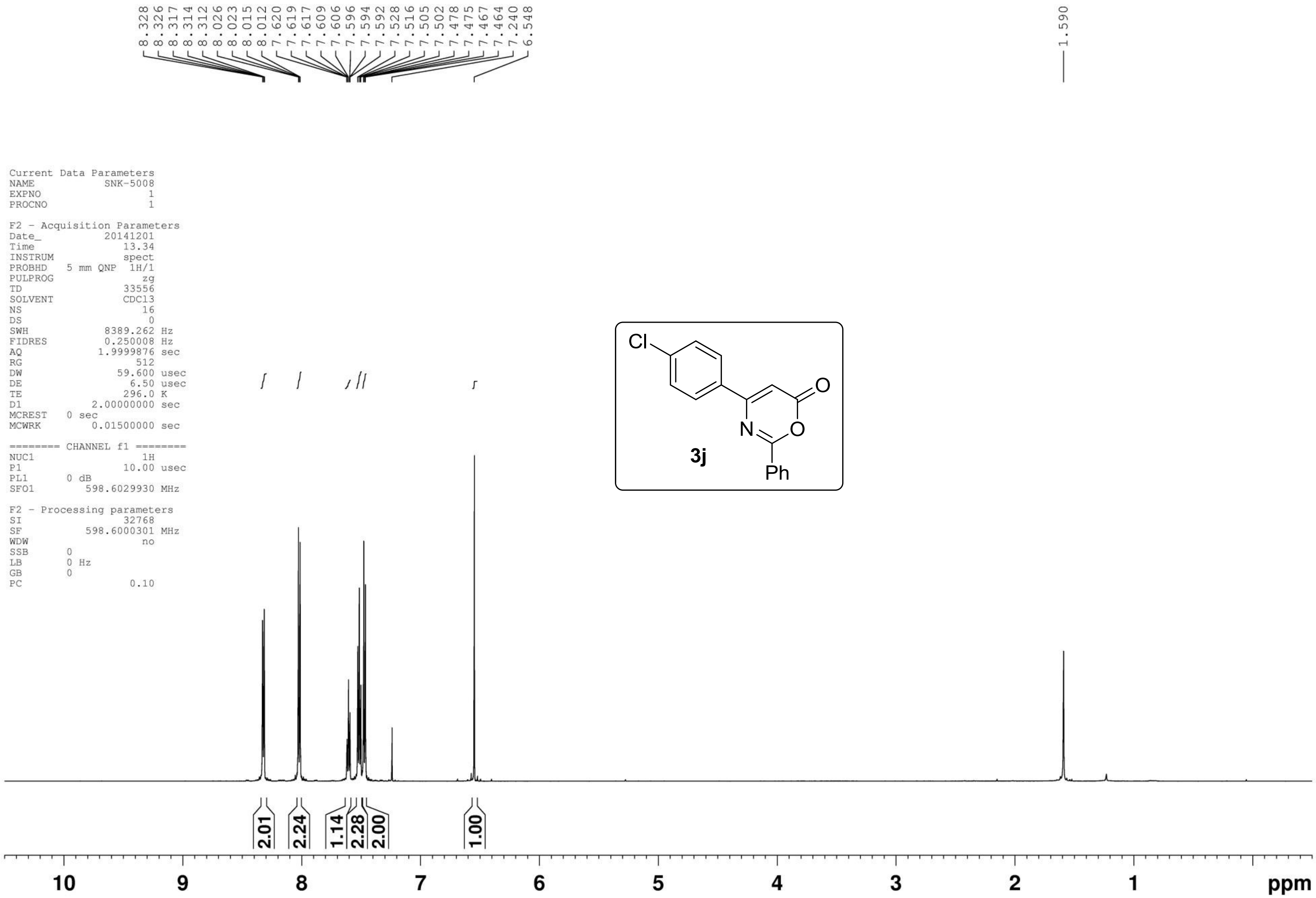
8.328
8.326
8.317
8.314
8.312
8.026
8.023
8.015
8.012
7.620
7.619
7.617
7.609
7.606
7.596
7.594
7.592
7.528
7.516
7.505
7.502
7.478
7.475
7.467
7.464
7.240
6.548
1.590
Current Data Parameters
NAME SNK-5008
EXPNO 1
PROCNO 1
F2 - Acquisition Parameters
Date_ 20141201
Time 13.34
INSTRUM spect
PROBHD 5 mm QNP 1H/1
PULPROG zg
TD 33556
SOLVENT CDCl3
NS 16
DS 0
SWH 8389.262 Hz
FIDRES 0.250008 Hz
AQ 1.9999876 sec
RG 512
DW 59.600 usec
DE 6.50 usec
TE 296.0 K
D1 2.00000000 sec
MCREST 0 sec
MCWRK 0.01500000 sec
======== CHANNEL f1 ========
NUC1 1H
P1 10.00 usec
PL1 0 dB
SFO1 598.6029930 MHz
F2 - Processing parameters
SI 32768
SF 598.6000301 MHz
WDW no
SSB 0
LB 0 Hz
GB 0
PC 0.10
Cl
O
N
O
3j
Ph
2.01
2.24
1.14
2.28
2.00
1.00
10
9
8
7
6
5
4
3
2
1
ppm

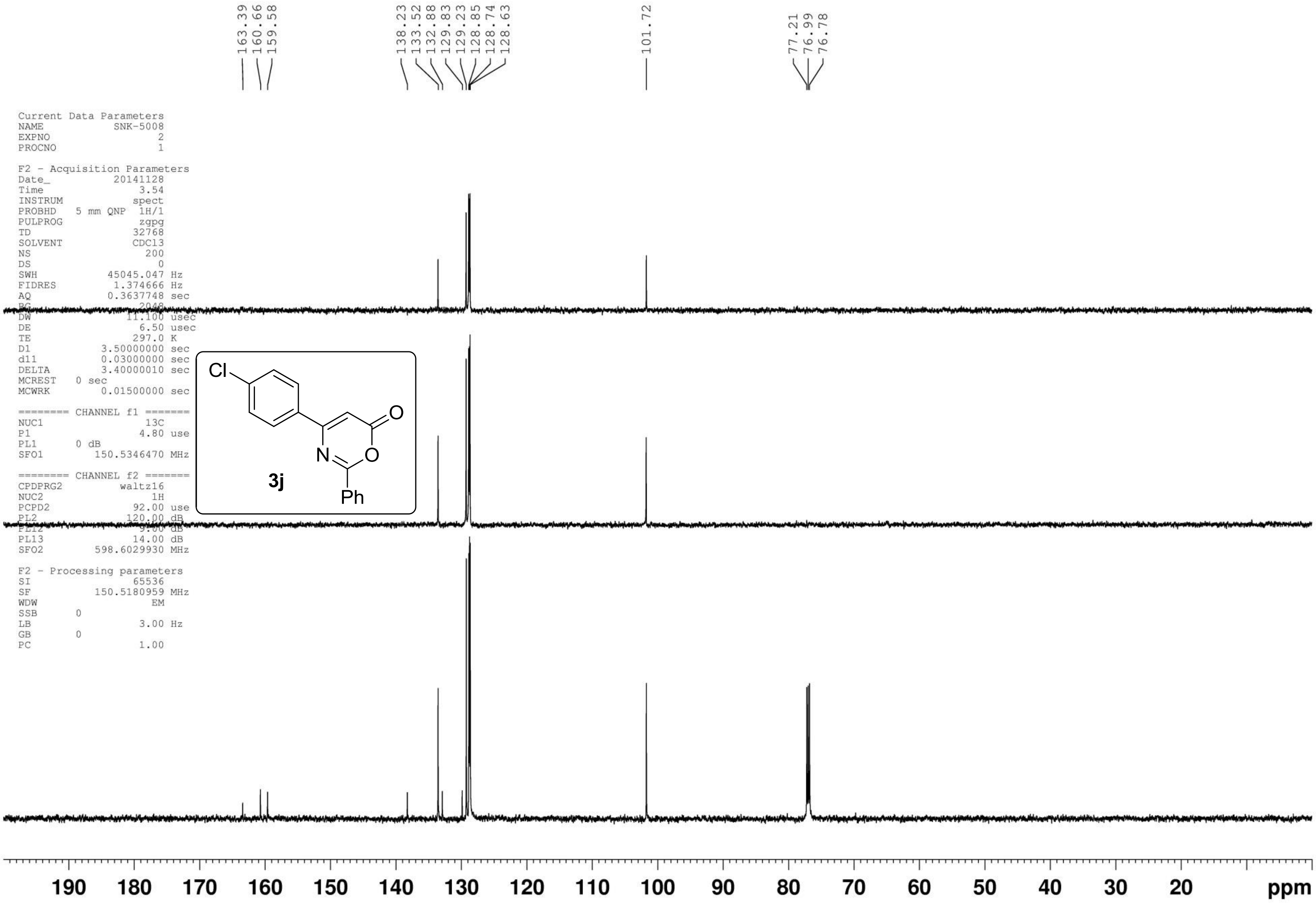
163.39
160.66
159.58
138.23
133.52
132.88
129.83
129.23
128.85
128.74
128.63
101.72
77.21
76.99
76.78
Current Data Parameters
NAME SNK-5008
EXPNO 2
PROCNO 1
F2 - Acquisition Parameters
Date_ 20141128
Time 3.54
INSTRUM spect
PROBHD 5 mm QNP 1H/1
PULPROG zgpg
TD 32768
SOLVENT CDCl3
NS 200
DS 0
SWH 45045.047 Hz
FIDRES 1.374666 Hz
AQ 0.3637748 sec
DW 11.100 usec
DE 6.50 usec
TE 297.0 K
D1 3.50000000 sec
d11 0.03000000 sec
DELTA 3.40000010 sec
MCREST 0 sec
MCWRK 0.01500000 sec
======== CHANNEL f1 ========
NUC1 13C
P1 4.80 use
PL1 0 dB
SFO1 150.5346470 MHz
======== CHANNEL f2 ========
CPDPRG2 waltz16
NUC2 1H
PCPD2 92.00 use
PL2 120.00 dB
PL13 14.00 dB
SFO2 598.6029930 MHz
F2 - Processing parameters
SI 65536
SF 150.5180959 MHz
WDW EM
SSB 0
LB 3.00 Hz
GB 0
PC 1.00
Cl
O
N
O
3j
Ph
190 180 170 160 150 140 130 120 110 100 90 80 70 60 50 40 30 20 ppm

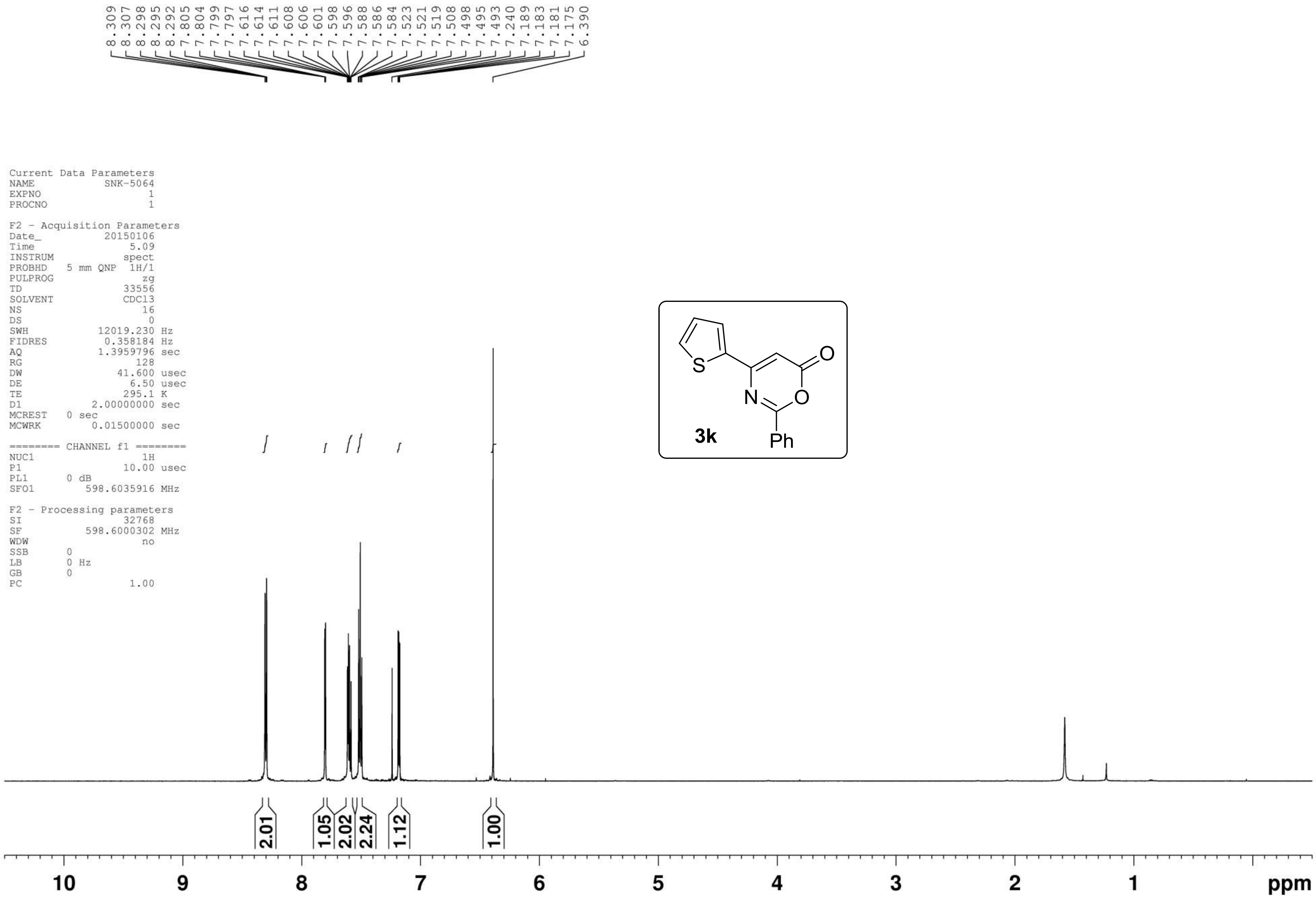
8.309
8.307
8.298
8.295
8.292
7.805
7.804
7.799
7.797
7.616
7.614
7.611
7.608
7.606
7.601
7.598
7.596
7.588
7.586
7.584
7.523
7.521
7.519
7.508
7.498
7.495
7.493
7.240
7.189
7.183
7.181
7.175
6.390
Current Data Parameters
NAME SNK-5064
EXPNO 1
PROCNO 1
F2 - Acquisition Parameters
Date_ 20150106
Time 5.09
INSTRUM spect
PROBHD 5 mm QNP 1H/1
PULPROG zg
TD 33556
SOLVENT CDCl3
NS 16
DS 0
SWH 12019.230 Hz
FIDRES 0.358184 Hz
AQ 1.3959796 sec
RG 128
DW 41.600 usec
DE 6.50 usec
TE 295.1 K
D1 2.00000000 sec
MCREST 0 sec
MCWRK 0.01500000 sec
======== CHANNEL f1 ========
NUC1 1H
P1 10.00 usec
PL1 0 dB
SFO1 598.6035916 MHz
F2 - Processing parameters
SI 32768
SF 598.6000302 MHz
WDW no
SSB 0
LB 0 Hz
GB 0
PC 1.00
S
O
N
O
3k
Ph
2.01
1.05
2.02
2.24
1.12
1.00
10
9
8
7
6
5
4
3
2
1
ppm

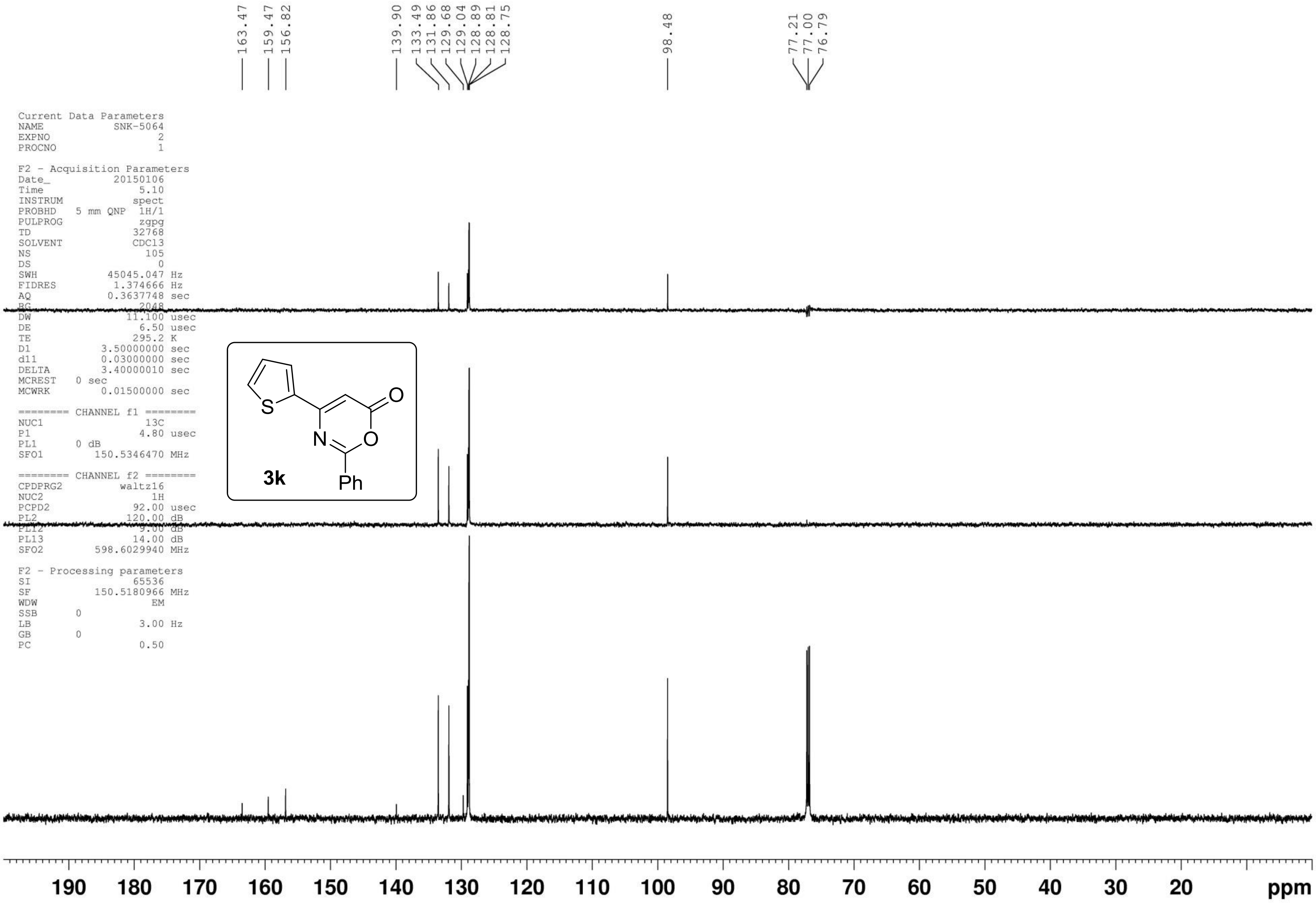
163.47
159.47
156.82
139.90
133.49
131.86
129.68
129.04
128.89
128.81
128.75
98.48
77.21
77.00
76.79
Current Data Parameters
NAME SNK-5064
EXPNO 2
PROCNO 1
F2 - Acquisition Parameters
Date_ 20150106
Time 5.10
INSTRUM spect
PROBHD 5 mm QNP 1H/1
PULPROG zgpg
TD 32768
SOLVENT CDCl3
NS 105
DS 0
SWH 45045.047 Hz
FIDRES 1.374666 Hz
AQ 0.3637748 sec
RG 2048
DW 11.100 usec
DE 6.50 usec
TE 295.2 K
D1 3.50000000 sec
d11 0.03000000 sec
DELTA 3.40000010 sec
MCREST 0 sec
MCWRK 0.01500000 sec
======== CHANNEL f1 ========
NUC1 13C
P1 4.80 usec
PL1 0 dB
SFO1 150.5346470 MHz
======== CHANNEL f2 ========
CPDPRG2 waltz16
NUC2 1H
PCPD2 92.00 usec
PL2 120.00 dB
PL12 9.00 dB
PL13 14.00 dB
SFO2 598.6029940 MHz
F2 - Processing parameters
SI 65536
SF 150.5180966 MHz
WDW EM
SSB 0
LB 3.00 Hz
GB 0
PC 0.50
S
N
O
O
3k
Ph
190 180 170 160 150 140 130 120 110 100 90 80 70 60 50 40 30 20 ppm

Current Data Parameters
NAME SNK-5067
EXPNO 1
PROCNO 1

F2 - Acquisition Parameters
Date_ 20150116
Time 4.41
INSTRUM spect
PROBHD 5 mm QNP 1H/1
PULPROG zg
TD 33556
SOLVENT CDCl3
NS 16
DS 0
SWH 8389.262 Hz
FIDRES 0.250008 Hz
AQ 1.9999876 sec
RG 128
DW 59.600 usec
DE 6.50 usec
TE 295.5 K
D1 2.00000000 sec
MCREST 0 sec
MCWRK 0.01500000 sec

======== CHANNEL f1 ========
NUC1 1H
P1 10.00 usec
PL1 0 dB
SFO1 598.6029930 MHz

F2 - Processing parameters
SI 32768
SF 598.6000301 MHz
WDW no
SSB 0
LB 0 Hz
GB 0
PC 1.00

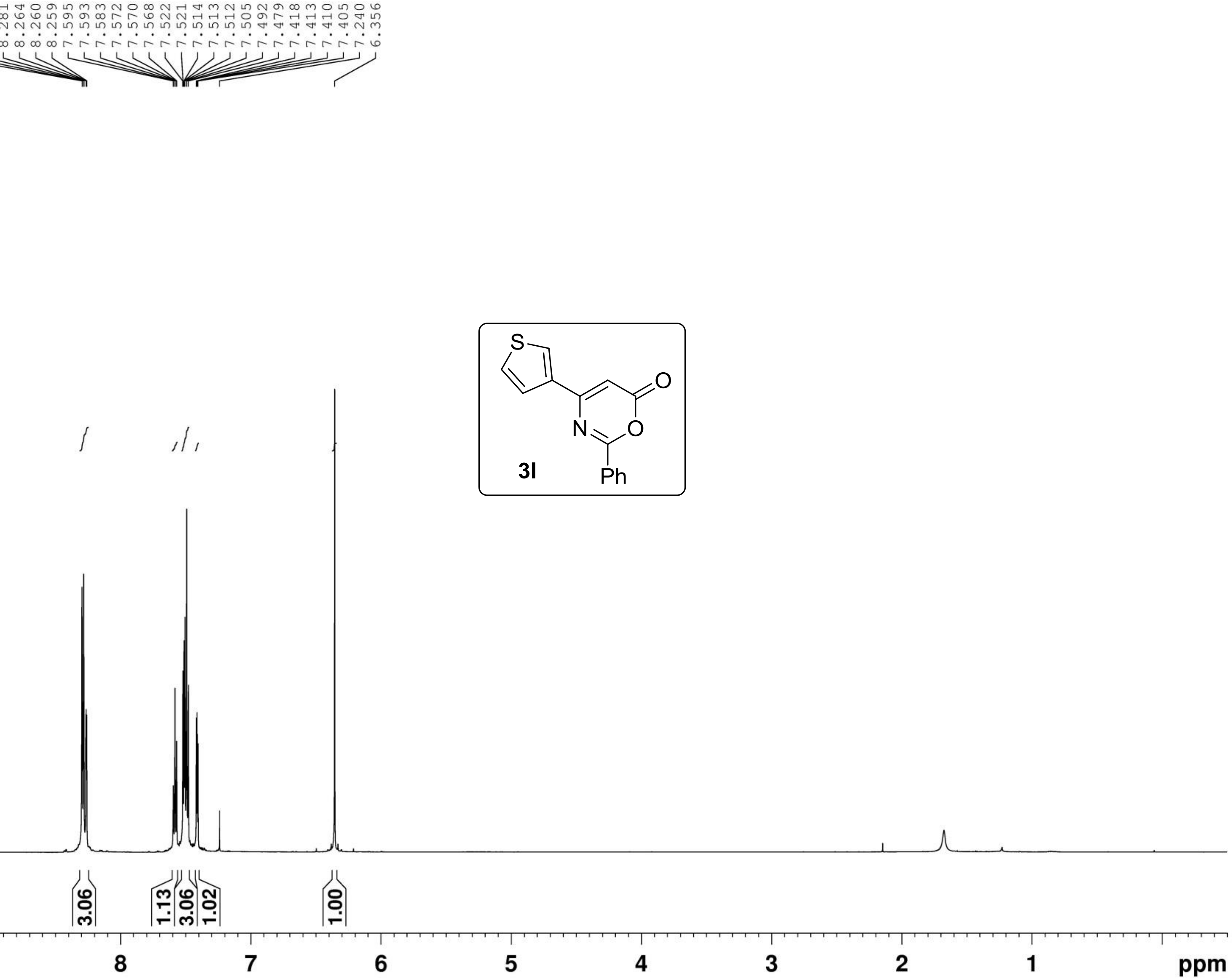

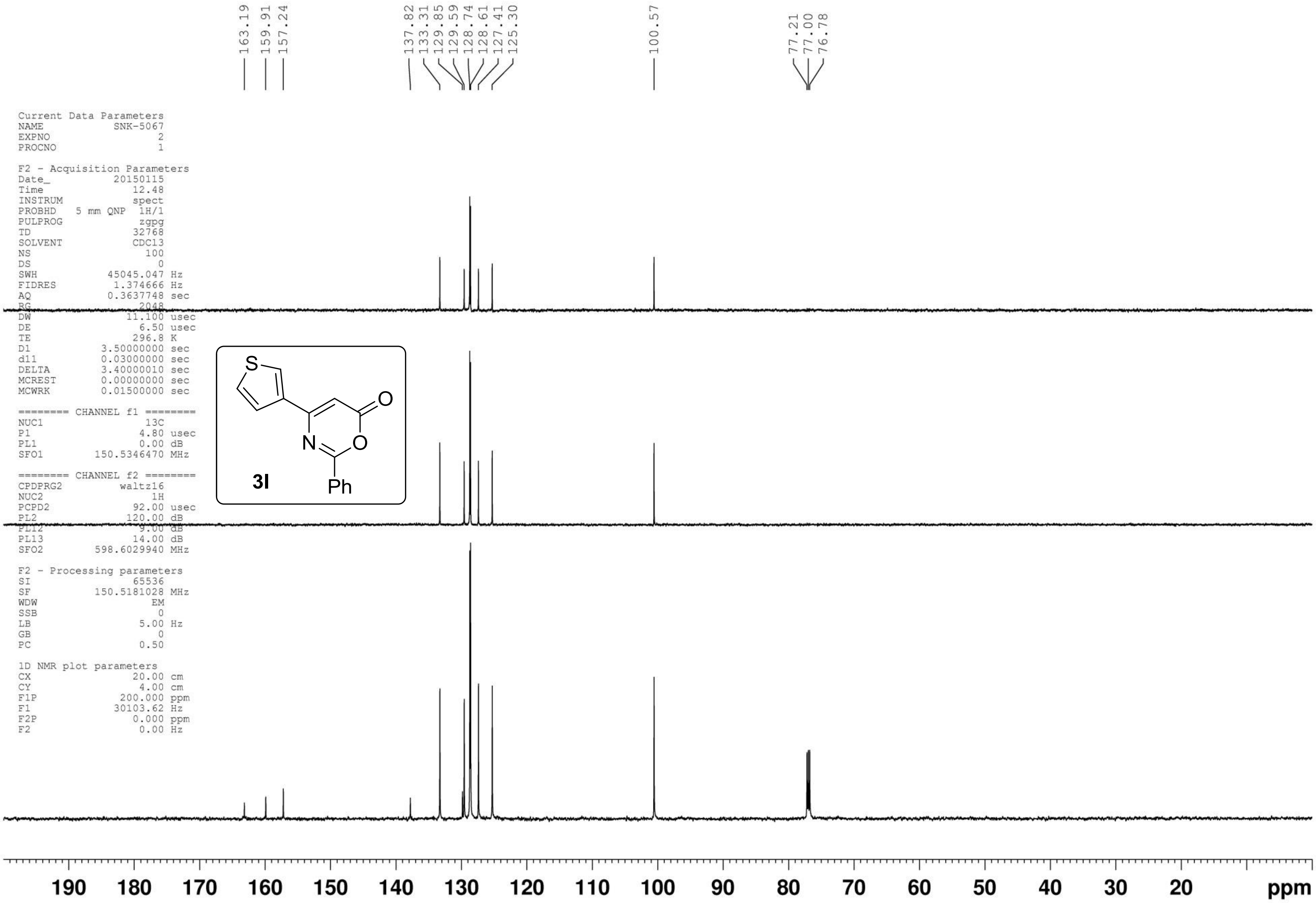
163.19
159.91
157.24
137.82
133.31
129.85
129.59
128.74
128.61
127.41
125.30
100.57
77.21
77.00
76.78
Current Data Parameters
NAME SNK-5067
EXPNO 2
PROCNO 1
F2 - Acquisition Parameters
Date_ 20150115
Time 12.48
INSTRUM spect
PROBHD 5 mm QNP 1H/1
PULPROG zgpg
TD 32768
SOLVENT CDCl3
NS 100
DS 0
SWH 45045.047 Hz
FIDRES 1.374666 Hz
AQ 0.3637748 sec
RG 2048
DW 11.100 usec
DE 6.50 usec
TE 296.8 K
D1 3.50000000 sec
d11 0.03000000 sec
DELTA 3.40000010 sec
MCREST 0.00000000 sec
MCWRK 0.01500000 sec
======== CHANNEL f1 ========
NUC1 13C
P1 4.80 usec
PL1 0.00 dB
SFO1 150.5346470 MHz
======== CHANNEL f2 ========
CPDPRG2 waltz16
NUC2 1H
PCPD2 92.00 usec
PL2 120.00 dB
PL12 9.00 dB
PL13 14.00 dB
SFO2 598.6029940 MHz
F2 - Processing parameters
SI 65536
SF 150.5181028 MHz
WDW EM
SSB 0
LB 5.00 Hz
GB 0
PC 0.50
1D NMR plot parameters
CX 20.00 cm
CY 4.00 cm
F1P 200.000 ppm
F1 30103.62 Hz
F2P 0.000 ppm
F2 0.00 Hz
190 180 170 160 150 140 130 120 110 100 90 80 70 60 50 40 30 20 ppm

3l

```
Current Data Parameters
NAME          SNK-5200A
EXPNO                 1
PROCNO                1

F2 - Acquisition Parameters
Date_          20150306
Time               2.51
INSTRUM           spect
PROBHD   5 mm QNP  1H/1
PULPROG              zg
TD                33556
SOLVENT           CDCl3
NS                   16
DS                    0
SWH            8389.262 Hz
FIDRES         0.250008 Hz
AQ            1.9999876 sec
RG                  128
DW               59.600 usec
DE                 6.50 usec
TE                294.9 K
D1           2.00000000 sec
MCREST   0 sec
MCWRK        0.01500000 sec

======== CHANNEL f1 ========
NUC1                 1H
P1                10.00 usec
PL1      0 dB
SFO1        598.6032923 MHz

F2 - Processing parameters
SI                32768
SF          598.6000283 MHz
WDW                  no
SSB      0
LB       0 Hz
GB       0
PC                 1.00
```

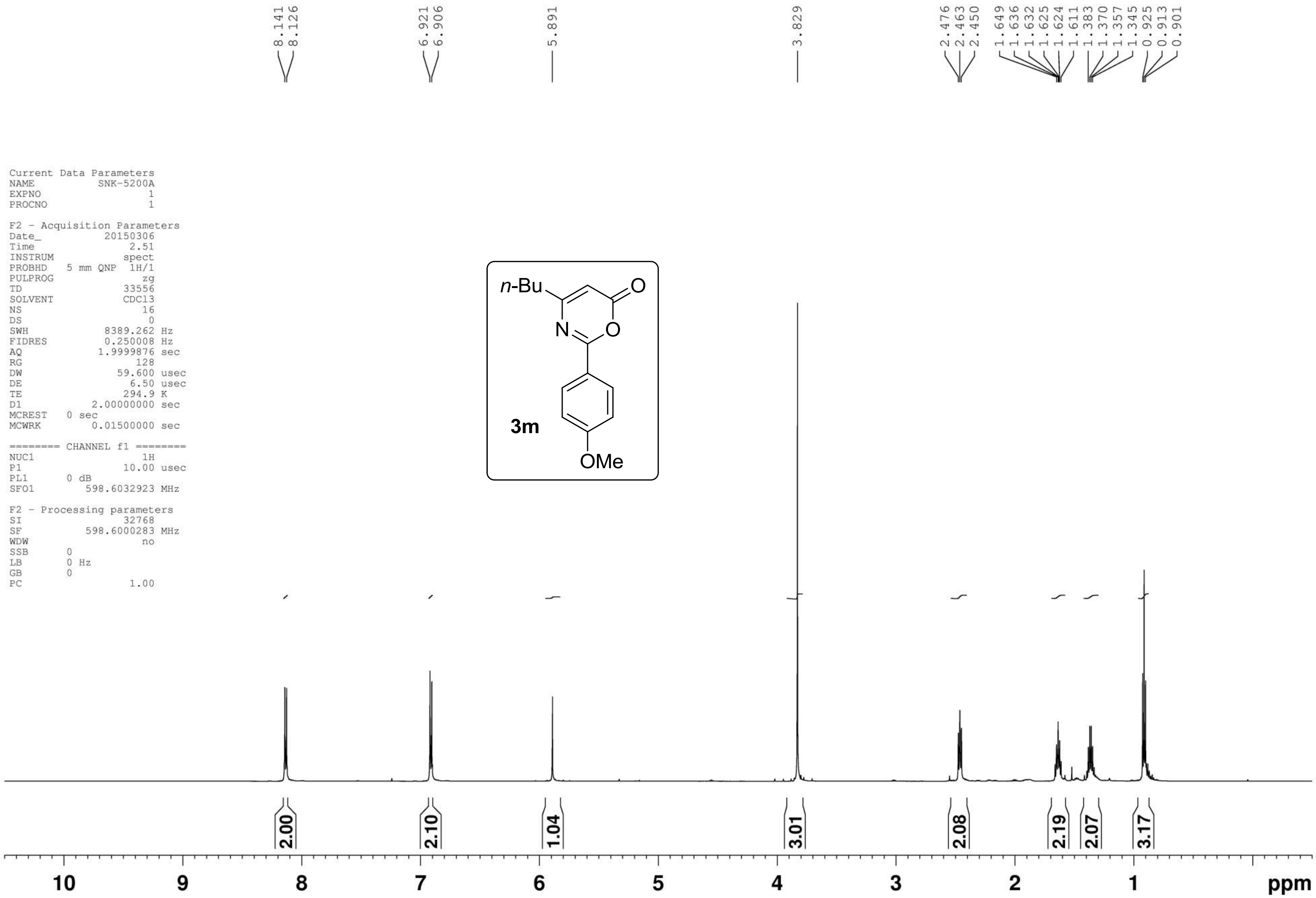

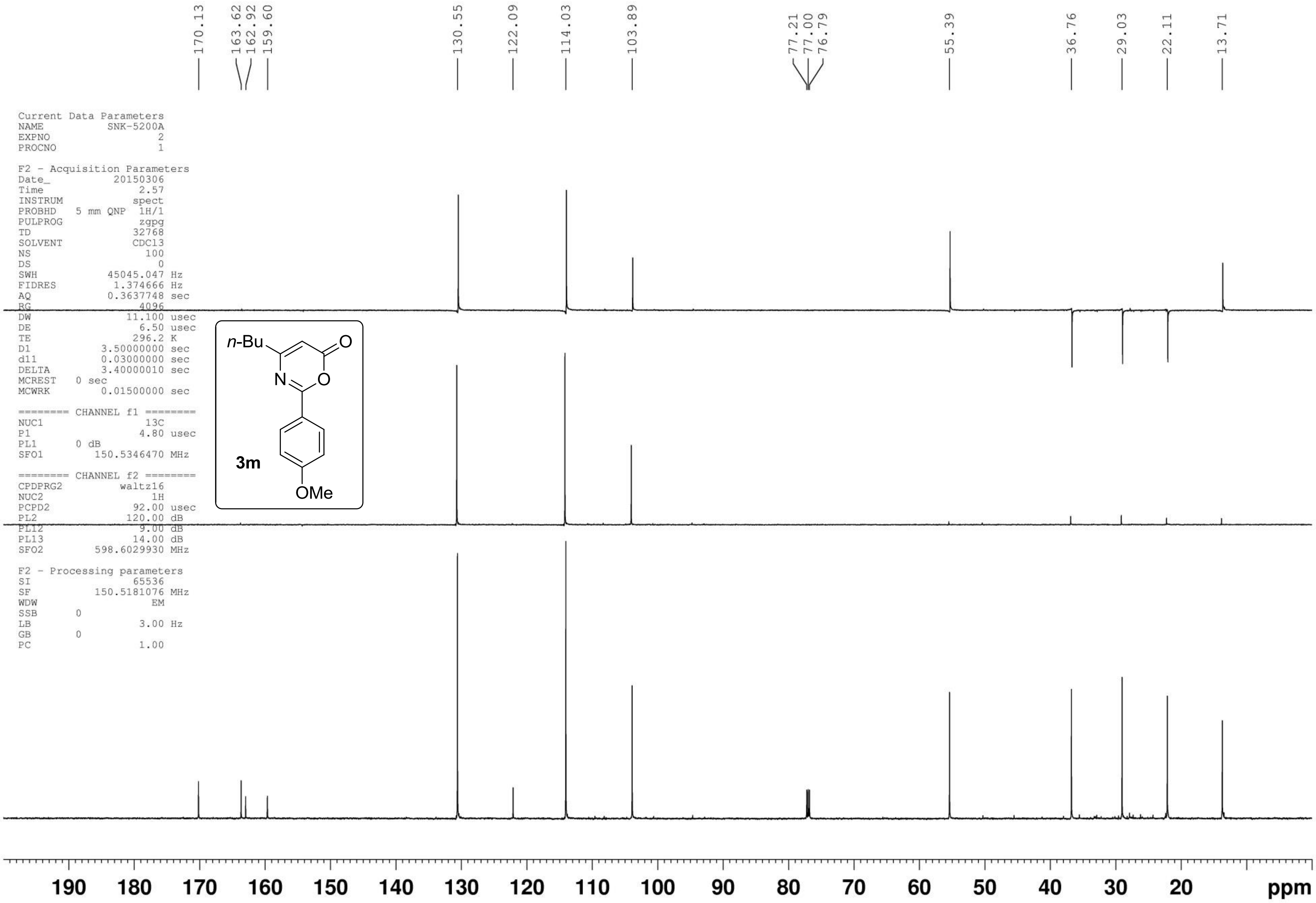
170.13
163.62
162.92
159.60
130.55
122.09
114.03
103.89
77.21
77.00
76.79
55.39
36.76
29.03
22.11
13.71
Current Data Parameters
NAME SNK-5200A
EXPNO 2
PROCNO 1
F2 - Acquisition Parameters
Date_ 20150306
Time 2.57
INSTRUM spect
PROBHD 5 mm QNP 1H/1
PULPROG zgpg
TD 32768
SOLVENT CDCl3
NS 100
DS 0
SWH 45045.047 Hz
FIDRES 1.374666 Hz
AQ 0.3637748 sec
RG 4096
DW 11.100 usec
DE 6.50 usec
TE 296.2 K
D1 3.50000000 sec
d11 0.03000000 sec
DELTA 3.40000010 sec
MCREST 0 sec
MCWRK 0.01500000 sec
======== CHANNEL f1 ========
NUC1 13C
P1 4.80 usec
PL1 0 dB
SFO1 150.5346470 MHz
======== CHANNEL f2 ========
CPDPRG2 waltz16
NUC2 1H
PCPD2 92.00 usec
PL2 120.00 dB
PL12 9.00 dB
PL13 14.00 dB
SFO2 598.6029930 MHz
F2 - Processing parameters
SI 65536
SF 150.5181076 MHz
WDW EM
SSB 0
LB 3.00 Hz
GB 0
PC 1.00
n-Bu
O
N
O
OMe
3m
190
180
170
160
150
140
130
120
110
100
90
80
70
60
50
40
30
20
ppm

```
Current Data Parameters
NAME          SNK-5200G
EXPNO                 1
PROCNO                1

F2 - Acquisition Parameters
Date_          20150310
Time               3.54
INSTRUM           spect
PROBHD   5 mm QNP  1H/1
PULPROG              zg
TD                32768
SOLVENT           CDCl3
NS                   16
DS                    0
SWH            8389.262 Hz
FIDRES         0.256020 Hz
AQ            1.9530228 sec
RG                   32
DW               59.600 usec
DE                 6.50 usec
TE                295.1 K
D1           1.50000000 sec
MCREST   0 sec
MCWRK        0.01500000 sec

======== CHANNEL f1 ========
NUC1                 1H
P1                10.00 usec
PL1      0 dB
SFO1        598.6029930 MHz

F2 - Processing parameters
SI                32768
SF          598.6000250 MHz
WDW                  no
SSB      0
LB       0 Hz
GB       0
PC                 1.00
```

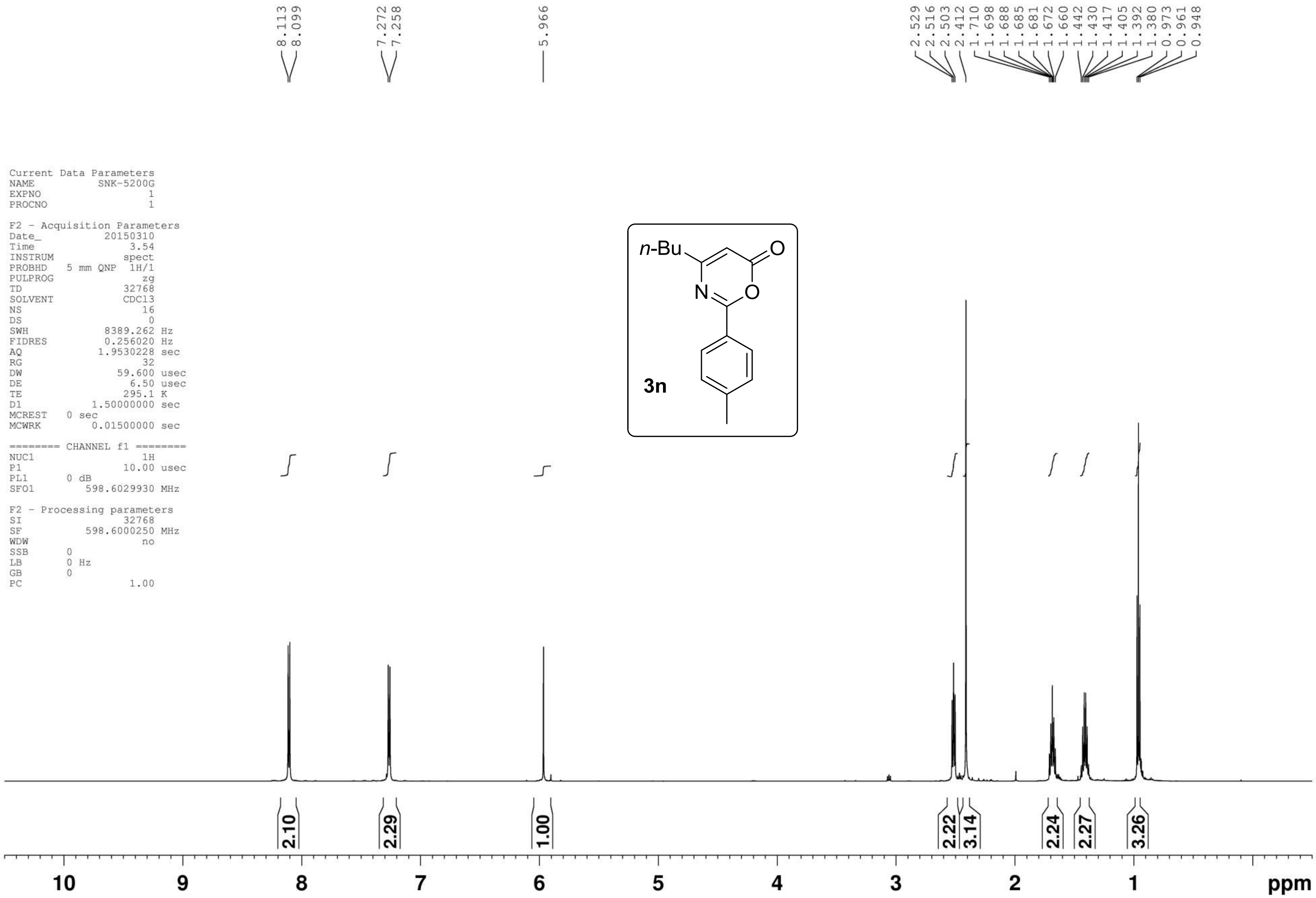

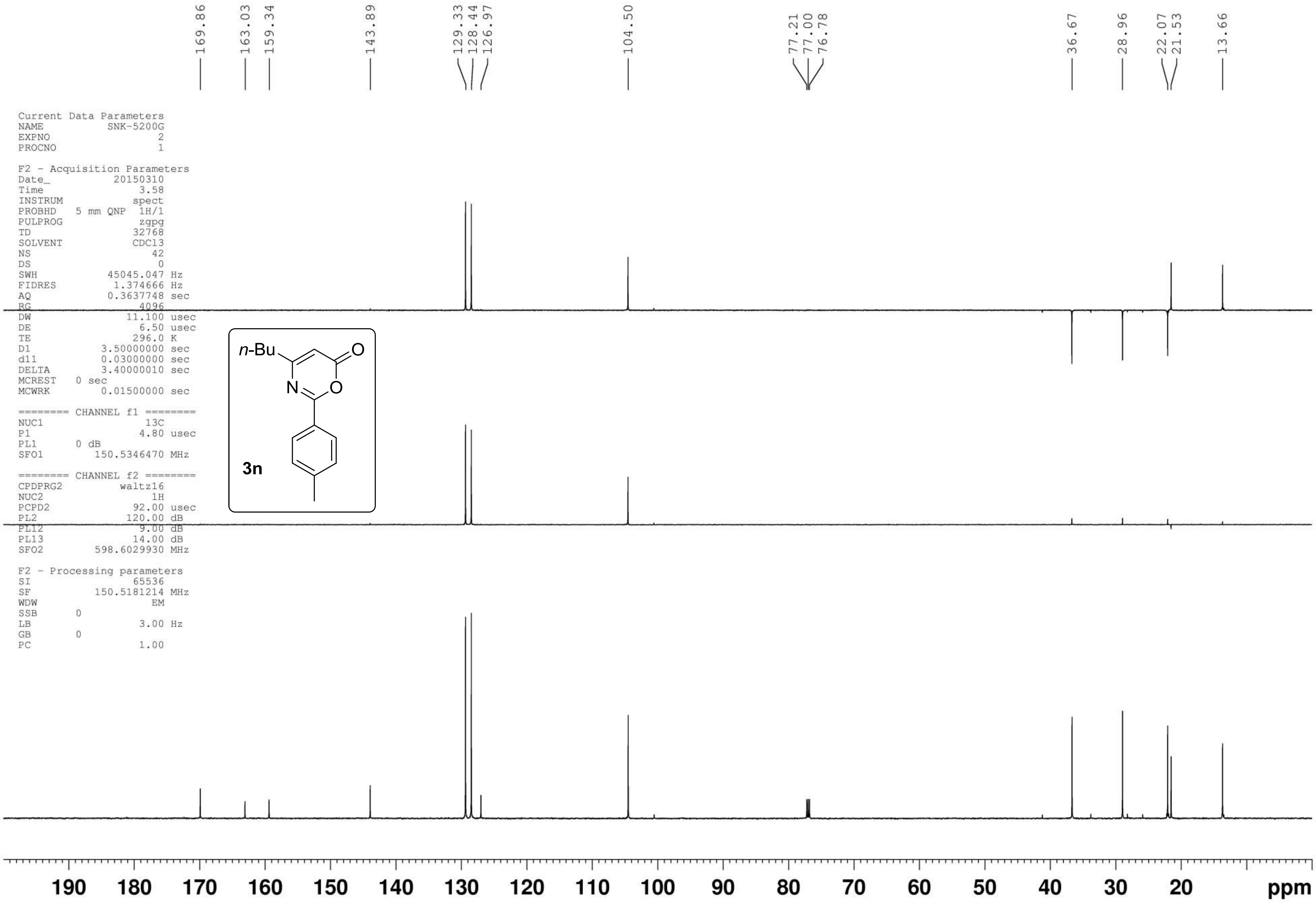
169.86
163.03
159.34
143.89
129.33
128.44
126.97
104.50
77.21
77.00
76.78
36.67
28.96
22.07
21.53
13.66
Current Data Parameters
NAME SNK-5200G
EXPNO 2
PROCNO 1
F2 - Acquisition Parameters
Date_ 20150310
Time 3.58
INSTRUM spect
PROBHD 5 mm QNP 1H/1
PULPROG zgpg
TD 32768
SOLVENT CDCl3
NS 42
DS 0
SWH 45045.047 Hz
FIDRES 1.374666 Hz
AQ 0.3637748 sec
RG 4096
DW 11.100 usec
DE 6.50 usec
TE 296.0 K
D1 3.50000000 sec
d11 0.03000000 sec
DELTA 3.40000010 sec
MCREST 0 sec
MCWRK 0.01500000 sec
======== CHANNEL f1 ========
NUC1 13C
P1 4.80 usec
PL1 0 dB
SFO1 150.5346470 MHz
======== CHANNEL f2 ========
CPDPRG2 waltz16
NUC2 1H
PCPD2 92.00 usec
PL2 120.00 dB
PL12 9.00 dB
PL13 14.00 dB
SFO2 598.6029930 MHz
F2 - Processing parameters
SI 65536
SF 150.5181214 MHz
WDW EM
SSB 0
LB 3.00 Hz
GB 0
PC 1.00
n-Bu
O
N
O
3n
190
180
170
160
150
140
130
120
110
100
90
80
70
60
50
40
30
20
ppm

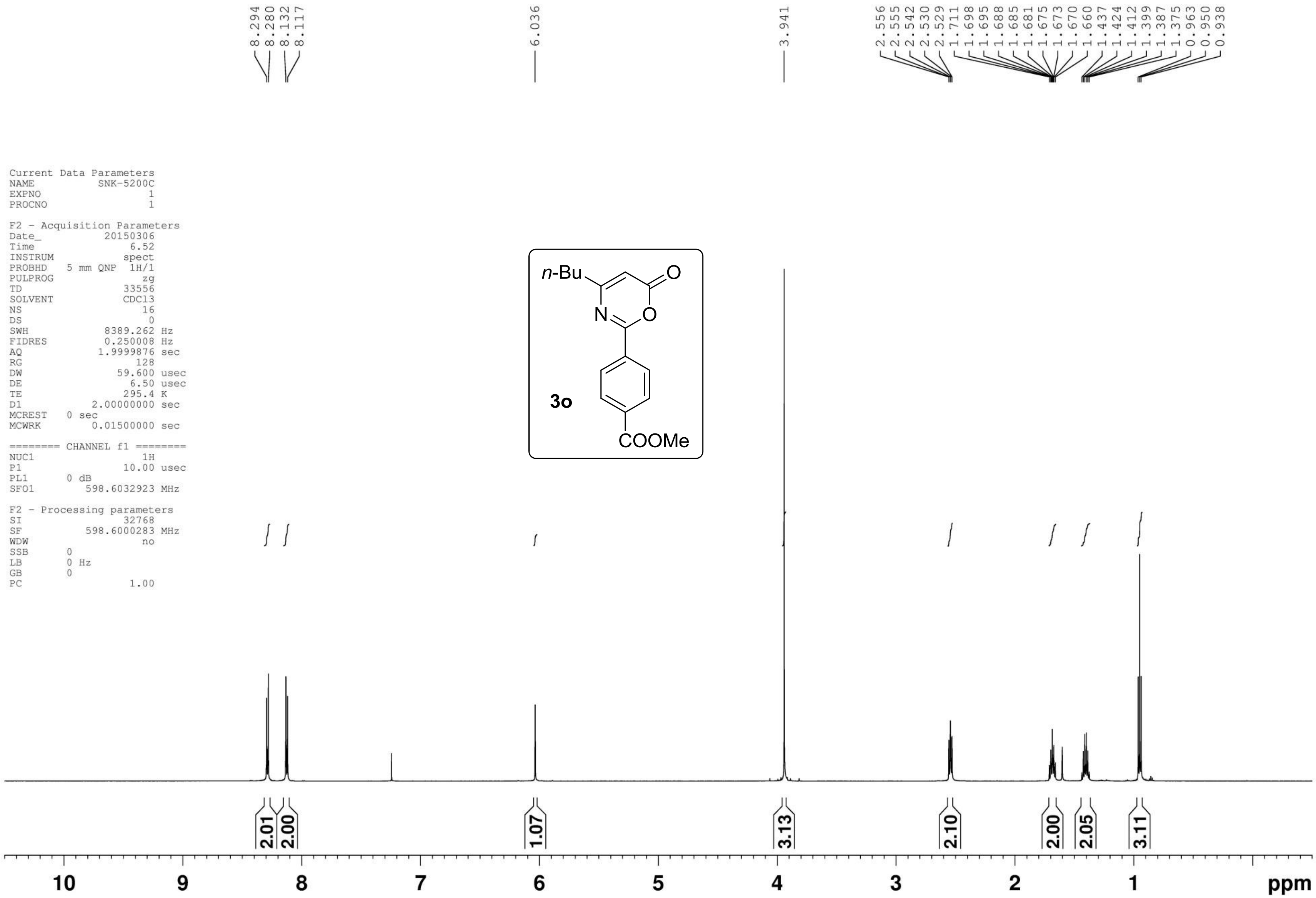
8.294
8.280
8.132
8.117
6.036
3.941
2.556
2.555
2.542
2.530
2.529
1.711
1.698
1.695
1.688
1.685
1.681
1.675
1.673
1.670
1.660
1.437
1.424
1.412
1.399
1.387
1.375
0.963
0.950
0.938
Current Data Parameters
NAME SNK-5200C
EXPNO 1
PROCNO 1
F2 - Acquisition Parameters
Date_ 20150306
Time 6.52
INSTRUM spect
PROBHD 5 mm QNP 1H/1
PULPROG zg
TD 33556
SOLVENT CDCl3
NS 16
DS 0
SWH 8389.262 Hz
FIDRES 0.250008 Hz
AQ 1.9999876 sec
RG 128
DW 59.600 usec
DE 6.50 usec
TE 295.4 K
D1 2.00000000 sec
MCREST 0 sec
MCWRK 0.01500000 sec
======== CHANNEL f1 ========
NUC1 1H
P1 10.00 usec
PL1 0 dB
SFO1 598.6032923 MHz
F2 - Processing parameters
SI 32768
SF 598.6000283 MHz
WDW no
SSB 0
LB 0 Hz
GB 0
PC 1.00
n-Bu
O
N
O
3o
COOMe
2.01
2.00
1.07
3.13
2.10
2.00
2.05
3.11
10
9
8
7
6
5
4
3
2
1
ppm

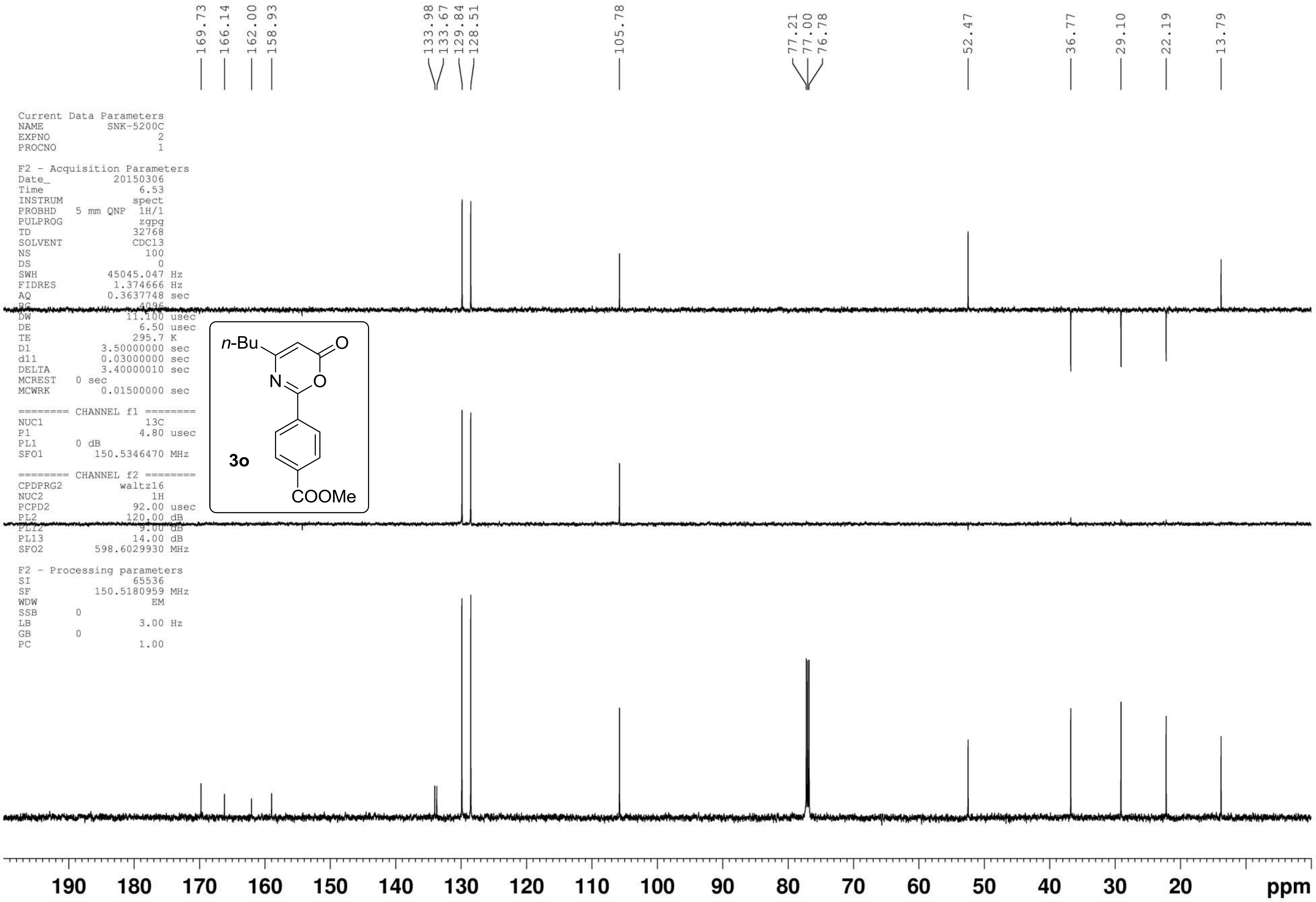
169.73
166.14
162.00
158.93
133.98
133.67
129.84
128.51
105.78
77.21
77.00
76.78
52.47
36.77
29.10
22.19
13.79
Current Data Parameters
NAME SNK-5200C
EXPNO 2
PROCNO 1
F2 - Acquisition Parameters
Date_ 20150306
Time 6.53
INSTRUM spect
PROBHD 5 mm QNP 1H/1
PULPROG zgpg
TD 32768
SOLVENT CDCl3
NS 100
DS 0
SWH 45045.047 Hz
FIDRES 1.374666 Hz
AQ 0.3637748 sec
DW 11.100 usec
DE 6.50 usec
TE 295.7 K
D1 3.50000000 sec
d11 0.03000000 sec
DELTA 3.40000010 sec
MCREST 0 sec
MCWRK 0.01500000 sec
======== CHANNEL f1 ========
NUC1 13C
P1 4.80 usec
PL1 0 dB
SFO1 150.5346470 MHz
======== CHANNEL f2 ========
CPDPRG2 waltz16
NUC2 1H
PCPD2 92.00 usec
PL2 120.00 dB
PL13 14.00 dB
SFO2 598.6029930 MHz
F2 - Processing parameters
SI 65536
SF 150.5180959 MHz
WDW EM
SSB 0
LB 3.00 Hz
GB 0
PC 1.00
n-Bu
O
N
O
3o
COOMe
190 180 170 160 150 140 130 120 110 100 90 80 70 60 50 40 30 20 ppm

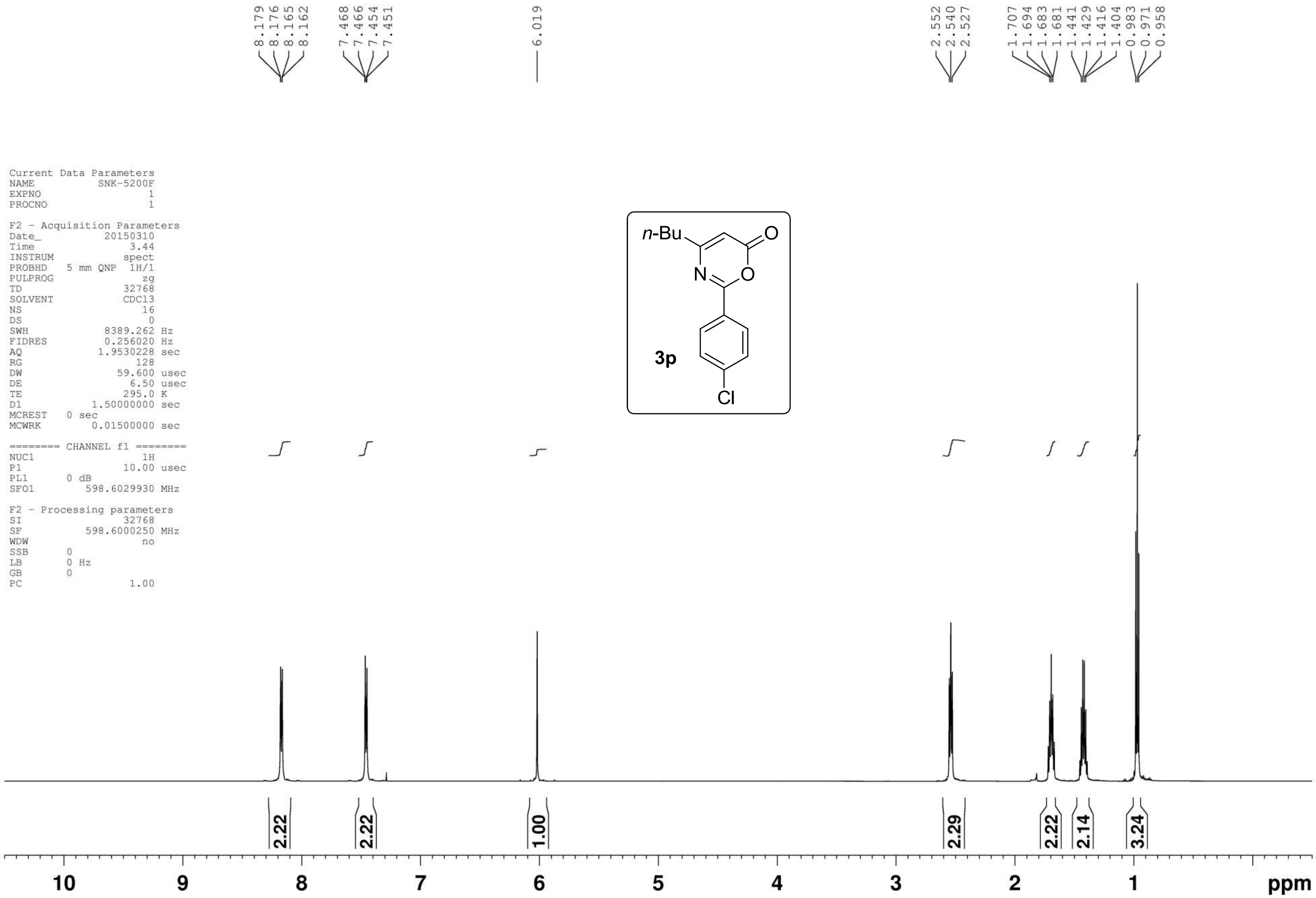
8.179
8.176
8.165
8.162
7.468
7.466
7.454
7.451
6.019
2.552
2.540
2.527
1.707
1.694
1.683
1.681
1.441
1.429
1.416
1.404
0.983
0.971
0.958
Current Data Parameters
NAME SNK-5200F
EXPNO 1
PROCNO 1
F2 - Acquisition Parameters
Date_ 20150310
Time 3.44
INSTRUM spect
PROBHD 5 mm QNP 1H/1
PULPROG zg
TD 32768
SOLVENT CDCl3
NS 16
DS 0
SWH 8389.262 Hz
FIDRES 0.256020 Hz
AQ 1.9530228 sec
RG 128
DW 59.600 usec
DE 6.50 usec
TE 295.0 K
D1 1.50000000 sec
MCREST 0 sec
MCWRK 0.01500000 sec
======== CHANNEL f1 ========
NUC1 1H
P1 10.00 usec
PL1 0 dB
SFO1 598.6029930 MHz
F2 - Processing parameters
SI 32768
SF 598.6000250 MHz
WDW no
SSB 0
LB 0 Hz
GB 0
PC 1.00
n-Bu
O
N
O
3p
Cl
2.22
2.22
1.00
2.29
2.22
2.14
3.24
10
9
8
7
6
5
4
3
2
1
ppm

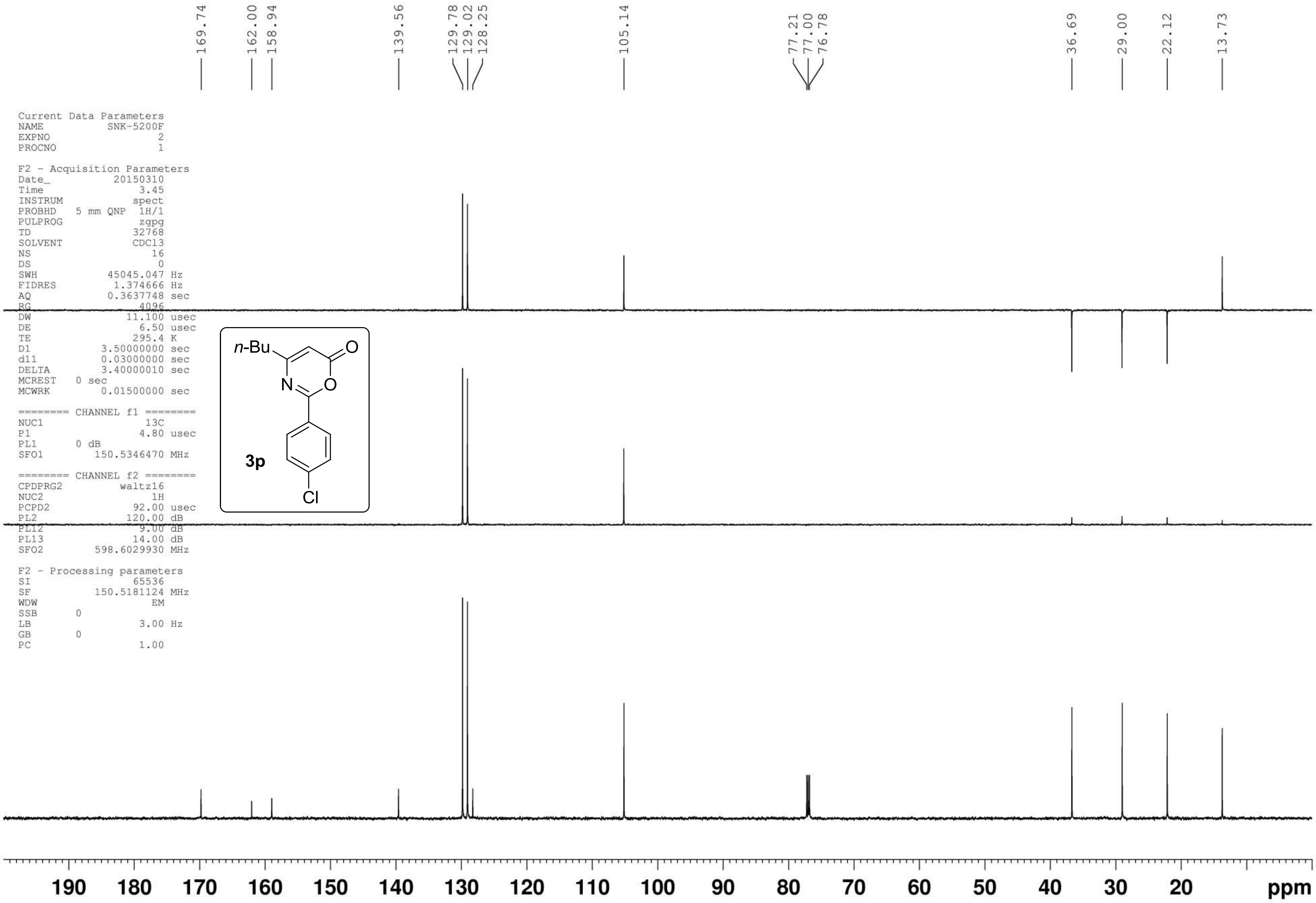
169.74
162.00
158.94
139.56
129.78
129.02
128.25
105.14
77.21
77.00
76.78
36.69
29.00
22.12
13.73
Current Data Parameters
NAME SNK-5200F
EXPNO 2
PROCNO 1
F2 - Acquisition Parameters
Date_ 20150310
Time 3.45
INSTRUM spect
PROBHD 5 mm QNP 1H/1
PULPROG zgpg
TD 32768
SOLVENT CDCl3
NS 16
DS 0
SWH 45045.047 Hz
FIDRES 1.374666 Hz
AQ 0.3637748 sec
RG 4096
DW 11.100 usec
DE 6.50 usec
TE 295.4 K
D1 3.50000000 sec
d11 0.03000000 sec
DELTA 3.40000010 sec
MCREST 0 sec
MCWRK 0.01500000 sec
======== CHANNEL f1 ========
NUC1 13C
P1 4.80 usec
PL1 0 dB
SFO1 150.5346470 MHz
======== CHANNEL f2 ========
CPDPRG2 waltz16
NUC2 1H
PCPD2 92.00 usec
PL2 120.00 dB
PL12 9.00 dB
PL13 14.00 dB
SFO2 598.6029930 MHz
F2 - Processing parameters
SI 65536
SF 150.5181124 MHz
WDW EM
SSB 0
LB 3.00 Hz
GB 0
PC 1.00
n-Bu
O
N
O
Cl
3p
190
180
170
160
150
140
130
120
110
100
90
80
70
60
50
40
30
20
ppm

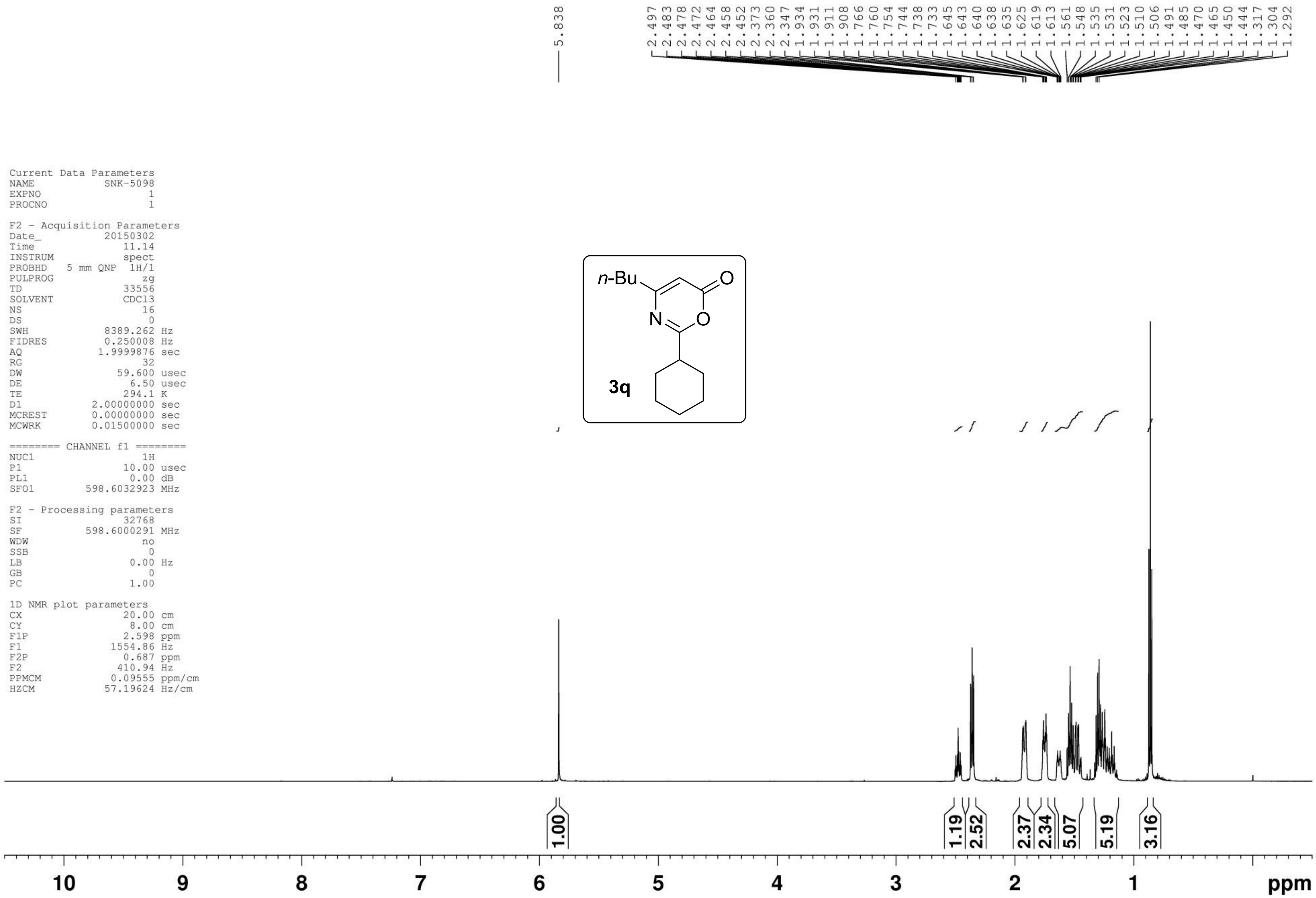
5.838
2.497 2.483 2.478 2.472 2.464 2.458 2.452 2.373 2.360 2.347 1.934 1.931 1.911 1.908 1.766 1.760 1.754 1.744 1.738 1.733 1.645 1.643 1.640 1.638 1.635 1.625 1.619 1.613 1.561 1.548 1.535 1.531 1.523 1.510 1.506 1.491 1.485 1.470 1.465 1.450 1.444 1.317 1.304 1.292
Current Data Parameters
NAME SNK-5098
EXPNO 1
PROCNO 1
F2 - Acquisition Parameters
Date_ 20150302
Time 11.14
INSTRUM spect
PROBHD 5 mm QNP 1H/1
PULPROG zg
TD 33556
SOLVENT CDCl3
NS 16
DS 0
SWH 8389.262 Hz
FIDRES 0.250008 Hz
AQ 1.9999876 sec
RG 32
DW 59.600 usec
DE 6.50 usec
TE 294.1 K
D1 2.00000000 sec
MCREST 0.00000000 sec
MCWRK 0.01500000 sec
======== CHANNEL f1 ========
NUC1 1H
P1 10.00 usec
PL1 0.00 dB
SFO1 598.6032923 MHz
F2 - Processing parameters
SI 32768
SF 598.6000291 MHz
WDW no
SSB 0
LB 0.00 Hz
GB 0
PC 1.00
1D NMR plot parameters
CX 20.00 cm
CY 8.00 cm
F1P 2.598 ppm
F1 1554.86 Hz
F2P 0.687 ppm
F2 410.94 Hz
PPMCM 0.09555 ppm/cm
HZCM 57.19624 Hz/cm
n-Bu
O
N
O
3q
1.00
1.19
2.52
2.37
2.34
5.07
5.19
3.16
10
9
8
7
6
5
4
3
2
1
ppm

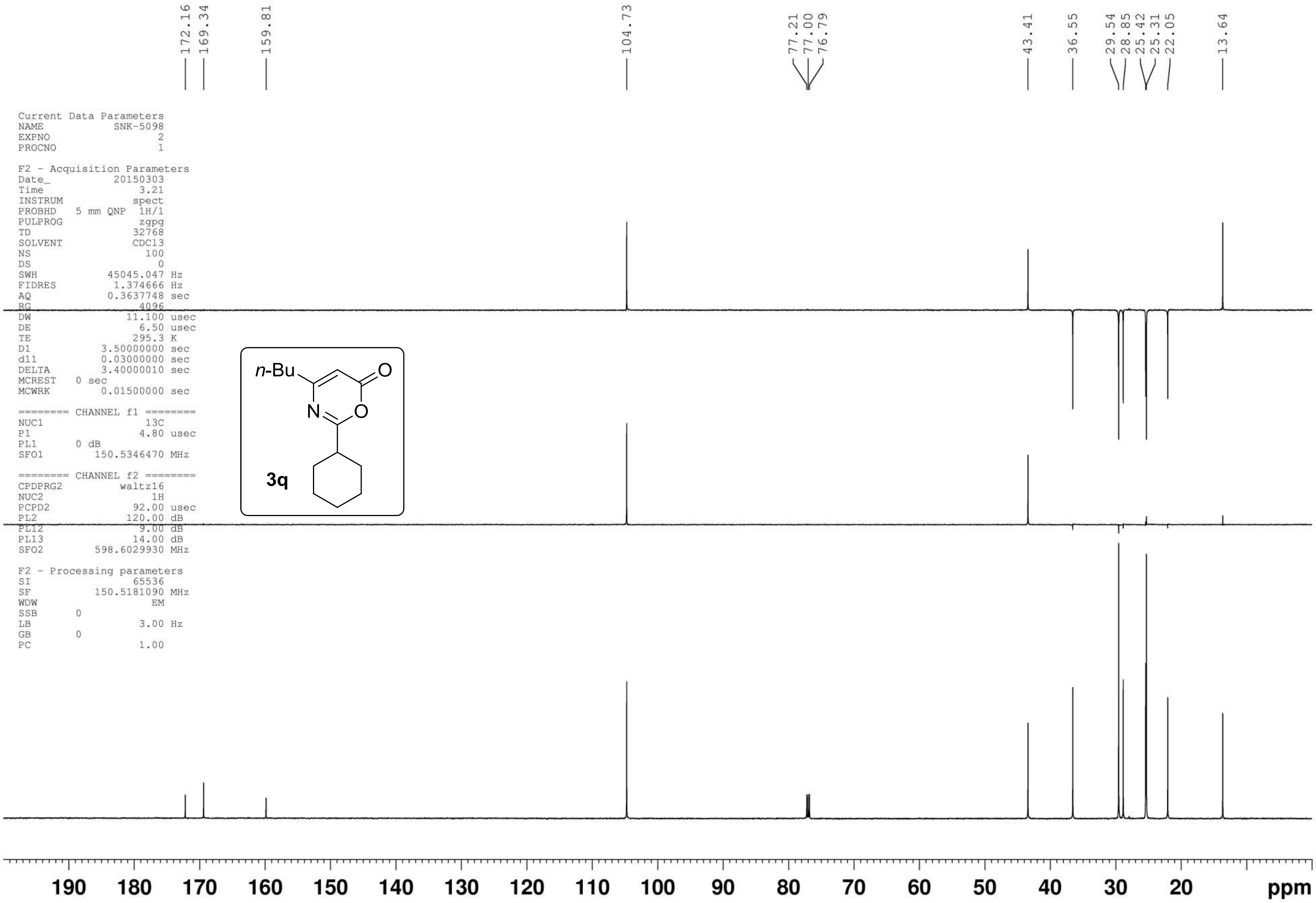

Current Data Parameters
NAME SNK-5098
EXPNO 2
PROCNO 1

F2 - Acquisition Parameters
Date_ 20150303
Time 3.21
INSTRUM spect
PROBHD 5 mm QNP 1H/1
PULPROG zgpg
TD 32768
SOLVENT CDCl3
NS 100
DS 0
SWH 45045.047 Hz
FIDRES 1.374666 Hz
AQ 0.3637748 sec
RG 4096
DW 11.100 usec
DE 6.50 usec
TE 295.3 K
D1 3.50000000 sec
d11 0.03000000 sec
DELTA 3.40000010 sec
MCREST 0 sec
MCWRK 0.01500000 sec

======== CHANNEL f1 ========
NUC1 13C
P1 4.80 usec
PL1 0 dB
SFO1 150.5346470 MHz

======== CHANNEL f2 ========
CPDPRG2 waltz16
NUC2 1H
PCPD2 92.00 usec
PL2 120.00 dB
PL12 9.00 dB
PL13 14.00 dB
SFO2 598.6029930 MHz

F2 - Processing parameters
SI 65536
SF 150.5181090 MHz
WDW EM
SSB 0
LB 3.00 Hz
GB 0
PC 1.00

7.815
7.788
7.530
7.529
7.522
7.516
7.513
7.372
7.370
7.364
7.361
6.681
6.654
5.912
2.454
2.453
2.441
2.428
2.428
1.640
1.627
1.624
1.618
1.615
1.610
1.604
1.602
1.599
1.589
1.583
1.394
1.382
1.369
1.356
1.344
1.332
0.929
0.916
0.904

Current Data Parameters
NAME SNK-5099
EXPNO 1
PROCNO 1

F2 - Acquisition Parameters
Date_ 20150304
Time 0.43
INSTRUM spect
PROBHD 5 mm QNP 1H/1
PULPROG zg
TD 33556
SOLVENT CDCl3
NS 16
DS 0
SWH 8389.262 Hz
FIDRES 0.250008 Hz
AQ 1.9999876 sec
RG 32
DW 59.600 usec
DE 6.50 usec
TE 295.1 K
D1 2.00000000 sec
MCREST 0 sec
MCWRK 0.01500000 sec

======== CHANNEL f1 ========
NUC1 1H
P1 10.00 usec
PL1 0 dB
SFO1 598.6032923 MHz

F2 - Processing parameters
SI 32768
SF 598.6000301 MHz
WDW no
SSB 0
LB 0 Hz
GB 0
PC 1.00

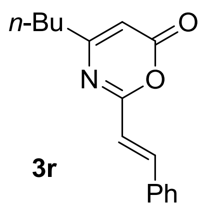

3r

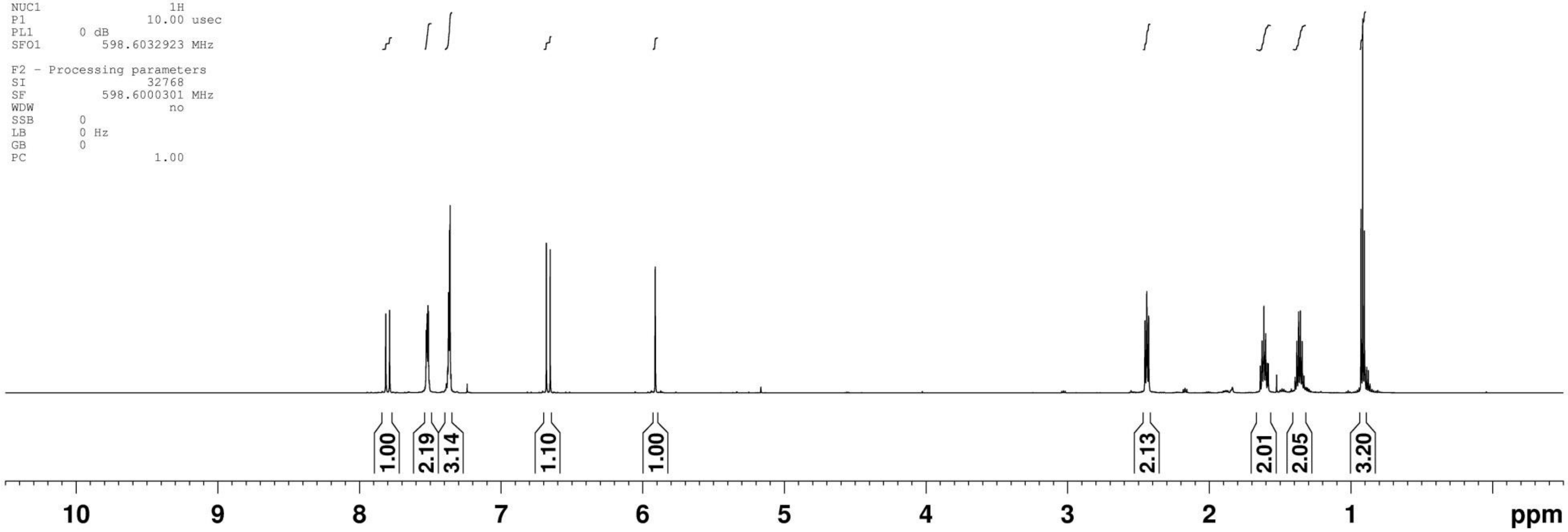

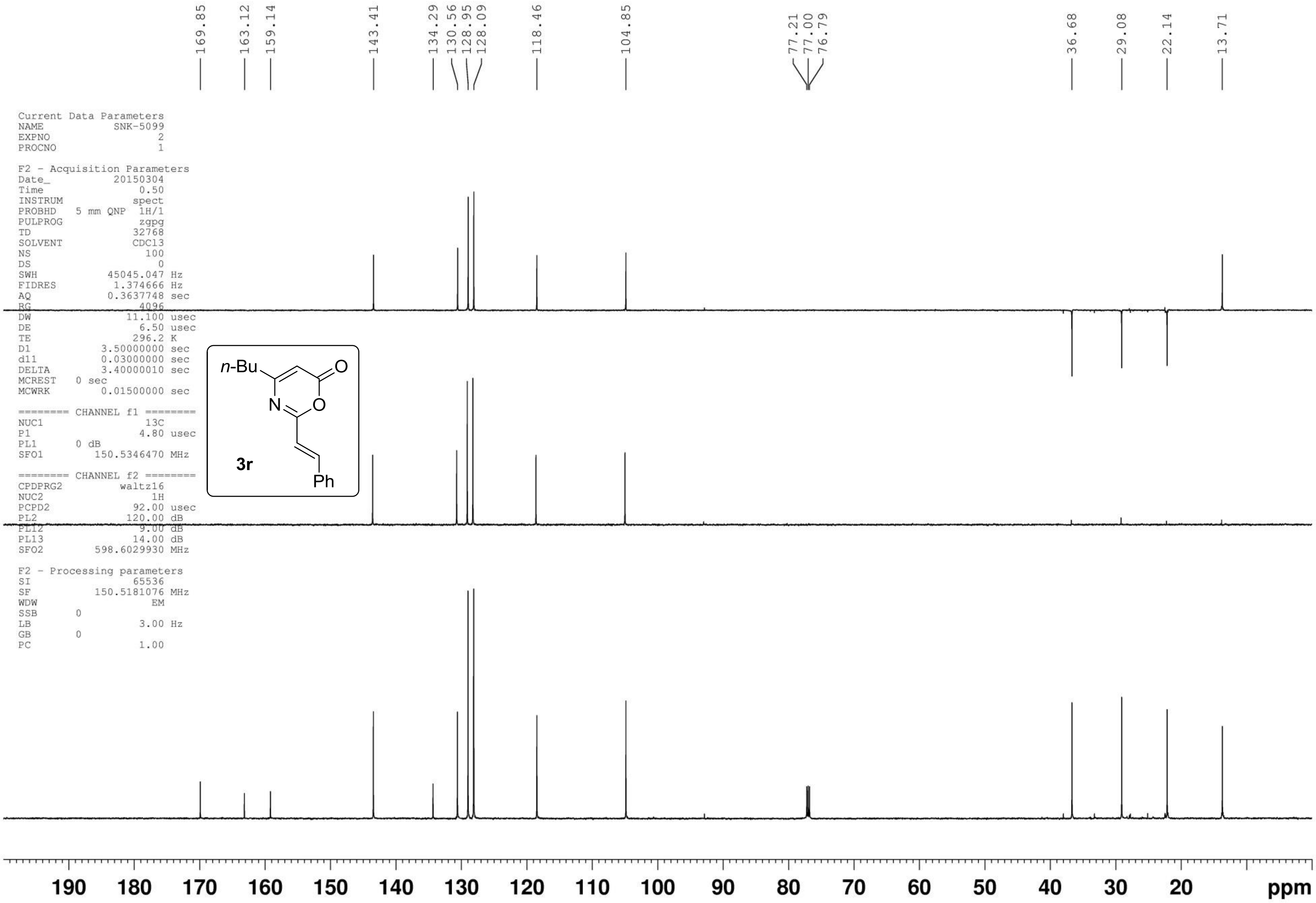

169.85
163.12
159.14
143.41
134.29
130.56
128.95
128.09
118.46
104.85
77.21
77.00
76.79
36.68
29.08
22.14
13.71
Current Data Parameters
NAME SNK-5099
EXPNO 2
PROCNO 1
F2 - Acquisition Parameters
Date_ 20150304
Time 0.50
INSTRUM spect
PROBHD 5 mm QNP 1H/1
PULPROG zgpg
TD 32768
SOLVENT CDCl3
NS 100
DS 0
SWH 45045.047 Hz
FIDRES 1.374666 Hz
AQ 0.3637748 sec
RG 4096
DW 11.100 usec
DE 6.50 usec
TE 296.2 K
D1 3.50000000 sec
d11 0.03000000 sec
DELTA 3.40000010 sec
MCREST 0 sec
MCWRK 0.01500000 sec
======== CHANNEL f1 ========
NUC1 13C
P1 4.80 usec
PL1 0 dB
SFO1 150.5346470 MHz
======== CHANNEL f2 ========
CPDPRG2 waltz16
NUC2 1H
PCPD2 92.00 usec
PL2 120.00 dB
PL12 9.00 dB
PL13 14.00 dB
SFO2 598.6029930 MHz
F2 - Processing parameters
SI 65536
SF 150.5181076 MHz
WDW EM
SSB 0
LB 3.00 Hz
GB 0
PC 1.00
n-Bu
O
N
O
3r
Ph
190
180
170
160
150
140
130
120
110
100
90
80
70
60
50
40
30
20
ppm

Current Data Parameters
NAME SNK-5096
EXPNO 1
PROCNO 1

F2 - Acquisition Parameters
Date_ 20150303
Time 1.58
INSTRUM spect
PROBHD 5 mm QNP 1H/1
PULPROG zg
TD 33556
SOLVENT CDCl3
NS 16
DS 0
SWH 8389.262 Hz
FIDRES 0.250008 Hz
AQ 1.9999876 sec
RG 32
DW 59.600 usec
DE 6.50 usec
TE 294.1 K
D1 2.00000000 sec
MCREST 0 sec
MCWRK 0.01500000 sec

======== CHANNEL f1 ========
NUC1 1H
P1 10.00 usec
PL1 0 dB
SFO1 598.6032923 MHz

F2 - Processing parameters
SI 32768
SF 598.6000309 MHz
WDW no
SSB 0
LB 0 Hz
GB 0
PC 1.00

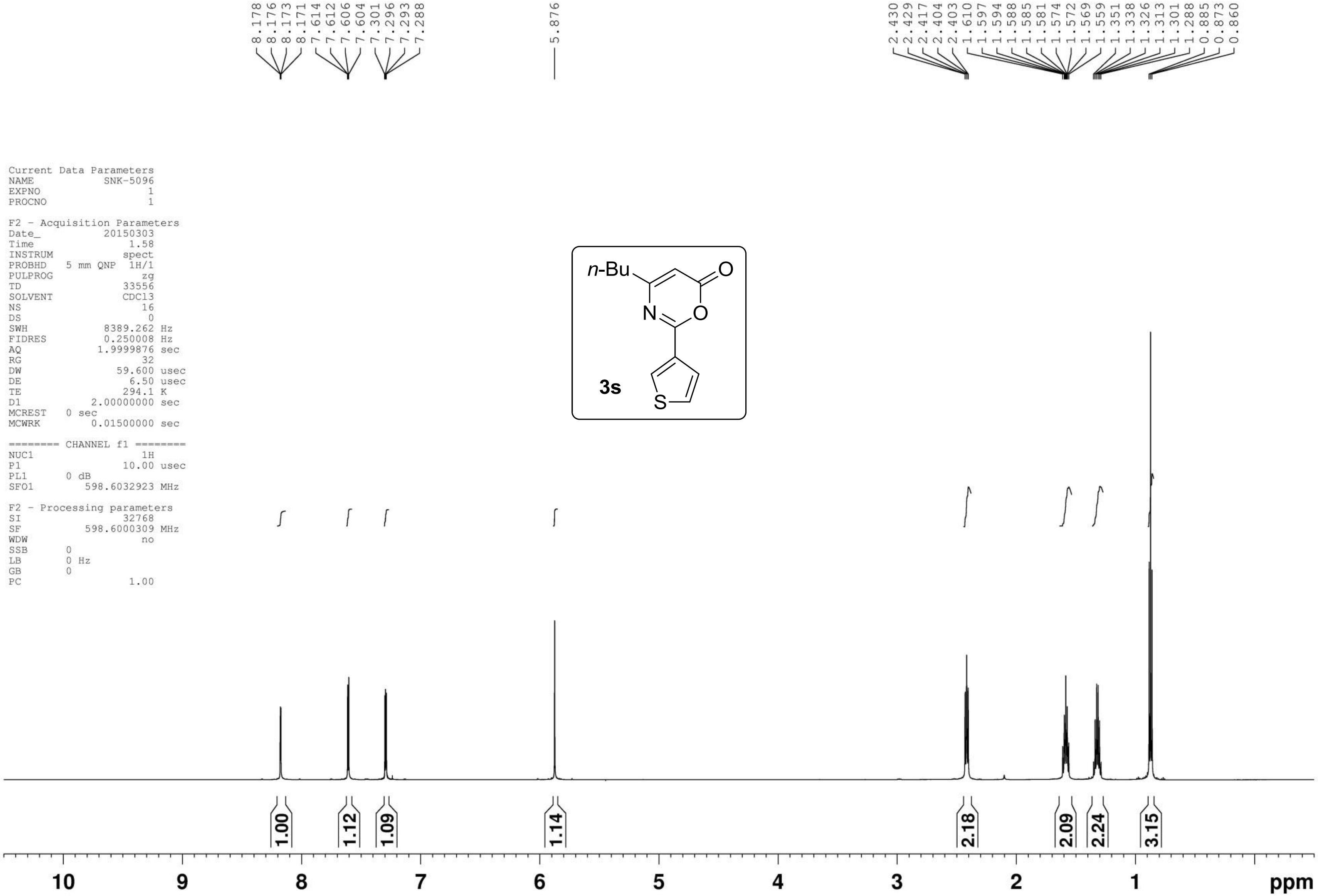

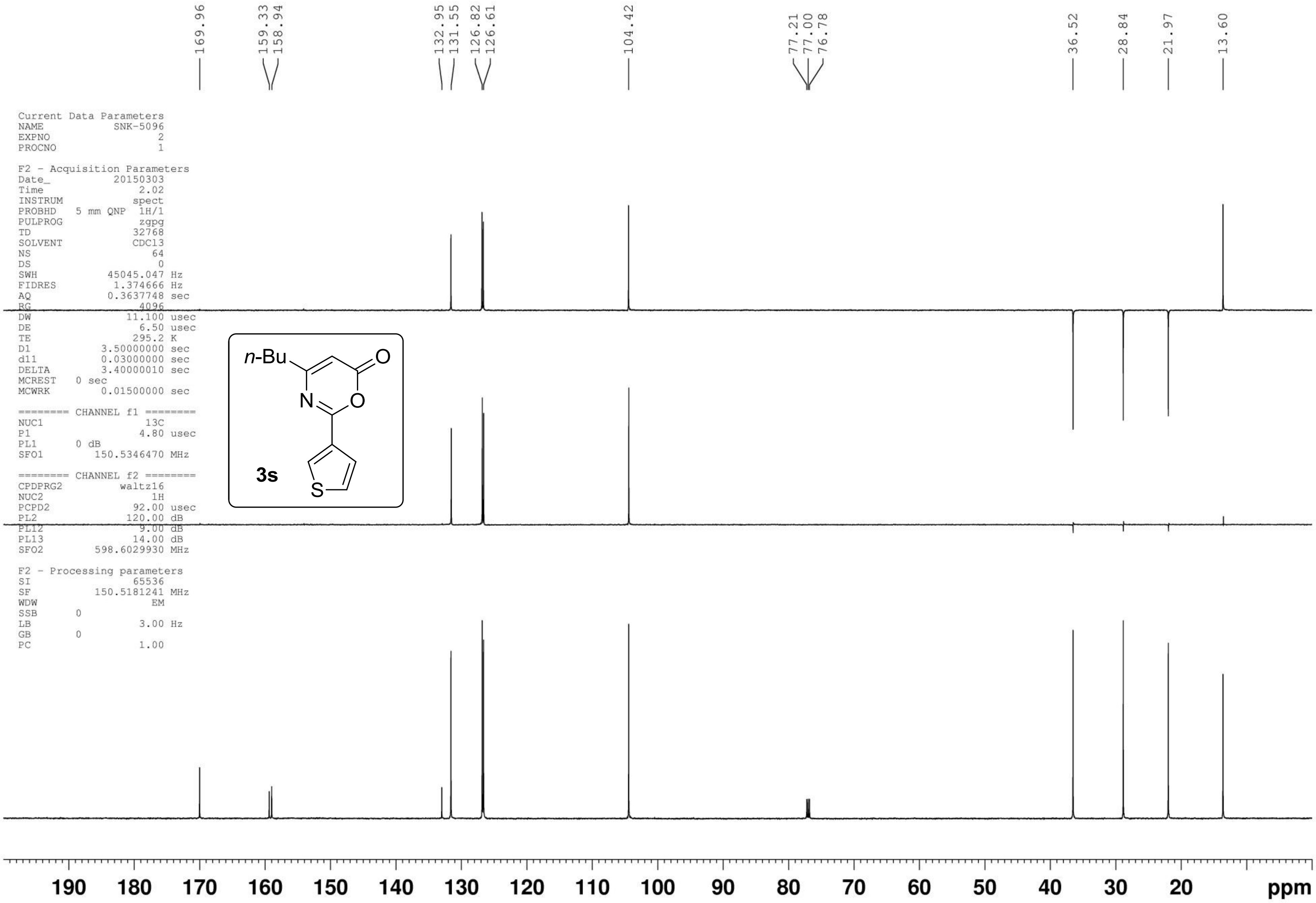

169.96
159.33
158.94
132.95
131.55
126.82
126.61
104.42
77.21
77.00
76.78
36.52
28.84
21.97
13.60
Current Data Parameters
NAME SNK-5096
EXPNO 2
PROCNO 1
F2 - Acquisition Parameters
Date_ 20150303
Time 2.02
INSTRUM spect
PROBHD 5 mm QNP 1H/1
PULPROG zgpg
TD 32768
SOLVENT CDCl3
NS 64
DS 0
SWH 45045.047 Hz
FIDRES 1.374666 Hz
AQ 0.3637748 sec
RG 4096
DW 11.100 usec
DE 6.50 usec
TE 295.2 K
D1 3.50000000 sec
d11 0.03000000 sec
DELTA 3.40000010 sec
MCREST 0 sec
MCWRK 0.01500000 sec
======== CHANNEL f1 ========
NUC1 13C
P1 4.80 usec
PL1 0 dB
SFO1 150.5346470 MHz
======== CHANNEL f2 ========
CPDPRG2 waltz16
NUC2 1H
PCPD2 92.00 usec
PL2 120.00 dB
PL12 9.00 dB
PL13 14.00 dB
SFO2 598.6029930 MHz
F2 - Processing parameters
SI 65536
SF 150.5181241 MHz
WDW EM
SSB 0
LB 3.00 Hz
GB 0
PC 1.00
n-Bu
O
N
O
S
3s
190
180
170
160
150
140
130
120
110
100
90
80
70
60
50
40
30
20
ppm

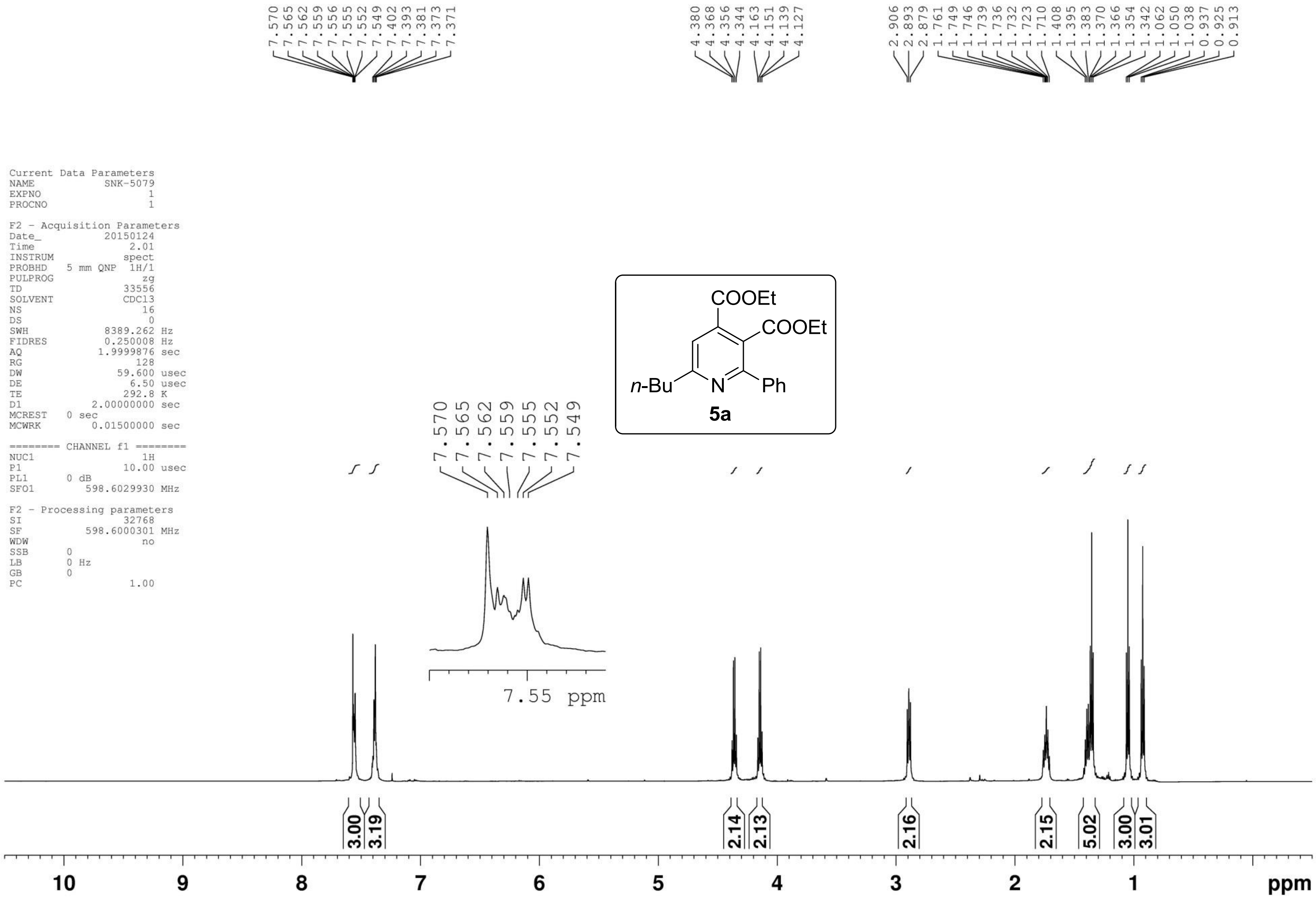

7.570 7.565 7.562 7.559 7.556 7.555 7.552 7.549 7.402 7.393 7.381 7.373 7.371
4.380 4.368 4.356 4.344 4.163 4.151 4.139 4.127
2.906 2.893 2.879 1.761 1.749 1.746 1.739 1.736 1.732 1.723 1.710 1.408 1.395 1.383 1.370 1.366 1.354 1.342 1.062 1.050 1.038 0.937 0.925 0.913
Current Data Parameters
NAME SNK-5079
EXPNO 1
PROCNO 1
F2 - Acquisition Parameters
Date_ 20150124
Time 2.01
INSTRUM spect
PROBHD 5 mm QNP 1H/1
PULPROG zg
TD 33556
SOLVENT CDCl3
NS 16
DS 0
SWH 8389.262 Hz
FIDRES 0.250008 Hz
AQ 1.9999876 sec
RG 128
DW 59.600 usec
DE 6.50 usec
TE 292.8 K
D1 2.00000000 sec
MCREST 0 sec
MCWRK 0.01500000 sec
======== CHANNEL f1 ========
NUC1 1H
P1 10.00 usec
PL1 0 dB
SFO1 598.6029930 MHz
F2 - Processing parameters
SI 32768
SF 598.6000301 MHz
WDW no
SSB 0
LB 0 Hz
GB 0
PC 1.00
COOEt
COOEt
n-Bu
N
Ph
5a
7.570 7.565 7.562 7.559 7.555 7.552 7.549
7.55 ppm
3.00 3.19 2.14 2.13 2.16 2.15 5.02 3.00 3.01
10 9 8 7 6 5 4 3 2 1 ppm

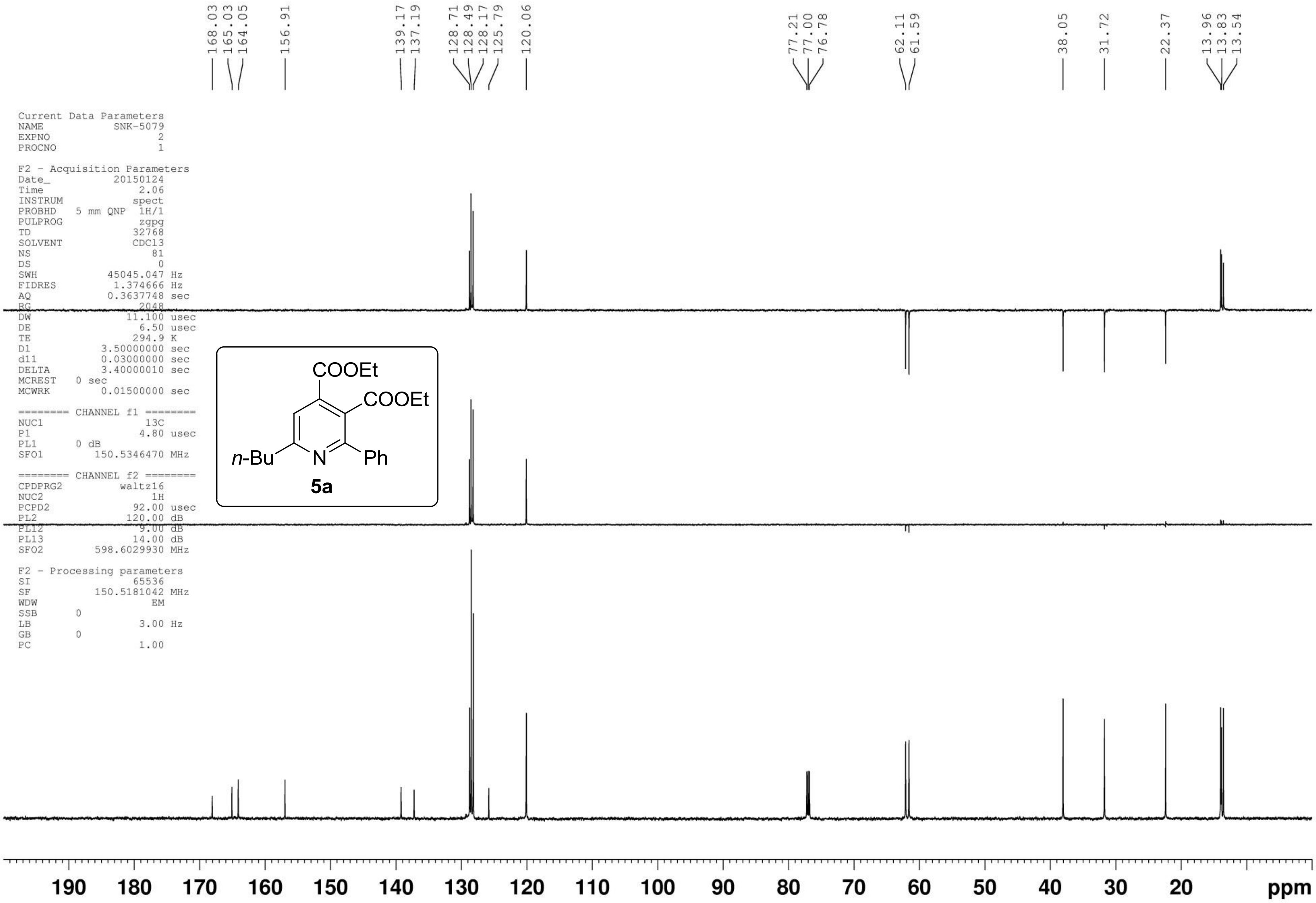


```
Current Data Parameters
NAME            SNK-5079
EXPNO                  2
PROCNO                 1

F2 - Acquisition Parameters
Date_           20150124
Time                2.06
INSTRUM            spect
PROBHD   5 mm QNP  1H/1
PULPROG             zgpg
TD                 32768
SOLVENT            CDC13
NS                    81
DS                     0
SWH            45045.047 Hz
FIDRES          1.374666 Hz
AQ             0.3637748 sec
RG                  2048
DW                11.100 usec
DE                  6.50 usec
TE                 294.9 K
D1            3.50000000 sec
d11           0.03000000 sec
DELTA         3.40000010 sec
MCREST   0 sec
MCWRK         0.01500000 sec

======== CHANNEL f1 ========
NUC1                 13C
P1                  4.80 usec
PL1      0 dB
SFO1         150.5346470 MHz

======== CHANNEL f2 ========
CPDPRG2          waltz16
NUC2                  1H
PCPD2              92.00 usec
PL2               120.00 dB
PL12                9.00 dB
PL13               14.00 dB
SFO2         598.6029930 MHz

F2 - Processing parameters
SI                 65536
SF           150.5181042 MHz
WDW                   EM
SSB      0
LB                  3.00 Hz
GB       0
PC                  1.00
```

5a

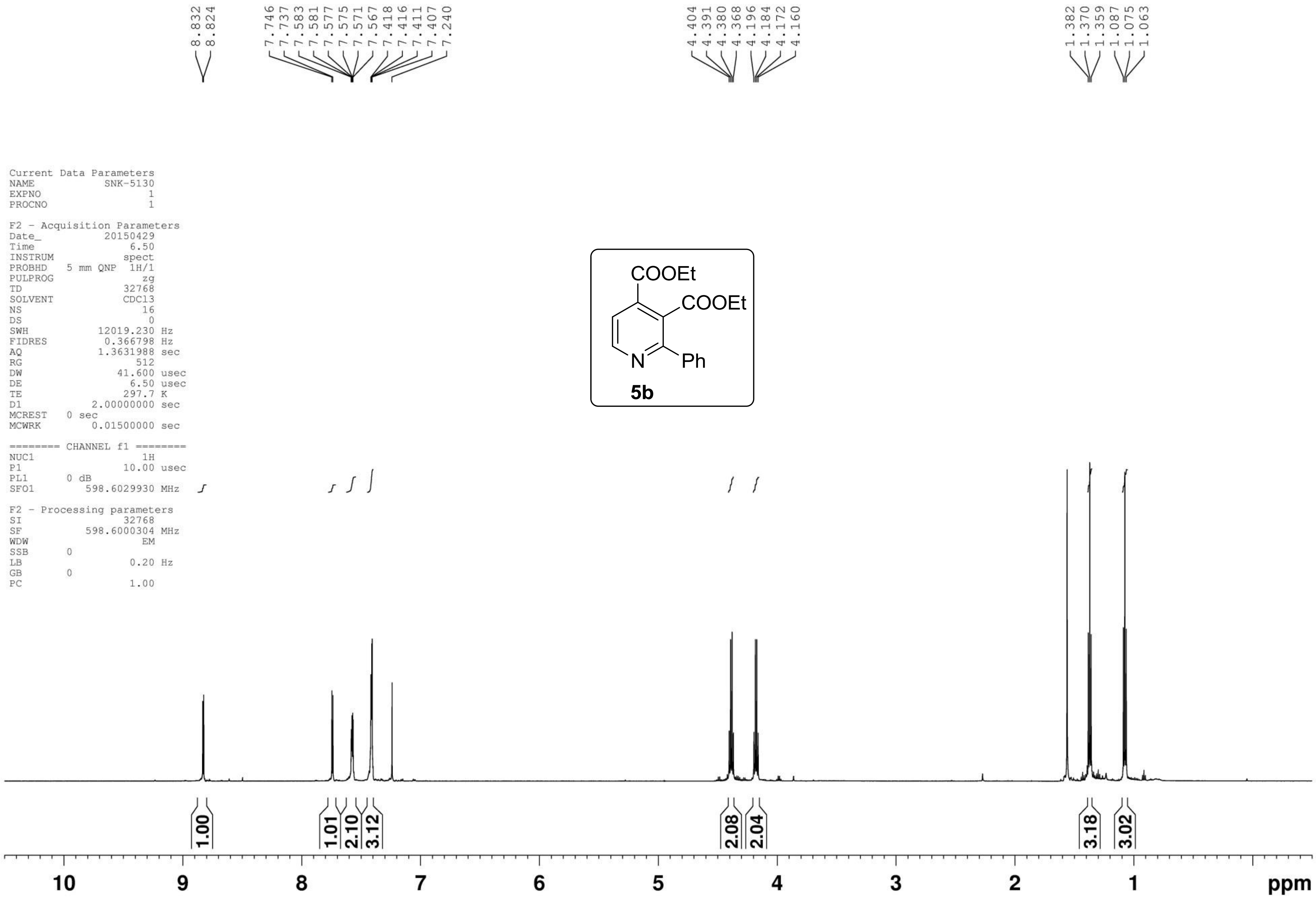
8.832
8.824
7.746
7.737
7.583
7.581
7.577
7.575
7.571
7.567
7.418
7.416
7.411
7.407
7.240
4.404
4.391
4.380
4.368
4.196
4.184
4.172
4.160
1.382
1.370
1.359
1.087
1.075
1.063
Current Data Parameters
NAME SNK-5130
EXPNO 1
PROCNO 1
F2 - Acquisition Parameters
Date_ 20150429
Time 6.50
INSTRUM spect
PROBHD 5 mm QNP 1H/1
PULPROG zg
TD 32768
SOLVENT CDCl3
NS 16
DS 0
SWH 12019.230 Hz
FIDRES 0.366798 Hz
AQ 1.3631988 sec
RG 512
DW 41.600 usec
DE 6.50 usec
TE 297.7 K
D1 2.00000000 sec
MCREST 0 sec
MCWRK 0.01500000 sec
======== CHANNEL f1 ========
NUC1 1H
P1 10.00 usec
PL1 0 dB
SFO1 598.6029930 MHz
F2 - Processing parameters
SI 32768
SF 598.6000304 MHz
WDW EM
SSB 0
LB 0.20 Hz
GB 0
PC 1.00
COOEt
COOEt
N
Ph
5b
1.00
1.01
2.10
3.12
2.08
2.04
3.18
3.02
10
9
8
7
6
5
4
3
2
1
ppm

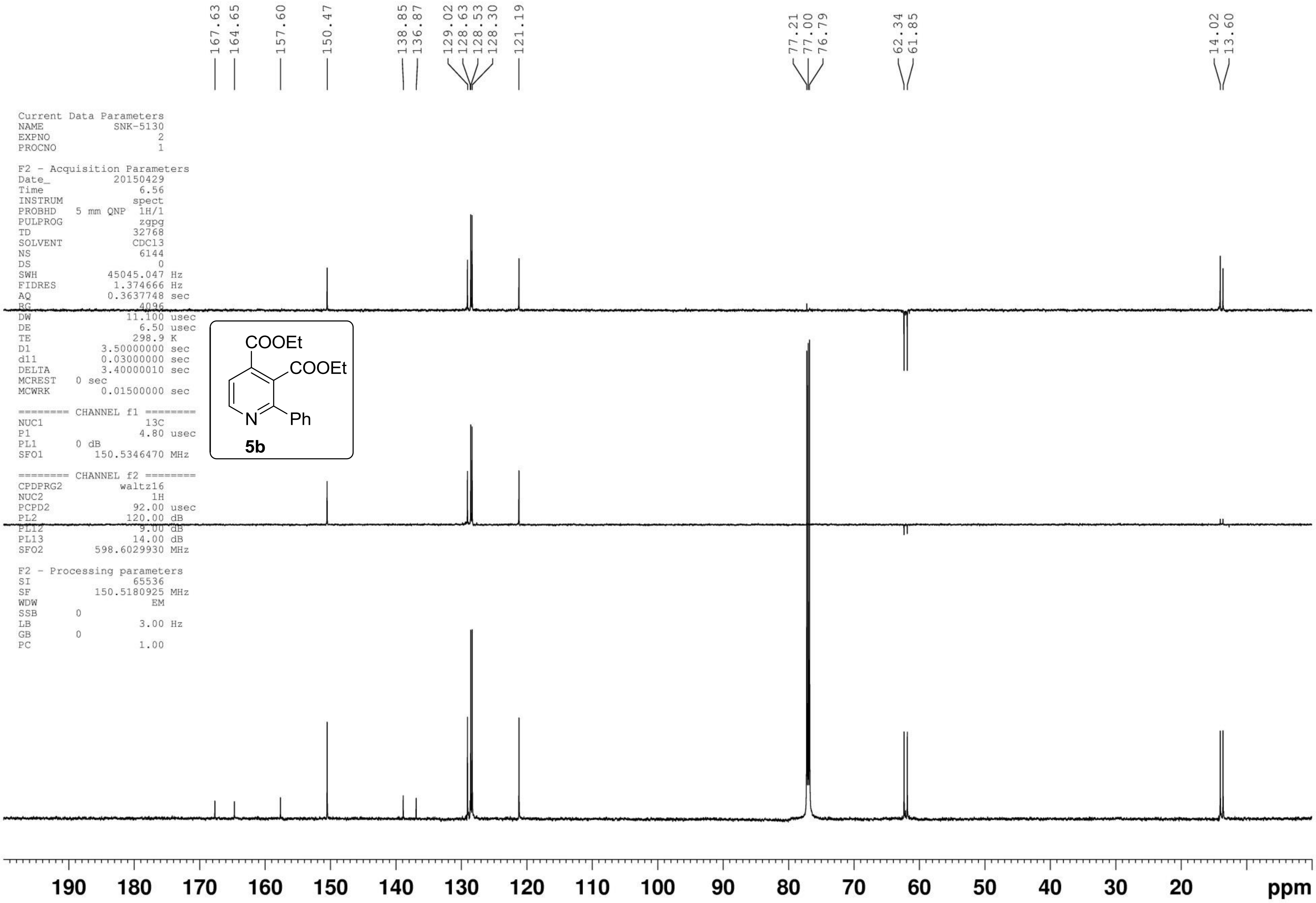
167.63
164.65
157.60
150.47
138.85
136.87
129.02
128.63
128.53
128.30
121.19
77.21
77.00
76.79
62.34
61.85
14.02
13.60
Current Data Parameters
NAME SNK-5130
EXPNO 2
PROCNO 1
F2 - Acquisition Parameters
Date_ 20150429
Time 6.56
INSTRUM spect
PROBHD 5 mm QNP 1H/1
PULPROG zgpg
TD 32768
SOLVENT CDCl3
NS 6144
DS 0
SWH 45045.047 Hz
FIDRES 1.374666 Hz
AQ 0.3637748 sec
RG 4096
DW 11.100 usec
DE 6.50 usec
TE 298.9 K
D1 3.50000000 sec
d11 0.03000000 sec
DELTA 3.40000010 sec
MCREST 0 sec
MCWRK 0.01500000 sec
======== CHANNEL f1 ========
NUC1 13C
P1 4.80 usec
PL1 0 dB
SFO1 150.5346470 MHz
======== CHANNEL f2 ========
CPDPRG2 waltz16
NUC2 1H
PCPD2 92.00 usec
PL2 120.00 dB
PL12 9.00 dB
PL13 14.00 dB
SFO2 598.6029930 MHz
F2 - Processing parameters
SI 65536
SF 150.5180925 MHz
WDW EM
SSB 0
LB 3.00 Hz
GB 0
PC 1.00
COOEt
COOEt
N
Ph
5b
190 180 170 160 150 140 130 120 110 100 90 80 70 60 50 40 30 20 ppm

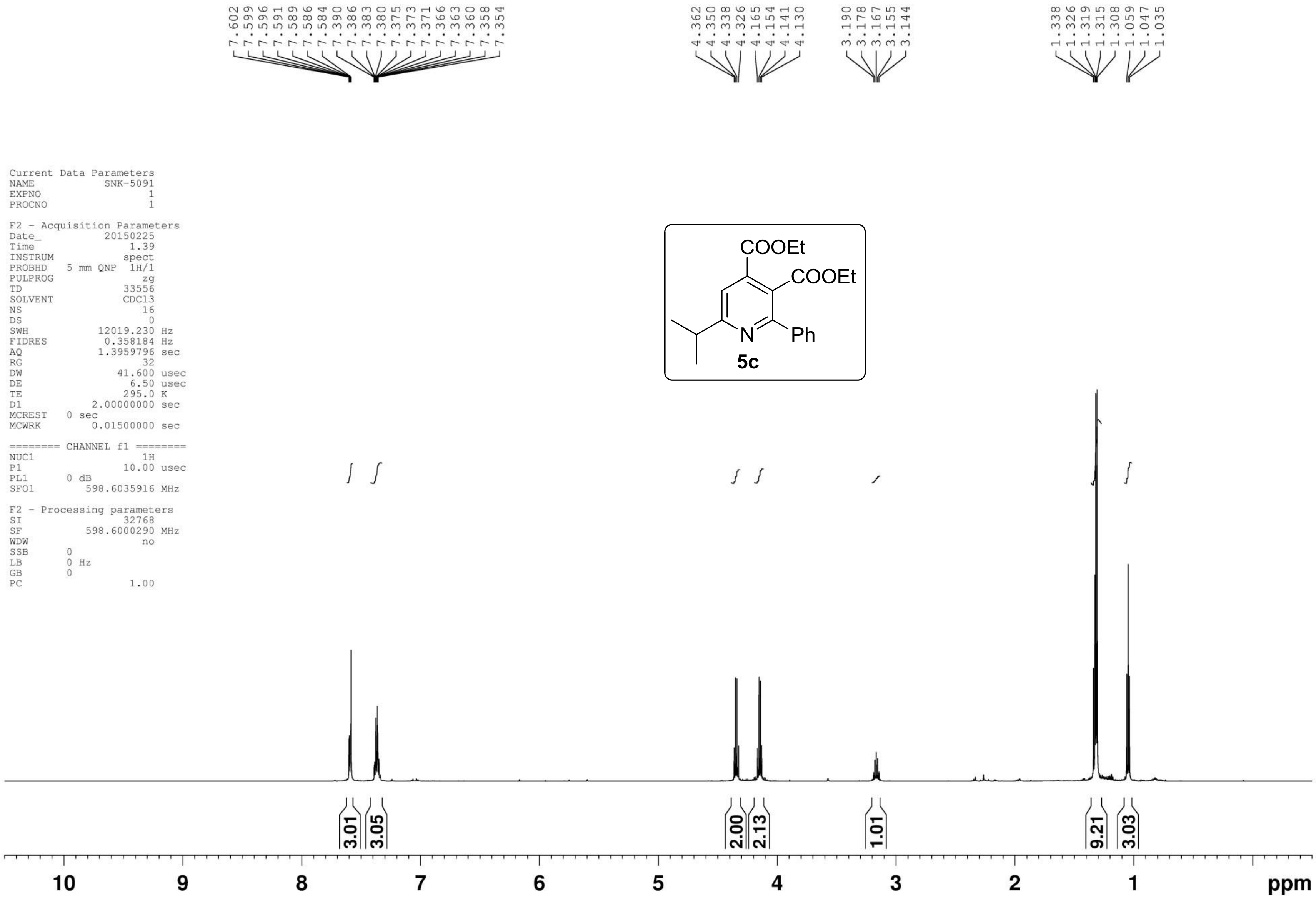
7.602
7.599
7.596
7.591
7.589
7.586
7.584
7.390
7.386
7.383
7.380
7.375
7.373
7.371
7.366
7.363
7.360
7.358
7.354
4.362
4.350
4.338
4.326
4.165
4.154
4.141
4.130
3.190
3.178
3.167
3.155
3.144
1.338
1.326
1.319
1.315
1.308
1.059
1.047
1.035
Current Data Parameters
NAME SNK-5091
EXPNO 1
PROCNO 1
F2 - Acquisition Parameters
Date_ 20150225
Time 1.39
INSTRUM spect
PROBHD 5 mm QNP 1H/1
PULPROG zg
TD 33556
SOLVENT CDCl3
NS 16
DS 0
SWH 12019.230 Hz
FIDRES 0.358184 Hz
AQ 1.3959796 sec
RG 32
DW 41.600 usec
DE 6.50 usec
TE 295.0 K
D1 2.00000000 sec
MCREST 0 sec
MCWRK 0.01500000 sec
======== CHANNEL f1 ========
NUC1 1H
P1 10.00 usec
PL1 0 dB
SFO1 598.6035916 MHz
F2 - Processing parameters
SI 32768
SF 598.6000290 MHz
WDW no
SSB 0
LB 0 Hz
GB 0
PC 1.00
COOEt
COOEt
N
Ph
5c
3.01
3.05
2.00
2.13
1.01
9.21
3.03
10
9
8
7
6
5
4
3
2
1
ppm

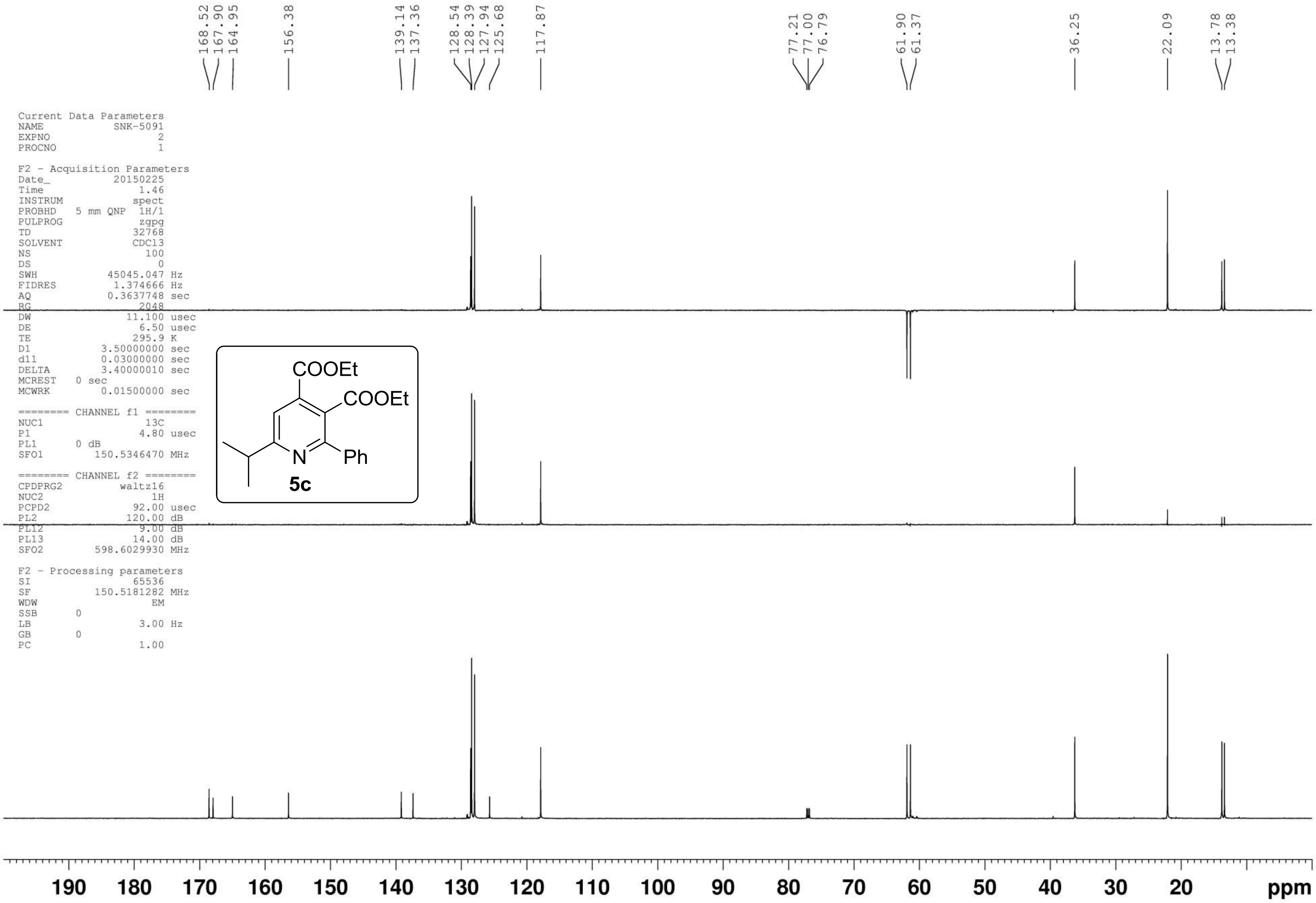
168.52
167.90
164.95
156.38
139.14
137.36
128.54
128.39
127.94
125.68
117.87
77.21
77.00
76.79
61.90
61.37
36.25
22.09
13.78
13.38
Current Data Parameters
NAME SNK-5091
EXPNO 2
PROCNO 1
F2 - Acquisition Parameters
Date_ 20150225
Time 1.46
INSTRUM spect
PROBHD 5 mm QNP 1H/1
PULPROG zgpg
TD 32768
SOLVENT CDCl3
NS 100
DS 0
SWH 45045.047 Hz
FIDRES 1.374666 Hz
AQ 0.3637748 sec
RG 2048
DW 11.100 usec
DE 6.50 usec
TE 295.9 K
D1 3.50000000 sec
d11 0.03000000 sec
DELTA 3.40000010 sec
MCREST 0 sec
MCWRK 0.01500000 sec
======== CHANNEL f1 ========
NUC1 13C
P1 4.80 usec
PL1 0 dB
SFO1 150.5346470 MHz
======== CHANNEL f2 ========
CPDPRG2 waltz16
NUC2 1H
PCPD2 92.00 usec
PL2 120.00 dB
PL12 9.00 dB
PL13 14.00 dB
SFO2 598.6029930 MHz
F2 - Processing parameters
SI 65536
SF 150.5181282 MHz
WDW EM
SSB 0
LB 3.00 Hz
GB 0
PC 1.00
COOEt
COOEt
N
Ph
5c
190 180 170 160 150 140 130 120 110 100 90 80 70 60 50 40 30 20 ppm

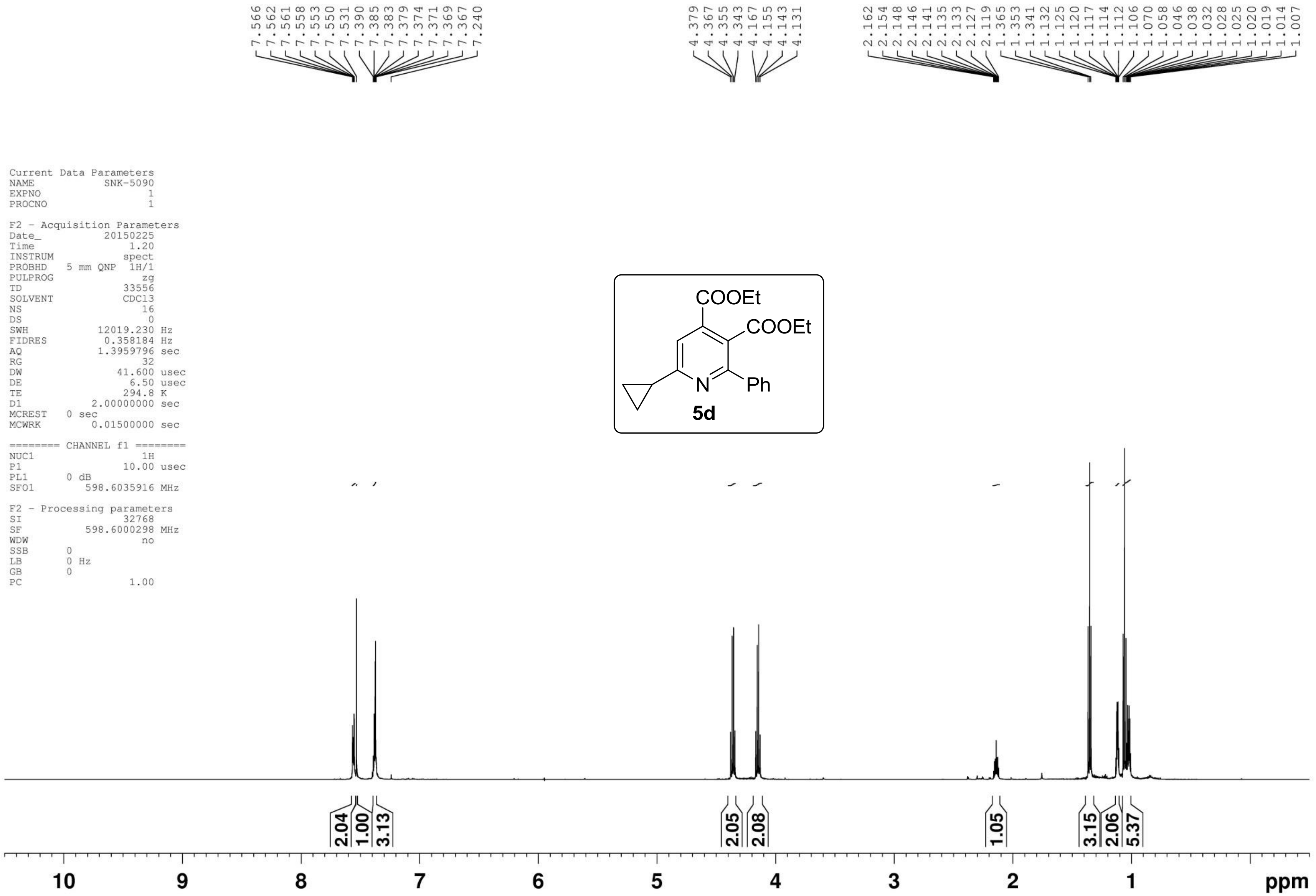
7.566
7.562
7.561
7.558
7.553
7.550
7.531
7.390
7.385
7.383
7.379
7.374
7.371
7.369
7.367
7.240
4.379
4.367
4.355
4.343
4.167
4.155
4.143
4.131
2.162
2.154
2.148
2.146
2.141
2.135
2.133
2.127
2.119
1.365
1.353
1.341
1.132
1.125
1.120
1.117
1.114
1.112
1.106
1.070
1.058
1.046
1.038
1.032
1.028
1.025
1.020
1.019
1.014
1.007
Current Data Parameters
NAME SNK-5090
EXPNO 1
PROCNO 1
F2 - Acquisition Parameters
Date_ 20150225
Time 1.20
INSTRUM spect
PROBHD 5 mm QNP 1H/1
PULPROG zg
TD 33556
SOLVENT CDCl3
NS 16
DS 0
SWH 12019.230 Hz
FIDRES 0.358184 Hz
AQ 1.3959796 sec
RG 32
DW 41.600 usec
DE 6.50 usec
TE 294.8 K
D1 2.00000000 sec
MCREST 0 sec
MCWRK 0.01500000 sec
======== CHANNEL f1 ========
NUC1 1H
P1 10.00 usec
PL1 0 dB
SFO1 598.6035916 MHz
F2 - Processing parameters
SI 32768
SF 598.6000298 MHz
WDW no
SSB 0
LB 0 Hz
GB 0
PC 1.00
2.04
1.00
3.13
2.05
2.08
1.05
3.15
2.06
5.37
10
9
8
7
6
5
4
3
2
1
ppm
COOEt
COOEt
N
Ph

5d

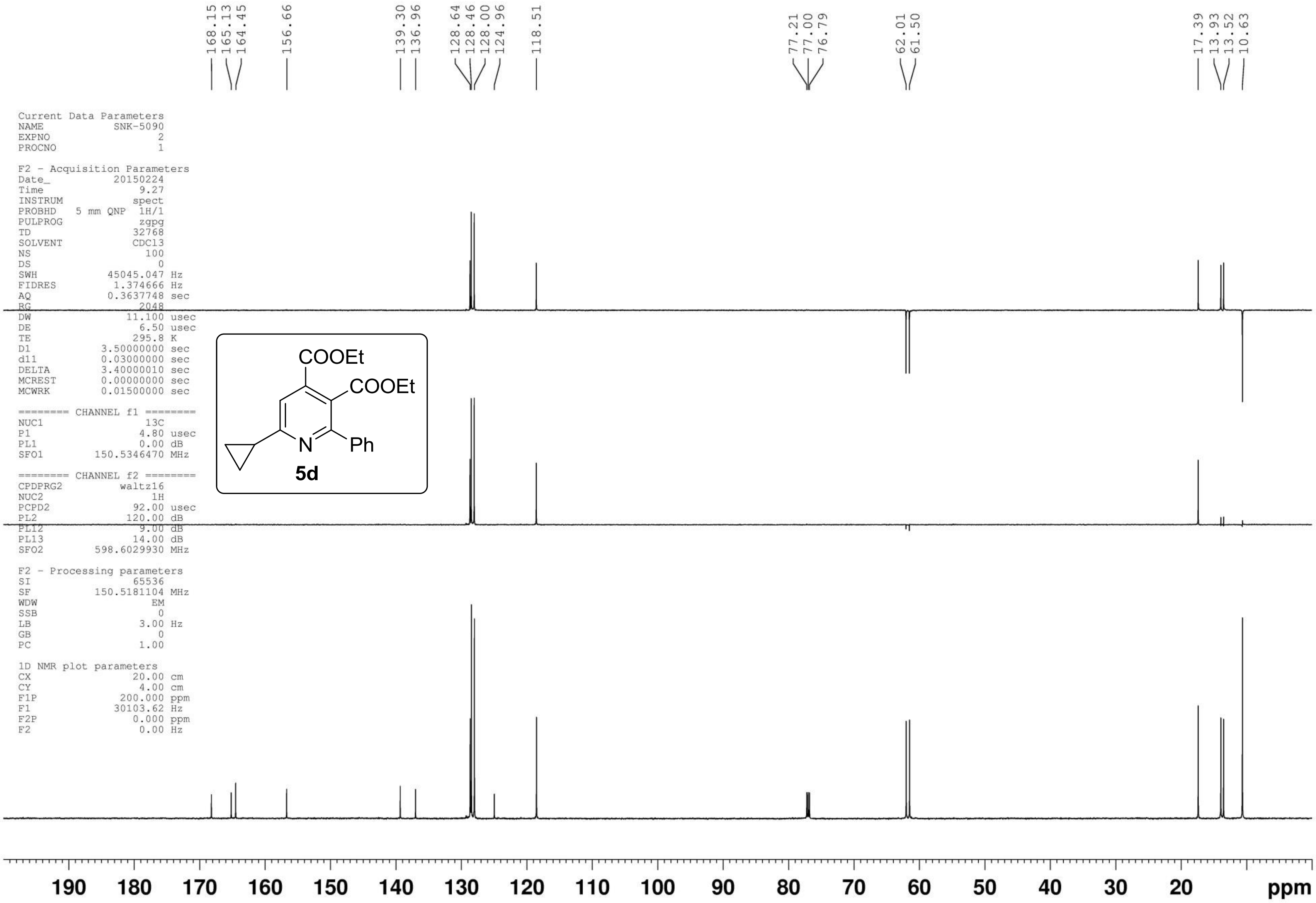
168.15
165.13
164.45
156.66
139.30
136.96
128.64
128.46
128.00
124.96
118.51
77.21
77.00
76.79
62.01
61.50
17.39
13.93
13.52
10.63
Current Data Parameters
NAME SNK-5090
EXPNO 2
PROCNO 1
F2 - Acquisition Parameters
Date_ 20150224
Time 9.27
INSTRUM spect
PROBHD 5 mm QNP 1H/1
PULPROG zgpg
TD 32768
SOLVENT CDCl3
NS 100
DS 0
SWH 45045.047 Hz
FIDRES 1.374666 Hz
AQ 0.3637748 sec
RG 2048
DW 11.100 usec
DE 6.50 usec
TE 295.8 K
D1 3.50000000 sec
d11 0.03000000 sec
DELTA 3.40000010 sec
MCREST 0.00000000 sec
MCWRK 0.01500000 sec
======== CHANNEL f1 ========
NUC1 13C
P1 4.80 usec
PL1 0.00 dB
SFO1 150.5346470 MHz
======== CHANNEL f2 ========
CPDPRG2 waltz16
NUC2 1H
PCPD2 92.00 usec
PL2 120.00 dB
PL12 9.00 dB
PL13 14.00 dB
SFO2 598.6029930 MHz
F2 - Processing parameters
SI 65536
SF 150.5181104 MHz
WDW EM
SSB 0
LB 3.00 Hz
GB 0
PC 1.00
1D NMR plot parameters
CX 20.00 cm
CY 4.00 cm
F1P 200.000 ppm
F1 30103.62 Hz
F2P 0.000 ppm
F2 0.00 Hz
COOEt
COOEt
N
Ph
5d
190 180 170 160 150 140 130 120 110 100 90 80 70 60 50 40 30 20 ppm

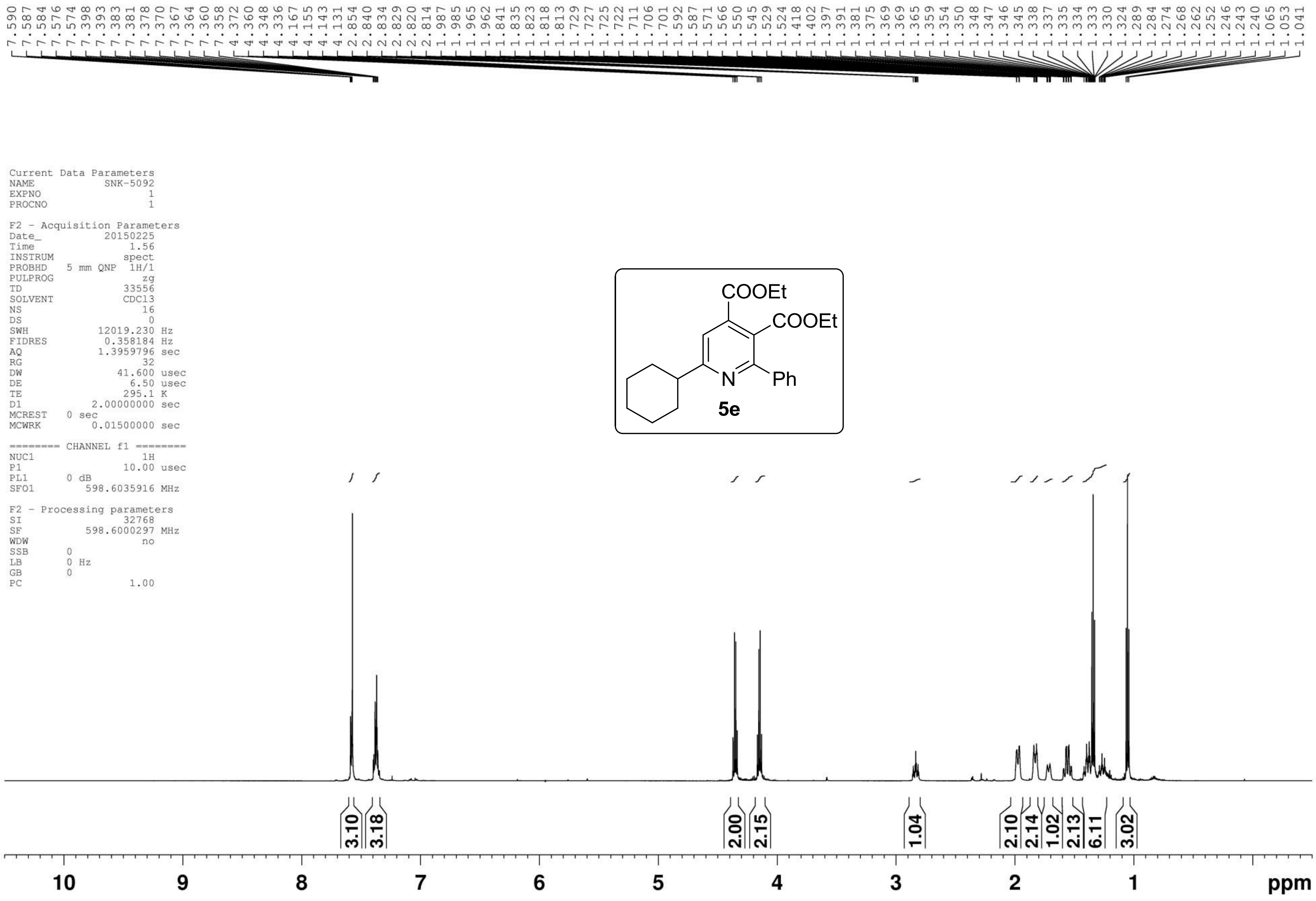
7.590 7.587 7.584 7.576 7.574 7.398 7.393 7.383 7.381 7.378 7.370 7.367 7.364 7.360 7.358 4.372 4.360 4.348 4.336 4.167 4.155 4.143 4.131 2.854 2.840 2.834 2.829 2.820 2.814 1.987 1.985 1.965 1.962 1.841 1.835 1.823 1.818 1.813 1.729 1.727 1.725 1.722 1.711 1.706 1.701 1.592 1.587 1.571 1.566 1.550 1.545 1.529 1.524 1.418 1.402 1.397 1.391 1.381 1.375 1.369 1.369 1.365 1.359 1.354 1.350 1.348 1.347 1.346 1.345 1.338 1.337 1.335 1.334 1.333 1.330 1.324 1.289 1.284 1.274 1.268 1.262 1.252 1.246 1.243 1.240 1.065 1.053 1.041
Current Data Parameters
NAME SNK-5092
EXPNO 1
PROCNO 1
F2 - Acquisition Parameters
Date_ 20150225
Time 1.56
INSTRUM spect
PROBHD 5 mm QNP 1H/1
PULPROG zg
TD 33556
SOLVENT CDCl3
NS 16
DS 0
SWH 12019.230 Hz
FIDRES 0.358184 Hz
AQ 1.3959796 sec
RG 32
DW 41.600 usec
DE 6.50 usec
TE 295.1 K
D1 2.00000000 sec
MCREST 0 sec
MCWRK 0.01500000 sec
======== CHANNEL f1 ========
NUC1 1H
P1 10.00 usec
PL1 0 dB
SFO1 598.6035916 MHz
F2 - Processing parameters
SI 32768
SF 598.6000297 MHz
WDW no
SSB 0
LB 0 Hz
GB 0
PC 1.00
COOEt
COOEt
N
Ph
5e
3.10 3.18 2.00 2.15 1.04 2.10 2.14 1.02 2.13 6.11 3.02
10 9 8 7 6 5 4 3 2 1 ppm

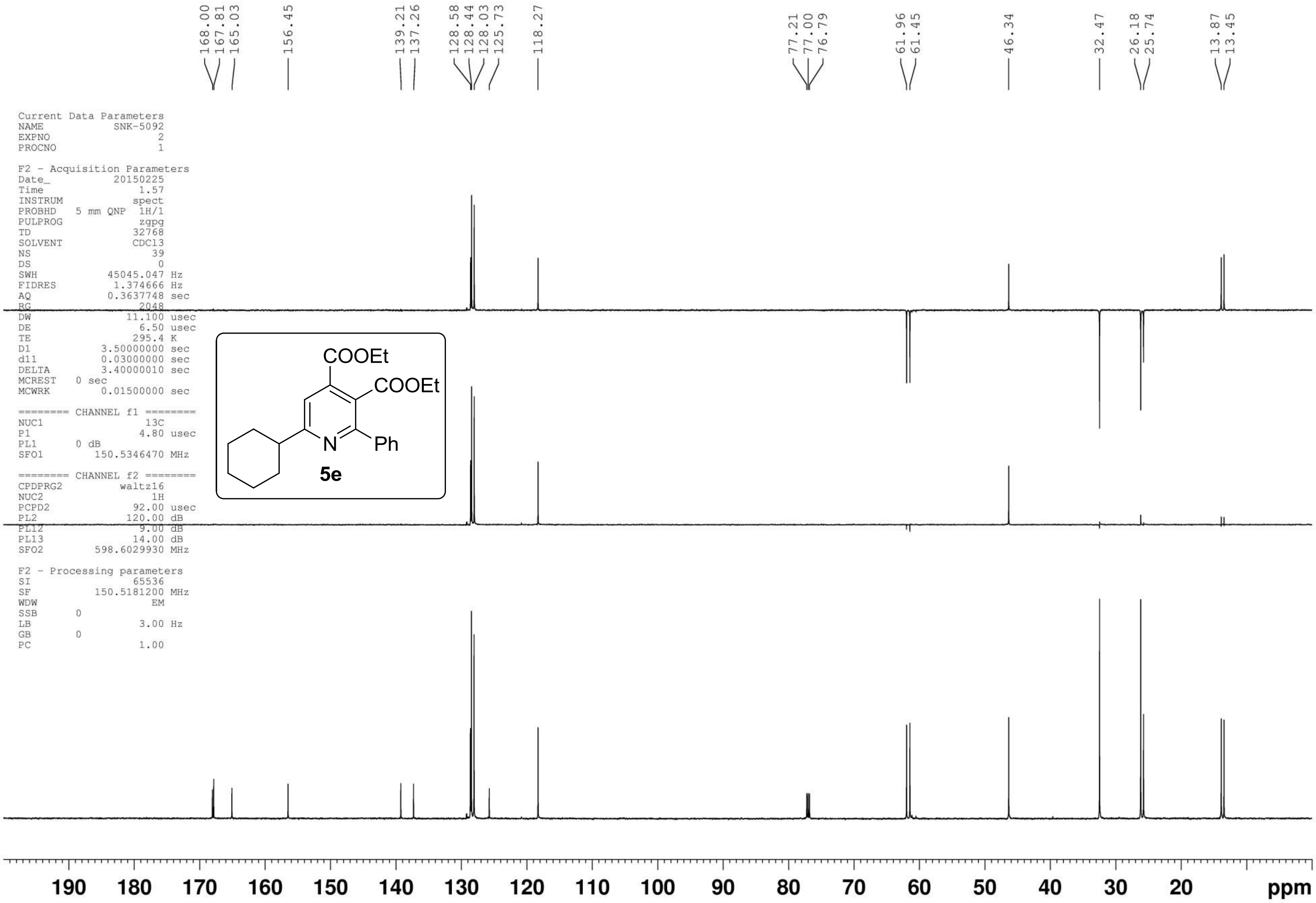

5e

```
Current Data Parameters
NAME            SNK-5092
EXPNO                  2
PROCNO                 1

F2 - Acquisition Parameters
Date_           20150225
Time                1.57
INSTRUM            spect
PROBHD   5 mm QNP  1H/1
PULPROG             zgpg
TD                 32768
SOLVENT            CDCl3
NS                    39
DS                     0
SWH            45045.047 Hz
FIDRES          1.374666 Hz
AQ             0.3637748 sec
RG                  2048
DW                11.100 usec
DE                  6.50 usec
TE                 295.4 K
D1            3.50000000 sec
d11           0.03000000 sec
DELTA         3.40000010 sec
MCREST   0 sec
MCWRK         0.01500000 sec

======== CHANNEL f1 ========
NUC1                 13C
P1                  4.80 usec
PL1      0 dB
SFO1         150.5346470 MHz

======== CHANNEL f2 ========
CPDPRG2          waltz16
NUC2                  1H
PCPD2              92.00 usec
PL2               120.00 dB
PL12                9.00 dB
PL13               14.00 dB
SFO2         598.6029930 MHz

F2 - Processing parameters
SI                 65536
SF           150.5181200 MHz
WDW                   EM
SSB      0
LB                  3.00 Hz
GB       0
PC                  1.00
```

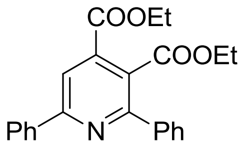

5f

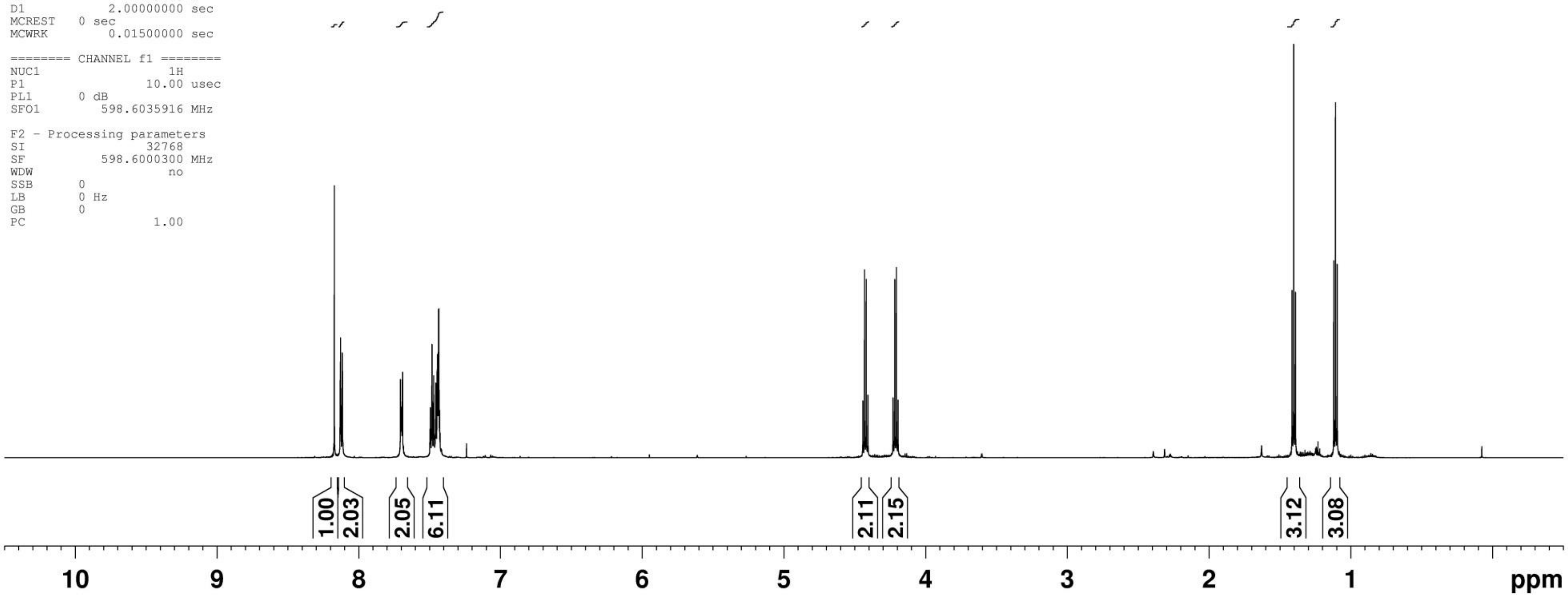
8.173
8.131
8.129
8.125
8.120
8.117
8.115
7.706
7.702
7.699
7.698
7.696
7.693
7.690
7.498
7.495
7.492
7.484
7.481
7.473
7.471
7.467
7.462
7.459
7.456
7.454
7.447
7.444
7.441
7.437
7.435
7.433
7.430
7.426
7.240
4.443
4.431
4.419
4.407
4.230
4.218
4.206
4.194
1.414
1.402
1.390
1.120
1.108
1.096
Current Data Parameters
NAME SNK-5089
EXPNO 1
PROCNO 1
F2 - Acquisition Parameters
Date_ 20150225
Time 0.21
INSTRUM spect
PROBHD 5 mm QNP 1H/1
PULPROG zg
TD 33556
SOLVENT CDCl3
NS 16
DS 0
SWH 12019.230 Hz
FIDRES 0.358184 Hz
AQ 1.3959796 sec
RG 128
DW 41.600 usec
DE 6.50 usec
TE 294.8 K
D1 2.00000000 sec
MCREST 0 sec
MCWRK 0.01500000 sec
======== CHANNEL f1 ========
NUC1 1H
P1 10.00 usec
PL1 0 dB
SFO1 598.6035916 MHz
F2 - Processing parameters
SI 32768
SF 598.6000300 MHz
WDW no
SSB 0
LB 0 Hz
GB 0
PC 1.00
1.00
2.03
2.05
6.11
2.11
2.15
3.12
3.08
10
9
8
7
6
5
4
3
2
1
ppm

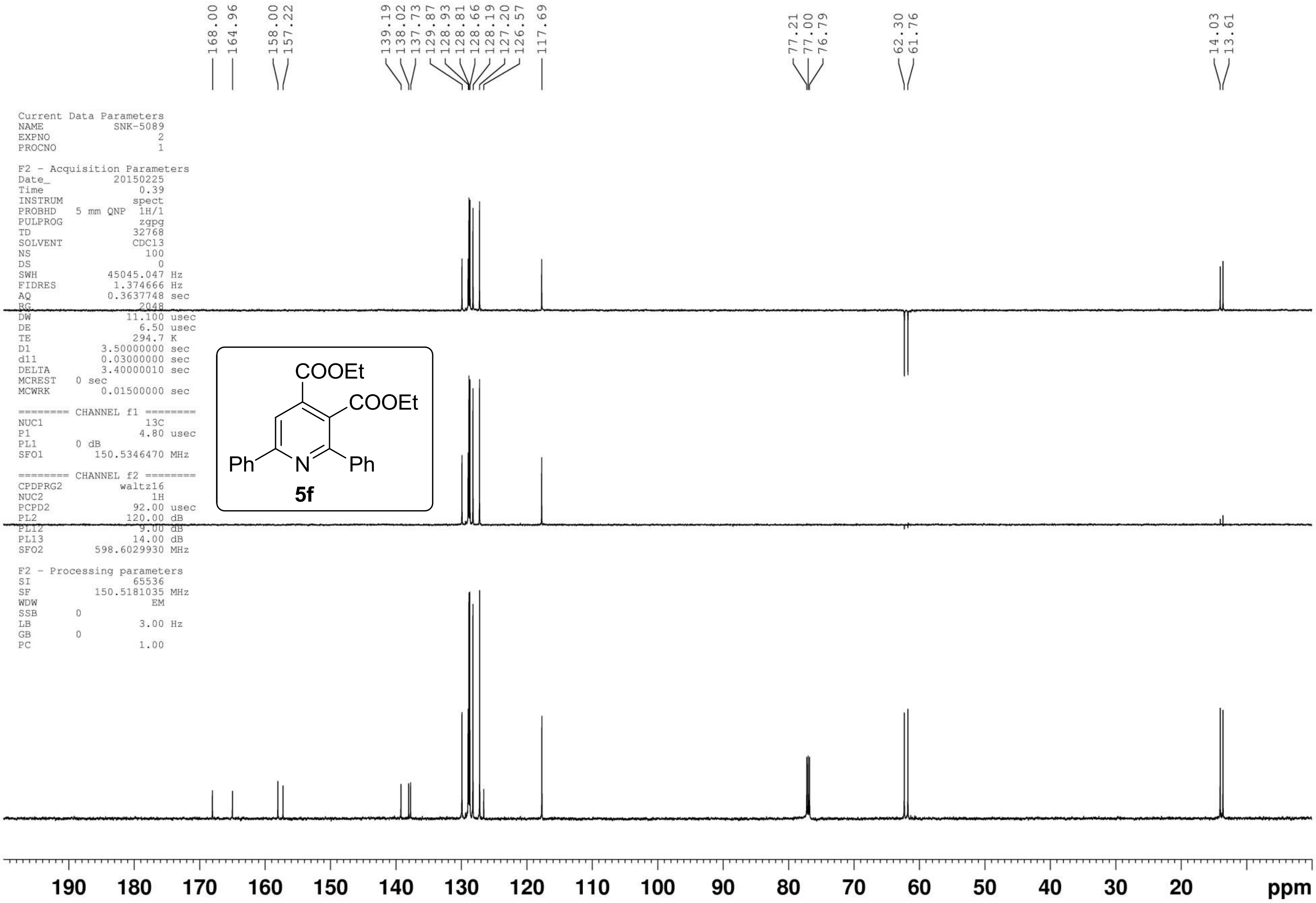

168.00
164.96
158.00
157.22
139.19
138.02
137.73
129.87
128.93
128.81
128.66
128.19
127.20
126.57
117.69
77.21
77.00
76.79
62.30
61.76
14.03
13.61
Current Data Parameters
NAME SNK-5089
EXPNO 2
PROCNO 1
F2 - Acquisition Parameters
Date_ 20150225
Time 0.39
INSTRUM spect
PROBHD 5 mm QNP 1H/1
PULPROG zgpg
TD 32768
SOLVENT CDCl3
NS 100
DS 0
SWH 45045.047 Hz
FIDRES 1.374666 Hz
AQ 0.3637748 sec
RG 2048
DW 11.100 usec
DE 6.50 usec
TE 294.7 K
D1 3.50000000 sec
d11 0.03000000 sec
DELTA 3.40000010 sec
MCREST 0 sec
MCWRK 0.01500000 sec
======== CHANNEL f1 ========
NUC1 13C
P1 4.80 usec
PL1 0 dB
SFO1 150.5346470 MHz
======== CHANNEL f2 ========
CPDPRG2 waltz16
NUC2 1H
PCPD2 92.00 usec
PL2 120.00 dB
PL12 9.00 dB
PL13 14.00 dB
SFO2 598.6029930 MHz
F2 - Processing parameters
SI 65536
SF 150.5181035 MHz
WDW EM
SSB 0
LB 3.00 Hz
GB 0
PC 1.00
COOEt
COOEt
Ph
N
Ph
5f
190
180
170
160
150
140
130
120
110
100
90
80
70
60
50
40
30
20
ppm

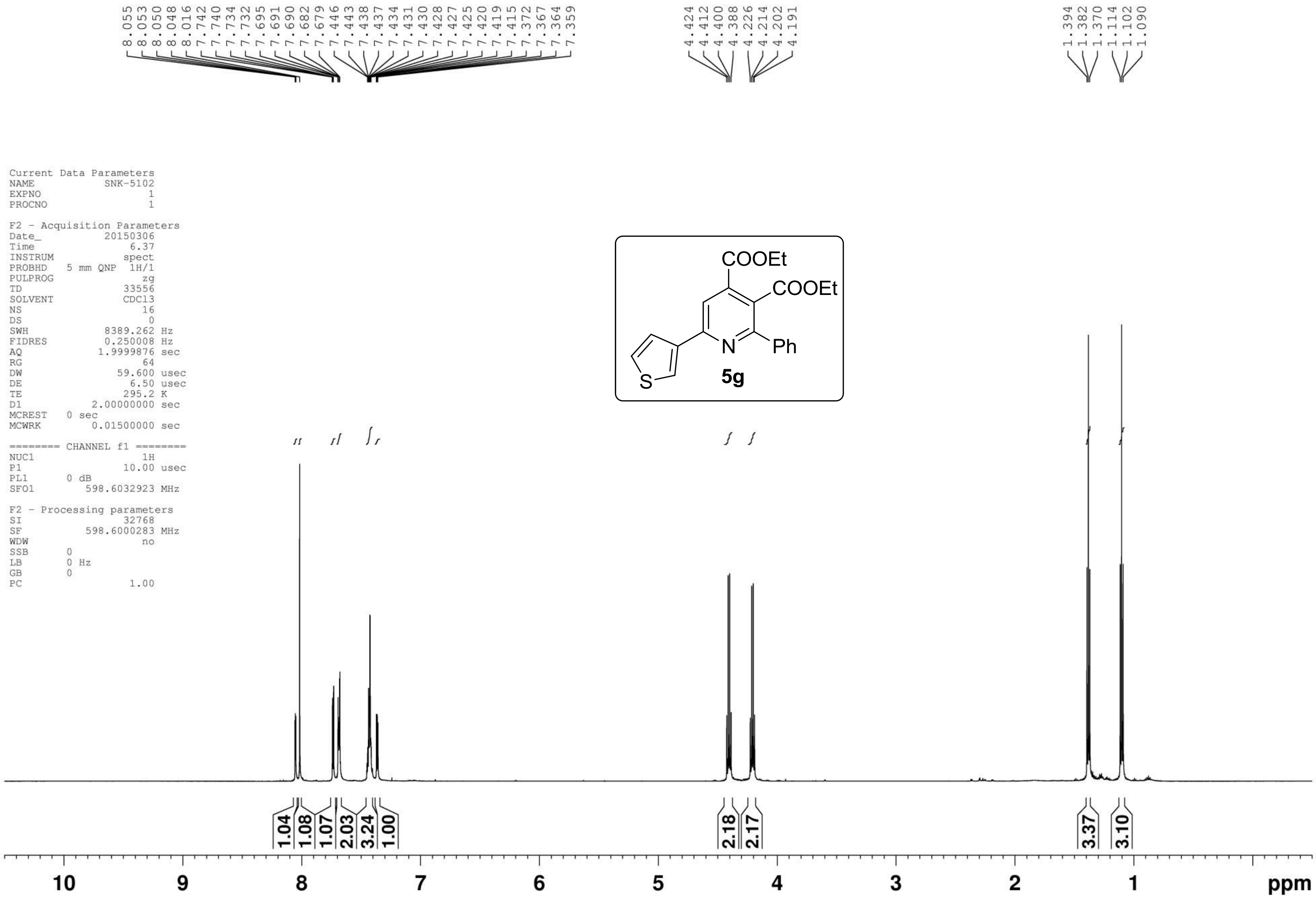

8.055 8.053 8.050 8.048 8.016 7.742 7.740 7.734 7.732 7.695 7.691 7.690 7.682 7.679 7.446 7.443 7.438 7.437 7.434 7.431 7.430 7.428 7.427 7.425 7.420 7.419 7.415 7.372 7.367 7.364 7.359
4.424 4.412 4.400 4.388 4.226 4.214 4.202 4.191
1.394 1.382 1.370 1.114 1.102 1.090
Current Data Parameters
NAME SNK-5102
EXPNO 1
PROCNO 1
F2 - Acquisition Parameters
Date_ 20150306
Time 6.37
INSTRUM spect
PROBHD 5 mm QNP 1H/1
PULPROG zg
TD 33556
SOLVENT CDCl3
NS 16
DS 0
SWH 8389.262 Hz
FIDRES 0.250008 Hz
AQ 1.9999876 sec
RG 64
DW 59.600 usec
DE 6.50 usec
TE 295.2 K
D1 2.00000000 sec
MCREST 0 sec
MCWRK 0.01500000 sec
======== CHANNEL f1 ========
NUC1 1H
P1 10.00 usec
PL1 0 dB
SFO1 598.6032923 MHz
F2 - Processing parameters
SI 32768
SF 598.6000283 MHz
WDW no
SSB 0
LB 0 Hz
GB 0
PC 1.00
COOEt
COOEt
N
Ph
S
5g
1.04 1.08 1.07 2.03 3.24 1.00
2.18 2.17
3.37 3.10
10 9 8 7 6 5 4 3 2 1 ppm

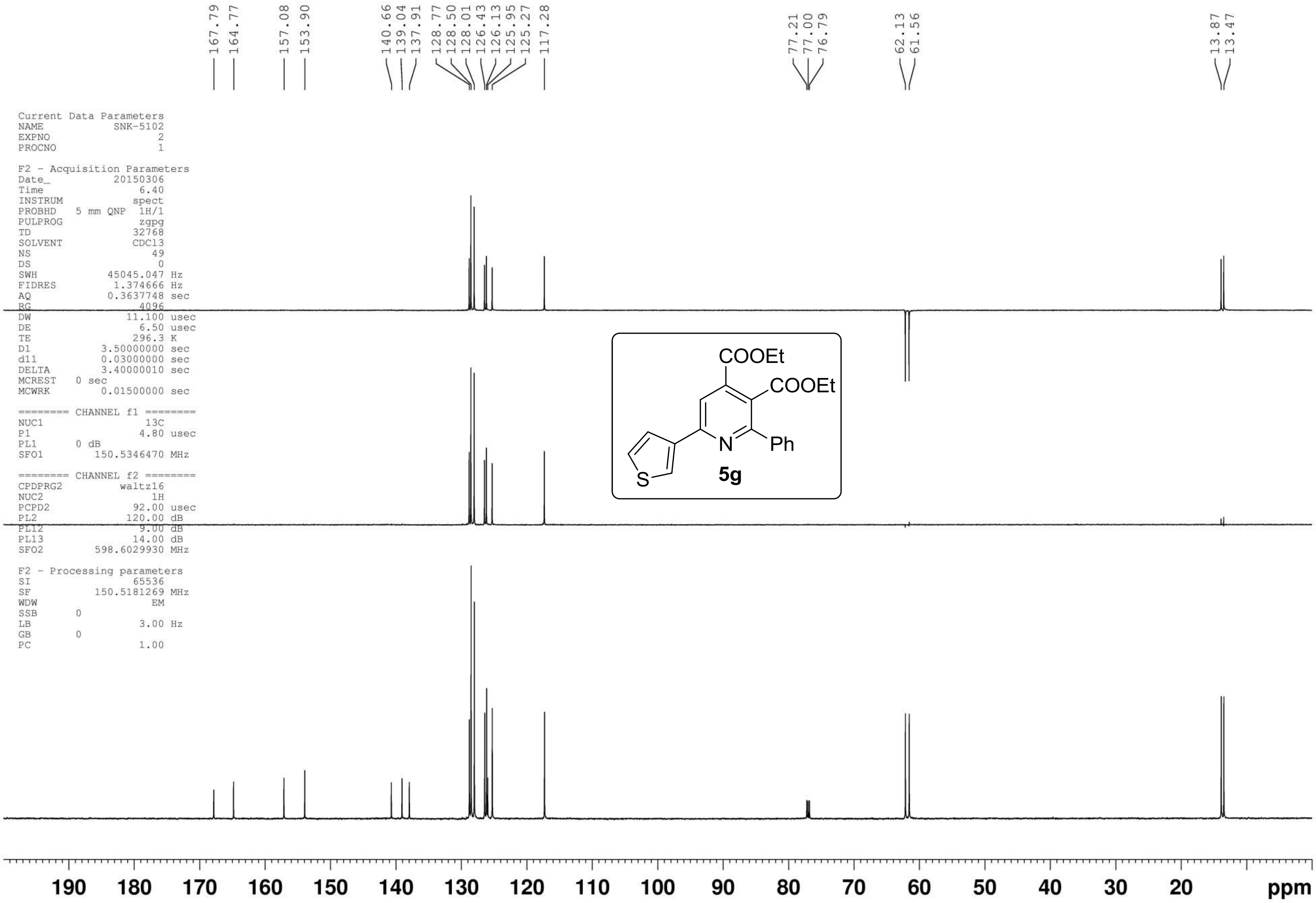
167.79
164.77
157.08
153.90
140.66
139.04
137.91
128.77
128.50
128.01
126.43
126.13
125.95
125.27
117.28
77.21
77.00
76.79
62.13
61.56
13.87
13.47
Current Data Parameters
NAME SNK-5102
EXPNO 2
PROCNO 1
F2 - Acquisition Parameters
Date_ 20150306
Time 6.40
INSTRUM spect
PROBHD 5 mm QNP 1H/1
PULPROG zgpg
TD 32768
SOLVENT CDCl3
NS 49
DS 0
SWH 45045.047 Hz
FIDRES 1.374666 Hz
AQ 0.3637748 sec
RG 4096
DW 11.100 usec
DE 6.50 usec
TE 296.3 K
D1 3.50000000 sec
d11 0.03000000 sec
DELTA 3.40000010 sec
MCREST 0 sec
MCWRK 0.01500000 sec
======== CHANNEL f1 ========
NUC1 13C
P1 4.80 usec
PL1 0 dB
SFO1 150.5346470 MHz
======== CHANNEL f2 ========
CPDPRG2 waltz16
NUC2 1H
PCPD2 92.00 usec
PL2 120.00 dB
PL12 9.00 dB
PL13 14.00 dB
SFO2 598.6029930 MHz
F2 - Processing parameters
SI 65536
SF 150.5181269 MHz
WDW EM
SSB 0
LB 3.00 Hz
GB 0
PC 1.00
COOEt
COOEt
N
Ph
S
5g
190 180 170 160 150 140 130 120 110 100 90 80 70 60 50 40 30 20 ppm

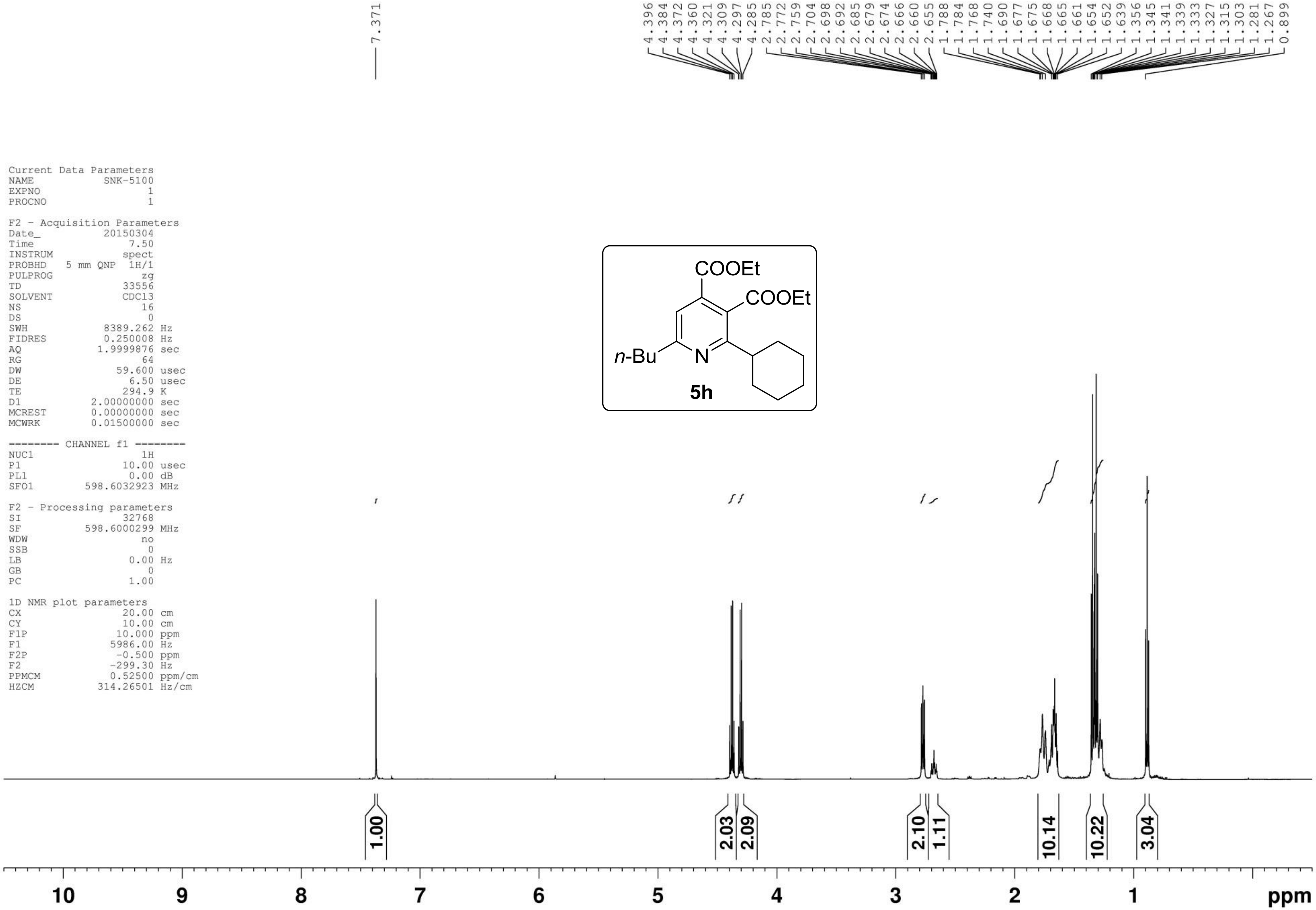
7.371
4.396 4.384 4.372 4.360 4.321 4.309 4.297 4.285 2.785 2.772 2.759 2.704 2.698 2.692 2.685 2.679 2.674 2.666 2.660 2.655 1.788 1.784 1.768 1.740 1.690 1.677 1.675 1.668 1.665 1.661 1.654 1.652 1.639 1.356 1.345 1.341 1.339 1.333 1.327 1.315 1.303 1.281 1.267 0.899
Current Data Parameters
NAME SNK-5100
EXPNO 1
PROCNO 1
F2 - Acquisition Parameters
Date_ 20150304
Time 7.50
INSTRUM spect
PROBHD 5 mm QNP 1H/1
PULPROG zg
TD 33556
SOLVENT CDCl3
NS 16
DS 0
SWH 8389.262 Hz
FIDRES 0.250008 Hz
AQ 1.9999876 sec
RG 64
DW 59.600 usec
DE 6.50 usec
TE 294.9 K
D1 2.00000000 sec
MCREST 0.00000000 sec
MCWRK 0.01500000 sec
======== CHANNEL f1 ========
NUC1 1H
P1 10.00 usec
PL1 0.00 dB
SFO1 598.6032923 MHz
F2 - Processing parameters
SI 32768
SF 598.6000299 MHz
WDW no
SSB 0
LB 0.00 Hz
GB 0
PC 1.00
1D NMR plot parameters
CX 20.00 cm
CY 10.00 cm
F1P 10.000 ppm
F1 5986.00 Hz
F2P -0.500 ppm
F2 -299.30 Hz
PPMCM 0.52500 ppm/cm
HZCM 314.26501 Hz/cm
COOEt
COOEt
n-Bu
N
5h
1.00
2.03
2.09
2.10
1.11
10.14
10.22
3.04
10
9
8
7
6
5
4
3
2
1
ppm

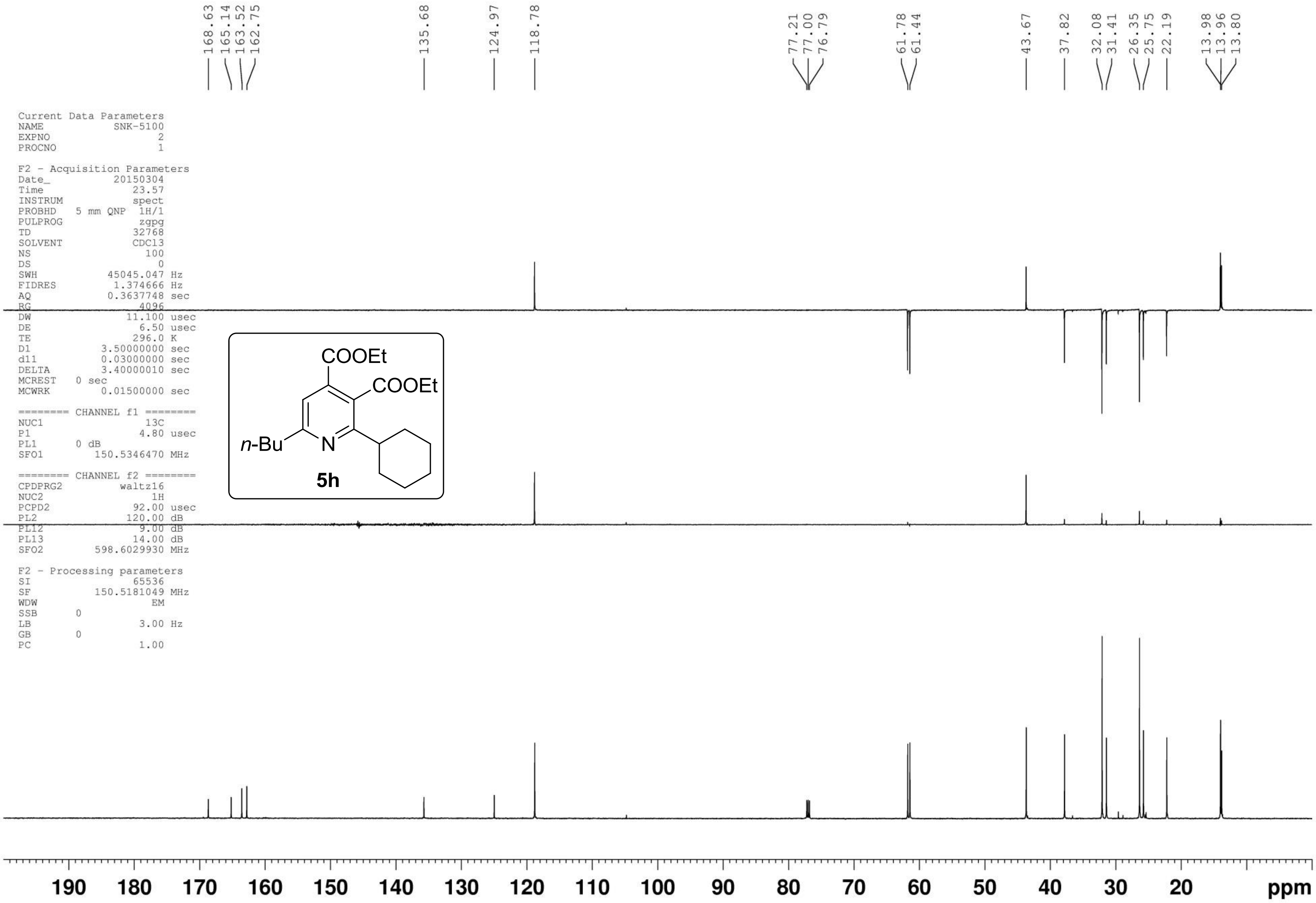
168.63
165.14
163.52
162.75
135.68
124.97
118.78
77.21
77.00
76.79
61.78
61.44
43.67
37.82
32.08
31.41
26.35
25.75
22.19
13.98
13.96
13.80
Current Data Parameters
NAME SNK-5100
EXPNO 2
PROCNO 1
F2 - Acquisition Parameters
Date_ 20150304
Time 23.57
INSTRUM spect
PROBHD 5 mm QNP 1H/1
PULPROG zgpg
TD 32768
SOLVENT CDCl3
NS 100
DS 0
SWH 45045.047 Hz
FIDRES 1.374666 Hz
AQ 0.3637748 sec
RG 4096
DW 11.100 usec
DE 6.50 usec
TE 296.0 K
D1 3.50000000 sec
d11 0.03000000 sec
DELTA 3.40000010 sec
MCREST 0 sec
MCWRK 0.01500000 sec
======== CHANNEL f1 ========
NUC1 13C
P1 4.80 usec
PL1 0 dB
SFO1 150.5346470 MHz
======== CHANNEL f2 ========
CPDPRG2 waltz16
NUC2 1H
PCPD2 92.00 usec
PL2 120.00 dB
PL12 9.00 dB
PL13 14.00 dB
SFO2 598.6029930 MHz
F2 - Processing parameters
SI 65536
SF 150.5181049 MHz
WDW EM
SSB 0
LB 3.00 Hz
GB 0
PC 1.00
190 180 170 160 150 140 130 120 110 100 90 80 70 60 50 40 30 20 ppm
COOEt
COOEt
n-Bu
N

5h

Current Data Parameters
NAME SNK-5097
EXPNO 1
PROCNO 1

F2 - Acquisition Parameters
Date_ 20150302
Time 10.58
INSTRUM spect
PROBHD 5 mm QNP 1H/1
PULPROG zg
TD 33556
SOLVENT CDCl3
NS 16
DS 0
SWH 8389.262 Hz
FIDRES 0.250008 Hz
AQ 1.9999876 sec
RG 32
DW 59.600 usec
DE 6.50 usec
TE 294.0 K
D1 2.00000000 sec
MCREST 0.00000000 sec
MCWRK 0.01500000 sec

======== CHANNEL f1 ========
NUC1 1H
P1 10.00 usec
PL1 0.00 dB
SFO1 598.6032923 MHz

F2 - Processing parameters
SI 32768
SF 598.6000286 MHz
WDW no
SSB 0
LB 0.00 Hz
GB 0
PC 1.00

1D NMR plot parameters
CX 20.00 cm
CY 8.00 cm
F1P 10.000 ppm
F1 5986.00 Hz
F2P -0.500 ppm
F2 -299.30 Hz
PPMCM 0.52500 ppm/cm
HZCM 314.26501 Hz/cm

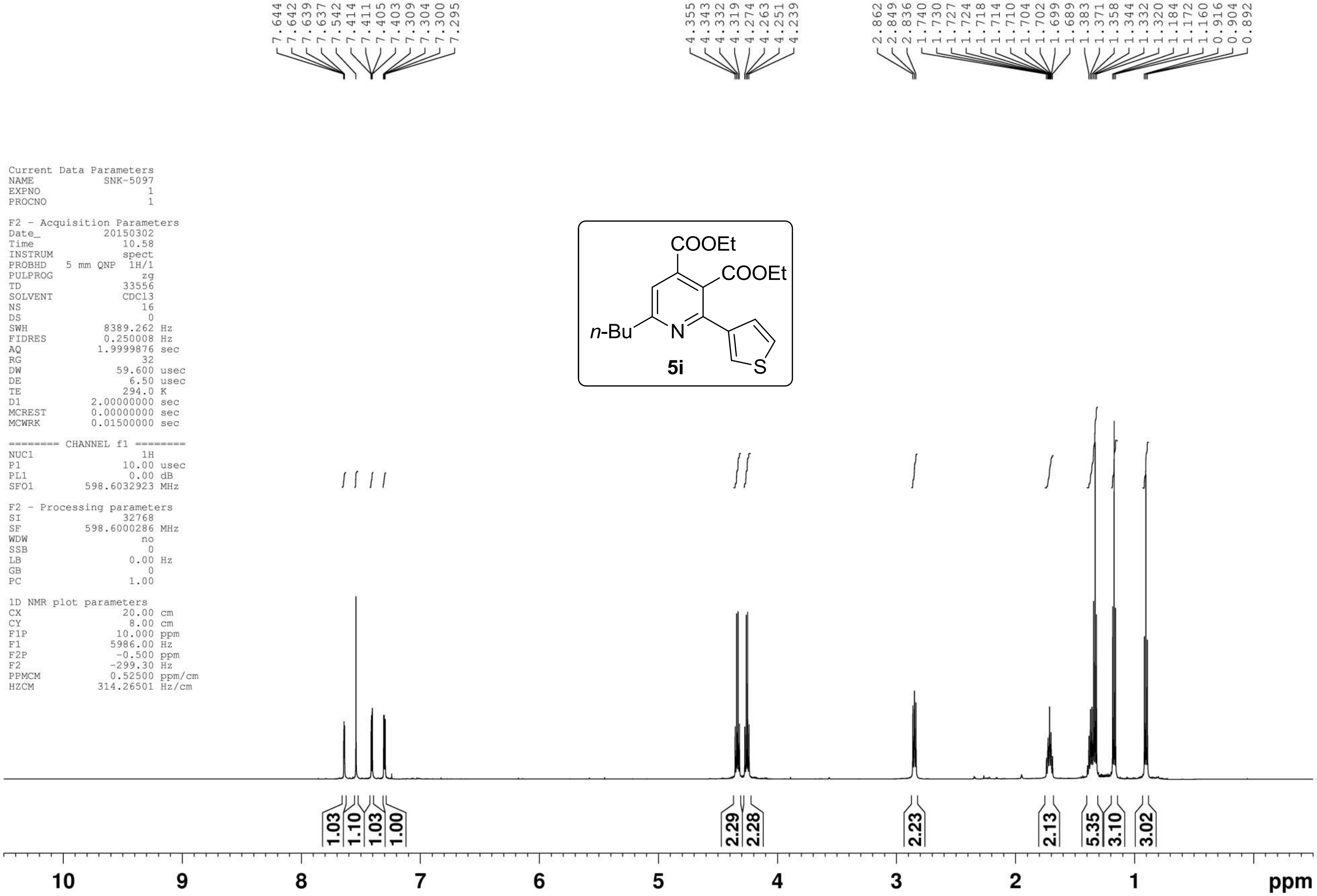

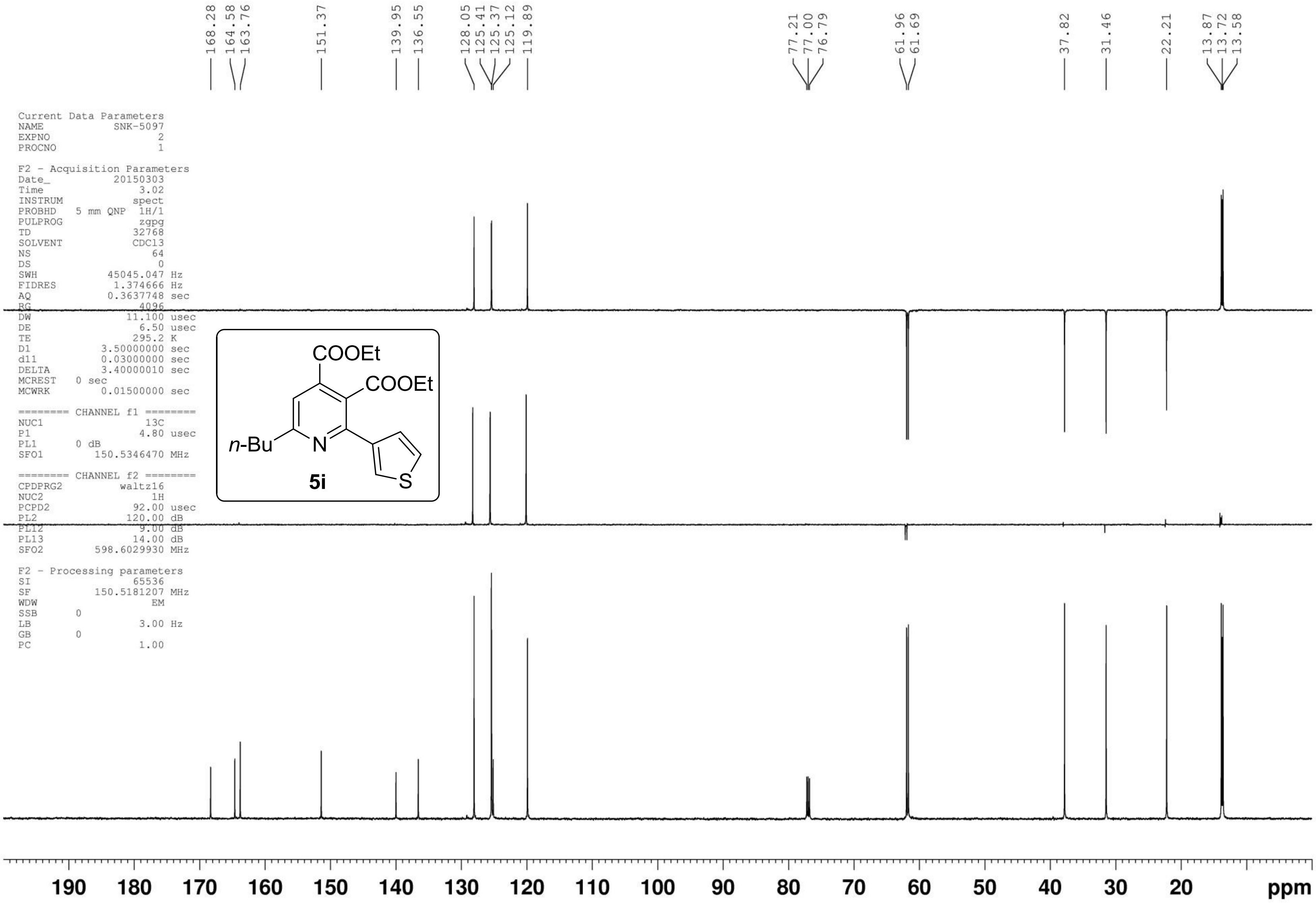
168.28
164.58
163.76
151.37
139.95
136.55
128.05
125.41
125.37
125.12
119.89
77.21
77.00
76.79
61.96
61.69
37.82
31.46
22.21
13.87
13.72
13.58
Current Data Parameters
NAME SNK-5097
EXPNO 2
PROCNO 1
F2 - Acquisition Parameters
Date_ 20150303
Time 3.02
INSTRUM spect
PROBHD 5 mm QNP 1H/1
PULPROG zgpg
TD 32768
SOLVENT CDCl3
NS 64
DS 0
SWH 45045.047 Hz
FIDRES 1.374666 Hz
AQ 0.3637748 sec
RG 4096
DW 11.100 usec
DE 6.50 usec
TE 295.2 K
D1 3.50000000 sec
d11 0.03000000 sec
DELTA 3.40000010 sec
MCREST 0 sec
MCWRK 0.01500000 sec
======== CHANNEL f1 ========
NUC1 13C
P1 4.80 usec
PL1 0 dB
SFO1 150.5346470 MHz
======== CHANNEL f2 ========
CPDPRG2 waltz16
NUC2 1H
PCPD2 92.00 usec
PL2 120.00 dB
PL12 9.00 dB
PL13 14.00 dB
SFO2 598.6029930 MHz
F2 - Processing parameters
SI 65536
SF 150.5181207 MHz
WDW EM
SSB 0
LB 3.00 Hz
GB 0
PC 1.00
COOEt
COOEt
n-Bu
N
S
5i
190
180
170
160
150
140
130
120
110
100
90
80
70
60
50
40
30
20
ppm

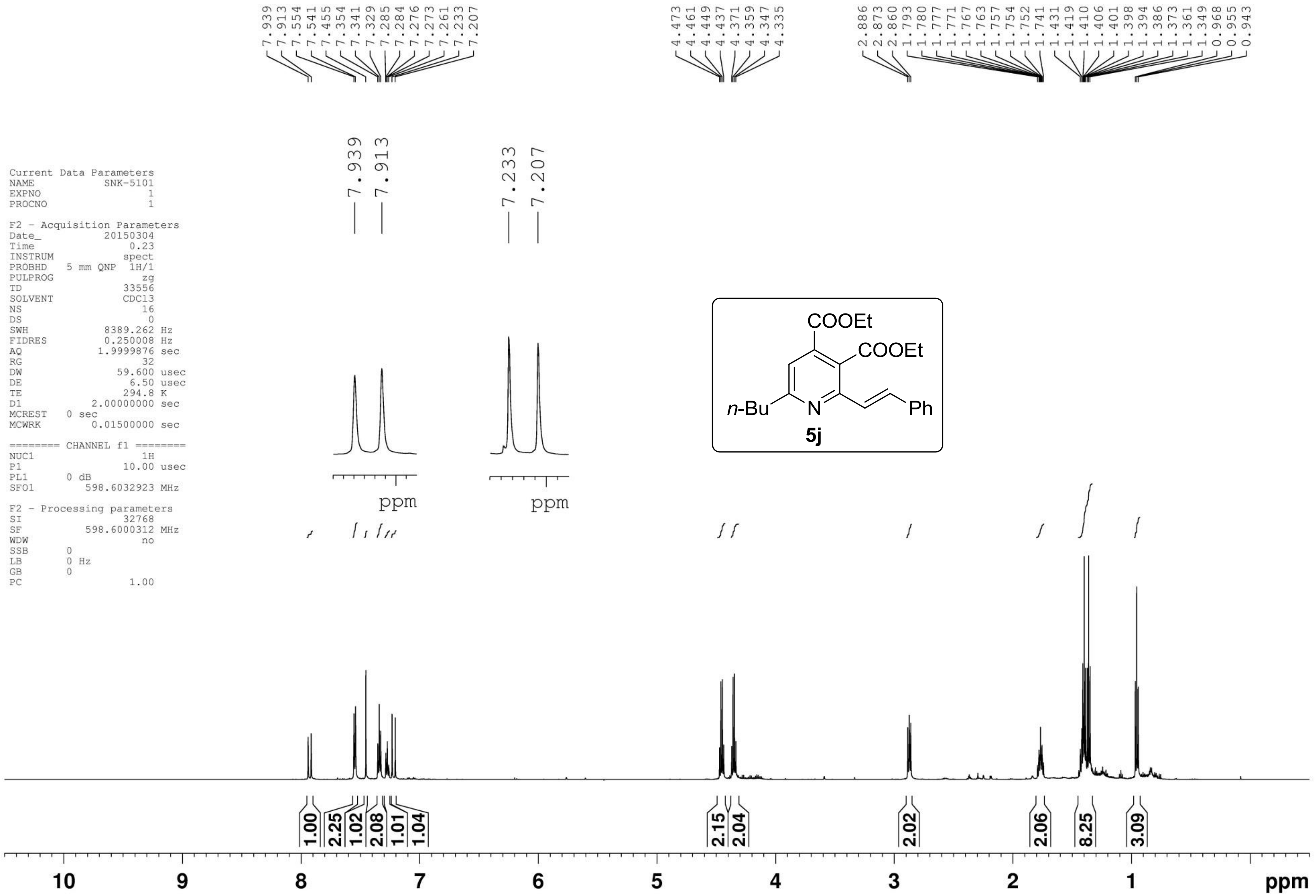
7.939 7.913 7.554 7.541 7.455 7.354 7.341 7.329 7.285 7.284 7.276 7.273 7.261 7.233 7.207
4.473 4.461 4.449 4.437 4.371 4.359 4.347 4.335
2.886 2.873 2.860 1.793 1.780 1.777 1.771 1.767 1.763 1.757 1.754 1.752 1.741 1.431 1.419 1.410 1.406 1.401 1.398 1.394 1.386 1.373 1.361 1.349 0.968 0.955 0.943
7.939
7.913
7.233
7.207
ppm
ppm
Current Data Parameters
NAME SNK-5101
EXPNO 1
PROCNO 1
F2 - Acquisition Parameters
Date_ 20150304
Time 0.23
INSTRUM spect
PROBHD 5 mm QNP 1H/1
PULPROG zg
TD 33556
SOLVENT CDCl3
NS 16
DS 0
SWH 8389.262 Hz
FIDRES 0.250008 Hz
AQ 1.9999876 sec
RG 32
DW 59.600 usec
DE 6.50 usec
TE 294.8 K
D1 2.00000000 sec
MCREST 0 sec
MCWRK 0.01500000 sec
======== CHANNEL f1 ========
NUC1 1H
P1 10.00 usec
PL1 0 dB
SFO1 598.6032923 MHz
F2 - Processing parameters
SI 32768
SF 598.6000312 MHz
WDW no
SSB 0
LB 0 Hz
GB 0
PC 1.00
COOEt
COOEt
n-Bu
N
Ph
5j
1.00 2.25 1.02 2.08 1.01 1.04
2.15 2.04
2.02
2.06 8.25 3.09
10 9 8 7 6 5 4 3 2 1 ppm

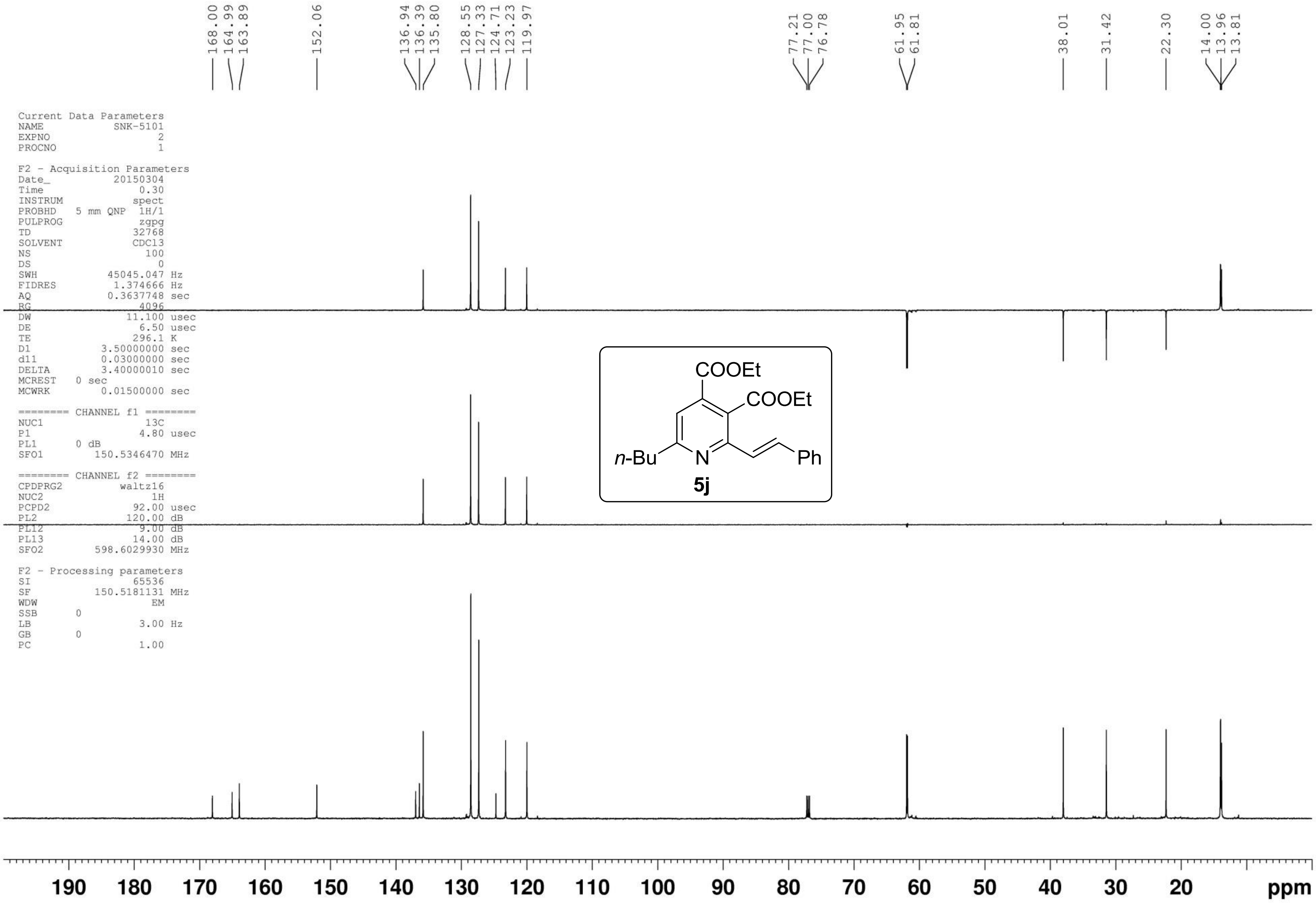
168.00
164.99
163.89
152.06
136.94
136.39
135.80
128.55
127.33
124.71
123.23
119.97
77.21
77.00
76.78
61.95
61.81
38.01
31.42
22.30
14.00
13.96
13.81
Current Data Parameters
NAME SNK-5101
EXPNO 2
PROCNO 1
F2 - Acquisition Parameters
Date_ 20150304
Time 0.30
INSTRUM spect
PROBHD 5 mm QNP 1H/1
PULPROG zgpg
TD 32768
SOLVENT CDCl3
NS 100
DS 0
SWH 45045.047 Hz
FIDRES 1.374666 Hz
AQ 0.3637748 sec
RG 4096
DW 11.100 usec
DE 6.50 usec
TE 296.1 K
D1 3.50000000 sec
d11 0.03000000 sec
DELTA 3.40000010 sec
MCREST 0 sec
MCWRK 0.01500000 sec
======== CHANNEL f1 ========
NUC1 13C
P1 4.80 usec
PL1 0 dB
SFO1 150.5346470 MHz
======== CHANNEL f2 ========
CPDPRG2 waltz16
NUC2 1H
PCPD2 92.00 usec
PL2 120.00 dB
PL12 9.00 dB
PL13 14.00 dB
SFO2 598.6029930 MHz
F2 - Processing parameters
SI 65536
SF 150.5181131 MHz
WDW EM
SSB 0
LB 3.00 Hz
GB 0
PC 1.00
COOEt
COOEt
n-Bu
N
Ph
5j
190
180
170
160
150
140
130
120
110
100
90
80
70
60
50
40
30
20
ppm

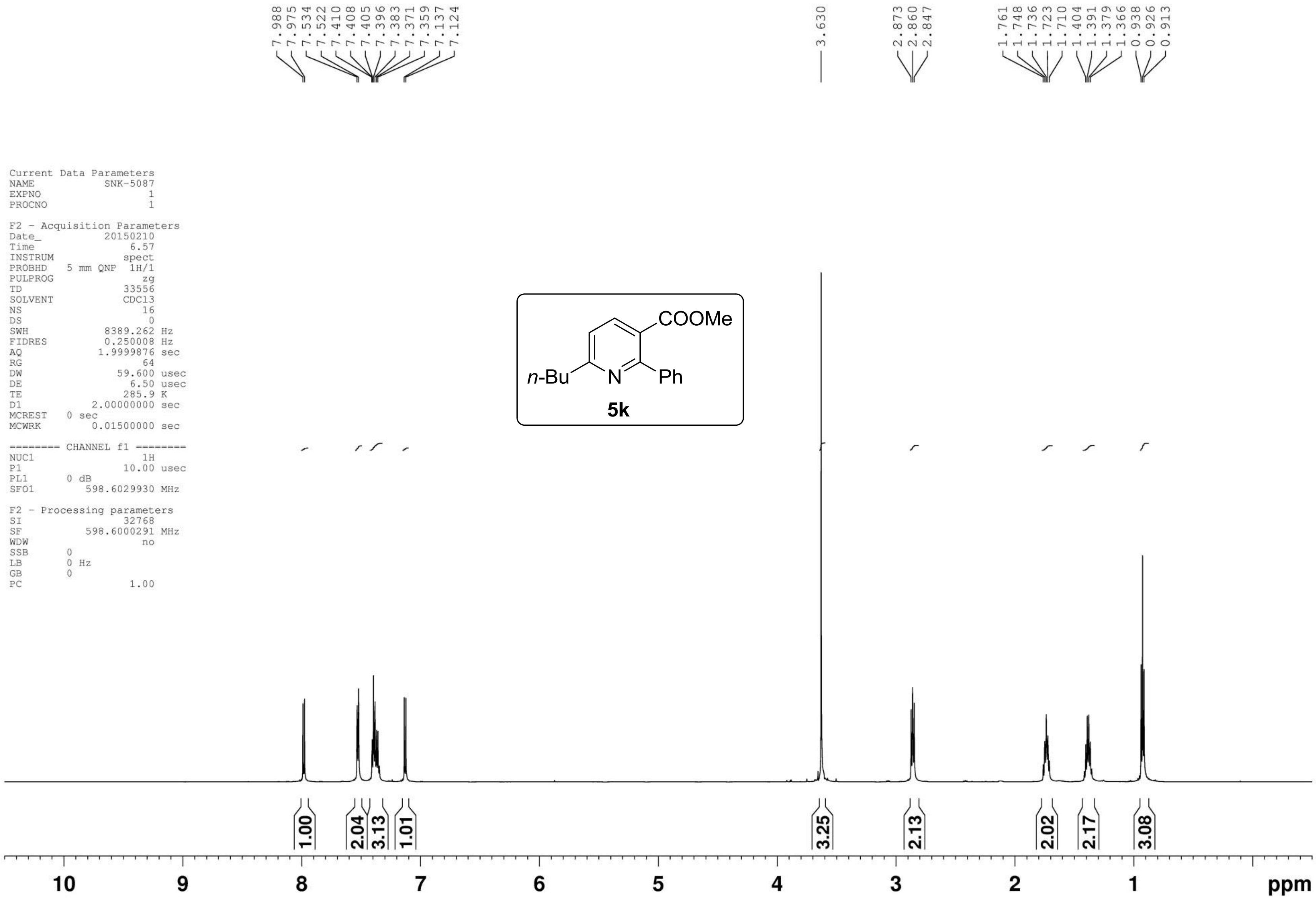
7.988
7.975
7.534
7.522
7.410
7.408
7.405
7.396
7.383
7.371
7.359
7.137
7.124
3.630
2.873
2.860
2.847
1.761
1.748
1.736
1.723
1.710
1.404
1.391
1.379
1.366
0.938
0.926
0.913
Current Data Parameters
NAME SNK-5087
EXPNO 1
PROCNO 1
F2 - Acquisition Parameters
Date_ 20150210
Time 6.57
INSTRUM spect
PROBHD 5 mm QNP 1H/1
PULPROG zg
TD 33556
SOLVENT CDCl3
NS 16
DS 0
SWH 8389.262 Hz
FIDRES 0.250008 Hz
AQ 1.9999876 sec
RG 64
DW 59.600 usec
DE 6.50 usec
TE 285.9 K
D1 2.00000000 sec
MCREST 0 sec
MCWRK 0.01500000 sec
======== CHANNEL f1 ========
NUC1 1H
P1 10.00 usec
PL1 0 dB
SFO1 598.6029930 MHz
F2 - Processing parameters
SI 32768
SF 598.6000291 MHz
WDW no
SSB 0
LB 0 Hz
GB 0
PC 1.00
COOMe
n-Bu
N
Ph
5k
1.00
2.04
3.13
1.01
3.25
2.13
2.02
2.17
3.08
10
9
8
7
6
5
4
3
2
1
ppm

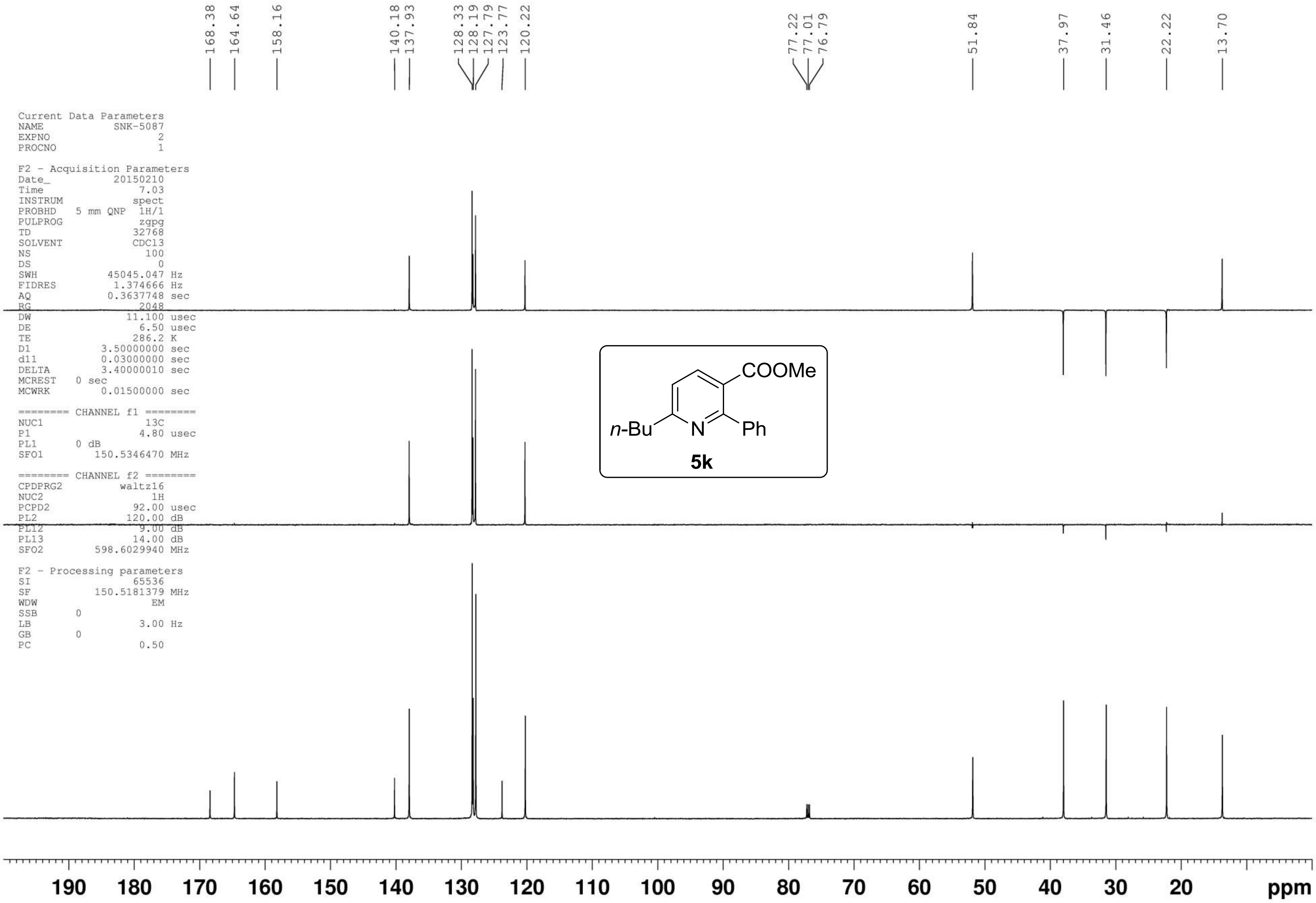
168.38
164.64
158.16
140.18
137.93
128.33
128.19
127.79
123.77
120.22
77.22
77.01
76.79
51.84
37.97
31.46
22.22
13.70
Current Data Parameters
NAME SNK-5087
EXPNO 2
PROCNO 1
F2 - Acquisition Parameters
Date_ 20150210
Time 7.03
INSTRUM spect
PROBHD 5 mm QNP 1H/1
PULPROG zgpg
TD 32768
SOLVENT CDCl3
NS 100
DS 0
SWH 45045.047 Hz
FIDRES 1.374666 Hz
AQ 0.3637748 sec
RG 2048
DW 11.100 usec
DE 6.50 usec
TE 286.2 K
D1 3.50000000 sec
d11 0.03000000 sec
DELTA 3.40000010 sec
MCREST 0 sec
MCWRK 0.01500000 sec
======== CHANNEL f1 ========
NUC1 13C
P1 4.80 usec
PL1 0 dB
SFO1 150.5346470 MHz
======== CHANNEL f2 ========
CPDPRG2 waltz16
NUC2 1H
PCPD2 92.00 usec
PL2 120.00 dB
PL12 9.00 dB
PL13 14.00 dB
SFO2 598.6029940 MHz
F2 - Processing parameters
SI 65536
SF 150.5181379 MHz
WDW EM
SSB 0
LB 3.00 Hz
GB 0
PC 0.50
COOMe
n-Bu
N
Ph
5k
190 180 170 160 150 140 130 120 110 100 90 80 70 60 50 40 30 20 ppm

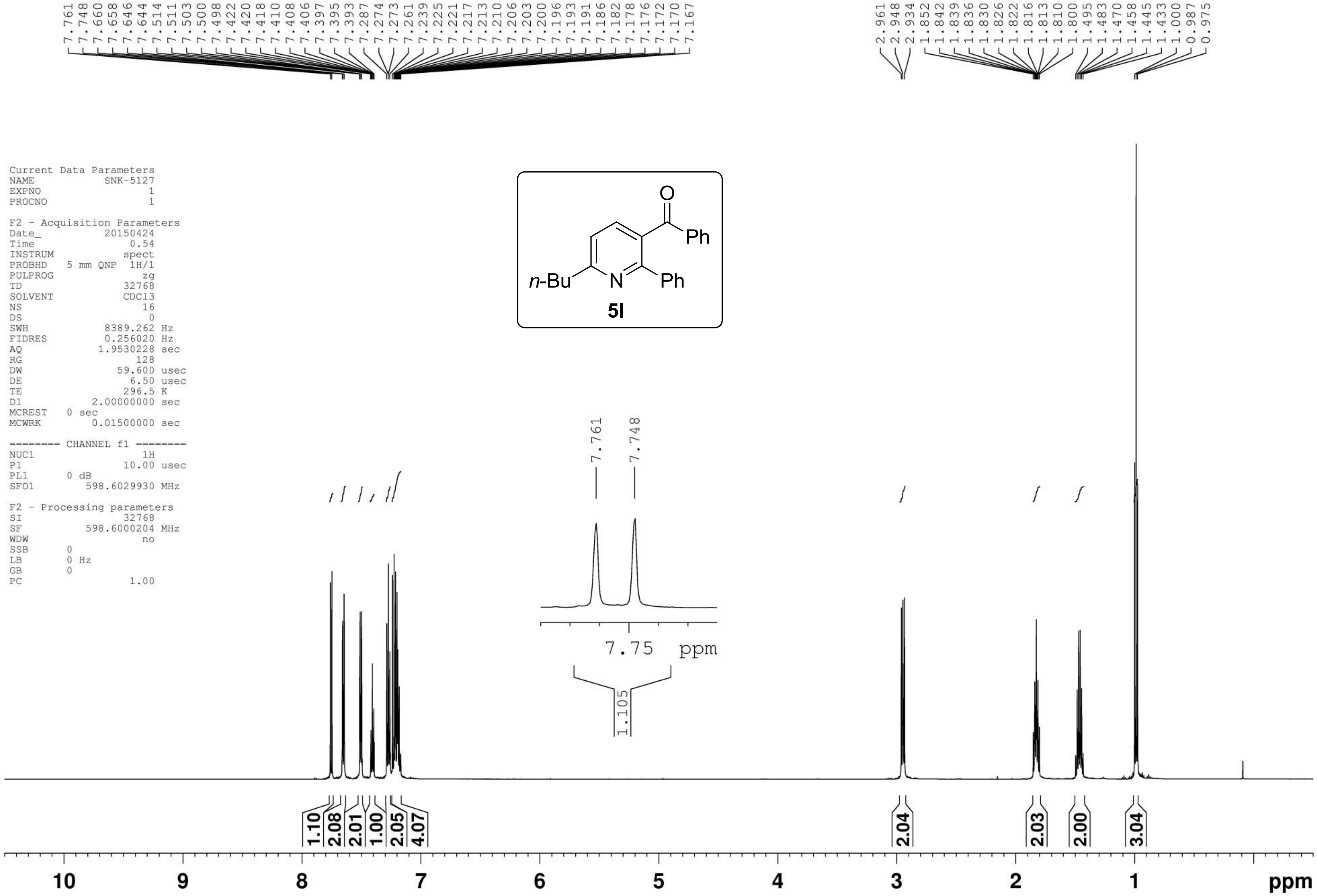
7.761 7.748 7.660 7.658 7.646 7.644 7.514 7.511 7.503 7.500 7.498 7.422 7.420 7.418 7.410 7.408 7.406 7.397 7.395 7.393 7.287 7.274 7.273 7.261 7.239 7.225 7.221 7.217 7.213 7.210 7.206 7.203 7.200 7.196 7.193 7.191 7.186 7.182 7.178 7.176 7.172 7.170 7.167
2.961 2.948 2.934 1.852 1.842 1.839 1.836 1.830 1.826 1.822 1.816 1.813 1.810 1.800 1.495 1.483 1.470 1.458 1.445 1.433 1.000 0.987 0.975
Current Data Parameters
NAME SNK-5127
EXPNO 1
PROCNO 1
F2 - Acquisition Parameters
Date_ 20150424
Time 0.54
INSTRUM spect
PROBHD 5 mm QNP 1H/1
PULPROG zg
TD 32768
SOLVENT CDCl3
NS 16
DS 0
SWH 8389.262 Hz
FIDRES 0.256020 Hz
AQ 1.9530228 sec
RG 128
DW 59.600 usec
DE 6.50 usec
TE 296.5 K
D1 2.00000000 sec
MCREST 0 sec
MCWRK 0.01500000 sec
======== CHANNEL f1 ========
NUC1 1H
P1 10.00 usec
PL1 0 dB
SFO1 598.6029930 MHz
F2 - Processing parameters
SI 32768
SF 598.6000204 MHz
WDW no
SSB 0
LB 0 Hz
GB 0
PC 1.00
O
Ph
n-Bu
N
Ph
5l
7.761
7.748
7.75 ppm
1.105
1.10 2.08 2.01 1.00 2.05 4.07
2.04
2.03
2.00
3.04
10 9 8 7 6 5 4 3 2 1 ppm

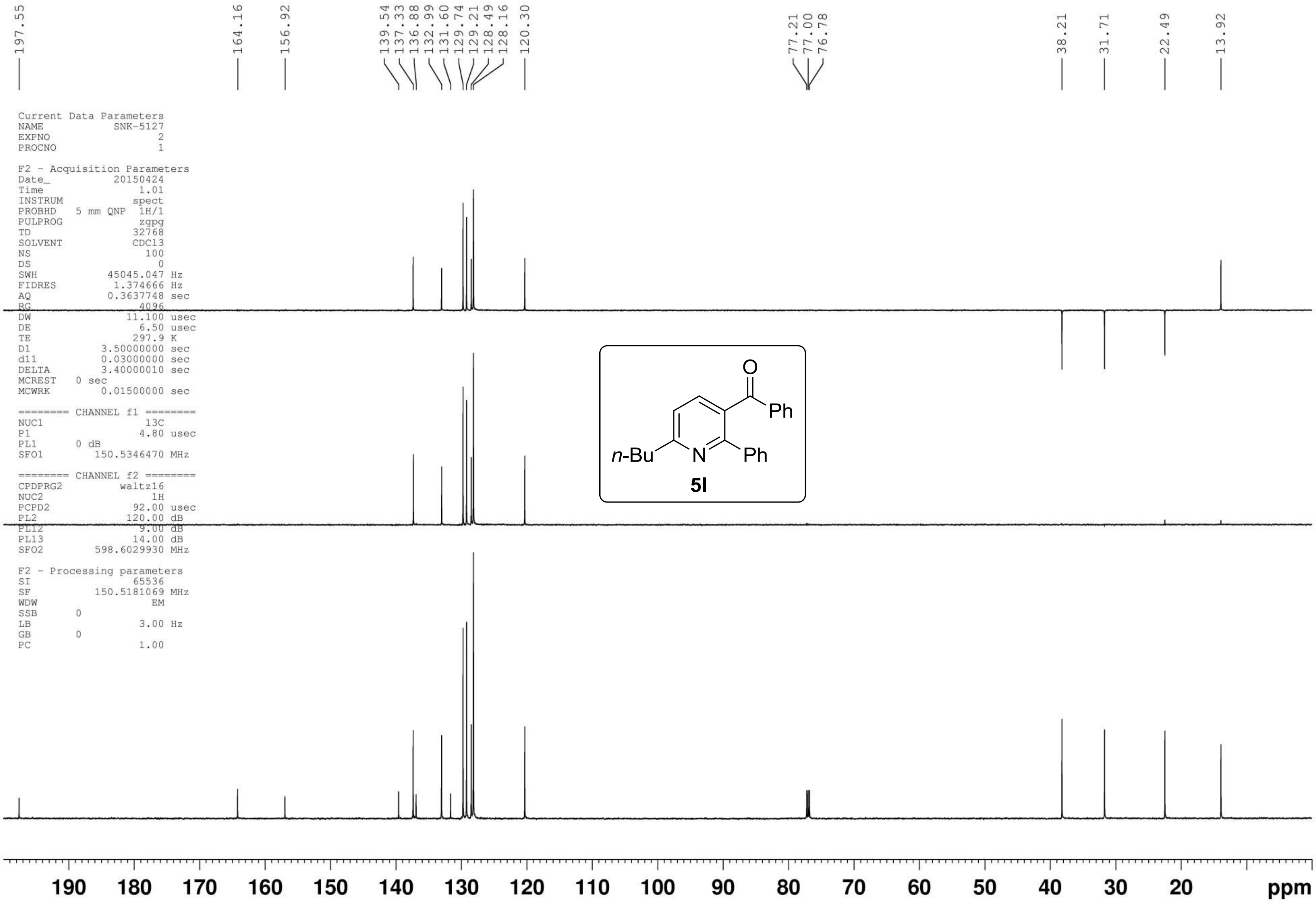
197.55
164.16
156.92
139.54
137.33
136.88
132.99
131.60
129.74
129.21
128.49
128.16
120.30
77.21
77.00
76.78
38.21
31.71
22.49
13.92
Current Data Parameters
NAME SNK-5127
EXPNO 2
PROCNO 1
F2 - Acquisition Parameters
Date_ 20150424
Time 1.01
INSTRUM spect
PROBHD 5 mm QNP 1H/1
PULPROG zgpg
TD 32768
SOLVENT CDCl3
NS 100
DS 0
SWH 45045.047 Hz
FIDRES 1.374666 Hz
AQ 0.3637748 sec
RG 4096
DW 11.100 usec
DE 6.50 usec
TE 297.9 K
D1 3.50000000 sec
d11 0.03000000 sec
DELTA 3.40000010 sec
MCREST 0 sec
MCWRK 0.01500000 sec
======== CHANNEL f1 ========
NUC1 13C
P1 4.80 usec
PL1 0 dB
SFO1 150.5346470 MHz
======== CHANNEL f2 ========
CPDPRG2 waltz16
NUC2 1H
PCPD2 92.00 usec
PL2 120.00 dB
PL12 9.00 dB
PL13 14.00 dB
SFO2 598.6029930 MHz
F2 - Processing parameters
SI 65536
SF 150.5181069 MHz
WDW EM
SSB 0
LB 3.00 Hz
GB 0
PC 1.00
O
Ph
n-Bu
N
Ph
5l
190 180 170 160 150 140 130 120 110 100 90 80 70 60 50 40 30 20 ppm

Current Data Parameters
NAME SNK-5086
EXPNO 1
PROCNO 1

F2 - Acquisition Parameters
Date_ 20150209
Time 23.40
INSTRUM spect
PROBHD 5 mm QNP 1H/1
PULPROG zg
TD 33556
SOLVENT CDCl3
NS 16
DS 0
SWH 8389.262 Hz
FIDRES 0.250008 Hz
AQ 1.9999876 sec
RG 64
DW 59.600 usec
DE 6.50 usec
TE 291.1 K
D1 2.00000000 sec
MCREST 0 sec
MCWRK 0.01500000 sec

======== CHANNEL f1 ========
NUC1 1H
P1 10.00 usec
PL1 0 dB
SFO1 598.6029930 MHz

F2 - Processing parameters
SI 32768
SF 598.6000296 MHz
WDW no
SSB 0
LB 0 Hz
GB 0
PC 1.00

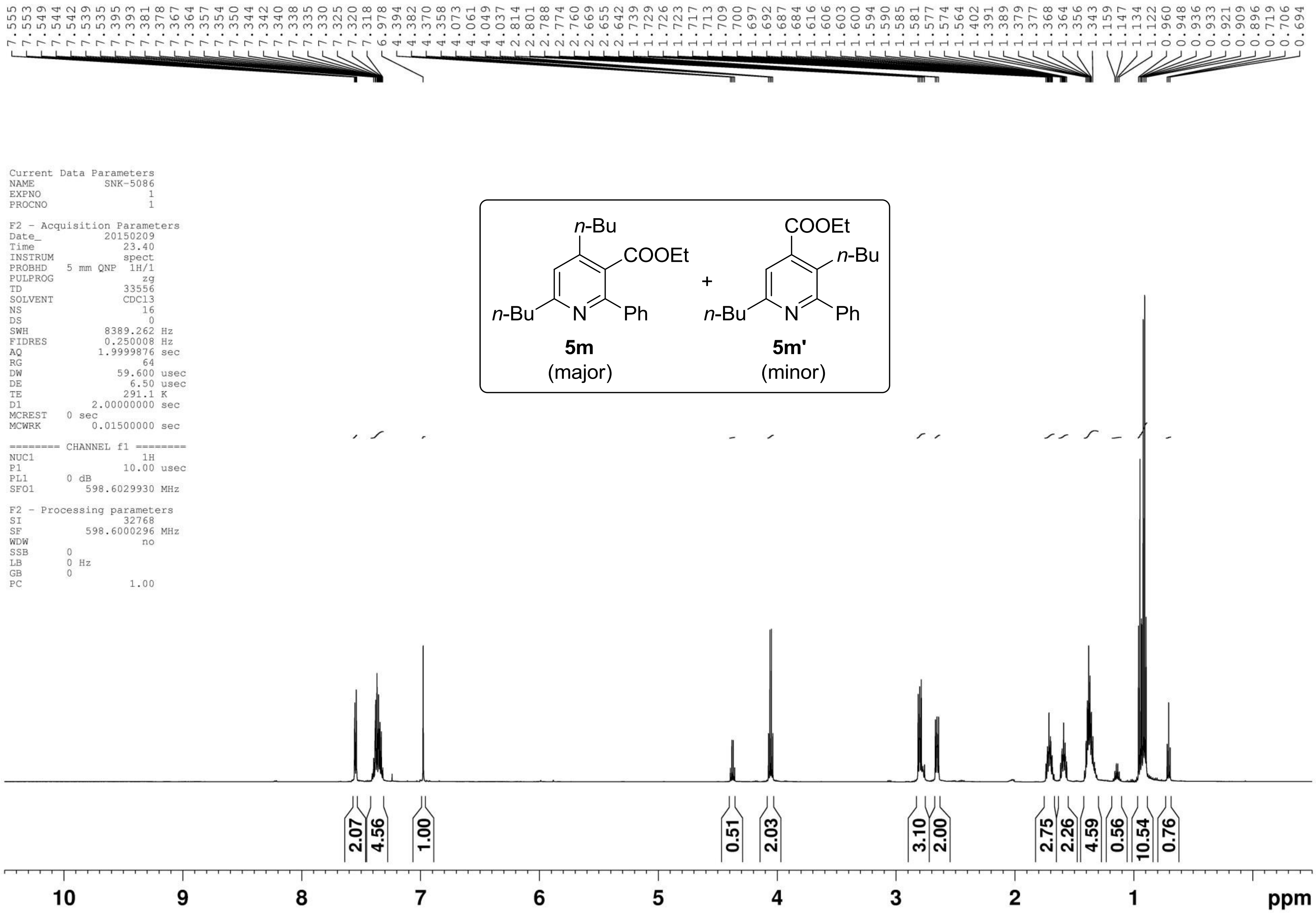

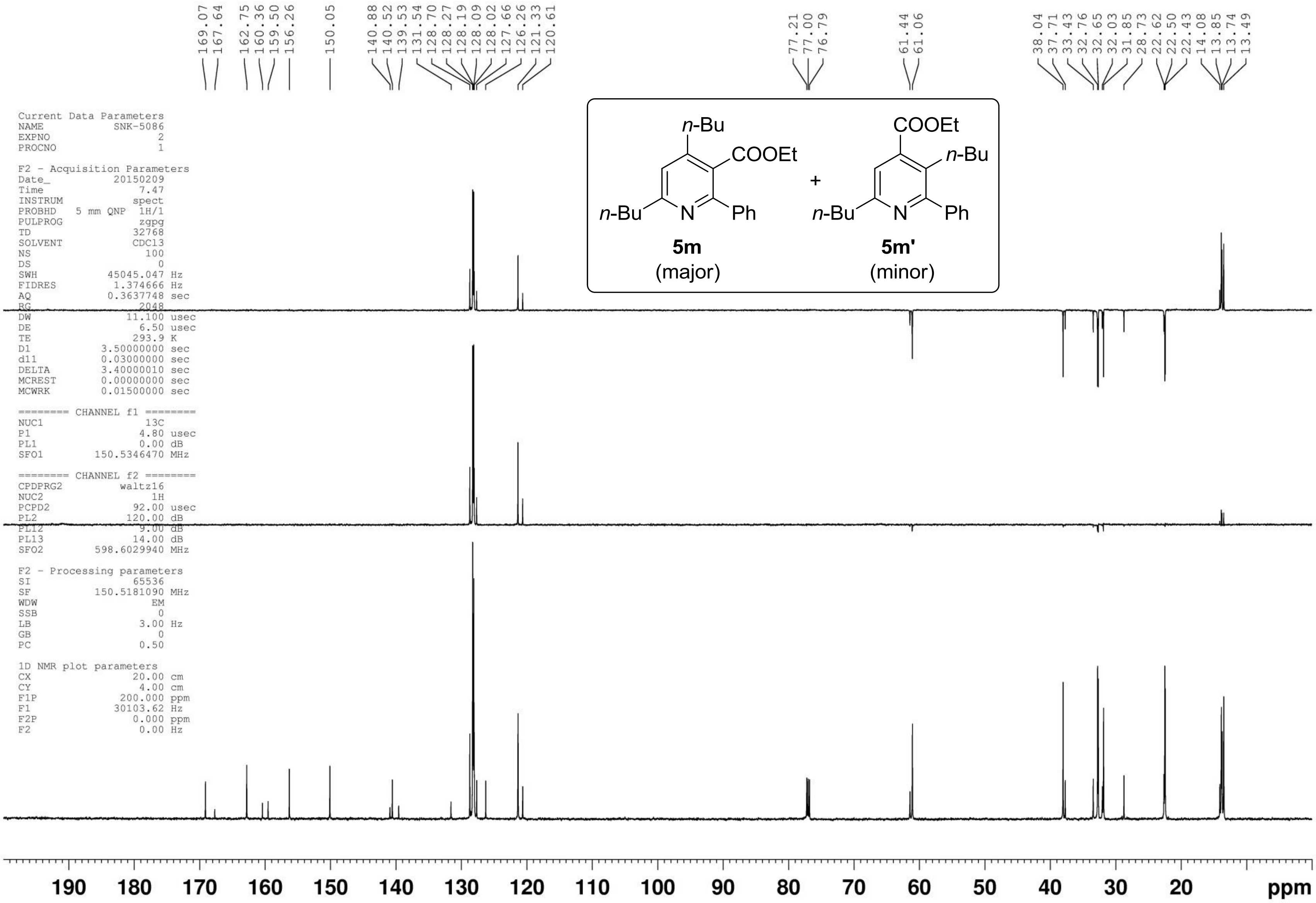
169.07
167.64
162.75
160.36
159.50
156.26
150.05
140.88
140.52
139.53
131.54
128.70
128.27
128.19
128.09
128.02
127.66
126.26
121.33
120.61
77.21
77.00
76.79
61.44
61.06
38.04
37.71
33.43
32.76
32.65
32.03
31.85
28.73
22.62
22.50
22.43
14.08
13.85
13.74
13.49
Current Data Parameters
NAME SNK-5086
EXPNO 2
PROCNO 1
F2 - Acquisition Parameters
Date_ 20150209
Time 7.47
INSTRUM spect
PROBHD 5 mm QNP 1H/1
PULPROG zgpg
TD 32768
SOLVENT CDCl3
NS 100
DS 0
SWH 45045.047 Hz
FIDRES 1.374666 Hz
AQ 0.3637748 sec
RG 2048
DW 11.100 usec
DE 6.50 usec
TE 293.9 K
D1 3.50000000 sec
d11 0.03000000 sec
DELTA 3.40000010 sec
MCREST 0.00000000 sec
MCWRK 0.01500000 sec
======== CHANNEL f1 ========
NUC1 13C
P1 4.80 usec
PL1 0.00 dB
SFO1 150.5346470 MHz
======== CHANNEL f2 ========
CPDPRG2 waltz16
NUC2 1H
PCPD2 92.00 usec
PL2 120.00 dB
PL12 9.00 dB
PL13 14.00 dB
SFO2 598.6029940 MHz
F2 - Processing parameters
SI 65536
SF 150.5181090 MHz
WDW EM
SSB 0
LB 3.00 Hz
GB 0
PC 0.50
1D NMR plot parameters
CX 20.00 cm
CY 4.00 cm
F1P 200.000 ppm
F1 30103.62 Hz
F2P 0.000 ppm
F2 0.00 Hz
n-Bu
COOEt
n-Bu
N
Ph
+
COOEt
n-Bu
n-Bu
N
Ph
5m
(major)
5m'
(minor)
190
180
170
160
150
140
130
120
110
100
90
80
70
60
50
40
30
20
ppm

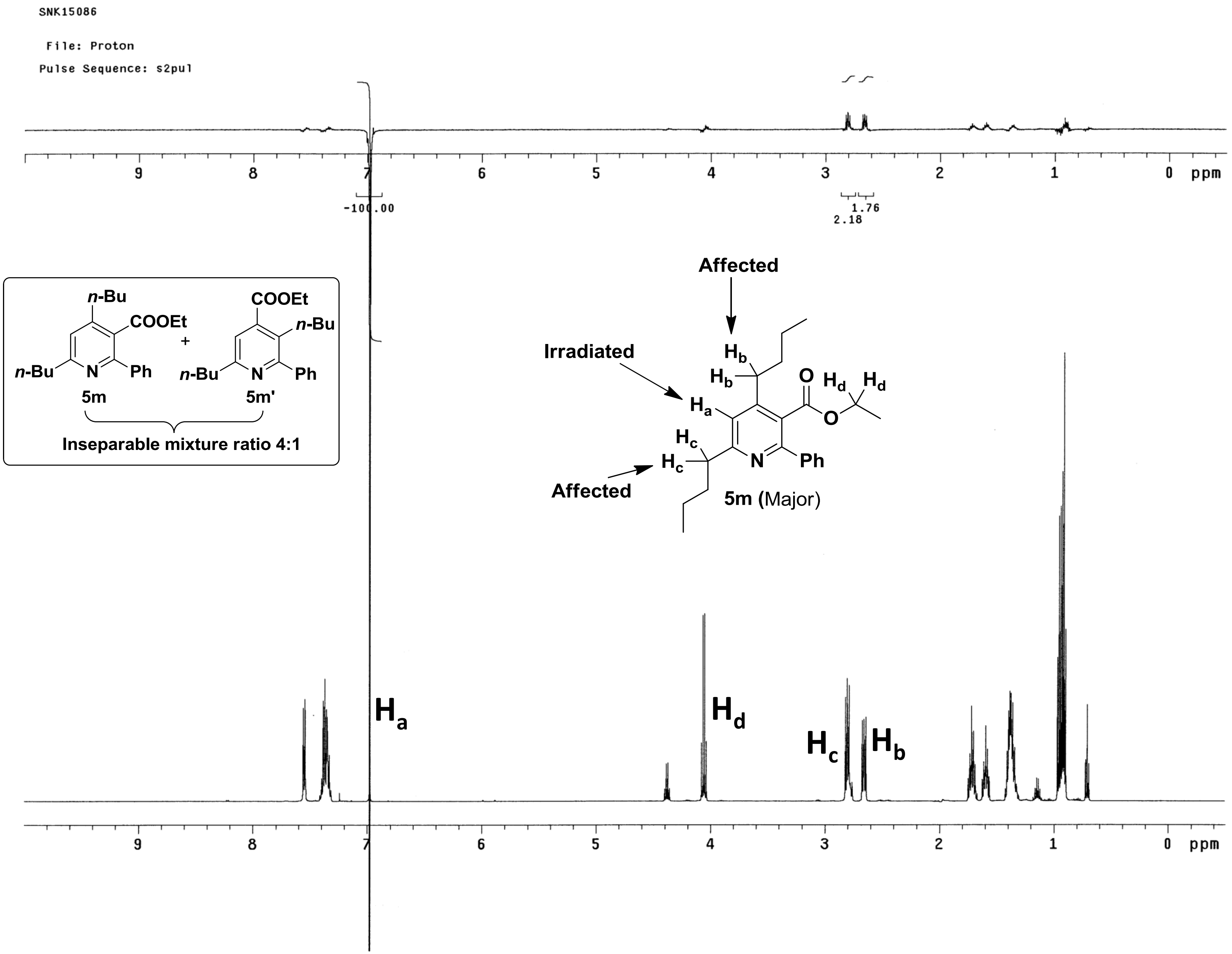
SNK15086
File: Proton
Pulse Sequence: s2pul
-100.00
2.18
1.76
9
8
7
6
5
4
3
2
1
0
ppm
n-Bu
COOEt
+
n-Bu
COOEt
n-Bu
N
Ph
n-Bu
N
Ph
5m
5m'
Inseparable mixture ratio 4:1
Affected
Irradiated
Affected
Hb
Hb
Ha
Hc
Hc
Hd
Hd
O
O
N
Ph
5m (Major)
Ha
Hd
Hc
Hb

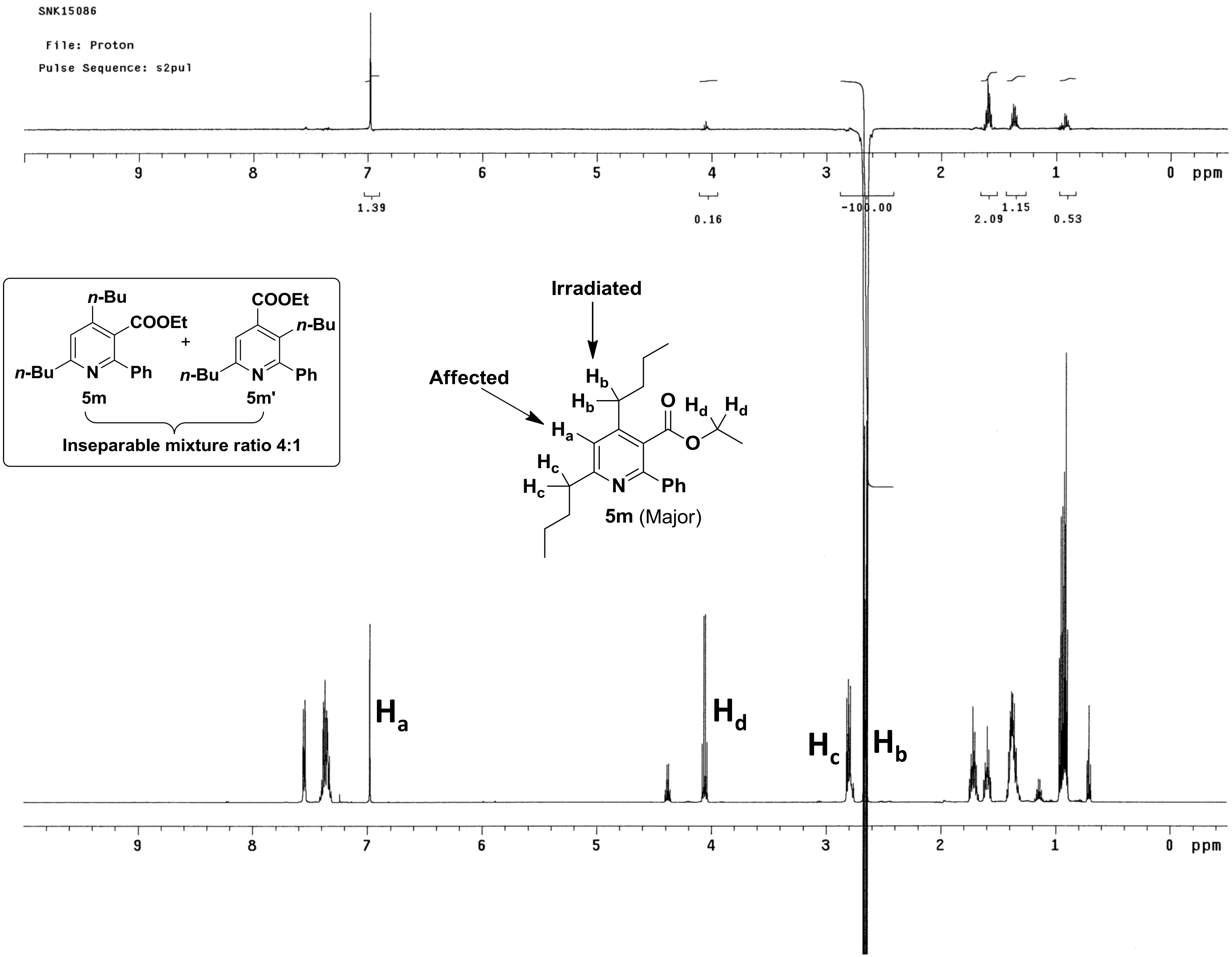

SNK15086
File: Proton
Pulse Sequence: s2pul
9
8
7
6
5
4
3
2
1
0 ppm
1.39
0.16
-100.00
2.09
1.15
0.53
n-Bu
COOEt
n-Bu
N
Ph
5m
+
COOEt
n-Bu
n-Bu
N
Ph
5m'
Inseparable mixture ratio 4:1
Irradiated
Affected
Ha
Hb
Hb
Hc
Hc
Hd
Hd
O
O
N
Ph
5m (Major)
Ha
Hd
Hc
Hb
9
8
7
6
5
4
3
2
1
0 ppm

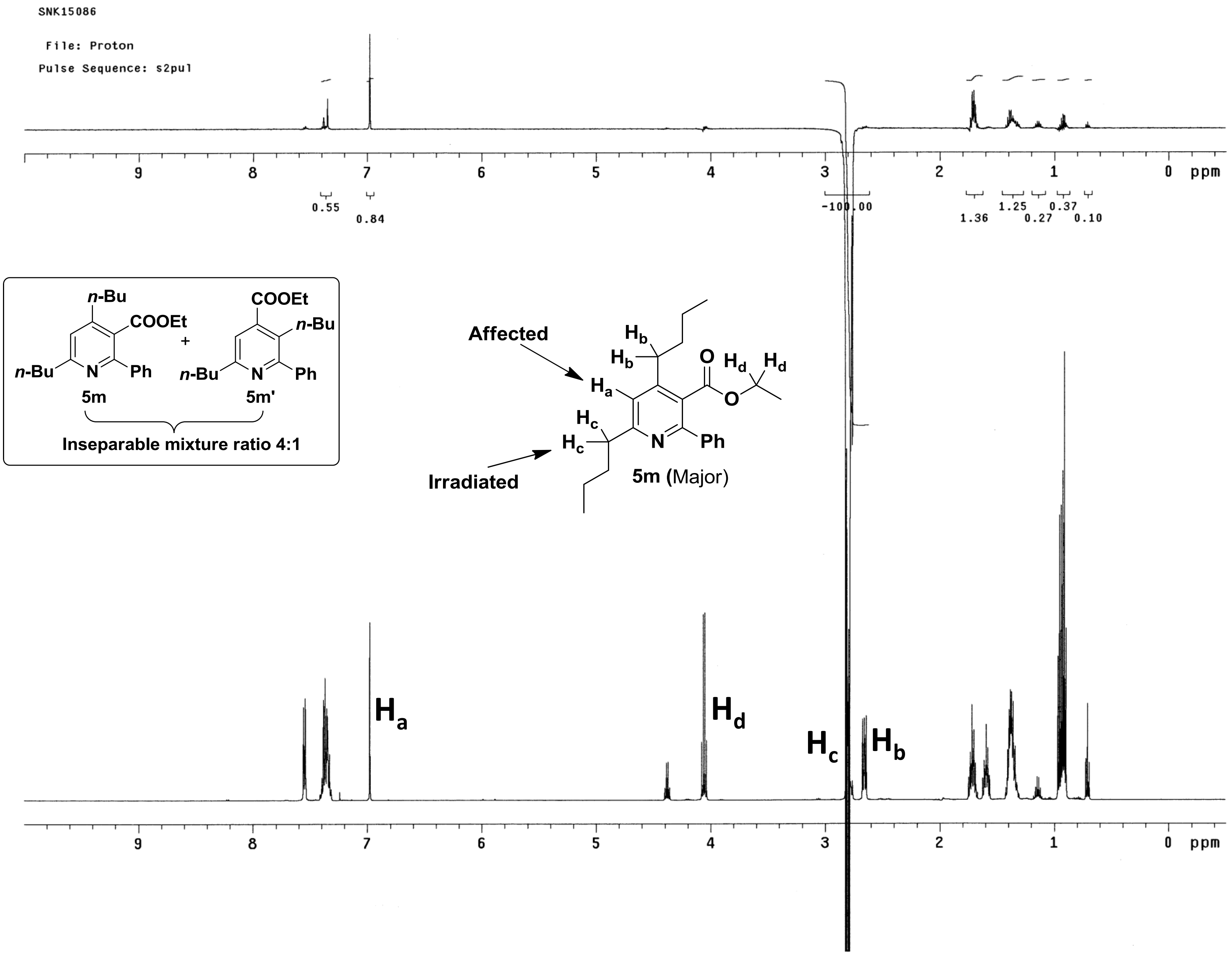
SNK15086
File: Proton
Pulse Sequence: s2pul
9
8
7
6
5
4
3
2
1
0 ppm
0.55
0.84
-100.00
1.36
1.25
0.27
0.37
0.10
n-Bu
COOEt
+
COOEt
n-Bu
n-Bu
N
Ph
n-Bu
N
Ph
5m
5m'
Inseparable mixture ratio 4:1
Affected
Irradiated
Hb
Hb
O
Hd
Hd
Ha
O
Hc
Hc
N
Ph
5m (Major)
Ha
Hd
Hc
Hb

Current Data Parameters
NAME SNK-5129
EXPNO 1
PROCNO 1

F2 - Acquisition Parameters
Date_ 20150424
Time 7.45
INSTRUM spect
PROBHD 5 mm QNP 1H/1
PULPROG zg
TD 32768
SOLVENT CDCl3
NS 16
DS 0
SWH 8389.262 Hz
FIDRES 0.256020 Hz
AQ 1.9530228 sec
RG 128
DW 59.600 usec
DE 6.50 usec
TE 296.7 K
D1 2.00000000 sec
MCREST 0.00000000 sec
MCWRK 0.01500000 sec

======== CHANNEL f1 ========
NUC1 1H
P1 10.00 usec
PL1 0.00 dB
SFO1 598.6029930 MHz

F2 - Processing parameters
SI 32768
SF 598.6000204 MHz
WDW no
SSB 0
LB 0.00 Hz
GB 0
PC 1.00

1D NMR plot parameters
CX 20.00 cm
CY 6.00 cm
F1P 3.056 ppm
F1 1829.44 Hz
F2P 0.563 ppm
F2 336.76 Hz
PPMCM 0.12468 ppm/cm
HZCM 74.63420 Hz/cm

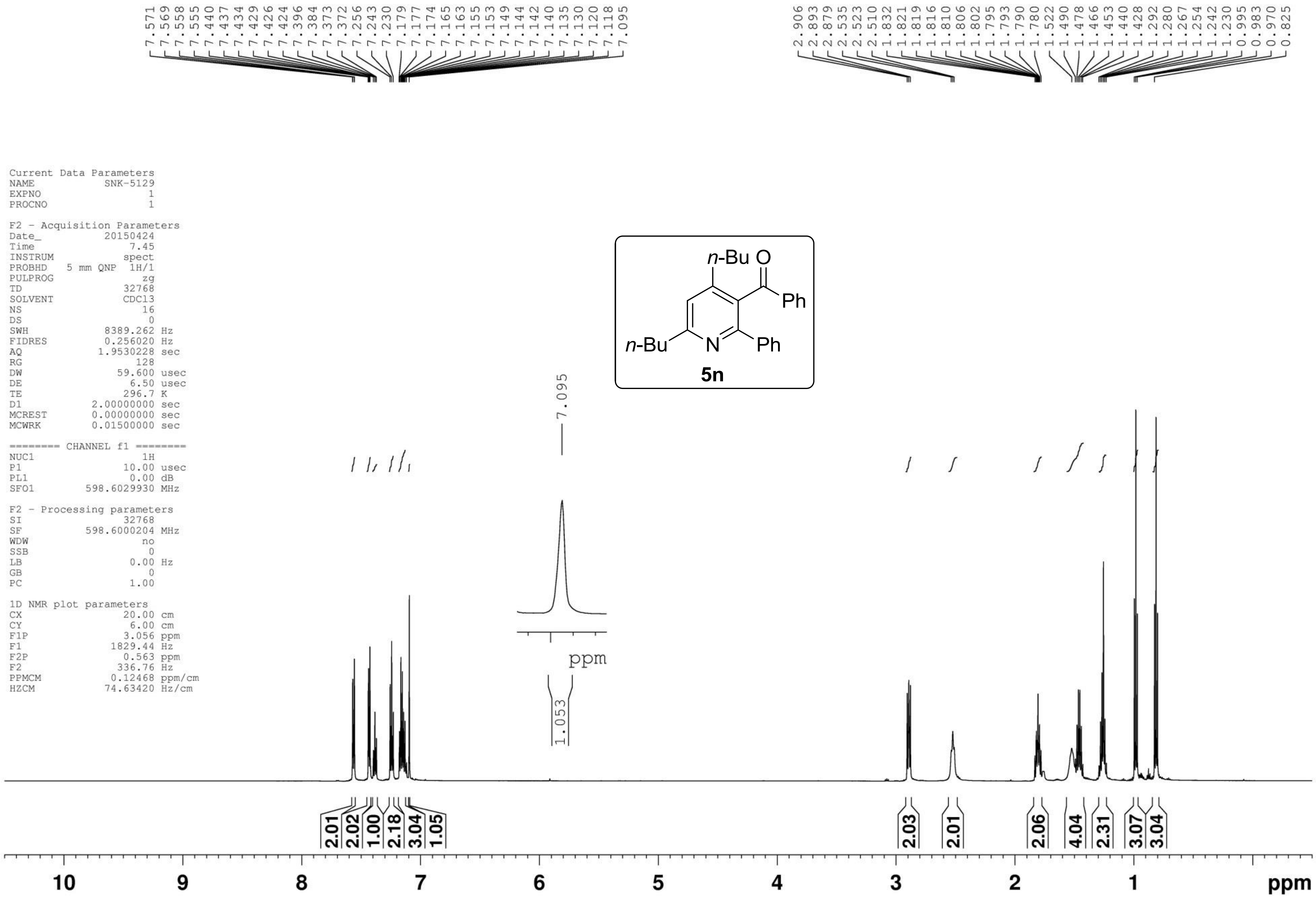

5n

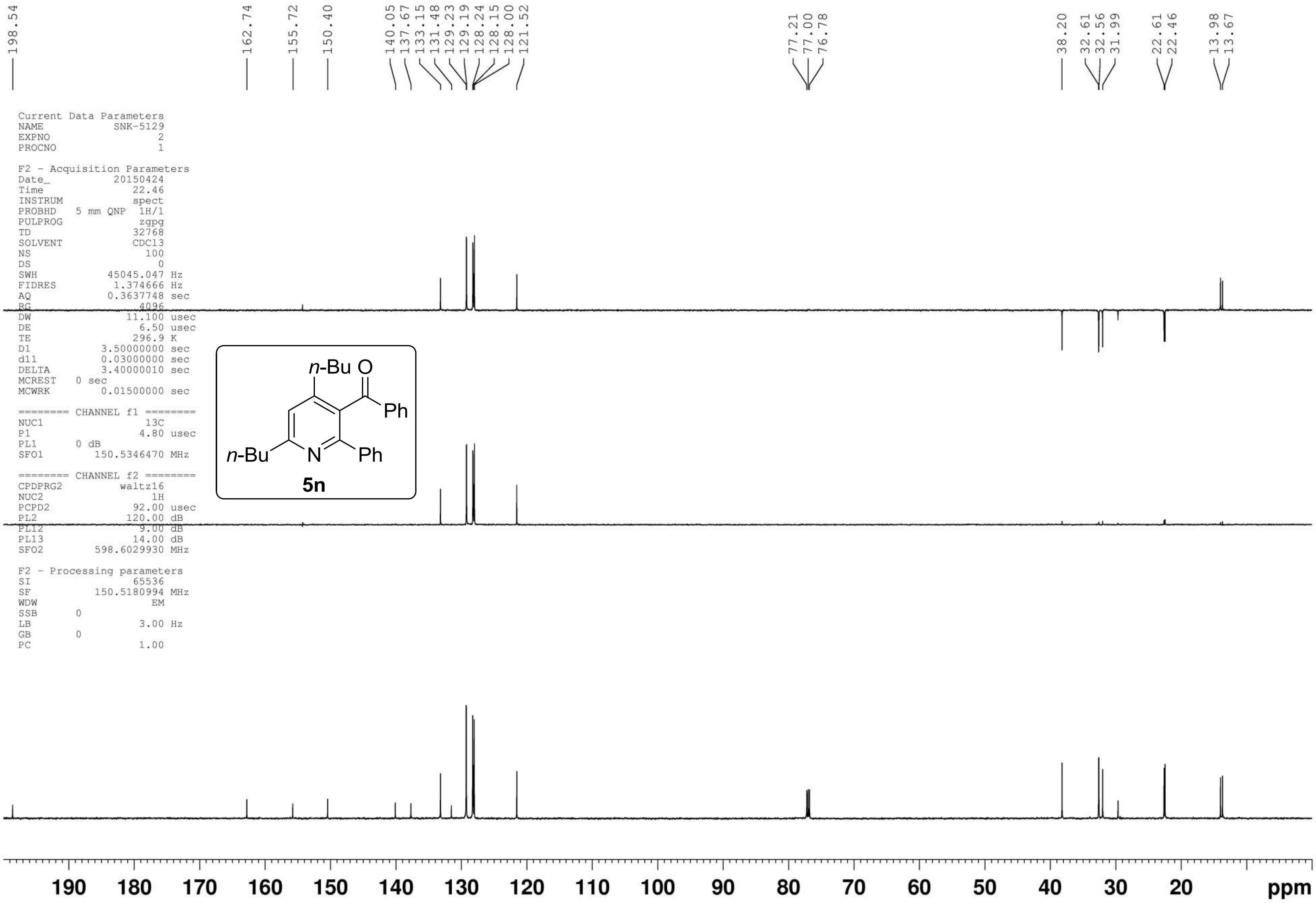
198.54
162.74
155.72
150.40
140.05
137.67
133.15
131.48
129.23
129.19
128.24
128.15
128.00
121.52
77.21
77.00
76.78
38.20
32.61
32.56
31.99
22.61
22.46
13.98
13.67
Current Data Parameters
NAME SNK-5129
EXPNO 2
PROCNO 1
F2 - Acquisition Parameters
Date_ 20150424
Time 22.46
INSTRUM spect
PROBHD 5 mm QNP 1H/1
PULPROG zgpg
TD 32768
SOLVENT CDCl3
NS 100
DS 0
SWH 45045.047 Hz
FIDRES 1.374666 Hz
AQ 0.3637748 sec
RG 4096
DW 11.100 usec
DE 6.50 usec
TE 296.9 K
D1 3.50000000 sec
d11 0.03000000 sec
DELTA 3.40000010 sec
MCREST 0 sec
MCWRK 0.01500000 sec
======== CHANNEL f1 ========
NUC1 13C
P1 4.80 usec
PL1 0 dB
SFO1 150.5346470 MHz
======== CHANNEL f2 ========
CPDPRG2 waltz16
NUC2 1H
PCPD2 92.00 usec
PL2 120.00 dB
PL12 9.00 dB
PL13 14.00 dB
SFO2 598.6029930 MHz
F2 - Processing parameters
SI 65536
SF 150.5180994 MHz
WDW EM
SSB 0
LB 3.00 Hz
GB 0
PC 1.00
n-Bu
O
Ph
n-Bu
N
Ph
5n
190
180
170
160
150
140
130
120
110
100
90
80
70
60
50
40
30
20
ppm

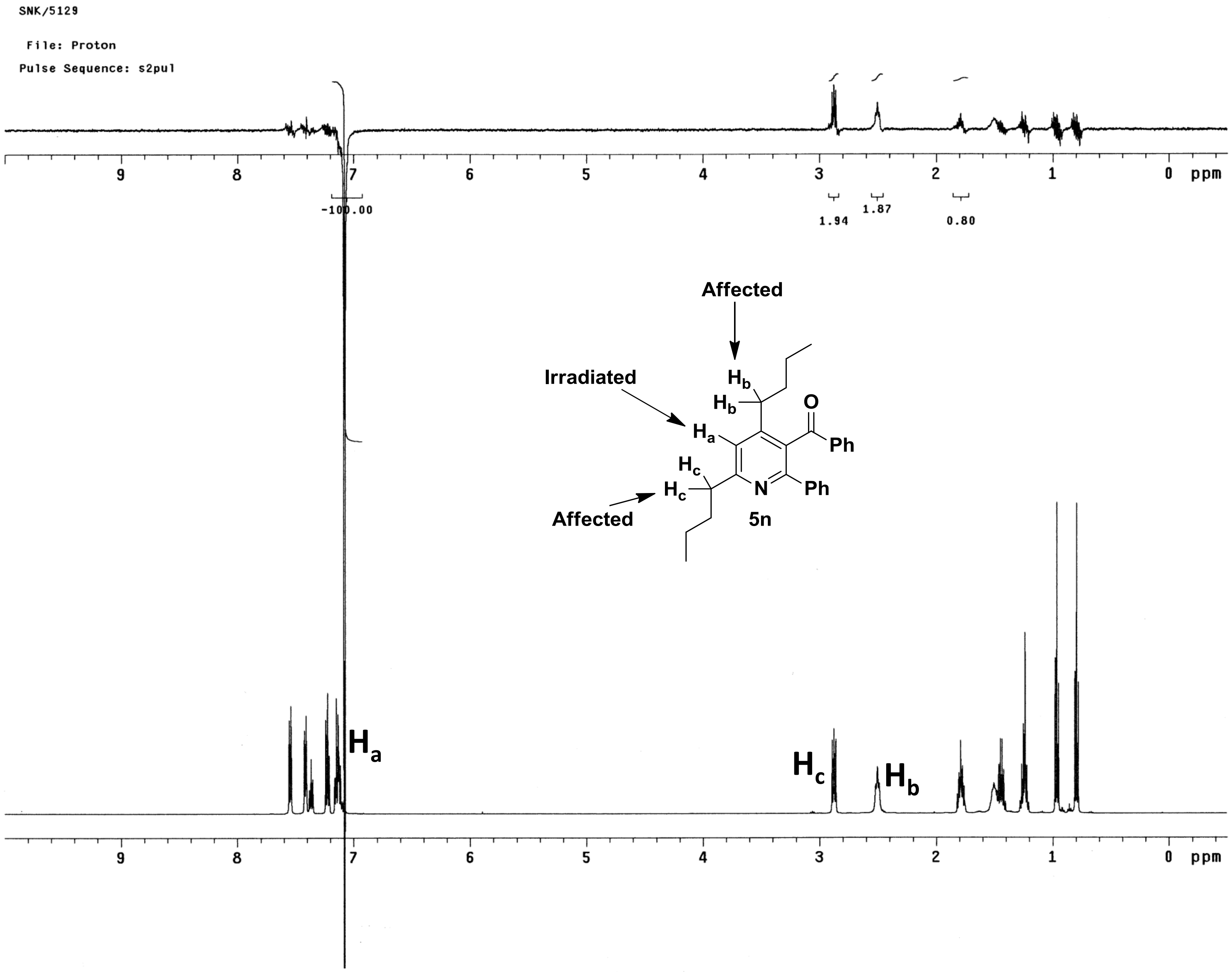
SNK/5129
File: Proton
Pulse Sequence: s2pul
9 8 7 6 5 4 3 2 1 0 ppm
-100.00
1.94
1.87
0.80
Affected
Irradiated
Affected
Hb
Hb
Ha
Hc
Hc
O
Ph
N
Ph
5n
Ha
Hc
Hb
9 8 7 6 5 4 3 2 1 0 ppm

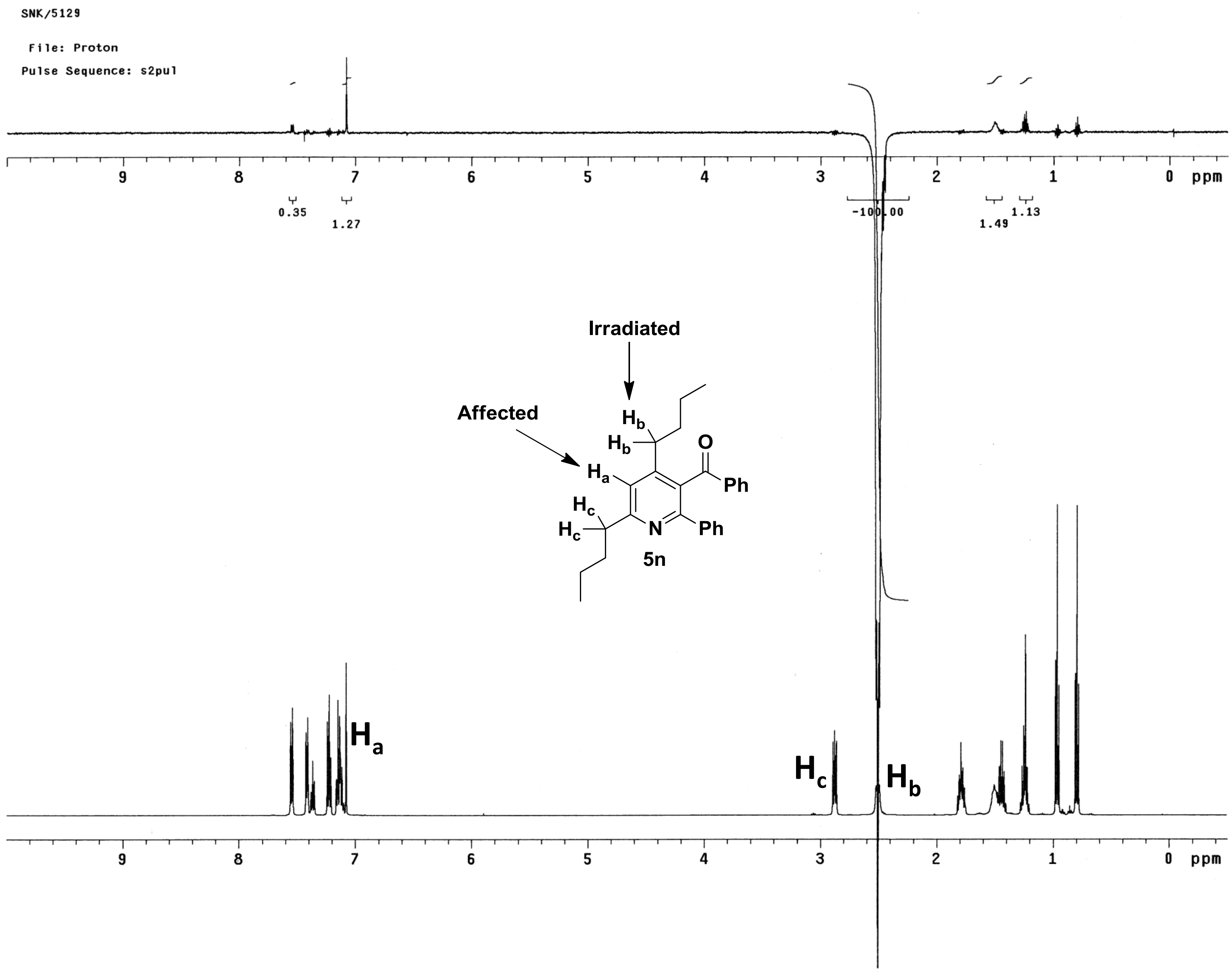
SNK/5129
File: Proton
Pulse Sequence: s2pul
ppm
0.35
1.27
-100.00
1.49
1.13
Irradiated
Affected
Hb
Ha
Hc
O
Ph
N
Ph
5n
Ha
Hc
Hb

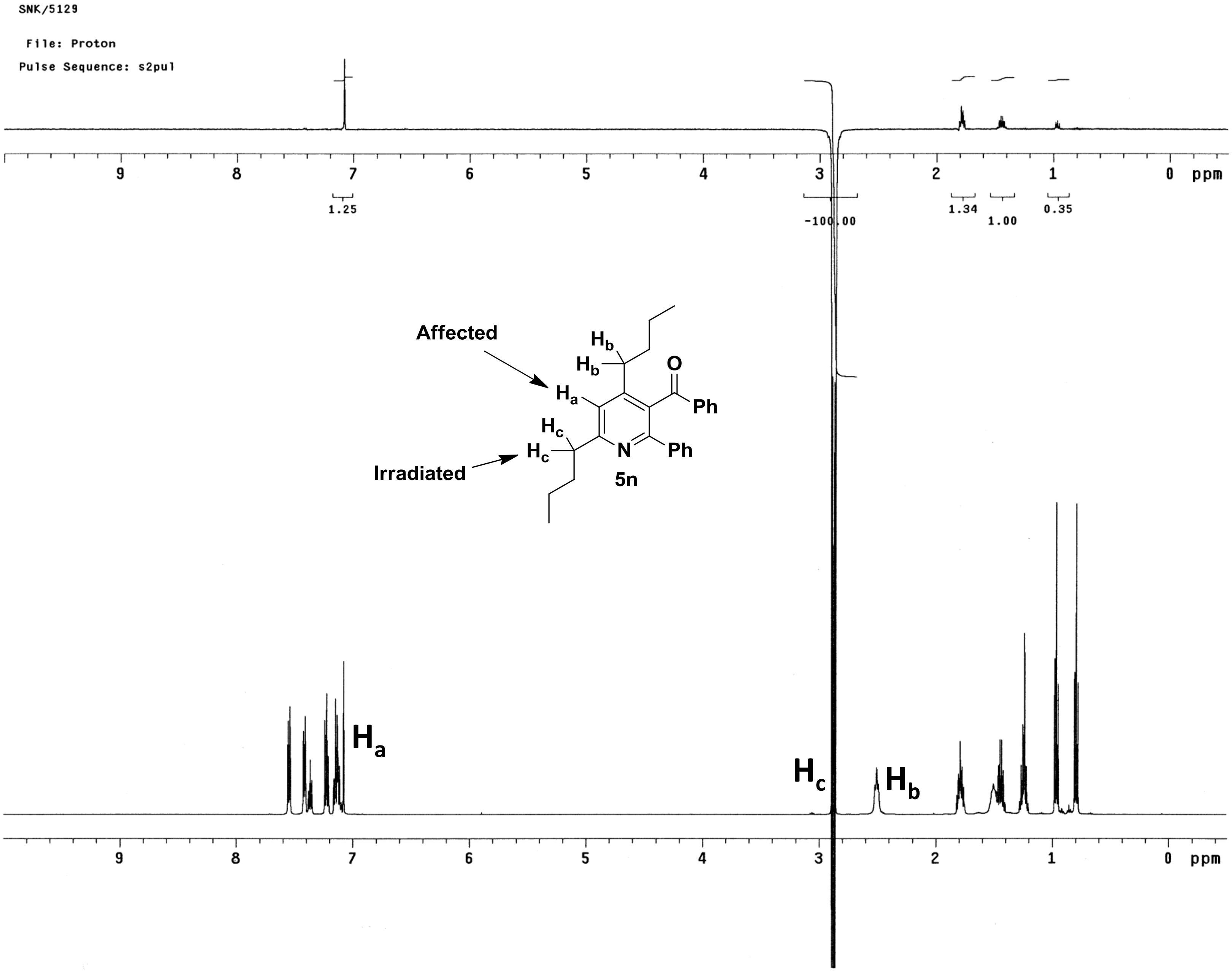
SNK/5129
File: Proton
Pulse Sequence: s2pul
9
8
7
6
5
4
3
2
1
0 ppm
1.25
-100.00
1.34
1.00
0.35
Affected
Irradiated
Ha
Hb
Hb
Hc
Hc
O
Ph
N
Ph
5n
Ha
Hc
Hb
9
8
7
6
5
4
3
2
1
0 ppm

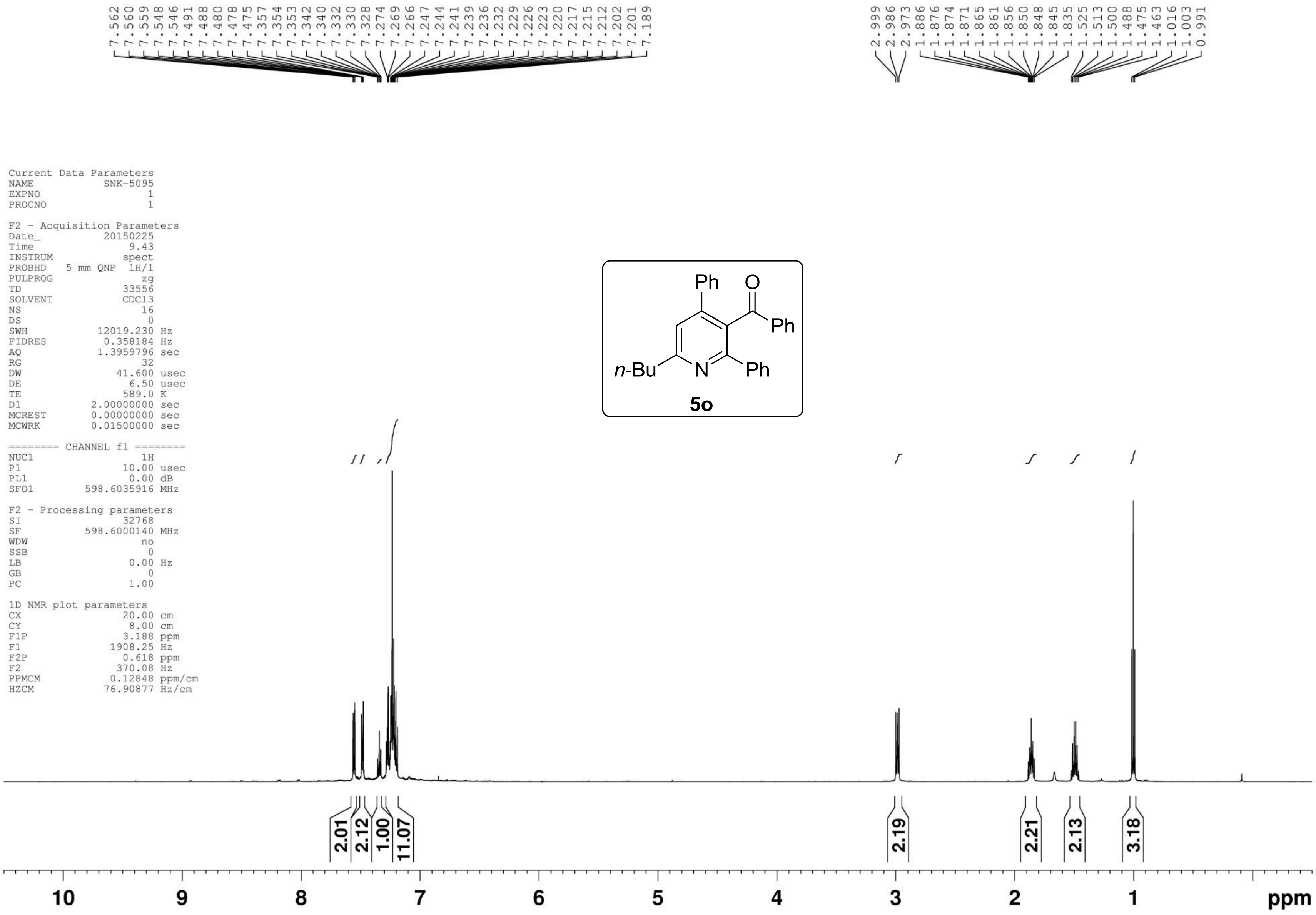
7.562
7.560
7.559
7.548
7.546
7.491
7.488
7.480
7.478
7.475
7.357
7.354
7.353
7.342
7.340
7.332
7.330
7.328
7.274
7.269
7.266
7.247
7.244
7.241
7.239
7.236
7.232
7.229
7.226
7.223
7.220
7.217
7.215
7.212
7.202
7.201
7.189
2.999
2.986
2.973
1.886
1.876
1.874
1.871
1.865
1.861
1.856
1.850
1.848
1.845
1.835
1.525
1.513
1.500
1.488
1.475
1.463
1.016
1.003
0.991
Current Data Parameters
NAME SNK-5095
EXPNO 1
PROCNO 1
F2 - Acquisition Parameters
Date_ 20150225
Time 9.43
INSTRUM spect
PROBHD 5 mm QNP 1H/1
PULPROG zg
TD 33556
SOLVENT CDCl3
NS 16
DS 0
SWH 12019.230 Hz
FIDRES 0.358184 Hz
AQ 1.3959796 sec
RG 32
DW 41.600 usec
DE 6.50 usec
TE 589.0 K
D1 2.00000000 sec
MCREST 0.00000000 sec
MCWRK 0.01500000 sec
======== CHANNEL f1 ========
NUC1 1H
P1 10.00 usec
PL1 0.00 dB
SFO1 598.6035916 MHz
F2 - Processing parameters
SI 32768
SF 598.6000140 MHz
WDW no
SSB 0
LB 0.00 Hz
GB 0
PC 1.00
1D NMR plot parameters
CX 20.00 cm
CY 8.00 cm
F1P 3.188 ppm
F1 1908.25 Hz
F2P 0.618 ppm
F2 370.08 Hz
PPMCM 0.12848 ppm/cm
HZCM 76.90877 Hz/cm
Ph
O
Ph
n-Bu
N
Ph
5o
2.01
2.12
1.00
11.07
2.19
2.21
2.13
3.18
10
9
8
7
6
5
4
3
2
1
ppm

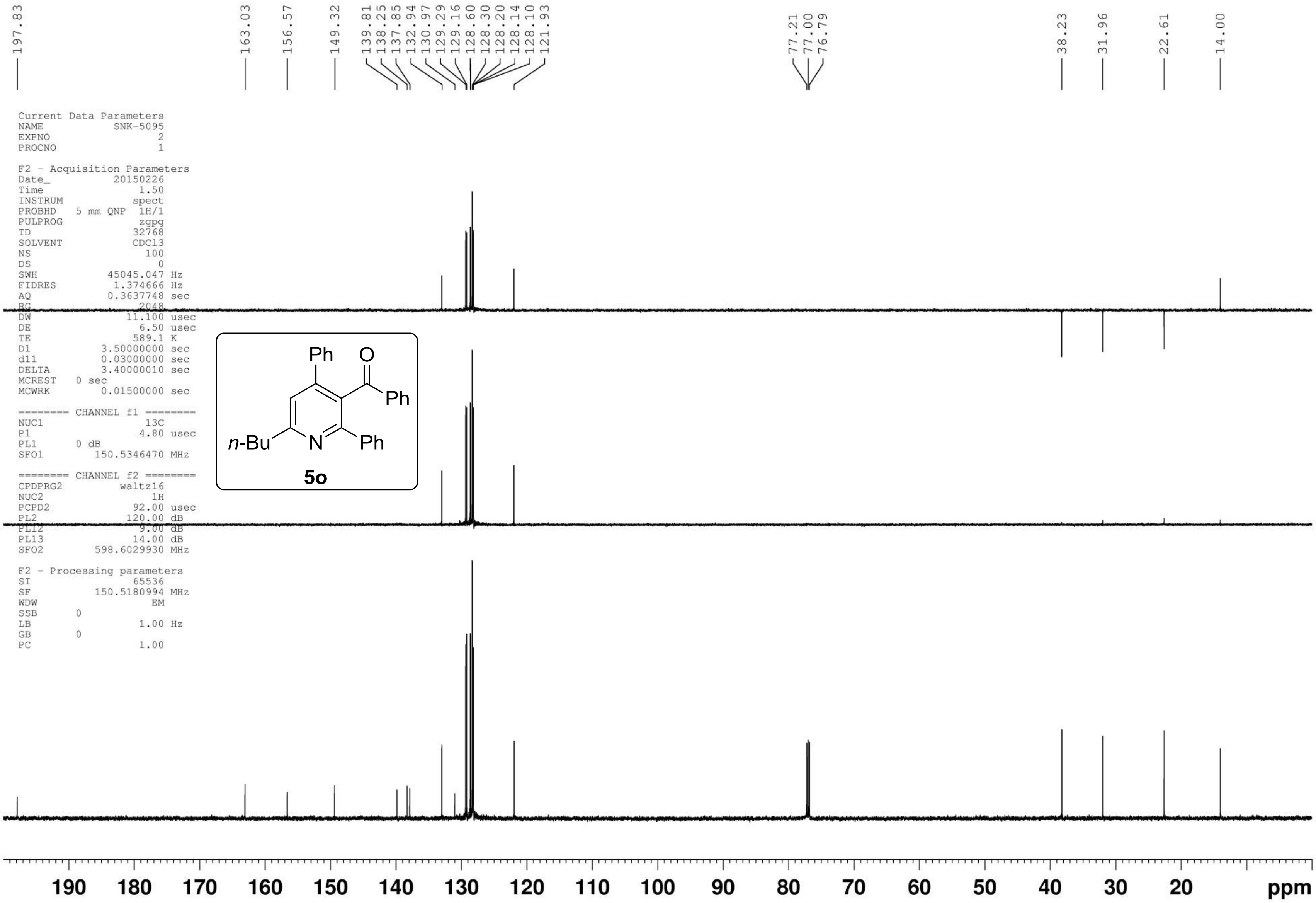

197.83
163.03
156.57
149.32
139.81
138.25
137.85
132.94
130.97
129.29
129.16
128.60
128.30
128.20
128.14
128.10
121.93
77.21
77.00
76.79
38.23
31.96
22.61
14.00
Current Data Parameters
NAME SNK-5095
EXPNO 2
PROCNO 1
F2 - Acquisition Parameters
Date_ 20150226
Time 1.50
INSTRUM spect
PROBHD 5 mm QNP 1H/1
PULPROG zgpg
TD 32768
SOLVENT CDCl3
NS 100
DS 0
SWH 45045.047 Hz
FIDRES 1.374666 Hz
AQ 0.3637748 sec
RG 2048
DW 11.100 usec
DE 6.50 usec
TE 589.1 K
D1 3.50000000 sec
d11 0.03000000 sec
DELTA 3.40000010 sec
MCREST 0 sec
MCWRK 0.01500000 sec
======== CHANNEL f1 ========
NUC1 13C
P1 4.80 usec
PL1 0 dB
SFO1 150.5346470 MHz
======== CHANNEL f2 ========
CPDPRG2 waltz16
NUC2 1H
PCPD2 92.00 usec
PL2 120.00 dB
PL12 9.00 dB
PL13 14.00 dB
SFO2 598.6029930 MHz
F2 - Processing parameters
SI 65536
SF 150.5180994 MHz
WDW EM
SSB 0
LB 1.00 Hz
GB 0
PC 1.00
Ph
O
Ph
n-Bu
N
Ph
5o
190 180 170 160 150 140 130 120 110 100 90 80 70 60 50 40 30 20 ppm

Heteronuclear single quantum coherence spectroscopy (HSQC) Full Spectrum (compound 5o)

Current Data Parameters

NAME	SNK-5095
EXPNO	12
PROCNO	1

F2 - Acquisition Parameters

Date_	20150226
Time	8.09
INSTRUM	spect
PROBHD	5 mm QNP 1H/1
PULPROG	hsqcetgpsi
TD	2048
SOLVENT	CDCl3
NS	4
DS	8
SWH	6009.615 Hz
FIDRES	2.934382 Hz
AQ	0.1704436 sec
RG	16384
DW	83.200 usec
DE	6.50 usec
TE	296.2 K
CNST2	145.0000000
d0	0.00000300 sec
D1	1.00000000 sec
d4	0.00172414 sec
d11	0.03000000 sec
d13	0.00000400 sec
D16	0.00050000 sec
D24	0.00089000 sec
DELTA	0.00157060 sec
DELTA1	0.00150800 sec
IN0	0.00001384 sec
MCREST	0.00000000 sec
MCWRK	0.16666700 sec
ST1CNT	64

======== CHANNEL f1 ========

NUC1	1H
P1	7.30 usec
p2	14.60 usec
P28	1000.00 usec
PL1	3.00 dB
SFO1	598.6023944 MHz

======== CHANNEL f2 ========

CPDPRG2	garp
NUC2	13C
P3	10.00 usec
p4	20.00 usec
PCPD2	70.00 usec
PL2	0.00 dB
PL12	20.00 dB
SFO2	150.5301314 MHz

====== GRADIENT CHANNEL =====

GPNAM1	SINE.100
GPNAM2	SINE.100
GPX1	0.00 %
GPX2	0.00 %
GPY1	0.00 %
GPY2	0.00 %
GPZ1	80.00 %
GPZ2	20.10 %
P16	1000.00 usec

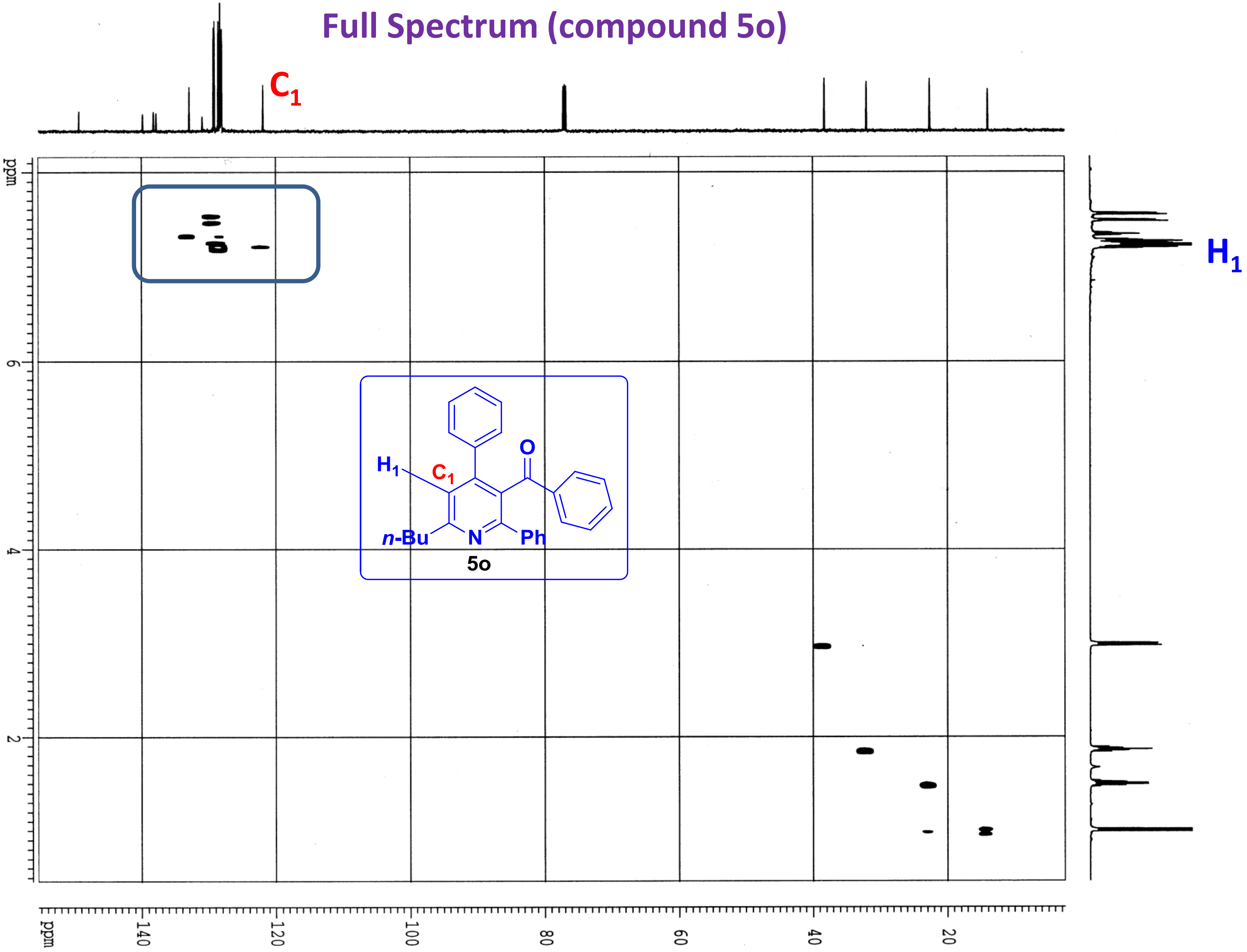

H_1 δ 7.20

Heteronuclear single quantum coherence spectroscopy (HSQC) Expansion (compound 5o)

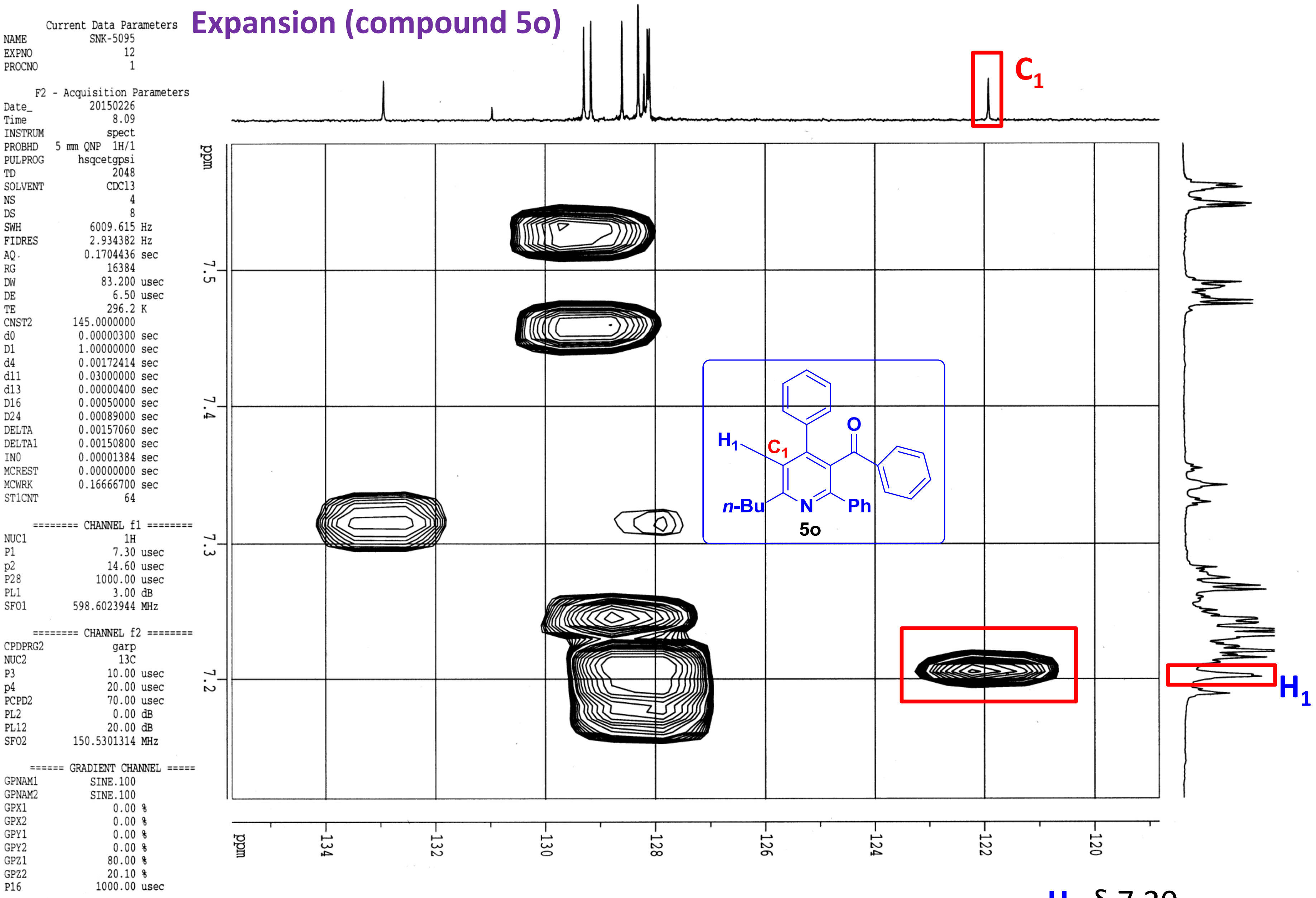

H_1 δ 7.20

Heteronuclear Multiple Bond Correlation (HMBC) Full Spectrum (compound 5o)

```
     Current Data Parameters
NAME            SNK-5095
EXPNO                 13
PROCNO                 1

     F2 - Acquisition Parameters
Date_           20150226
Time                8.30
INSTRUM            spect
PROBHD   5 mm QNP  1H/1
PULPROG   hmbcgplpndqf
TD                  2048
SOLVENT             DMSO
NS                     4
DS                     0
SWH             6009.615 Hz
FIDRES          2.934382 Hz
AQ             0.1704436 sec
RG                 32768
DW                83.200 usec
DE                  6.00 usec
TE                 297.1 K
CNST2        145.0000000
CNST13         7.0000000
d0            0.00000300 sec
D1            1.00000000 sec
d2            0.00344828 sec
d6            0.07142857 sec
D16           0.00050000 sec
IN0           0.00001384 sec
MCREST        0.00000000 sec
MCWRK         1.00000000 sec

======== CHANNEL f1 ========
NUC1                  1H
P1                  8.00 usec
p2                 16.00 usec
PL1                 3.00 dB
SFO1         598.6023944 MHz

======== CHANNEL f2 ========
NUC2                 13C
P3                 10.50 usec
PL2                 0.00 dB
SFO2         150.5316366 MHz

====== GRADIENT CHANNEL =====
GPNAM1          SINE.100
GPNAM2          SINE.100
GPNAM3          SINE.100
GPX1                0.00 %
GPX2                0.00 %
GPX3                0.00 %
GPY1                0.00 %
GPY2                0.00 %
GPY3                0.00 %
GPZ1               50.00 %
GPZ2               30.00 %
GPZ3               40.10 %
P16              1000.00 usec

     F1 - Acquisition parameters
ND0                    2
TD                   256
SFO1            150.5316 MHz
FIDRES        141.147247 Hz
SW               240.041 ppm
FnMODE                QF

     F2 - Processing parameters
SI                  4096
SF           598.6000159 MHz
WDW                QSINE
SSB                    2
LB                  0.00 Hz
GB                     0
PC                  1.00

     F1 - Processing parameters
SI                   512
MC2                   QF
SF           150.5180375 MHz
```

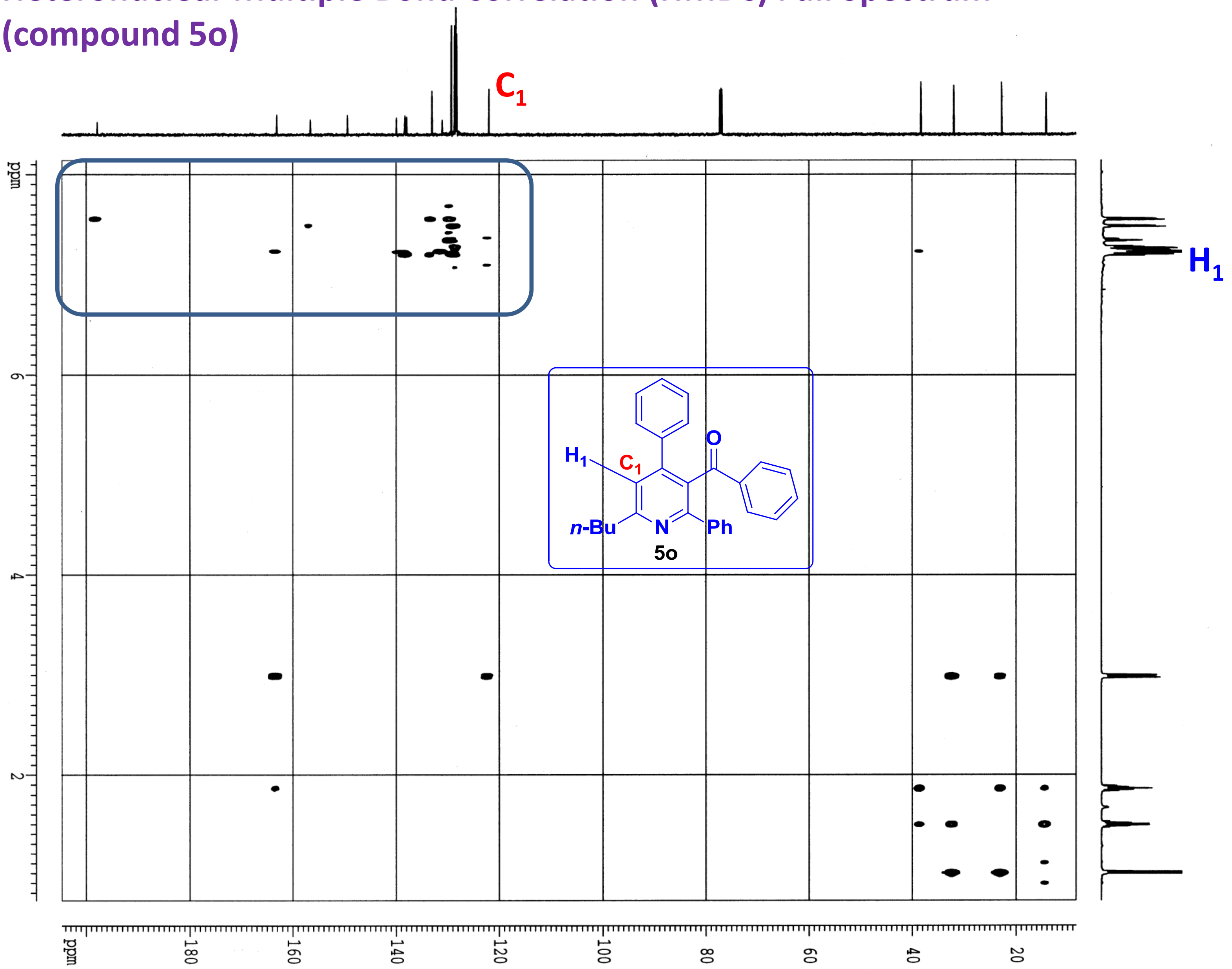

H_1 δ 7.20

Heteronuclear Multiple Bond Correlation (HMBC) Expansion (compound 5o)

Current Data Parameters
NAME SNK-5095
EXPNO 13
PROCNO 1

F2 - Acquisition Parameters
Date_ 20150226
Time 8.30
INSTRUM spect
PROBHD 5 mm QNP 1H/1
PULPROG hmbcgplpndqf
TD 2048
SOLVENT DMSO
NS 4
DS 0
SWH 6009.615 Hz
FIDRES 2.934382 Hz
AQ 0.1704436 sec
RG 32768
DW 83.200 usec
DE 6.00 usec
TE 297.1 K
CNST2 145.0000000
CNST13 7.0000000
d0 0.00000300 sec
D1 1.00000000 sec
d2 0.00344828 sec
d6 0.07142857 sec
D16 0.00050000 sec
IN0 0.00001384 sec
MCREST 0.00000000 sec
MCWRK 1.00000000 sec

======== CHANNEL f1 ========
NUC1 1H
P1 8.00 usec
p2 16.00 usec
PL1 3.00 dB
SFO1 598.6023944 MHz

======== CHANNEL f2 ========
NUC2 13C
P3 10.50 usec
PL2 0.00 dB
SFO2 150.5316366 MHz

====== GRADIENT CHANNEL =====
GPNAM1 SINE.100
GPNAM2 SINE.100
GPNAM3 SINE.100
GPX1 0.00 %
GPX2 0.00 %
GPX3 0.00 %
GPY1 0.00 %
GPY2 0.00 %
GPY3 0.00 %
GPZ1 50.00 %
GPZ2 30.00 %
GPZ3 40.10 %
P16 1000.00 usec

F1 - Acquisition parameters
ND0 2
TD 256
SFO1 150.5316 MHz
FIDRES 141.147247 Hz
SW 240.041 ppm
FnMODE QF

F2 - Processing parameters
SI 4096
SF 598.6000159 MHz
WDW QSINE
SSB 2
LB 0.00 Hz
GB 0
PC 1.00

F1 - Processing parameters
SI 512
MC2 QF
SF 150.5180375 MHz

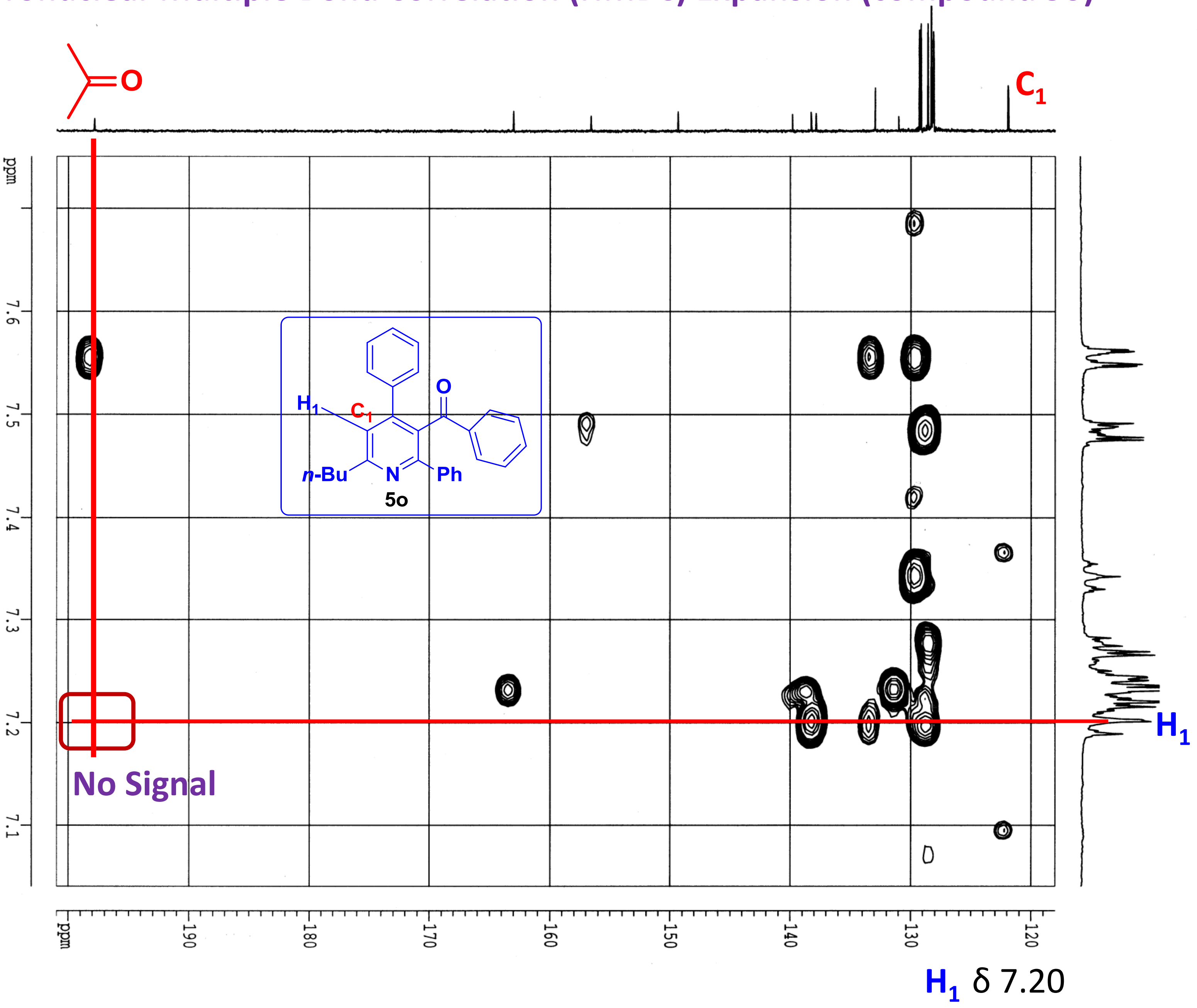

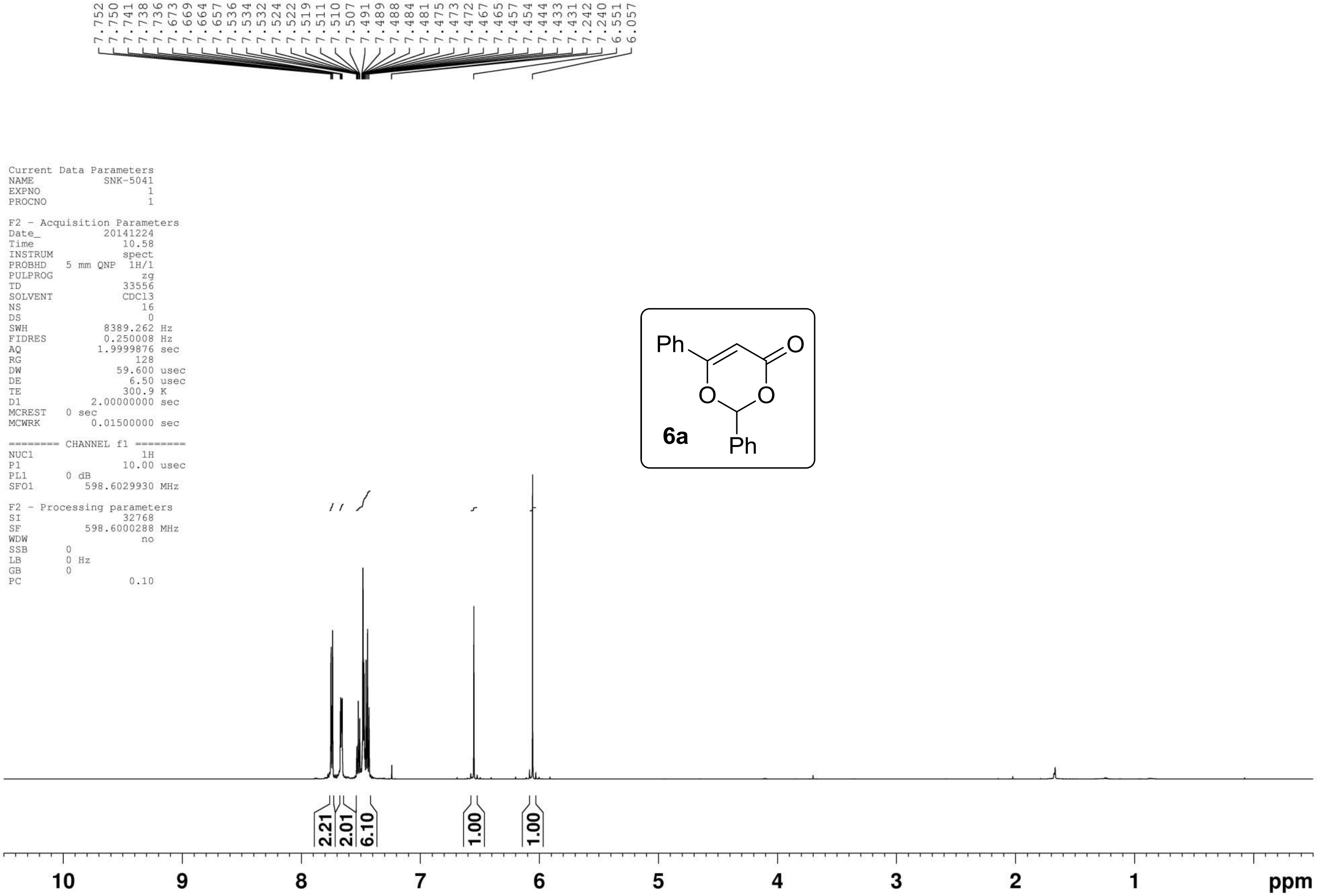
7.752 7.750 7.741 7.738 7.736 7.673 7.669 7.664 7.657 7.536 7.534 7.532 7.524 7.522 7.519 7.511 7.510 7.507 7.491 7.489 7.488 7.484 7.481 7.475 7.473 7.472 7.467 7.465 7.457 7.454 7.444 7.433 7.431 7.242 7.240 6.551 6.057
Current Data Parameters
NAME SNK-5041
EXPNO 1
PROCNO 1
F2 - Acquisition Parameters
Date_ 20141224
Time 10.58
INSTRUM spect
PROBHD 5 mm QNP 1H/1
PULPROG zg
TD 33556
SOLVENT CDCl3
NS 16
DS 0
SWH 8389.262 Hz
FIDRES 0.250008 Hz
AQ 1.9999876 sec
RG 128
DW 59.600 usec
DE 6.50 usec
TE 300.9 K
D1 2.00000000 sec
MCREST 0 sec
MCWRK 0.01500000 sec
======== CHANNEL f1 ========
NUC1 1H
P1 10.00 usec
PL1 0 dB
SFO1 598.6029930 MHz
F2 - Processing parameters
SI 32768
SF 598.6000288 MHz
WDW no
SSB 0
LB 0 Hz
GB 0
PC 0.10
Ph
O
O
O
Ph
6a
2.21
2.01
6.10
1.00
1.00
10
9
8
7
6
5
4
3
2
1
ppm

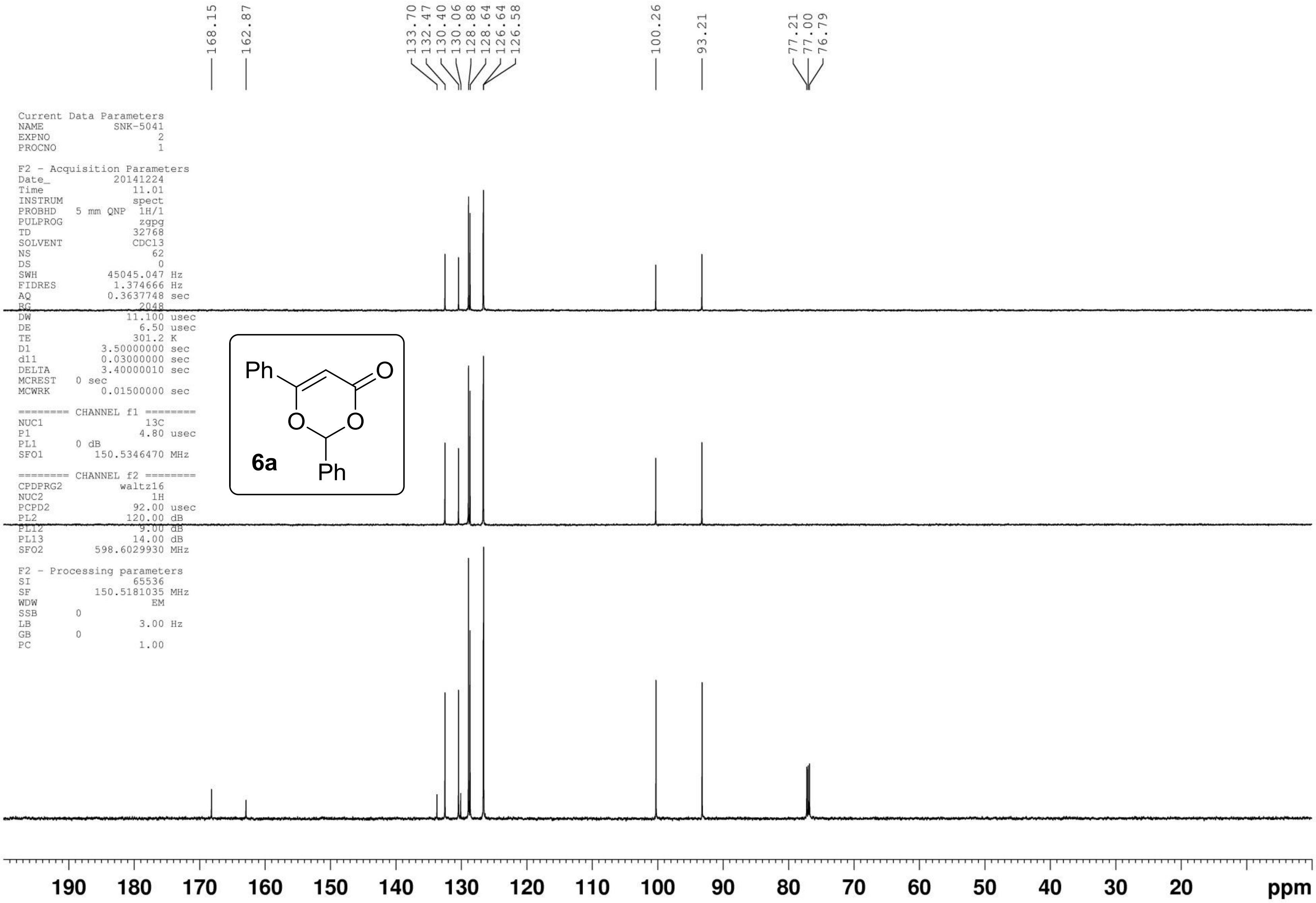
168.15
162.87
133.70
132.47
130.40
130.06
128.88
128.64
126.64
126.58
100.26
93.21
77.21
77.00
76.79
Current Data Parameters
NAME SNK-5041
EXPNO 2
PROCNO 1
F2 - Acquisition Parameters
Date_ 20141224
Time 11.01
INSTRUM spect
PROBHD 5 mm QNP 1H/1
PULPROG zgpg
TD 32768
SOLVENT CDCl3
NS 62
DS 0
SWH 45045.047 Hz
FIDRES 1.374666 Hz
AQ 0.3637748 sec
RG 2048
DW 11.100 usec
DE 6.50 usec
TE 301.2 K
D1 3.50000000 sec
d11 0.03000000 sec
DELTA 3.40000010 sec
MCREST 0 sec
MCWRK 0.01500000 sec
======== CHANNEL f1 ========
NUC1 13C
P1 4.80 usec
PL1 0 dB
SFO1 150.5346470 MHz
======== CHANNEL f2 ========
CPDPRG2 waltz16
NUC2 1H
PCPD2 92.00 usec
PL2 120.00 dB
PL12 9.00 dB
PL13 14.00 dB
SFO2 598.6029930 MHz
F2 - Processing parameters
SI 65536
SF 150.5181035 MHz
WDW EM
SSB 0
LB 3.00 Hz
GB 0
PC 1.00
Ph
O
O
O
Ph
6a
190 180 170 160 150 140 130 120 110 100 90 80 70 60 50 40 30 20 ppm

```
Current Data Parameters
NAME             SNK-5059
EXPNO                   1
PROCNO                  1

F2 - Acquisition Parameters
Date_            20150109
Time                 5.00
INSTRUM             spect
PROBHD   5 mm QNP  1H/1
PULPROG                zg
TD                  33556
SOLVENT             CDCl3
NS                     16
DS                      0
SWH             12019.230 Hz
FIDRES           0.358184 Hz
AQ              1.3959796 sec
RG                     64
DW                 41.600 usec
DE                   6.50 usec
TE                  293.3 K
D1             2.00000000 sec
MCREST   0 sec
MCWRK          0.01500000 sec

======== CHANNEL f1 ========
NUC1                   1H
P1                  10.00 usec
PL1      0 dB
SFO1          598.6035916 MHz

F2 - Processing parameters
SI                  32768
SF            598.6000298 MHz
WDW                    no
SSB      0
LB       0 Hz
GB       0
PC                   1.00
```

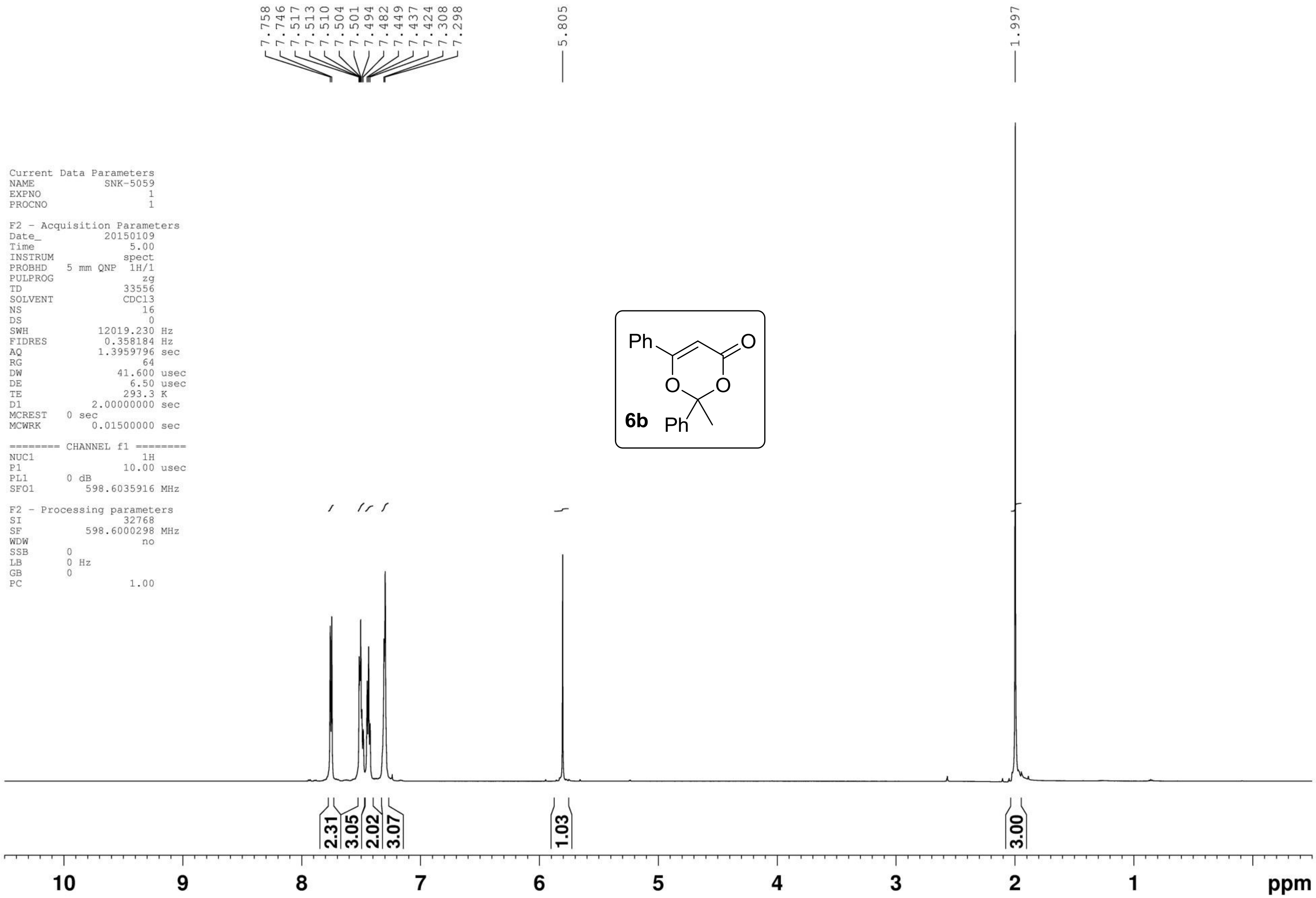

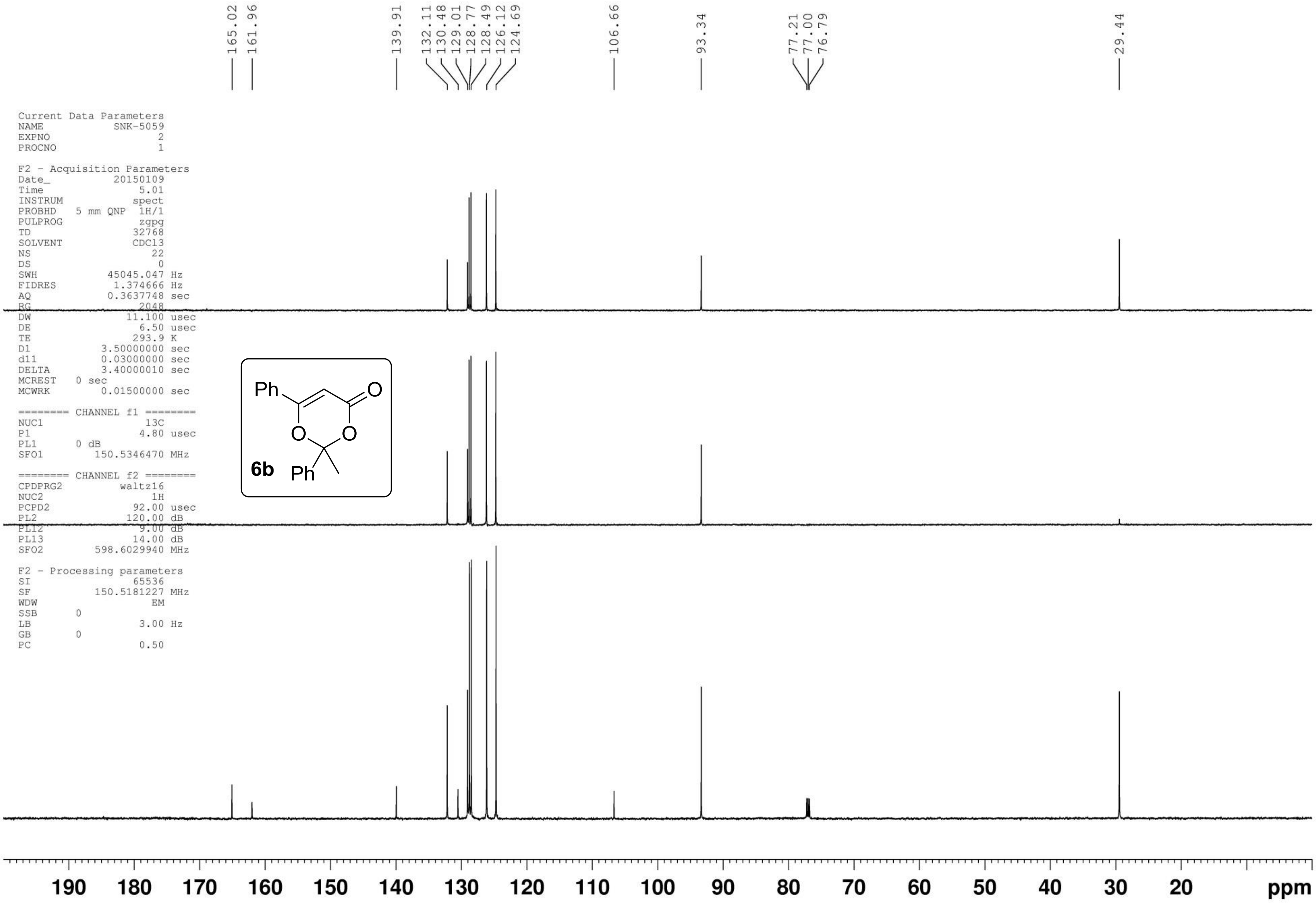
165.02
161.96
139.91
132.11
130.48
129.01
128.77
128.49
126.12
124.69
106.66
93.34
77.21
77.00
76.79
29.44
Current Data Parameters
NAME SNK-5059
EXPNO 2
PROCNO 1
F2 - Acquisition Parameters
Date_ 20150109
Time 5.01
INSTRUM spect
PROBHD 5 mm QNP 1H/1
PULPROG zgpg
TD 32768
SOLVENT CDC13
NS 22
DS 0
SWH 45045.047 Hz
FIDRES 1.374666 Hz
AQ 0.3637748 sec
RG 2048
DW 11.100 usec
DE 6.50 usec
TE 293.9 K
D1 3.50000000 sec
d11 0.03000000 sec
DELTA 3.40000010 sec
MCREST 0 sec
MCWRK 0.01500000 sec
======== CHANNEL f1 ========
NUC1 13C
P1 4.80 usec
PL1 0 dB
SFO1 150.5346470 MHz
======== CHANNEL f2 ========
CPDPRG2 waltz16
NUC2 1H
PCPD2 92.00 usec
PL2 120.00 dB
PL12 9.00 dB
PL13 14.00 dB
SFO2 598.6029940 MHz
F2 - Processing parameters
SI 65536
SF 150.5181227 MHz
WDW EM
SSB 0
LB 3.00 Hz
GB 0
PC 0.50
Ph
O
O
O
Ph
6b
190
180
170
160
150
140
130
120
110
100
90
80
70
60
50
40
30
20
ppm

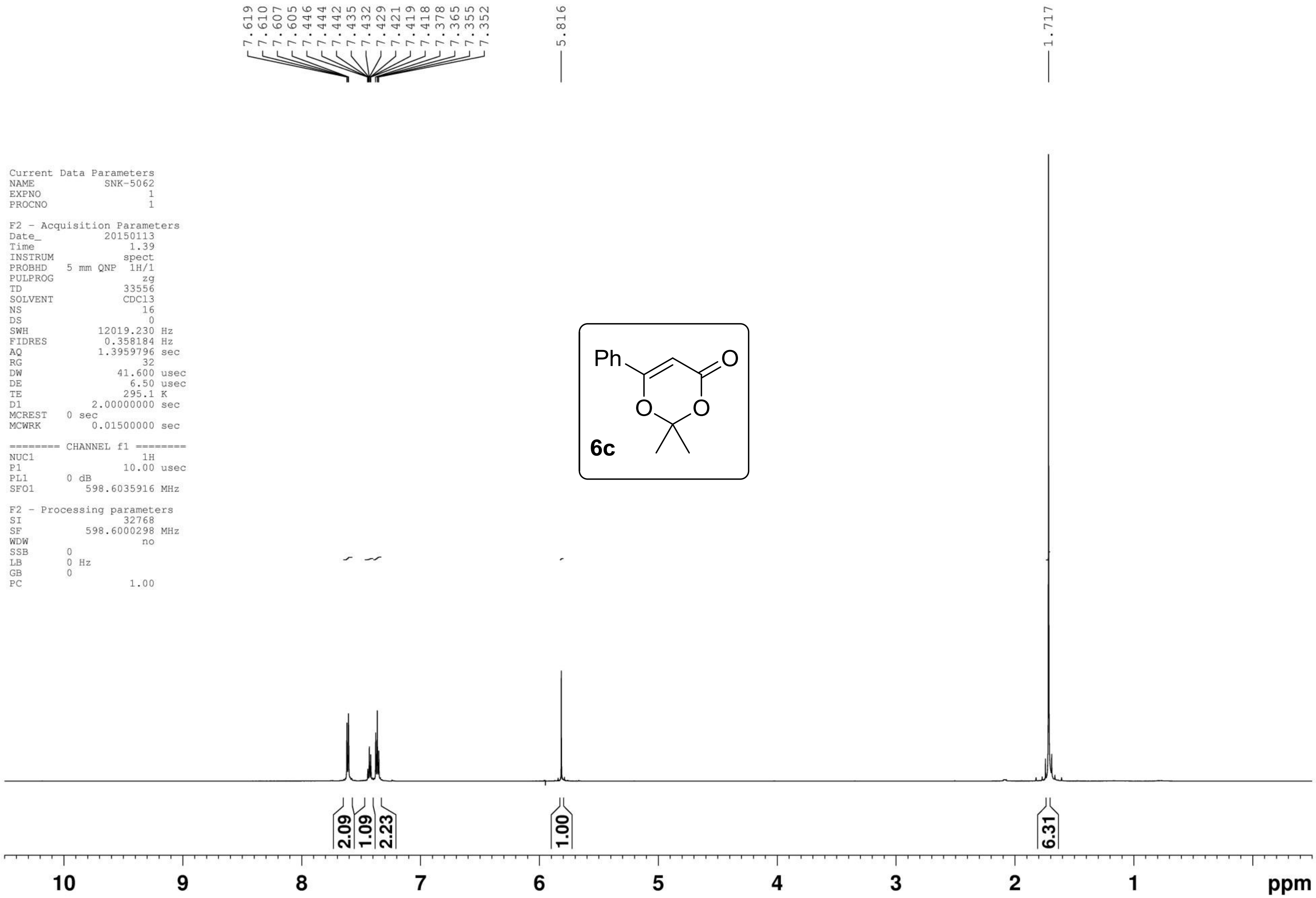

7.619
7.610
7.607
7.605
7.446
7.444
7.442
7.435
7.432
7.429
7.421
7.419
7.418
7.378
7.365
7.355
7.352
5.816
1.717
Current Data Parameters
NAME SNK-5062
EXPNO 1
PROCNO 1
F2 - Acquisition Parameters
Date_ 20150113
Time 1.39
INSTRUM spect
PROBHD 5 mm QNP 1H/1
PULPROG zg
TD 33556
SOLVENT CDCl3
NS 16
DS 0
SWH 12019.230 Hz
FIDRES 0.358184 Hz
AQ 1.3959796 sec
RG 32
DW 41.600 usec
DE 6.50 usec
TE 295.1 K
D1 2.00000000 sec
MCREST 0 sec
MCWRK 0.01500000 sec
======== CHANNEL f1 ========
NUC1 1H
P1 10.00 usec
PL1 0 dB
SFO1 598.6035916 MHz
F2 - Processing parameters
SI 32768
SF 598.6000298 MHz
WDW no
SSB 0
LB 0 Hz
GB 0
PC 1.00
Ph
O
O
O
6c
2.09
1.09
2.23
1.00
6.31
10
9
8
7
6
5
4
3
2
1
ppm

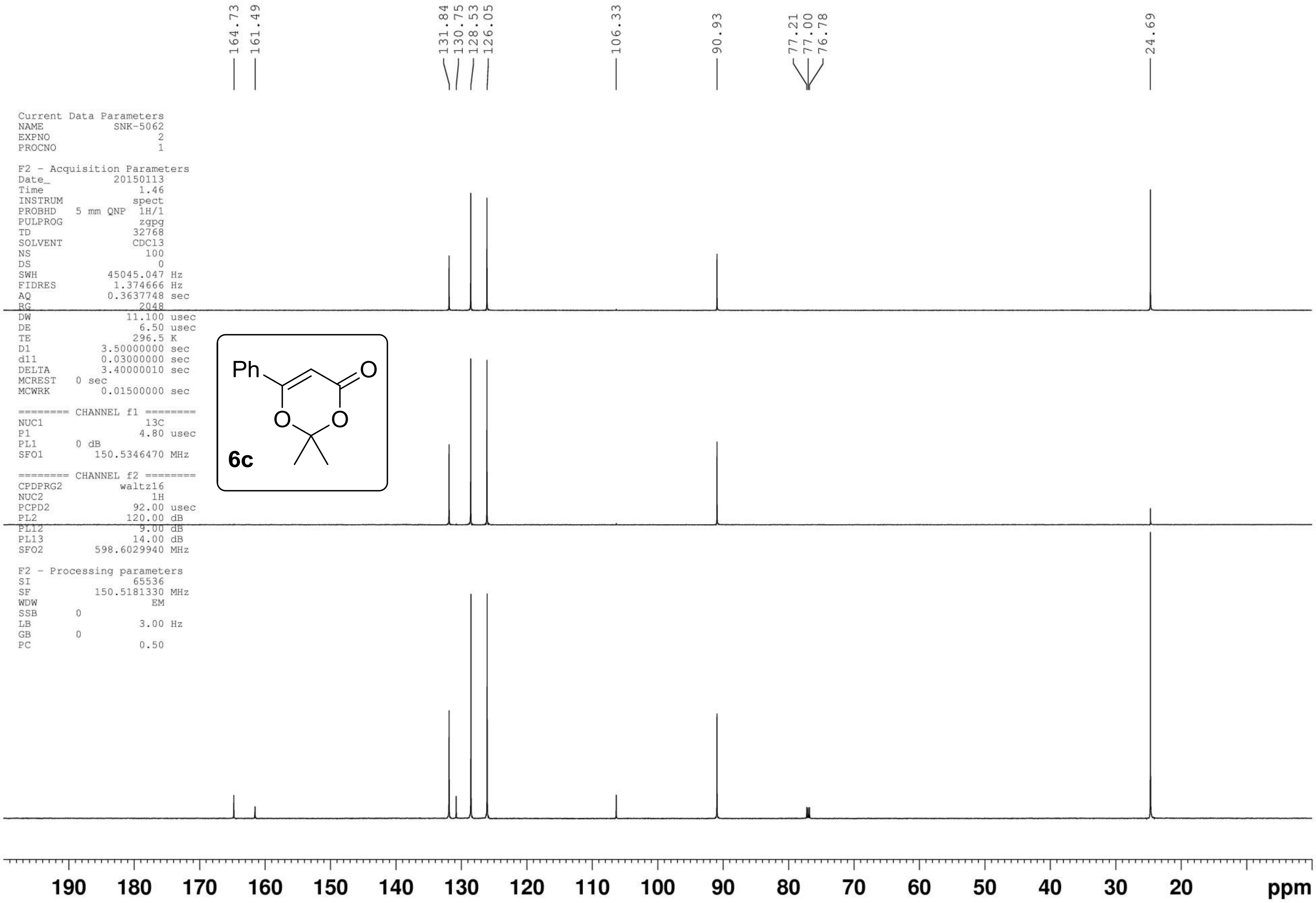
164.73
161.49
131.84
130.75
128.53
126.05
106.33
90.93
77.21
77.00
76.78
24.69
Current Data Parameters
NAME SNK-5062
EXPNO 2
PROCNO 1
F2 - Acquisition Parameters
Date_ 20150113
Time 1.46
INSTRUM spect
PROBHD 5 mm QNP 1H/1
PULPROG zgpg
TD 32768
SOLVENT CDCl3
NS 100
DS 0
SWH 45045.047 Hz
FIDRES 1.374666 Hz
AQ 0.3637748 sec
RG 2048
DW 11.100 usec
DE 6.50 usec
TE 296.5 K
D1 3.50000000 sec
d11 0.03000000 sec
DELTA 3.40000010 sec
MCREST 0 sec
MCWRK 0.01500000 sec
======== CHANNEL f1 ========
NUC1 13C
P1 4.80 usec
PL1 0 dB
SFO1 150.5346470 MHz
======== CHANNEL f2 ========
CPDPRG2 waltz16
NUC2 1H
PCPD2 92.00 usec
PL2 120.00 dB
PL12 9.00 dB
PL13 14.00 dB
SFO2 598.6029940 MHz
F2 - Processing parameters
SI 65536
SF 150.5181330 MHz
WDW EM
SSB 0
LB 3.00 Hz
GB 0
PC 0.50
Ph
O
O
O
6c
190 180 170 160 150 140 130 120 110 100 90 80 70 60 50 40 30 20 ppm

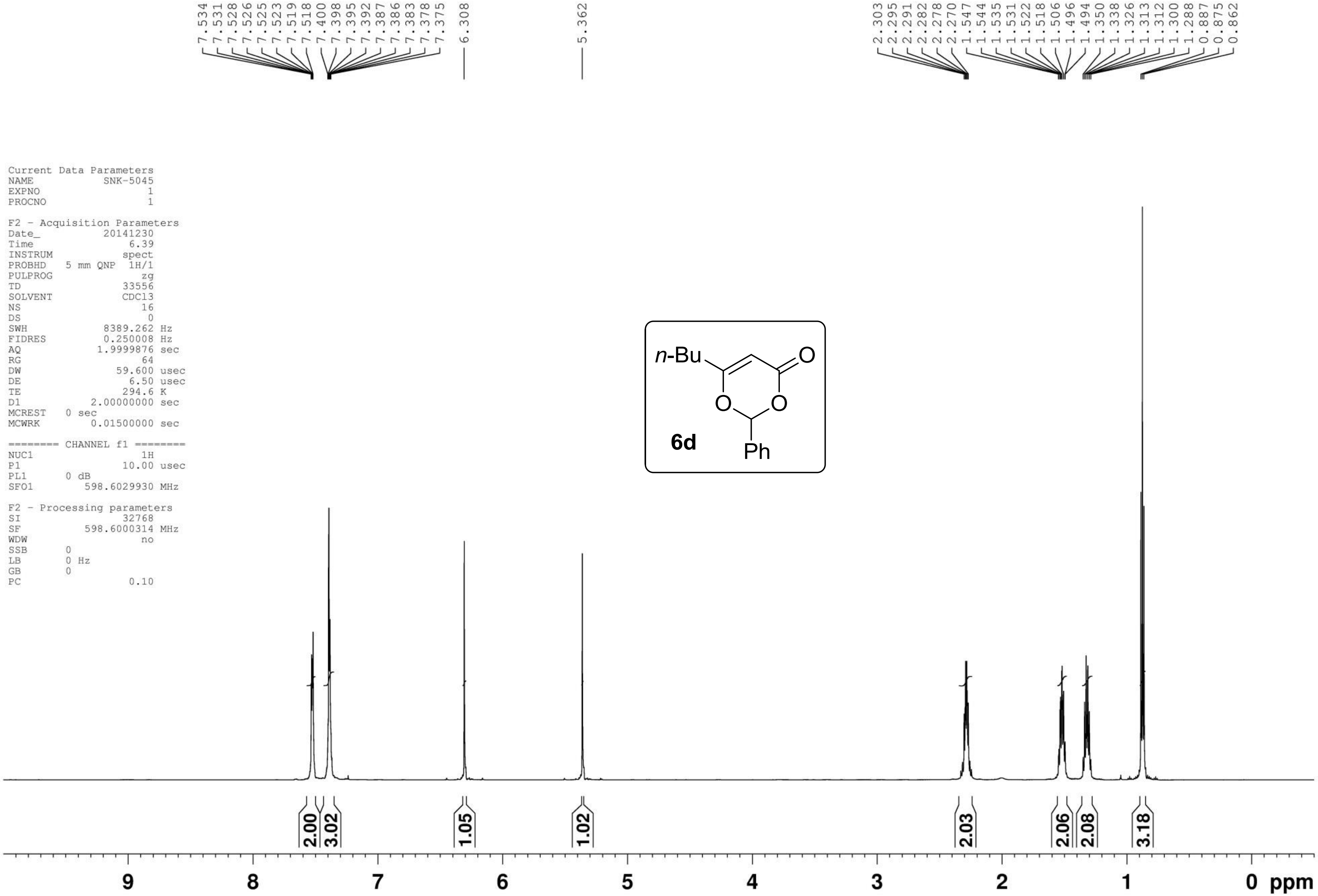
7.534
7.531
7.528
7.526
7.525
7.523
7.519
7.518
7.400
7.398
7.395
7.392
7.387
7.386
7.383
7.378
7.375
6.308
5.362
2.303
2.295
2.291
2.282
2.278
2.270
1.547
1.544
1.535
1.531
1.522
1.518
1.506
1.496
1.494
1.350
1.338
1.326
1.313
1.312
1.300
1.288
0.887
0.875
0.862
Current Data Parameters
NAME SNK-5045
EXPNO 1
PROCNO 1
F2 - Acquisition Parameters
Date_ 20141230
Time 6.39
INSTRUM spect
PROBHD 5 mm QNP 1H/1
PULPROG zg
TD 33556
SOLVENT CDCl3
NS 16
DS 0
SWH 8389.262 Hz
FIDRES 0.250008 Hz
AQ 1.9999876 sec
RG 64
DW 59.600 usec
DE 6.50 usec
TE 294.6 K
D1 2.00000000 sec
MCREST 0 sec
MCWRK 0.01500000 sec
======== CHANNEL f1 ========
NUC1 1H
P1 10.00 usec
PL1 0 dB
SFO1 598.6029930 MHz
F2 - Processing parameters
SI 32768
SF 598.6000314 MHz
WDW no
SSB 0
LB 0 Hz
GB 0
PC 0.10
n-Bu
O
O
O
6d
Ph
2.00
3.02
1.05
1.02
2.03
2.06
2.08
3.18
9
8
7
6
5
4
3
2
1
0 ppm

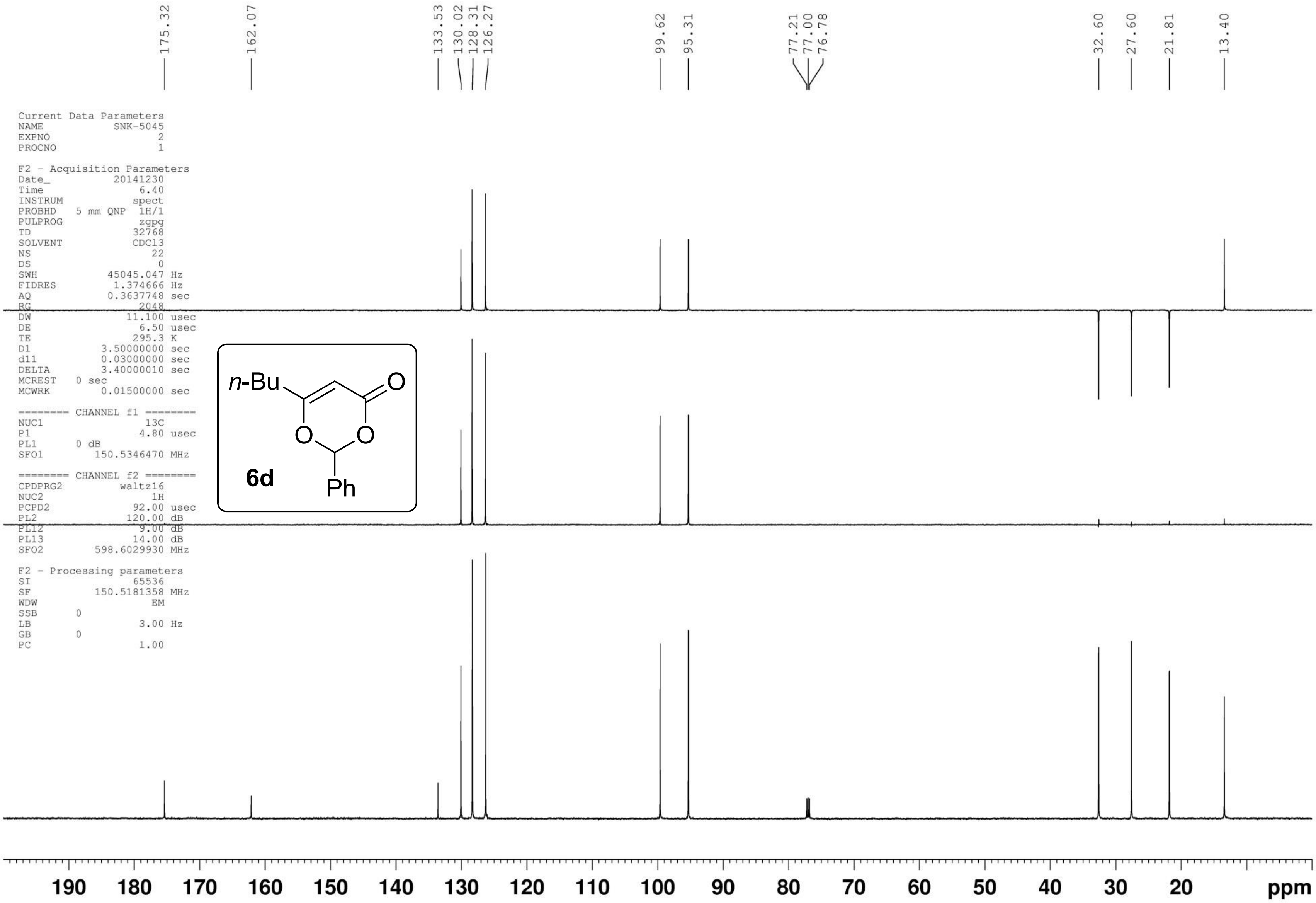
175.32
162.07
133.53
130.02
128.31
126.27
99.62
95.31
77.21
77.00
76.78
32.60
27.60
21.81
13.40
Current Data Parameters
NAME SNK-5045
EXPNO 2
PROCNO 1
F2 - Acquisition Parameters
Date_ 20141230
Time 6.40
INSTRUM spect
PROBHD 5 mm QNP 1H/1
PULPROG zgpg
TD 32768
SOLVENT CDCl3
NS 22
DS 0
SWH 45045.047 Hz
FIDRES 1.374666 Hz
AQ 0.3637748 sec
RG 2048
DW 11.100 usec
DE 6.50 usec
TE 295.3 K
D1 3.50000000 sec
d11 0.03000000 sec
DELTA 3.40000010 sec
MCREST 0 sec
MCWRK 0.01500000 sec
======== CHANNEL f1 ========
NUC1 13C
P1 4.80 usec
PL1 0 dB
SFO1 150.5346470 MHz
======== CHANNEL f2 ========
CPDPRG2 waltz16
NUC2 1H
PCPD2 92.00 usec
PL2 120.00 dB
PL12 9.00 dB
PL13 14.00 dB
SFO2 598.6029930 MHz
F2 - Processing parameters
SI 65536
SF 150.5181358 MHz
WDW EM
SSB 0
LB 3.00 Hz
GB 0
PC 1.00
n-Bu
O
O
O
Ph
6d
190 180 170 160 150 140 130 120 110 100 90 80 70 60 50 40 30 20 ppm

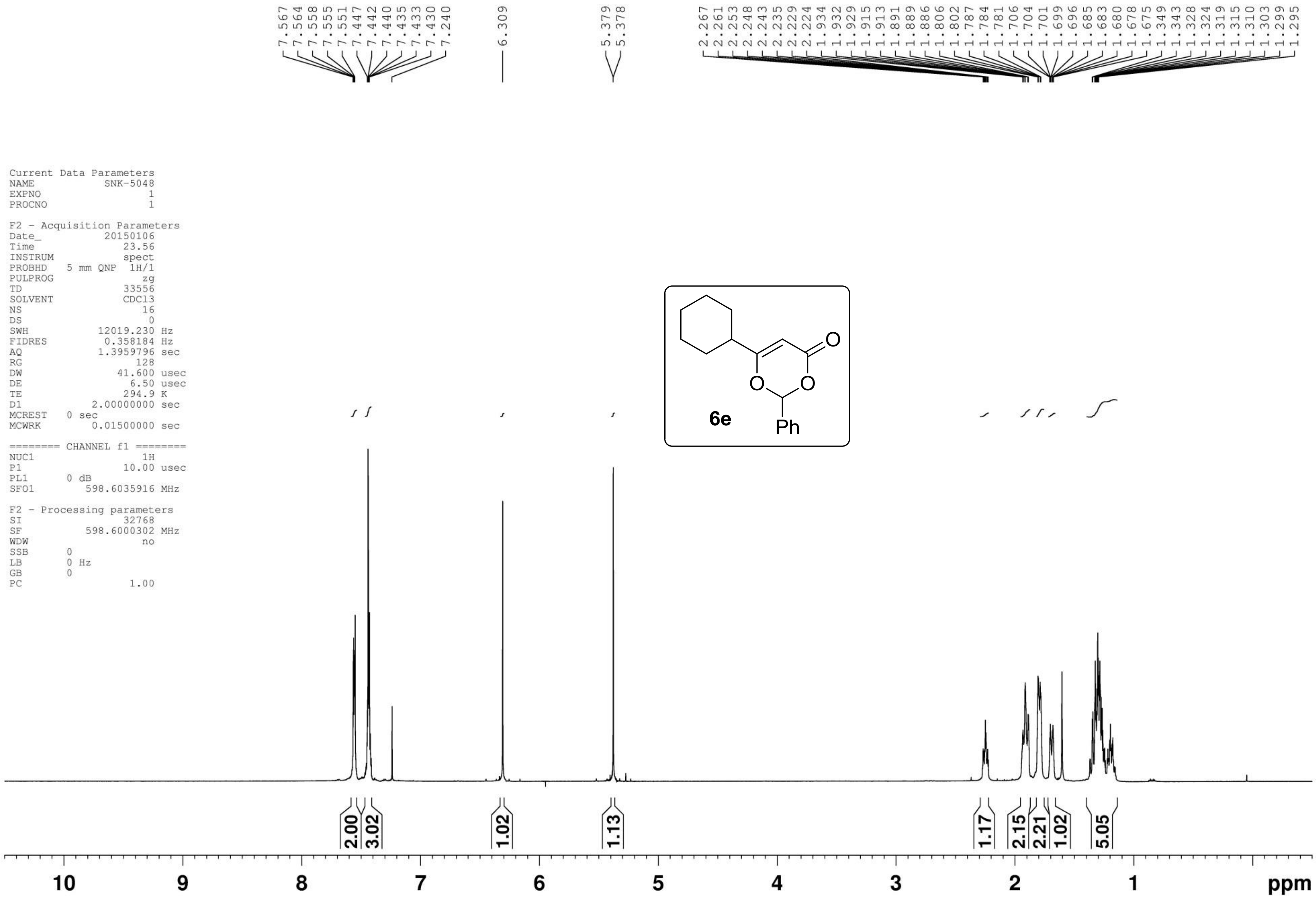

Current Data Parameters
NAME SNK-5048
EXPNO 1
PROCNO 1
F2 - Acquisition Parameters
Date_ 20150106
Time 23.56
INSTRUM spect
PROBHD 5 mm QNP 1H/1
PULPROG zg
TD 33556
SOLVENT CDCl3
NS 16
DS 0
SWH 12019.230 Hz
FIDRES 0.358184 Hz
AQ 1.3959796 sec
RG 128
DW 41.600 usec
DE 6.50 usec
TE 294.9 K
D1 2.00000000 sec
MCREST 0 sec
MCWRK 0.01500000 sec
======== CHANNEL f1 ========
NUC1 1H
P1 10.00 usec
PL1 0 dB
SFO1 598.6035916 MHz
F2 - Processing parameters
SI 32768
SF 598.6000302 MHz
WDW no
SSB 0
LB 0 Hz
GB 0
PC 1.00
7.567 7.564 7.558 7.555 7.551 7.447 7.442 7.440 7.435 7.433 7.430 7.240
6.309
5.379 5.378
2.267 2.261 2.253 2.248 2.243 2.235 2.229 2.224 1.934 1.932 1.929 1.915 1.913 1.891 1.889 1.886 1.806 1.802 1.787 1.784 1.781 1.706 1.704 1.701 1.699 1.696 1.685 1.683 1.680 1.678 1.675 1.349 1.343 1.328 1.324 1.319 1.315 1.310 1.303 1.299 1.295
O
O
O
6e
Ph
2.00
3.02
1.02
1.13
1.17
2.15
2.21
1.02
5.05
10
9
8
7
6
5
4
3
2
1
ppm

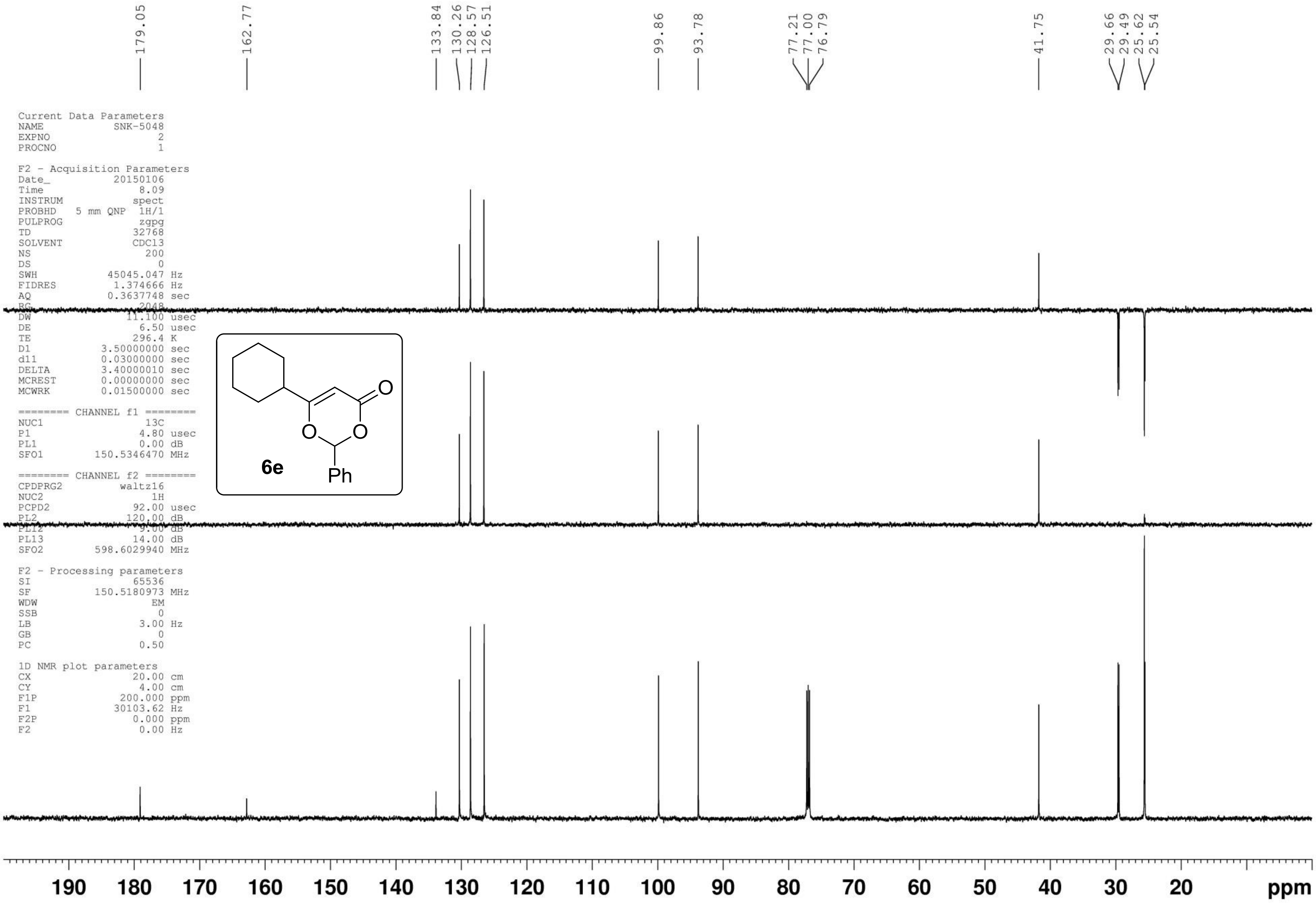
179.05
162.77
133.84
130.26
128.57
126.51
99.86
93.78
77.21
77.00
76.79
41.75
29.66
29.49
25.62
25.54
Current Data Parameters
NAME SNK-5048
EXPNO 2
PROCNO 1
F2 - Acquisition Parameters
Date_ 20150106
Time 8.09
INSTRUM spect
PROBHD 5 mm QNP 1H/1
PULPROG zgpg
TD 32768
SOLVENT CDCl3
NS 200
DS 0
SWH 45045.047 Hz
FIDRES 1.374666 Hz
AQ 0.3637748 sec
RG 2048
DW 11.100 usec
DE 6.50 usec
TE 296.4 K
D1 3.50000000 sec
d11 0.03000000 sec
DELTA 3.40000010 sec
MCREST 0.00000000 sec
MCWRK 0.01500000 sec
======== CHANNEL f1 ========
NUC1 13C
P1 4.80 usec
PL1 0.00 dB
SFO1 150.5346470 MHz
======== CHANNEL f2 ========
CPDPRG2 waltz16
NUC2 1H
PCPD2 92.00 usec
PL2 120.00 dB
PL12 9.00 dB
PL13 14.00 dB
SFO2 598.6029940 MHz
F2 - Processing parameters
SI 65536
SF 150.5180973 MHz
WDW EM
SSB 0
LB 3.00 Hz
GB 0
PC 0.50
1D NMR plot parameters
CX 20.00 cm
CY 4.00 cm
F1P 200.000 ppm
F1 30103.62 Hz
F2P 0.000 ppm
F2 0.00 Hz
O
O
O
6e
Ph
190 180 170 160 150 140 130 120 110 100 90 80 70 60 50 40 30 20 ppm

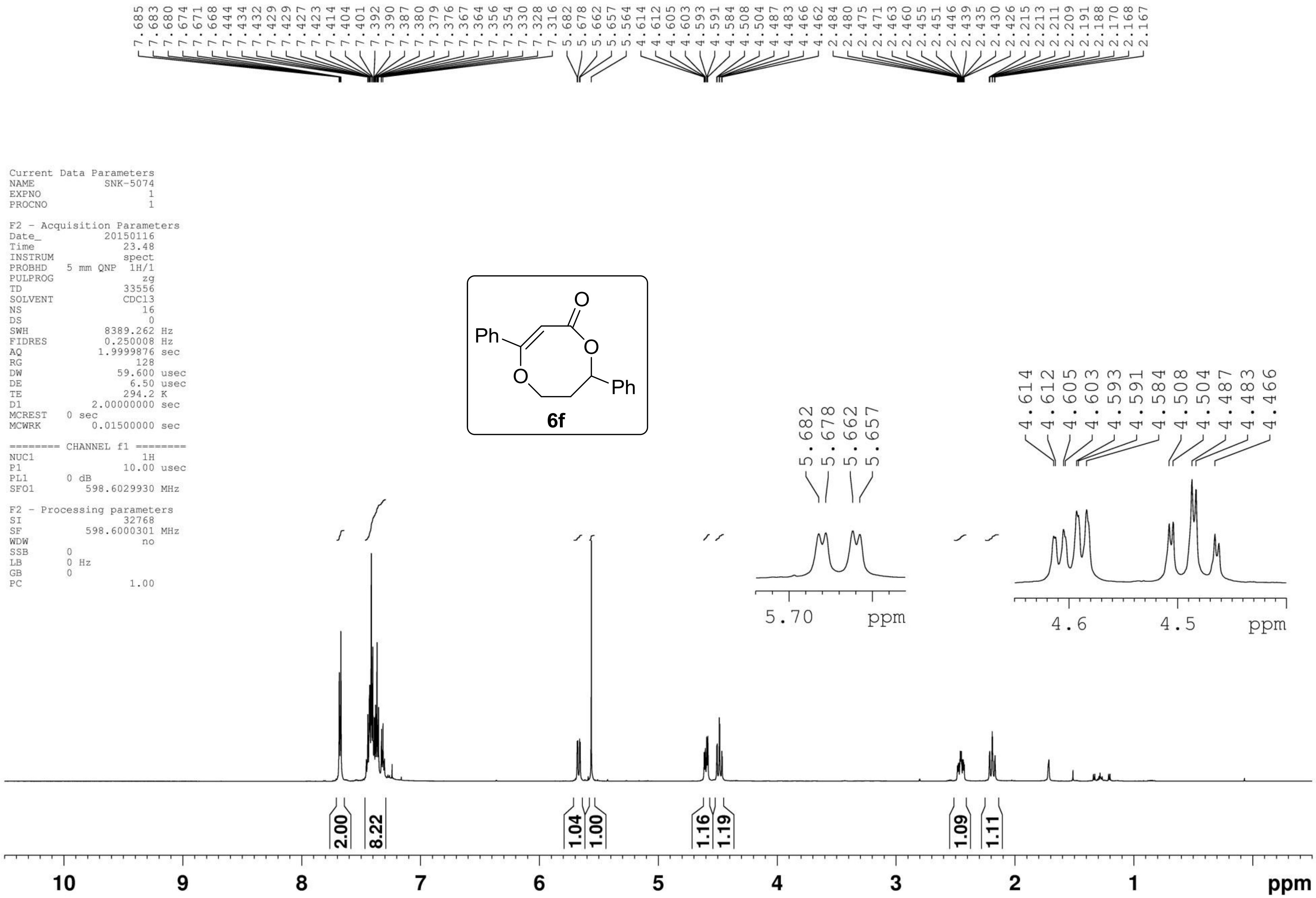

7.685 7.683 7.680 7.674 7.671 7.668 7.444 7.434 7.432 7.429 7.429 7.427 7.423 7.414 7.404 7.401 7.392 7.390 7.387 7.380 7.379 7.376 7.367 7.364 7.356 7.354 7.330 7.328 7.316 5.682 5.678 5.662 5.657 5.564 4.614 4.612 4.605 4.603 4.593 4.591 4.584 4.508 4.504 4.487 4.483 4.466 4.462 2.484 2.480 2.475 2.471 2.463 2.460 2.455 2.451 2.446 2.439 2.435 2.430 2.426 2.215 2.213 2.211 2.209 2.191 2.188 2.170 2.168 2.167
Current Data Parameters
NAME SNK-5074
EXPNO 1
PROCNO 1
F2 - Acquisition Parameters
Date_ 20150116
Time 23.48
INSTRUM spect
PROBHD 5 mm QNP 1H/1
PULPROG zg
TD 33556
SOLVENT CDCl3
NS 16
DS 0
SWH 8389.262 Hz
FIDRES 0.250008 Hz
AQ 1.9999876 sec
RG 128
DW 59.600 usec
DE 6.50 usec
TE 294.2 K
D1 2.00000000 sec
MCREST 0 sec
MCWRK 0.01500000 sec
======== CHANNEL f1 ========
NUC1 1H
P1 10.00 usec
PL1 0 dB
SFO1 598.6029930 MHz
F2 - Processing parameters
SI 32768
SF 598.6000301 MHz
WDW no
SSB 0
LB 0 Hz
GB 0
PC 1.00
Ph
O
O
O
Ph
6f
5.682 5.678 5.662 5.657
5.70 ppm
4.614 4.612 4.605 4.603 4.593 4.591 4.584 4.508 4.504 4.487 4.483 4.466
4.6 4.5 ppm
2.00 8.22 1.04 1.00 1.16 1.19 1.09 1.11
10 9 8 7 6 5 4 3 2 1 ppm

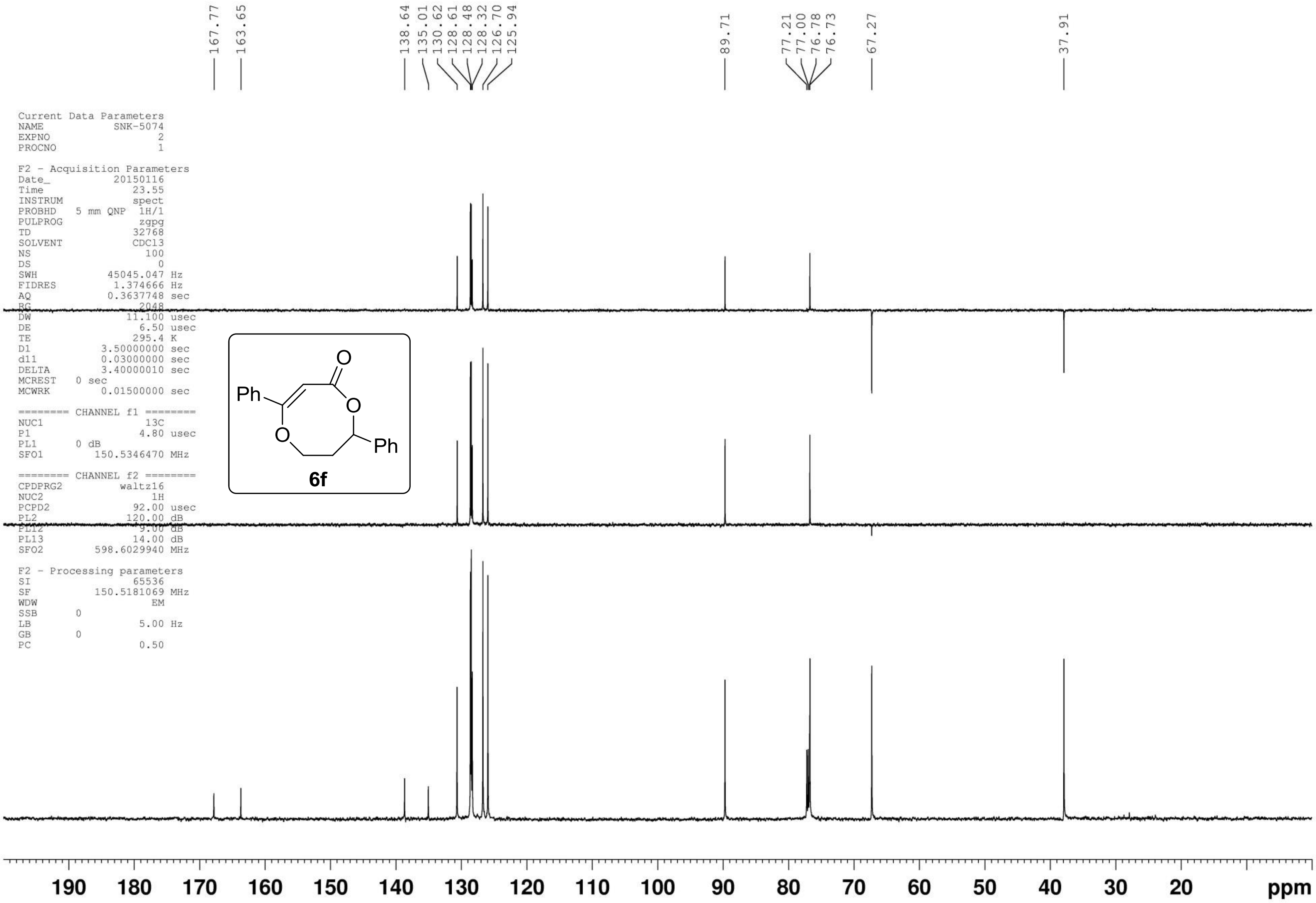
167.77
163.65
138.64
135.01
130.62
128.61
128.48
128.32
126.70
125.94
89.71
77.21
77.00
76.78
76.73
67.27
37.91
Current Data Parameters
NAME SNK-5074
EXPNO 2
PROCNO 1
F2 - Acquisition Parameters
Date_ 20150116
Time 23.55
INSTRUM spect
PROBHD 5 mm QNP 1H/1
PULPROG zgpg
TD 32768
SOLVENT CDCl3
NS 100
DS 0
SWH 45045.047 Hz
FIDRES 1.374666 Hz
AQ 0.3637748 sec
RG 2048
DW 11.100 usec
DE 6.50 usec
TE 295.4 K
D1 3.50000000 sec
d11 0.03000000 sec
DELTA 3.40000010 sec
MCREST 0 sec
MCWRK 0.01500000 sec
======== CHANNEL f1 ========
NUC1 13C
P1 4.80 usec
PL1 0 dB
SFO1 150.5346470 MHz
======== CHANNEL f2 ========
CPDPRG2 waltz16
NUC2 1H
PCPD2 92.00 usec
PL2 120.00 dB
PL12 9.00 dB
PL13 14.00 dB
SFO2 598.6029940 MHz
F2 - Processing parameters
SI 65536
SF 150.5181069 MHz
WDW EM
SSB 0
LB 5.00 Hz
GB 0
PC 0.50
Ph
O
O
O
Ph
6f
190 180 170 160 150 140 130 120 110 100 90 80 70 60 50 40 30 20 ppm

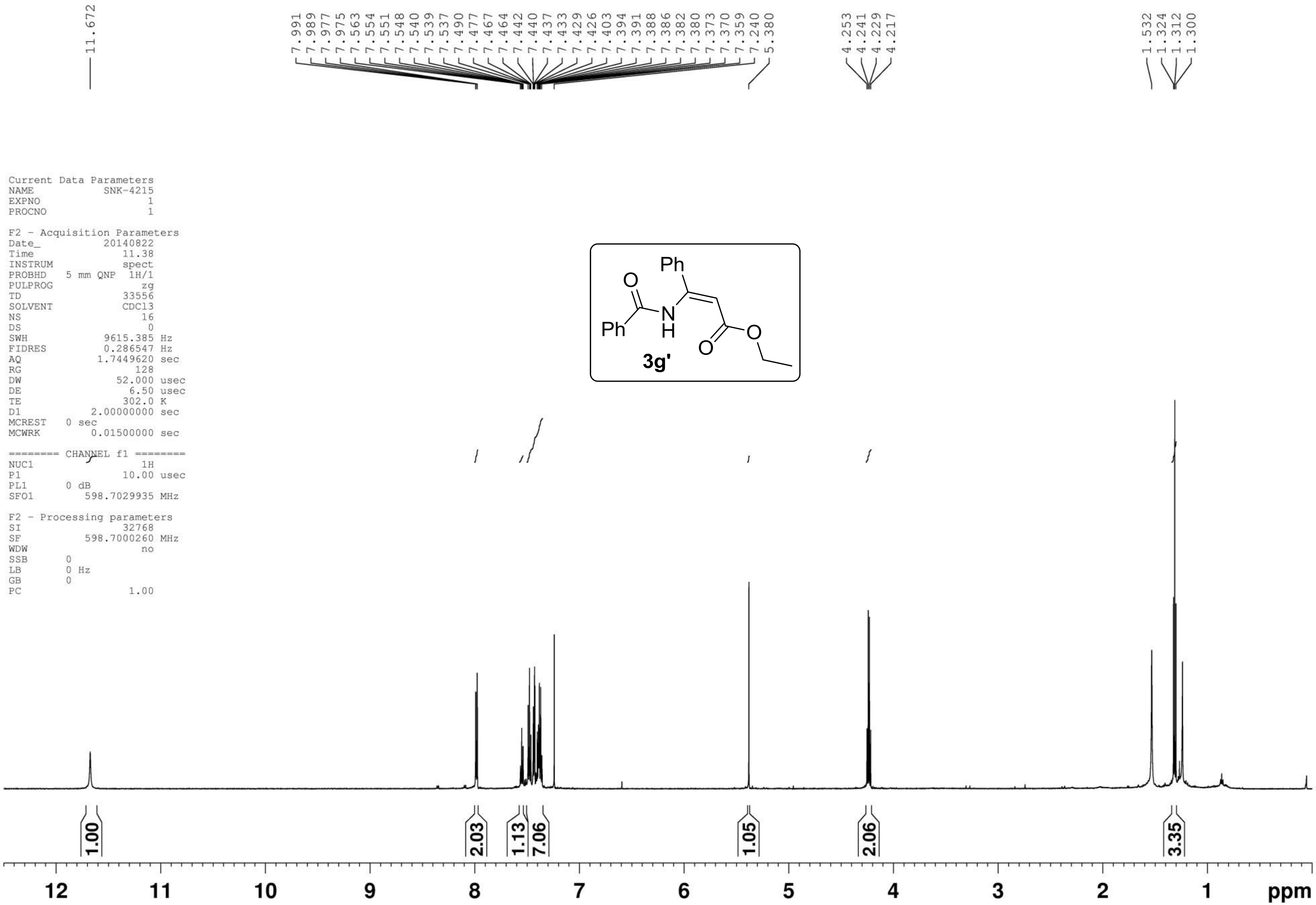
11.672
7.991
7.989
7.977
7.975
7.563
7.554
7.551
7.548
7.540
7.539
7.537
7.490
7.477
7.467
7.464
7.442
7.440
7.437
7.433
7.429
7.426
7.403
7.394
7.391
7.388
7.386
7.382
7.380
7.373
7.370
7.359
7.240
5.380
4.253
4.241
4.229
4.217
1.532
1.324
1.312
1.300
Current Data Parameters
NAME SNK-4215
EXPNO 1
PROCNO 1
F2 - Acquisition Parameters
Date_ 20140822
Time 11.38
INSTRUM spect
PROBHD 5 mm QNP 1H/1
PULPROG zg
TD 33556
SOLVENT CDCl3
NS 16
DS 0
SWH 9615.385 Hz
FIDRES 0.286547 Hz
AQ 1.7449620 sec
RG 128
DW 52.000 usec
DE 6.50 usec
TE 302.0 K
D1 2.00000000 sec
MCREST 0 sec
MCWRK 0.01500000 sec
======== CHANNEL f1 ========
NUC1 1H
P1 10.00 usec
PL1 0 dB
SFO1 598.7029935 MHz
F2 - Processing parameters
SI 32768
SF 598.7000260 MHz
WDW no
SSB 0
LB 0 Hz
GB 0
PC 1.00
Ph
O
N
H
Ph
O
O
3g'
1.00
2.03
1.13
7.06
1.05
2.06
3.35
12
11
10
9
8
7
6
5
4
3
2
1
ppm

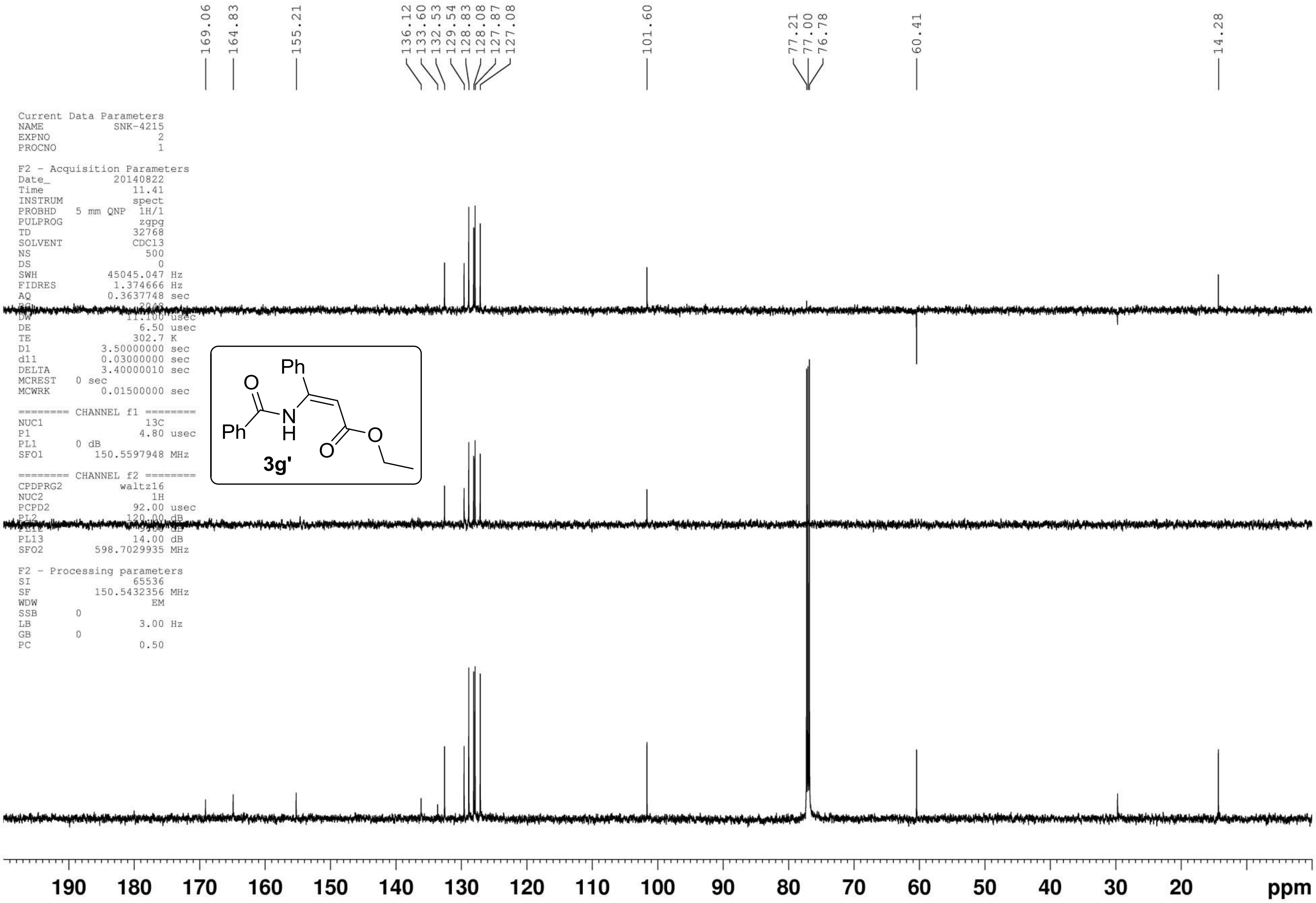

169.06
164.83
155.21
136.12
133.60
132.53
129.54
128.83
128.08
127.87
127.08
101.60
77.21
77.00
76.78
60.41
14.28
Current Data Parameters
NAME SNK-4215
EXPNO 2
PROCNO 1
F2 - Acquisition Parameters
Date_ 20140822
Time 11.41
INSTRUM spect
PROBHD 5 mm QNP 1H/1
PULPROG zgpg
TD 32768
SOLVENT CDCl3
NS 500
DS 0
SWH 45045.047 Hz
FIDRES 1.374666 Hz
AQ 0.3637748 sec
DW 11.100 usec
DE 6.50 usec
TE 302.7 K
D1 3.50000000 sec
d11 0.03000000 sec
DELTA 3.40000010 sec
MCREST 0 sec
MCWRK 0.01500000 sec
======== CHANNEL f1 ========
NUC1 13C
P1 4.80 usec
PL1 0 dB
SFO1 150.5597948 MHz
======== CHANNEL f2 ========
CPDPRG2 waltz16
NUC2 1H
PCPD2 92.00 usec
PL13 14.00 dB
SFO2 598.7029935 MHz
F2 - Processing parameters
SI 65536
SF 150.5432356 MHz
WDW EM
SSB 0
LB 3.00 Hz
GB 0
PC 0.50
Ph
O
N
H
Ph
O
O
3g'
190
180
170
160
150
140
130
120
110
100
90
80
70
60
50
40
30
20
ppm

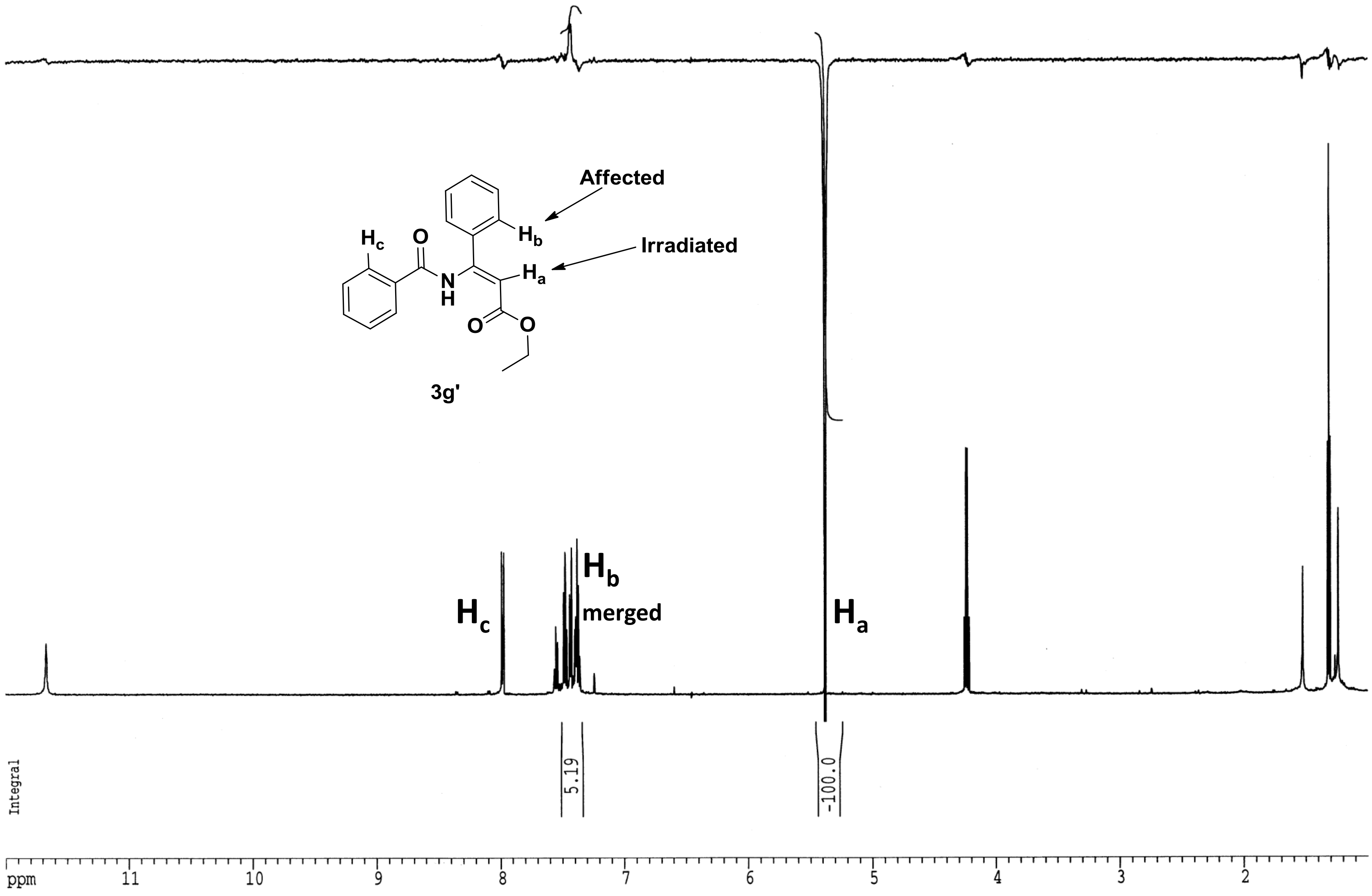

Affected
Irradiated
3g'
Hc
Hb
merged
Ha
5.19
-100.0
Integral
ppm
11
10
9
8
7
6
5
4
3
2

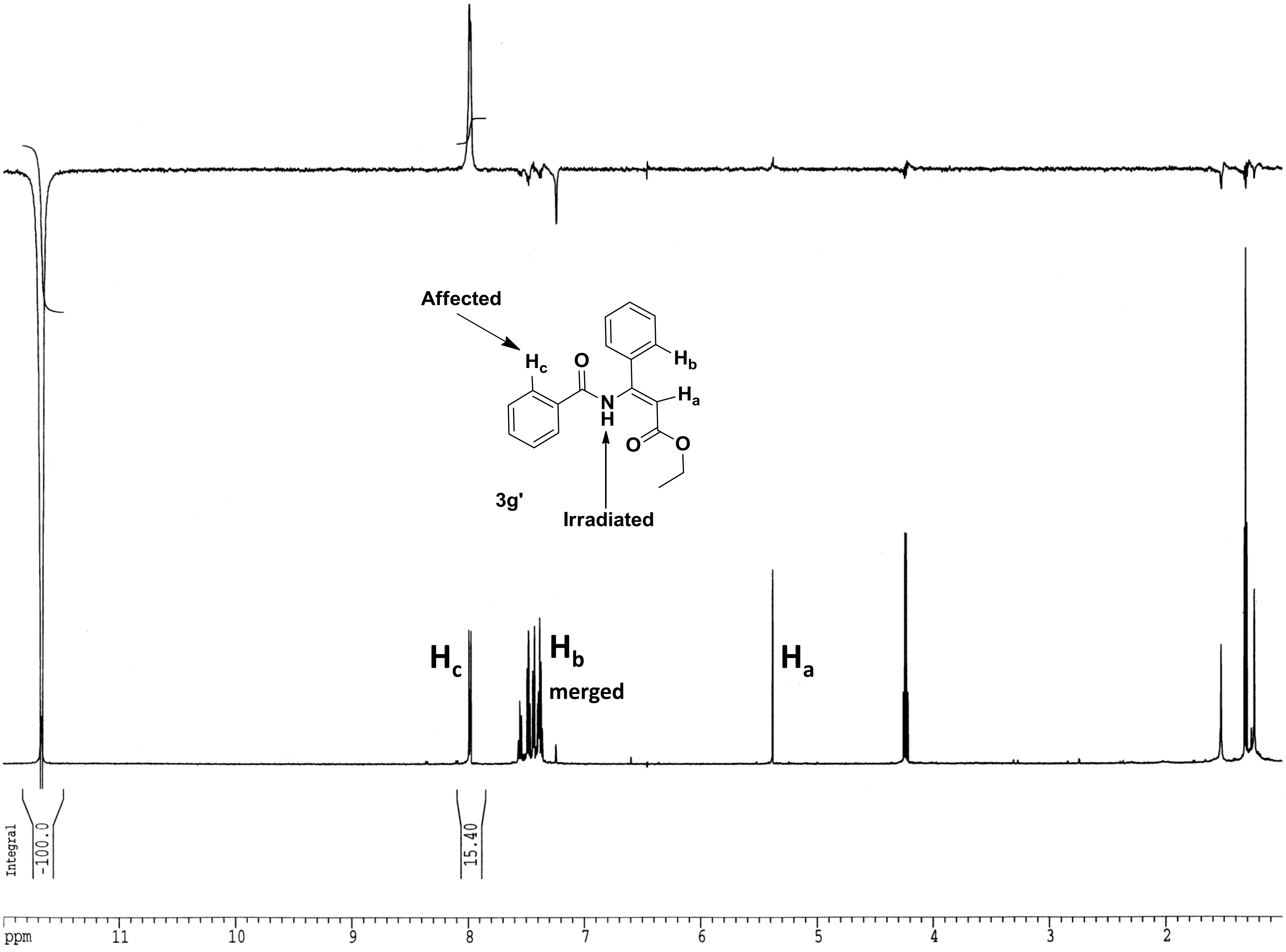
Affected
Hc
O
Hb
Ha
N
H
O
O
3g'
Irradiated
Hc
Hb
merged
Ha
Integral
-100.0
15.40
ppm
11
10
9
8
7
6
5
4
3
2

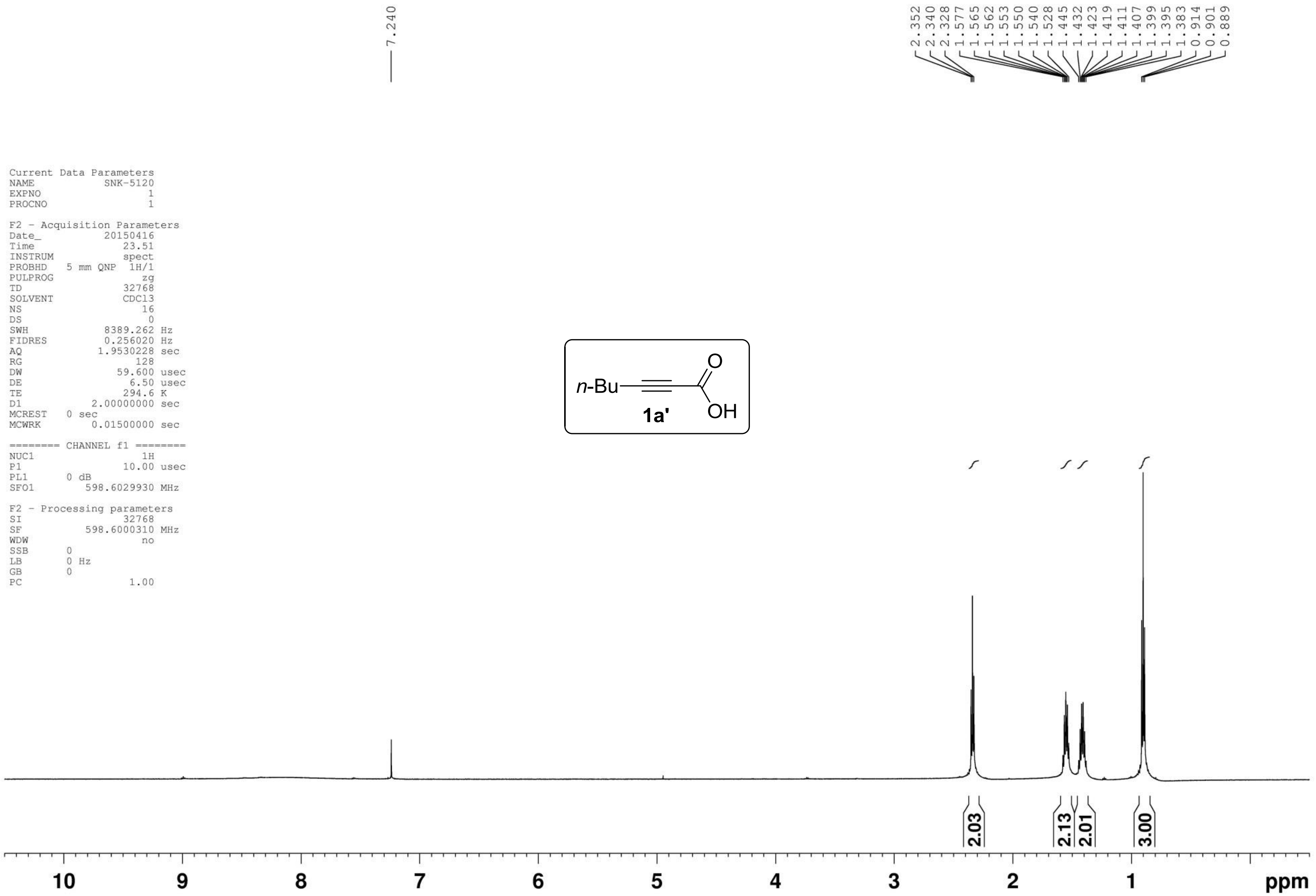
7.240
2.352
2.340
2.328
1.577
1.565
1.562
1.553
1.550
1.540
1.528
1.445
1.432
1.423
1.419
1.411
1.407
1.399
1.395
1.383
0.914
0.901
0.889
Current Data Parameters
NAME SNK-5120
EXPNO 1
PROCNO 1
F2 - Acquisition Parameters
Date_ 20150416
Time 23.51
INSTRUM spect
PROBHD 5 mm QNP 1H/1
PULPROG zg
TD 32768
SOLVENT CDCl3
NS 16
DS 0
SWH 8389.262 Hz
FIDRES 0.256020 Hz
AQ 1.9530228 sec
RG 128
DW 59.600 usec
DE 6.50 usec
TE 294.6 K
D1 2.00000000 sec
MCREST 0 sec
MCWRK 0.01500000 sec
======== CHANNEL f1 ========
NUC1 1H
P1 10.00 usec
PL1 0 dB
SFO1 598.6029930 MHz
F2 - Processing parameters
SI 32768
SF 598.6000310 MHz
WDW no
SSB 0
LB 0 Hz
GB 0
PC 1.00
n-Bu
O
OH
1a'
2.03
2.13
2.01
3.00
10
9
8
7
6
5
4
3
2
1
ppm

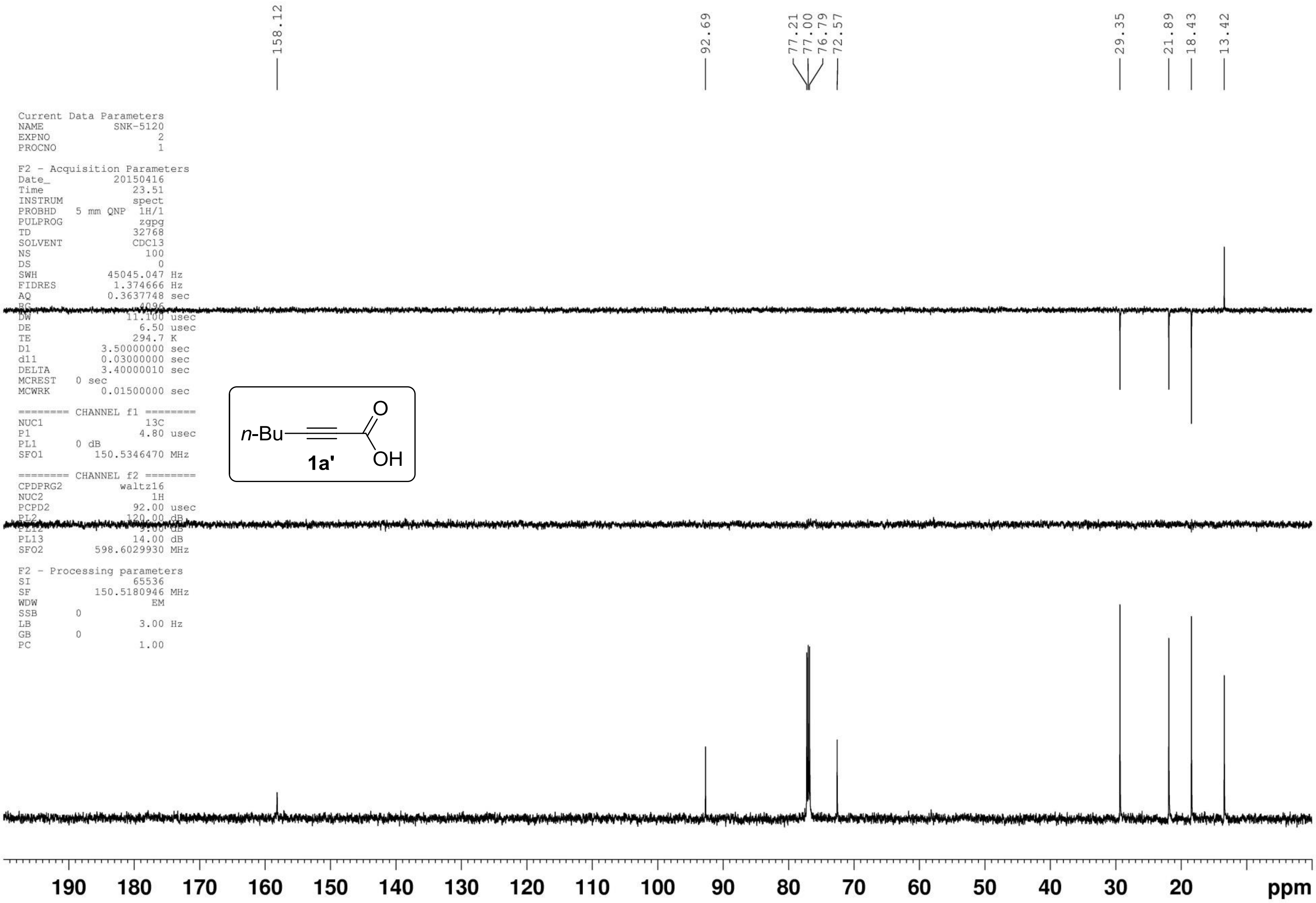

Current Data Parameters
NAME SNK-5120
EXPNO 2
PROCNO 1

F2 - Acquisition Parameters
Date_ 20150416
Time 23.51
INSTRUM spect
PROBHD 5 mm QNP 1H/1
PULPROG zgpg
TD 32768
SOLVENT CDCl3
NS 100
DS 0
SWH 45045.047 Hz
FIDRES 1.374666 Hz
AQ 0.3637748 sec
RG 4096
DW 11.100 usec
DE 6.50 usec
TE 294.7 K
D1 3.50000000 sec
d11 0.03000000 sec
DELTA 3.40000010 sec
MCREST 0 sec
MCWRK 0.01500000 sec

======== CHANNEL f1 ========
NUC1 13C
P1 4.80 usec
PL1 0 dB
SFO1 150.5346470 MHz

======== CHANNEL f2 ========
CPDPRG2 waltz16
NUC2 1H
PCPD2 92.00 usec
PL2 120.00 dB
PL12 [illegible] dB
PL13 14.00 dB
SFO2 598.6029930 MHz

F2 - Processing parameters
SI 65536
SF 150.5180946 MHz
WDW EM
SSB 0
LB 3.00 Hz
GB 0
PC 1.00

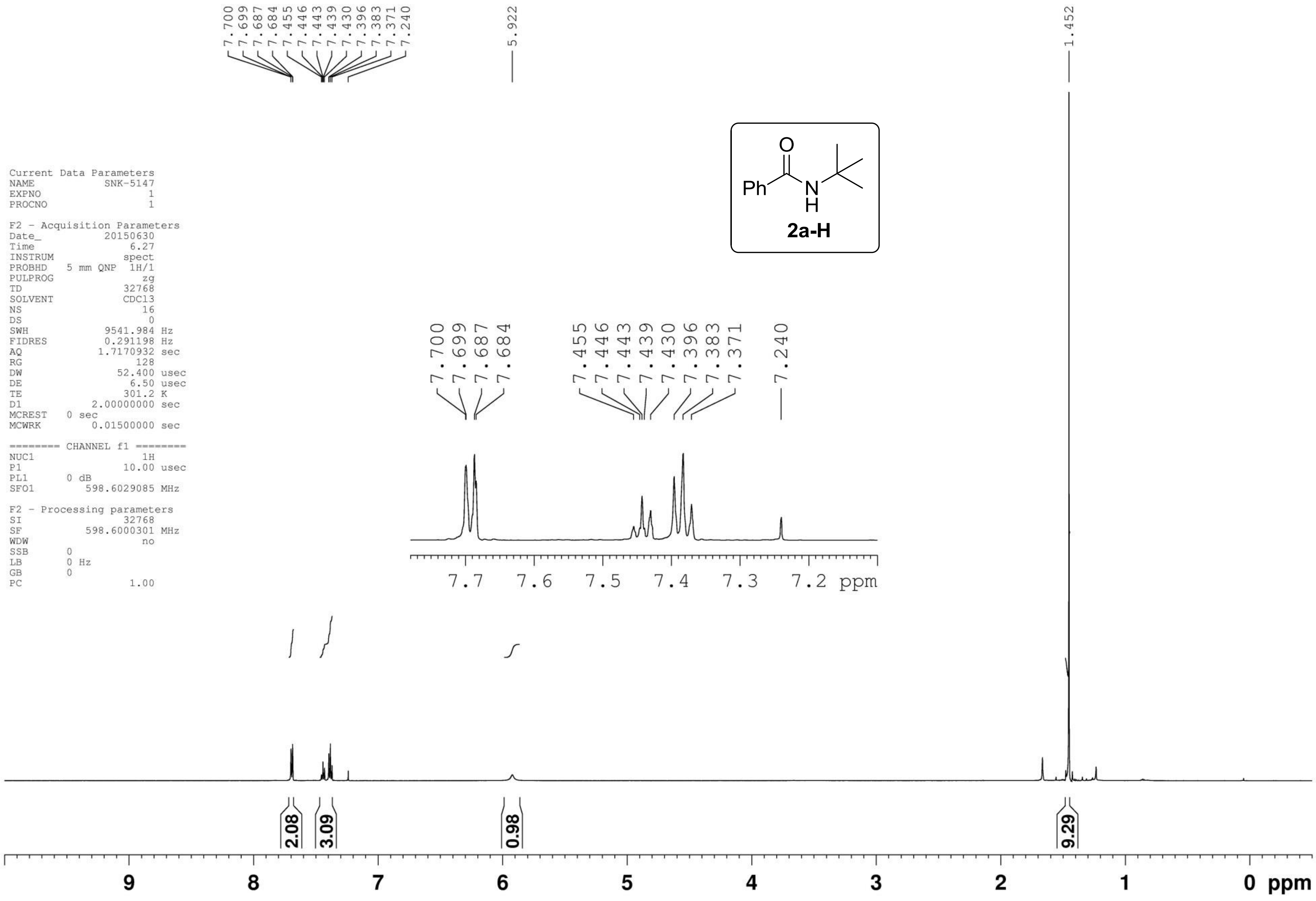
7.700
7.699
7.687
7.684
7.455
7.446
7.443
7.439
7.430
7.396
7.383
7.371
7.240
5.922
1.452
Ph
O
N
H
2a-H
Current Data Parameters
NAME SNK-5147
EXPNO 1
PROCNO 1
F2 - Acquisition Parameters
Date_ 20150630
Time 6.27
INSTRUM spect
PROBHD 5 mm QNP 1H/1
PULPROG zg
TD 32768
SOLVENT CDCl3
NS 16
DS 0
SWH 9541.984 Hz
FIDRES 0.291198 Hz
AQ 1.7170932 sec
RG 128
DW 52.400 usec
DE 6.50 usec
TE 301.2 K
D1 2.00000000 sec
MCREST 0 sec
MCWRK 0.01500000 sec
======== CHANNEL f1 ========
NUC1 1H
P1 10.00 usec
PL1 0 dB
SFO1 598.6029085 MHz
F2 - Processing parameters
SI 32768
SF 598.6000301 MHz
WDW no
SSB 0
LB 0 Hz
GB 0
PC 1.00
7.7 7.6 7.5 7.4 7.3 7.2 ppm
2.08
3.09
0.98
9.29
9 8 7 6 5 4 3 2 1 0 ppm

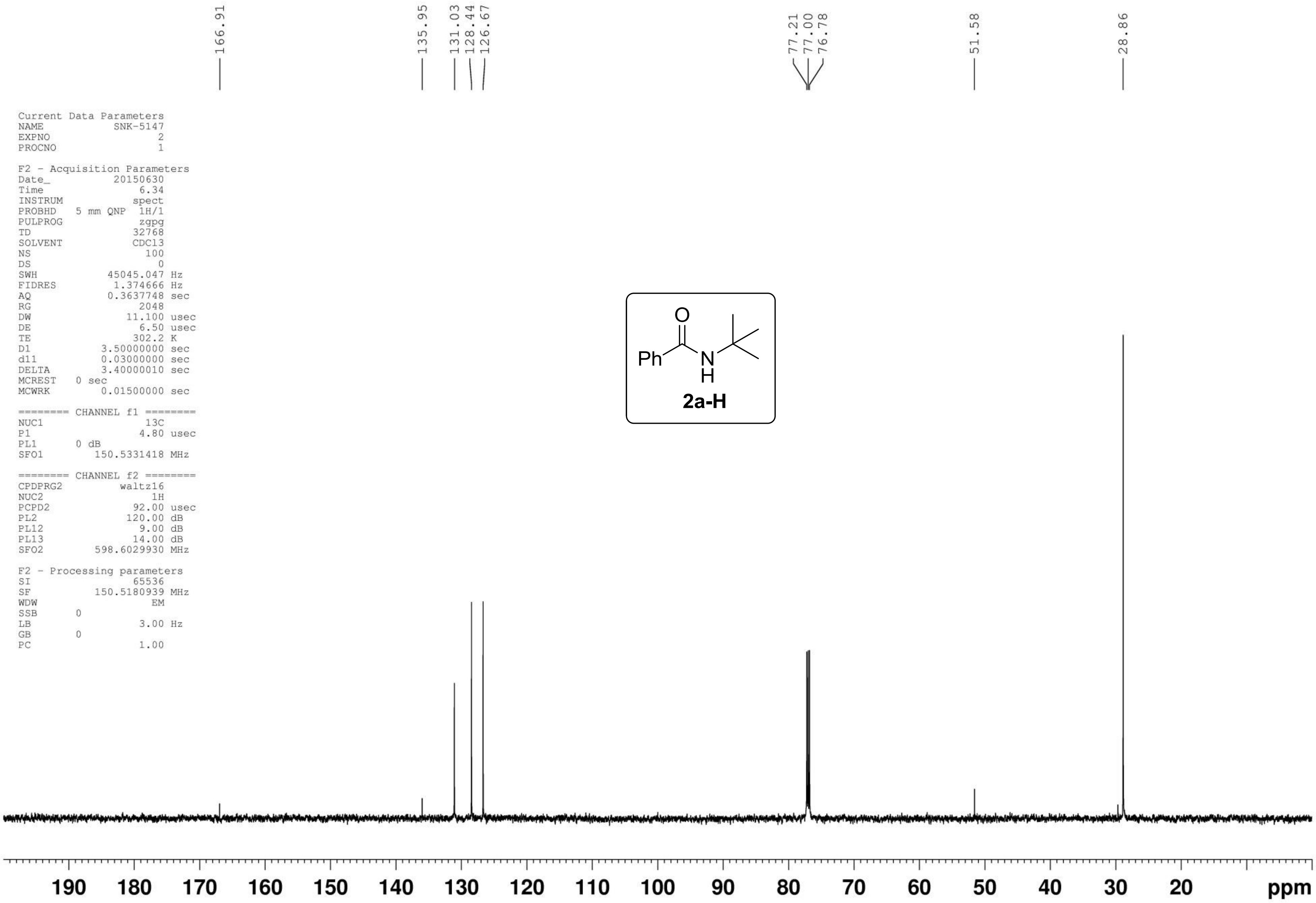
166.91
135.95
131.03
128.44
126.67
77.21
77.00
76.78
51.58
28.86
Current Data Parameters
NAME SNK-5147
EXPNO 2
PROCNO 1
F2 - Acquisition Parameters
Date_ 20150630
Time 6.34
INSTRUM spect
PROBHD 5 mm QNP 1H/1
PULPROG zgpg
TD 32768
SOLVENT CDCl3
NS 100
DS 0
SWH 45045.047 Hz
FIDRES 1.374666 Hz
AQ 0.3637748 sec
RG 2048
DW 11.100 usec
DE 6.50 usec
TE 302.2 K
D1 3.50000000 sec
d11 0.03000000 sec
DELTA 3.40000010 sec
MCREST 0 sec
MCWRK 0.01500000 sec
======== CHANNEL f1 ========
NUC1 13C
P1 4.80 usec
PL1 0 dB
SFO1 150.5331418 MHz
======== CHANNEL f2 ========
CPDPRG2 waltz16
NUC2 1H
PCPD2 92.00 usec
PL2 120.00 dB
PL12 9.00 dB
PL13 14.00 dB
SFO2 598.6029930 MHz
F2 - Processing parameters
SI 65536
SF 150.5180939 MHz
WDW EM
SSB 0
LB 3.00 Hz
GB 0
PC 1.00
Ph
O
N
H
2a-H
190 180 170 160 150 140 130 120 110 100 90 80 70 60 50 40 30 20 ppm
